# Coherent room-temperature dipole synchronization in plasmonic nanocavities

**DOI:** 10.1038/s41565-026-02242-w

**Published:** 2026-07-30

**Authors:** Rakesh Arul, Piper Fowler-Wright, Lille Børresen, Brendon W. Lovett, Jonathan Keeling, Jeremy J. Baumberg

**Affiliations:** 1 Nanophotonics Centre, Department of Physics, Cavendish Laboratory, University of Cambridge, Cambridge, UK; 2 SUPA, School of Physics and Astronomy, University of St. Andrews, St. Andrews, UK; 3 Department of Chemistry and Biochemistry, University of California San Diego, La Jolla, CA USA

**Keywords:** Nanophotonics and plasmonics, Nanophotonics and plasmonics, Quantum fluids and solids

## Abstract

Plasmonic nanocavities enable the synchronization of spatially distant emissive dipoles through strong near-field coupling in subnanometre gaps. Yet, it remains unclear how collective phase order can emerge in nanoscale optical systems where dissipation, disorder and ultrafast radiative loss normally suppress long-lived coherence. Here we report the formation of a room-temperature synchronized dipole state in locally ordered plasmonic nanogap two-dimensional arrays under non-resonant continuous-wave pumping. The system consists of methylene blue emitters confined in cucurbit[7]uril-defined subnanometre gaps between close-packed gold nanoparticles. Unlike lasers, photonic Bose–Einstein condensates or exciton–polariton condensates, this system exhibits spatial coherence across the dipoles, while rapid radiative emission suppresses temporal photon coherence. Increased coherence is observed with increasing pumping, marked by the spatial spread of *g*^(1)^ coherence, but without spectral narrowing or directional emission. This driven-dissipative system exhibits fast temporal coherence formation within 10 fs and complex spatial correlations. Combining ultralow mode volumes and high Purcell enhancement, it offers a scalable platform for studying dipole synchronization at room temperature for photonic and quantum technologies.

## Abstract (web summary)

Dipoles in self-assembled gold nanogaps synchronize at room temperature, generating spatially coherent light without laser-like narrowing.

## Main

Synchronization is ubiquitous, from starling flocks to macroscopic quantum states, and plays a pivotal role in many phenomena, including Huygens clocks, exciton–polariton condensation, lasing, superradiance and coherence in biological and chemical systems, as well as for quantum resources^1–9^. In optical dipole arrays, oscillating charges interact through electromagnetic fields. This interaction strengthens when emitters are coupled via an optical cavity. The ‘good-cavity’ limit occurs when the photon residence time exceeds the dipole lifetime. In this regime, stimulated emission dominates to produce phase-locked lasing and sharp spectral output. Conversely, the ‘bad-cavity’ limit couples many narrow-linewidth emitters through a lossy cavity. This regime shares a kinship with Huygens clocks but remains largely unexplored^10,11^.

Traditional microcavities typically operate in the good-cavity limit. These systems support exciton–polariton^12,13^, photon^14^ and plasmonic^15,16^ Bose–Einstein condensation^17^, as well as parametric oscillation^18^. Such microcavity polaritons enable applications in switching^19^, optical computing^20^, study of non-equilibrium physics^21,22^ and control of chemistry^23^. However, most current designs require microfabrication and cryogenic cooling. They lack the advantages of scalable self-assembly and ambient operation. Plasmonic condensates exist^24,25^ but fail to leverage plasmonics’ key advantage: extreme optical field enhancements from subnanometre gap confinement^26^. Furthermore, these systems often suffer from low emitter gain and material degradation^27–29^ under pulsed excitation^12,30^.

Here we demonstrate a room-temperature continuously driven synchronized dipole state in a self-assembled two-dimensional (2D) plasmonic nanogap array in the bad-cavity limit. Organic dye emitter molecules embedded within coupled subnanometre gaps form a system of emissive dipoles interacting strongly with a collective optical mode under continuous incoherent driving and strong dissipation. This leads to nonlinear synchronization between emitters, and to emergent extended spatial coherence with short temporal coherence. This driven-dissipative system differs from conventional and random lasers, Bose–Einstein condensates (BECs) and superradiant phases, offering a platform to explore how rapidly synchronization develops between emitters. Remarkably, here synchronization arises spontaneously despite high dissipation, incoherent pumping and room-temperature operation. Tuneable dipole densities and interaction strengths can now allow study of driven-dissipative dynamics in regimes where large cavity–dipole coupling rates and loss rates (≫kT) enable collective behaviour at room temperature.

## Dipolar excitons in plasmonic nanogaps

**Fig. 1:**
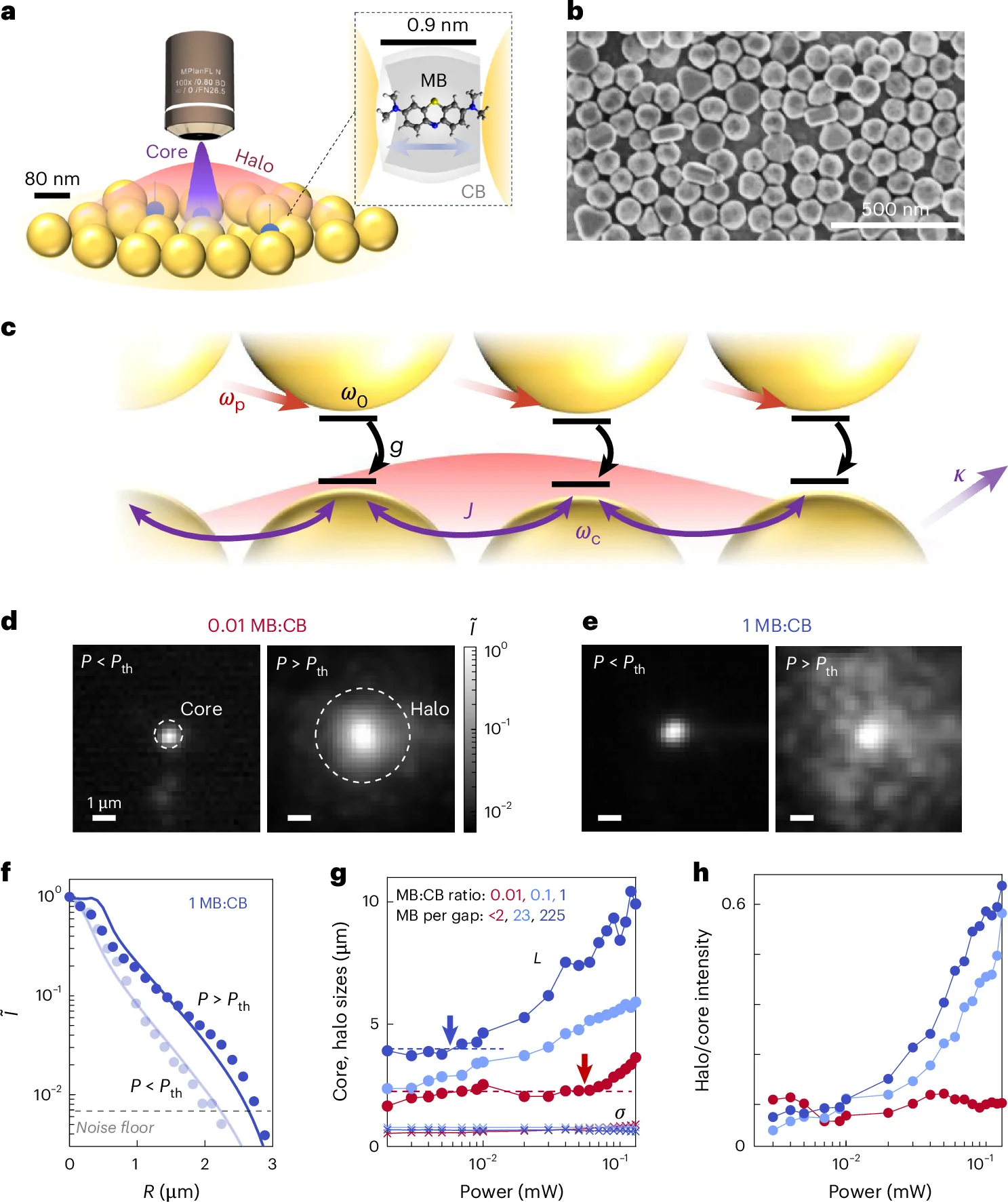
Synchronization of dipoles in plasmonic nanogaps. **a**, Emission from an array of NPs pumped by a tightly focused 633-nm laser exciting the core region that feeds the surrounding halo. The inset shows that an MB emitter within the CB[7] host forms an optical dipole (blue) in the nanogap between gold NPs. **b**, Scanning electron microscopy of the array of NPs. **c**, Each emitter is incoherently pumped by a laser of frequency *ω*_p_ (red arrows) and after non-radiative relaxation emits into the cavity mode with light–matter coupling strength *g*. The cavity is composed of the collective plasmonic nanocavities of individual frequency *ω*_c_ coupled with plasmon–plasmon coupling strength *J*. The cavity leaks light into the environment at loss rate *κ*, which is imaged. **d**,**e**, Imaged normalized emission intensity maps below (*P* = 2 µW) and above (*P* = 130 µW) the threshold pump power *P*_th_ (indicated by arrows in **g**, at which the nonlinear rise begins), for 0.01 (**e**) and 1 (**f**) MB:CB ratios. Core and halo regions are indicated with dotted circles. All images share the same scale bar. **f**, Radially averaged normalized intensity for 1 MB:CB below (*P* = 2 µW) and above (*P* = 130 µW) threshold. Solid lines are fits to the theoretical model (see text). **g**, Sizes of core (*σ*, full width at half maximum) and halo (*L*, exponential decay length). **h**, Ratio of total emitted intensity of the halo region to the core region versus pump power for different MB:CB ratios.

We assemble methylene blue (MB) molecules inside 0.9-nm nanogaps of a close-packed 2D array^31^ of 80-nm gold nanoparticles (NPs) (Fig. 1a–c). Cucurbit[7]uril (CB[7]) molecular scaffolds force MB dipoles to be perpendicular to the metal facets, aligning them with the optical field (Fig. 1a). This allows direct excitation of MB emitters by a focused laser, as well as imaging of their emission (after filtering out all pump laser light).

**Extended Data Fig. 1:**
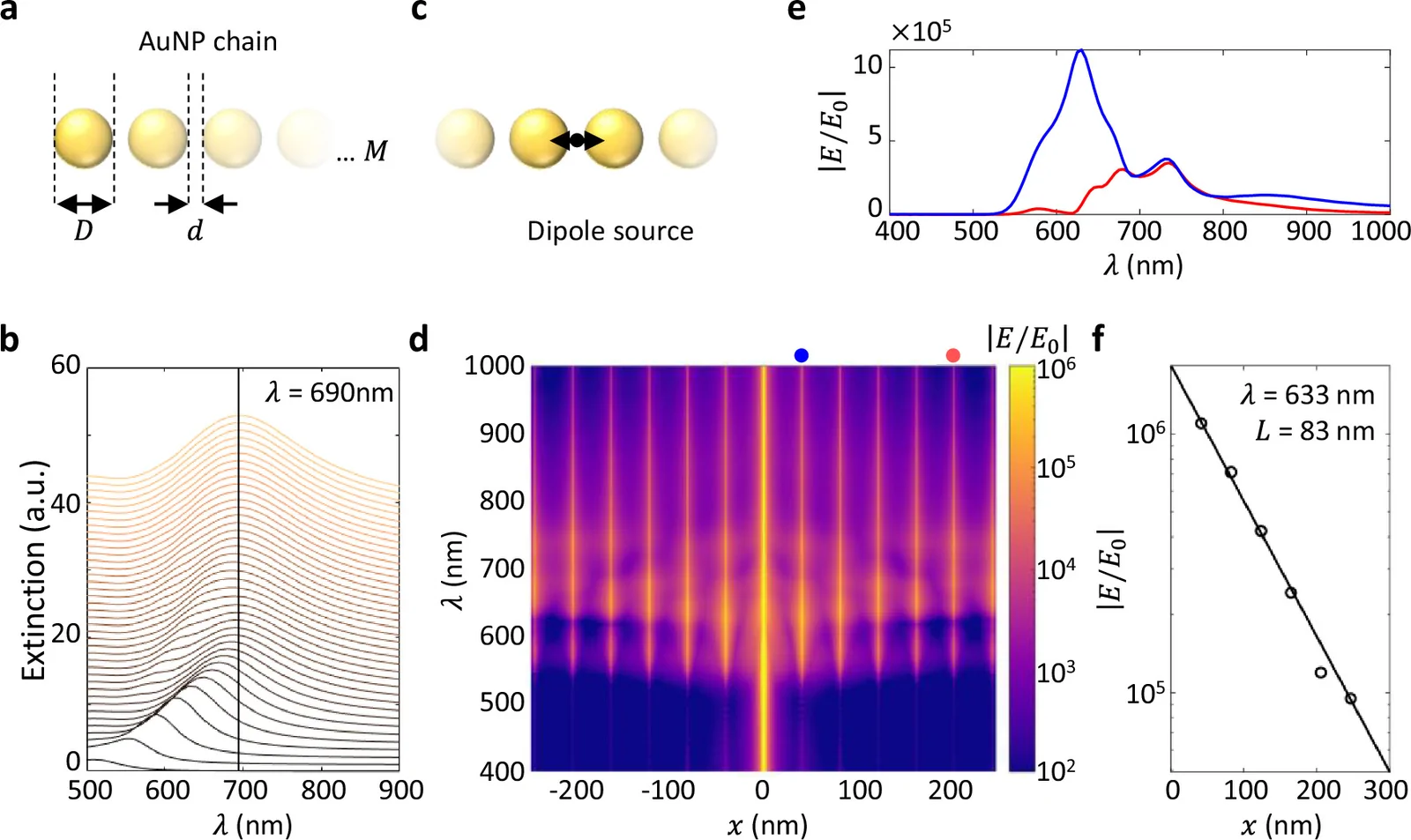
FDTD simulations of plasmonic nanoparticle chains. **a**, Schematic of linear 1D gold nanoparticle (AuNP) chains composed of *M* nanoparticles of diameter *D* and inter-particle gap spacing *d*. **b**, Simulated optical extinction spectrum of chains of (*D* = 80 nm) varying number of AuNPs in the chain from *M* = 1,…, 39 (from the bottom to the top,. offset by 1) under plane-wave excitation polarized parallel to the long axis of the 1D linear chain. Vertical line indicates the centre emission wavelength of methylene blue. **c**, Schematic of linear 1D gold nanoparticle (AuNP) chains excited by a broadband dipole source in the centre gap of the. chain, polarized along the long-axis of the chain. **d**, Optical near field magnitude |*E*| spectrum, normalized to a flat *E*_0_ field of 1 V/m, along the central axis of the 1D chain. Blue and red dots indicate the nanocavities closest to and most distant from the central dipole source, whose spectrum is respectively depicted in **e**, **f**, Decay length of near field magnitude from the nanogap closest to the dipole source to the furthest nanogap. Solid line is an exponential fit *E* = *E*_0_ *exp*(−*x*/*L*) to the decay with decay length *L*.

The nanogaps support highly confined optical modes determined by the NP diameter, gap size and refractive index. Coupling identical nanogaps forms delocalized plasmonic modes across >10 NPs (Supplementary Information section 2), offering unusual properties: high light–matter coupling strength *g* (collectively >100 meV), high photon loss *κ* (>100 meV) and strong radiative leakage. Self-assembly with MB and CB[7] controls the dipole density per gap *Ξ* (via dye loading) and self-aligns all dipoles along the local optical near field, overcoming typical misalignment of randomly oriented dipoles with microcavity optical fields. Furthermore, dipole–dipole coupling between adjacent nanogaps withstands disorder in the chains^32^ (Extended Data Fig. 1 and Supplementary Fig. 2).

This system behaves as collective dipoles that couple to each gap plasmonic mode with strength *g*, and plasmonic modes mutually coupling with hopping strength *J* in a tight-binding model (Fig. 1c). This model accounts for most plasmonic interaction, differing only slightly from the exact plasmonic chain dispersion (Supplementary Fig. 5). The coupled plasmonic modes exhibit low quality factor (*Q*) due to fast radiative (*κ*) and non-radiative (*Γ*) decay, providing a platform for observing dipole synchronization and collective dynamics.

## Spatial halo formation

The samples are pumped at high energy (633 nm), giving Stokes-shifted emission around *λ* = 700 nm after incoherent vibrational relaxation. At low pump powers, the emission spatially matches the Gaussian pump profile. However, as pump powers increase, a dramatic expansion of the emissive region occurs, extending well beyond the 0.5-µm-diameter pump (Fig. 1d,e), forming a ‘halo’ whose intensity grows with *Ξ* (Fig. 1f). Radially averaged images (Fig. 1f) decompose into a fixed Gaussian core (Fig. 1g) and an exponentially decaying larger radial halo. With increasing pump power, the emission from halo dipoles dominates over the core dipoles (Fig. 1h), which is unusual and not expected from ordinary emission where the Gaussian core should remain fixed in size.

To understand the emergence of non-local correlations in the plasmonic system, we first model a one-dimensional tight-binding^33^ chain of *M* sites, each site (position *r*_*n*_) hosting $x=\{1,\ldots ,\Xi \}$ two-level dipoles of frequency *ω*_0_ (Pauli matrices $\Delta ={\sigma_{nx}^\alpha}$) interacting with *M* plasmonic modes *ω*_c_, that is, photons (bosonic operator ${a}_{n}^{\dagger }$ localized in gap *n*) with detuning $\Delta ={{\rm{\omega }}}_{0}-{\omega }_{\rm{c}}$. The emitters are subject to incoherent pumping ${\varGamma }_{n}^{\uparrow }$, decay *Γ*^↓^ and dephasing *Γ* ^*z*^, as well as pair annihilation (within each gap) at rate *Γ* ^ee^. The excitation rate ${\varGamma}_{n}^{\uparrow }$ imprints the Gaussian pump profile at the centre of the chain. This corresponds to a master equation defined by

1 $$H=\sum _{n,x}\frac{\Delta }{2}{\sigma }_{{nx}}^{z}-J\sum _{n}\left({a}_{n+1}^{\dagger }{a}_{n}+{\mathrm{H.c.}}\right)+g\sum _{n,x}\left({a}_{n}{\sigma }_{{nx}}^{+}+{\mathrm{H.c.}}\right)$$

2 $$\begin{array}{l}{\partial }_{t}\rho =-i\left[H,\rho \right]+\sum _{n,x}\left\{\kappa L\left[{a}_{n}\right]+{\varGamma }_{n}^{\uparrow }L\left[{\sigma }_{{nx}}^{+}\right]\right.\\\left.\qquad\;+{\varGamma }^{\downarrow }L\left[{\sigma }_{{nx}}^{-}\right]+{{\varGamma }}^{z}L\left[{\sigma }_{{nx}}^{z}\right]\right\}+{\varGamma }^{\mathrm{ee}}\sum _{n,x,y}L\left[{\sigma }_{{nx}}^{-}{\sigma }_{{ny}}^{-}\right],\end{array}$$

where $L\left[X\right]=X\rho {X}^{\;\dagger }-\{{X}^{\;\dagger }X,\rho \}/2$. To describe this many-body system, we use a second-order cumulant expansion^34,35^ for developing light–matter ${P}_{{nm}}=\left\langle {a}_{n}{\sigma }_{m}^{+}\right\rangle$ and emitter–emitter ${C}_{nm,xy}=\langle {\sigma }_{nx}^{+}{\sigma }_{my}^{-}\rangle$ correlations, in addition to equations for photon–photon correlations ${N}_{{nm}}=\left\langle {a}_{n}^{\dagger }{a}_{m}\right\rangle$, emitter inversion ${S}_{n}=\left\langle {\sigma }_{n}^{z}\right\rangle$ and local emitter spin correlations ${Z}_{n}=\left\langle {\sigma }_{{nx}}^{z}{\sigma }_{{ny}}^{z}\right\rangle$. Further details of the tight-binding model and second-order cumulant expansion are provided in Supplementary Information section 3. This approach allows efficient simulation of the multimode many-molecule system by recognizing that emitters in a single gap have the same on-site average properties (*S*_*n*_) while crucially including the coherences that exist not only across gaps but also between different emitters in the same gap.

Solving for steady-state photon populations *N*_*nn*_ and total emission $I\propto {N}_{\mathrm{tot}}={\sum }_{n}{N}_{nn}$ under increasing pump strength ${\varGamma }_{n}^{\uparrow }$ shows linear, nonlinear and halo-dominated regimes. Increasing the pump amplitude ${\varGamma }_{n=0}^{\uparrow }$ reproduces the observed spatial photon emission profile *N*_*nn*_ (Fig. 1f). This predicted spatial profile (solid lines) shows the expansion of emission when transitioning between regimes. Disorder in our system gives slightly different realizations of the brighter spots (Fig. 1e) localized on the scale of our resolution, highlighting the role of local optical transport between nanogaps, out-scattering from these gaps, and the dependence on local NP gap arrangement (Fig. 1b). Despite this, robust synchronization is still observed even in the presence of disorder and dissipation^7,36,37^.

## Spatiotemporal coherence

**Fig. 2:**
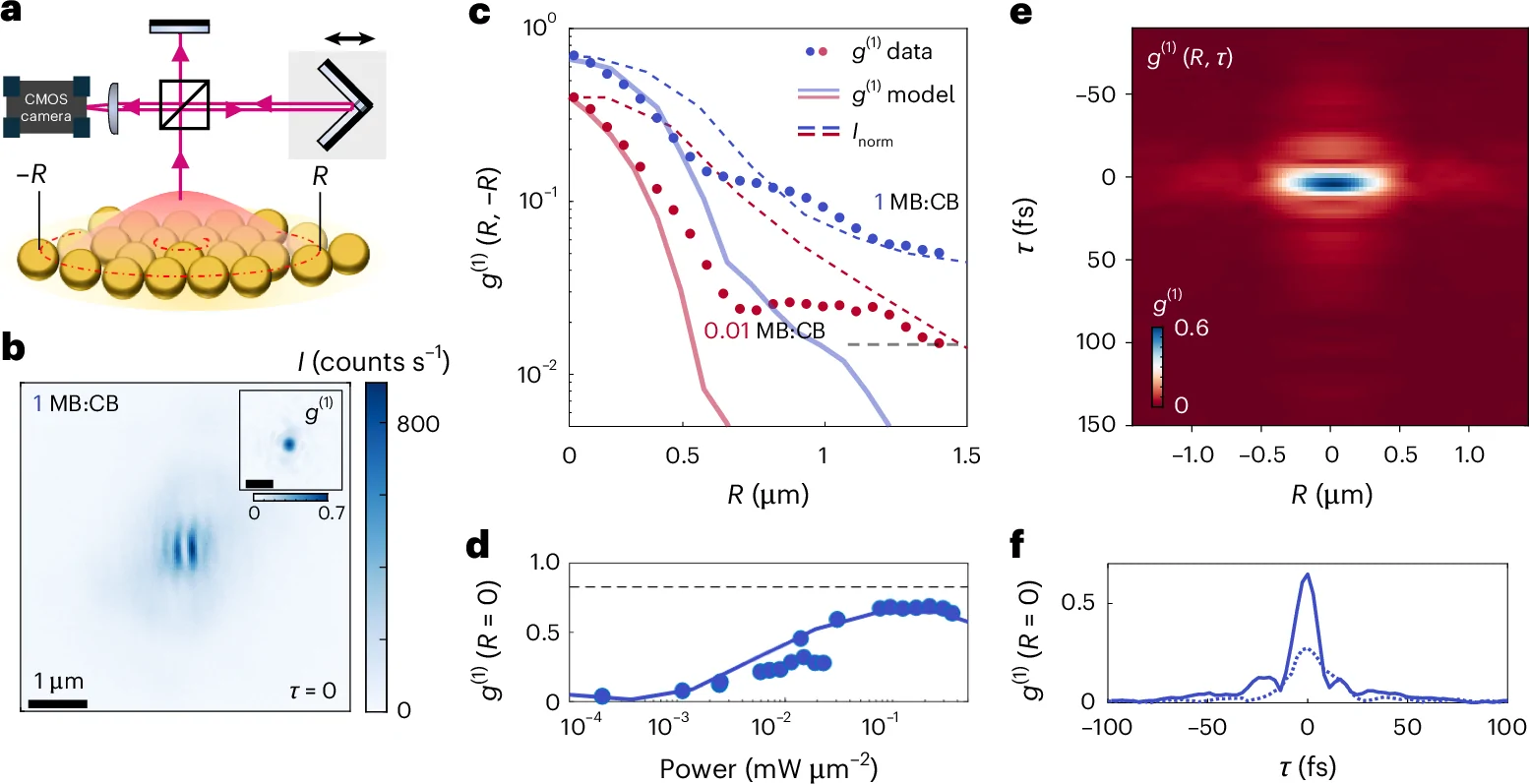
Spatial coherence of light emission. **a**, Flipped Michelson interferometer measuring spatial visibility fringes of emission from the 2D NP layer. **b**, Emission fringes from MB:CB = 1 sample at zero delay (*τ* = 0). The inset shows an extracted image of *g*^(1)^ spatial coherence (details in Supplementary Fig. 13), with the scale bar as in the main image, for pump power *P* = 0.2 mW. **c**, Decay of *g*^(1)^ spatial coherence over radially averaged distance from pump spot (points) for MB:CB = 0.01 and 1, together with intensity profiles (dotted lines) and simple theoretical prediction (solid lines), for pump power *P* = 0.13 mW. **d**, Coherence *g*^(1)^ at core centre versus pump power for MB:CB = 1, showing smooth crossover. The solid line is the theoretical model calculation (Supplementary Information section 3), and the dotted line indicates the measured coherence of the pump laser. **e**, Spatiotemporal *g*^(1)^(*R*, *τ*) decay for MB:CB = 1 at pump power *P* = 0.2 mW. **f**, Extracted time decay at the centre of the pumped region *g*^(1)^(*R* = 0, *τ*) for pump power *P* = 0.2 mW (solid line) and 0.02 mW (dotted line).

Further evidence of unusual, synchronized emission is provided by the coherence recorded from a Michelson interferometer (Fig. 2a). By inverting the emission image in one arm of the interferometer, interference fringes are recorded from increasingly laterally separated (*R*) emissive regions^38,39^ (Fig. 2b). This enables the spatial coherence ${g}^{\left(1\right)}(R)$ to be extracted from the visibility of interference fringes (Supplementary Information section 4) as a function of the relative time delay (*τ*).

At zero delay, *g*^(1)^(*R*, *τ* = 0) initially spans half the pump width but extends substantially into the halo region (Fig. 2c). As pump power increases, *g*^(1)^(*R* = 0, *τ* = 0) rises smoothly from 0.1 to 0.8, saturating near the pump laser coherence (Fig. 2d). This demonstrates that the system smoothly evolves into a coherent state above a critical pump power. The correlation function *N*_*nm*_ directly maps to coherence *g*^(1)^(*R* = 0, *τ* = 0) (Supplementary Information section 3) and is found to agree with our observations of *g*^(1)^(*R* = 0, *τ* = 0) rising across the transition (theory; Fig. 2c,d). Because the Michelson probe integrates over multiple nanogaps within the diffraction limit, the measured *g*^(1)^(*R* = 0, *τ* = 0) reflects spatially averaged mutual coherence.

**Extended Data Fig. 2:**
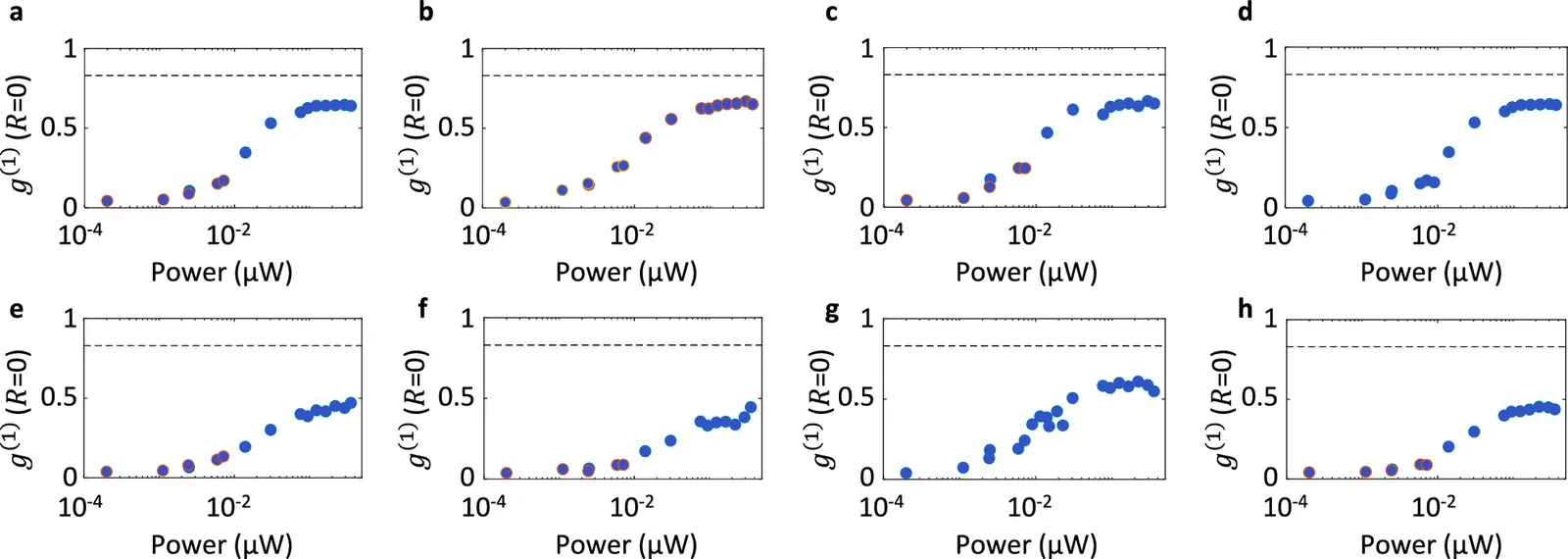
First order spatial coherence variation for MB:CB[7] = 1. (**a**–**h**) Pump power dependence of *g*^(1)^ (R = 0, τ = 0) for MB:CB = 1 across different positions separated laterally by 10 µm step sizes.

The spatial expansion of *g*^(1)^(*R*, *τ* = 0) reflects the spread of correlations as dipoles are driven into inversion farther from the pump by the plasmon-enhanced transport between gaps. Similar behaviour is seen in thermalized photon BECs^39^ and exciton–polariton condensates^40,41^, where long-range order arises from relaxation into a quasi-equilibrium ground state with correlation lengths exceeding the system size. While details of spatial decay profiles vary depending on trap potentials, pump configurations and system sizes, in common is a central highly coherent region, outside which is a near-exponential/power-law decay of quantum-degenerate low-energy states (Extended Data Fig. 2 and Supplementary Fig. 18). Our strongly driven-dissipative system also shows a more complex spatial dependence (Fig. 2c), discussed further below.

The temporal decay of *g*^(1)^(*R*, *τ*) shows an unusual coherence behaviour (Fig. 2e,f). Unlike the stretched exponential decay typical of exciton–polaritons^38^ or exponential decay of photon BECs^42^, *g*^(1)^(*R*, *τ*) exhibits extended spatial coherence at *τ* = 0 (Fig. 2e) that rapidly decays within 10 fs. In addition, the central coherence collapses and revives (Fig. 2f). This ultrafast dephasing arises from the high Purcell enhancement (>1,000), so that dipoles, although spatially correlated, experience extremely rapid coherent decay.

The secondary rise in *g*^(1)^(*R* = 0, *τ*) is found to originate from spectral beating between multiple emission components, as confirmed by Fourier analysis of the measured spectrum (Supplementary Fig. 21). However, phase-resolved interferometry reveals vortex nucleation near the first minimum of *g*^(1)^, indicating that transient spatial phase reconfiguration also contributes to the observed revival. Thus, the collapse-revival-like feature reflects the interplay of spectral beating and spatial phase dynamics rather than coherent Rabi oscillations.

## Nonlinear emission dynamics and universality

**Fig. 3:**
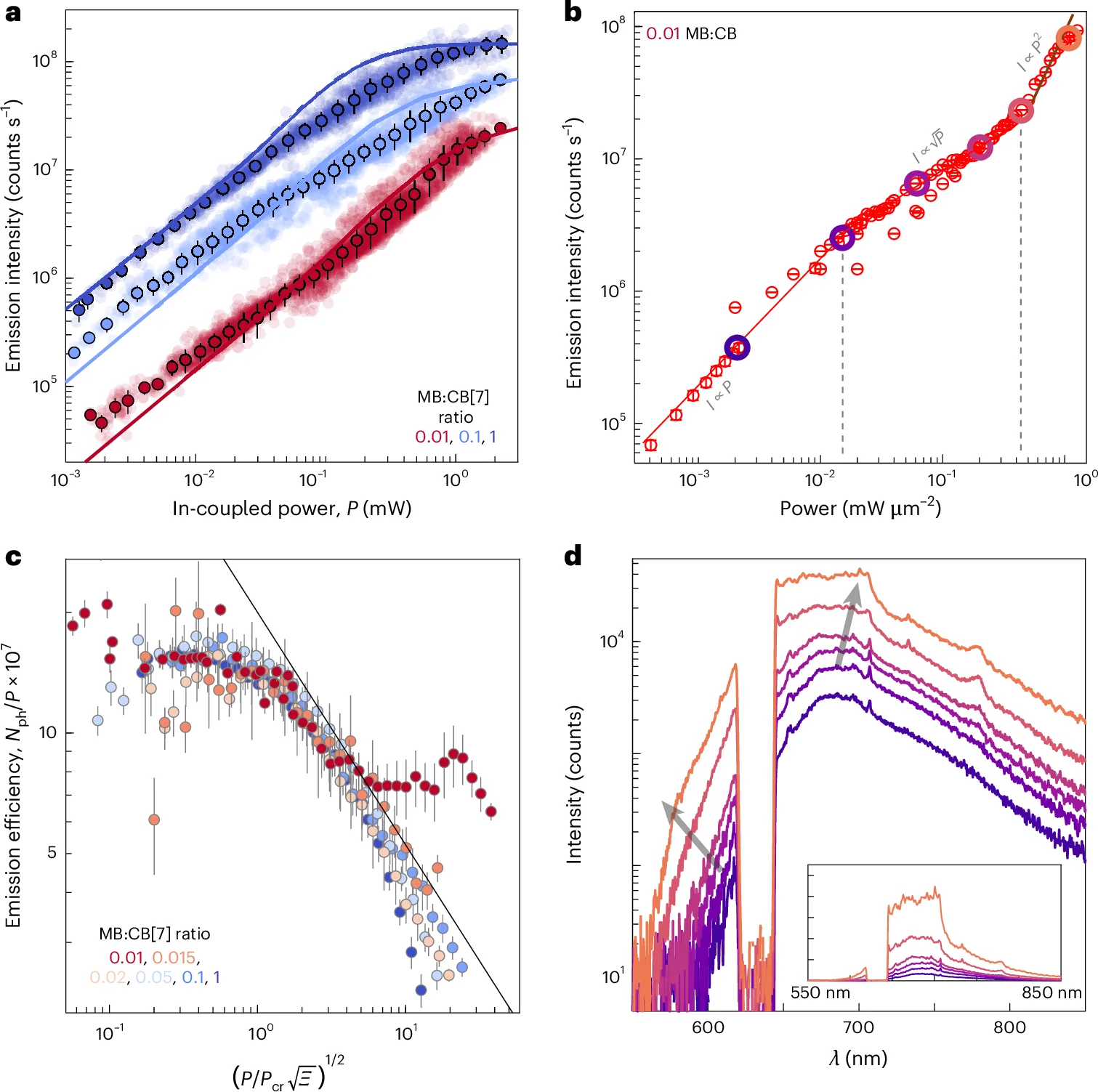
Nonlinear emission for increasing emitter concentration. **a**, Integrated intensity of MB emission versus in-coupled power (see details in Supplementary Information section 5) for increasing dye concentration, showing deviations from linear scaling for different regimes. Solid lines are theoretical predictions for the photon input–output curves. Darker points are the mean values with the standard error of replicates over at least 20 different positions on the sample, with original data as lighter points. **b**, Power dependence of emission intensity *I* at one location showing transitions from linear to sublinear to superlinear regimes (solid lines indicate power laws as labelled). Data are presented as the mean values with the standard error of three spectra acquired at the same location. **c**, Scaling of emitter efficiency (*I*/*P*) with increasing emitter number *Ξ* at power *P*. The black line shows scaling as ${(P/{P}_{{cr}}\sqrt{\Xi })}^{-1/2}$ in the intermediate regime above critical power *P*_cr_. Emitter number *Ξ* is estimated as 100 × MB:CB[7] ratio as there are ~100 molecules per nanogap. Error bars are the standard error of replicates over at least 20 different positions on the sample. Data are presented as the mean values with the standard error of replicates over at least 20 different positions on the sample. **d**, Emission spectra from the core region for MB:CB[7] = 0.01 with increasing pump power (corresponding to coloured circles in **b**). The inset shows spectra on a linear scale. Arrows highlight the nonlinear growth of new Stokes/anti-Stokes emission components. The region around the 633-nm pump is filtered to reject the laser pump scatter.

The total emitted power initially scales linearly with pump power, then becomes sublinear at the onset of coherence (Fig. 3a). To account for variations of in-coupling efficiency across several hundred different pump positions, we rescale the in-coupled power at each position using its low-power linear regime as a reference (Fig. 3a, points, and Supplementary Information section 5). Consistently across the sample, the averaged power dependence (open circles) reveals that the nonlinearity is influenced by *Ξ*, the number of molecules per gap.

The sublinear core emission regime is consistently observed across all MB:CB ratios (Fig. 3a, solid arrows). While increasing from *Ξ* ≈ 2 to 20 scales the emission by a factor of 10, further increasing to *Ξ* ≈ 200 results in only a modest doubling of emission. For smaller *Ξ*, a superlinear emission region also emerges above a second threshold. The results of theoretically predicted input–output curves for photon number versus pumping rate match the experimentally observed data well (Fig. 3a), showing linear, nonlinear and halo-dominated regimes arising from rapid plasmon-driven transport into the halo and saturation in the core (discussed below).

Detailed measurements of core emission versus power for low *Ξ*at a single spot reveals three regimes (Fig. 3b): linear, $I\propto \sqrt{P}$ above a first threshold (dashed line), and $I\,\propto\,{P}^{2}$ beyond a second threshold ~20 times larger. The exact value of power at which these thresholds occur varies from position to position (averaged results in Fig. 3a show smoother features, analysed further in Supplementary Fig. 23). Emission is stable over time, and the power dependence is consistently repeatable, indicating negligible bleaching because Purcell-accelerated radiative rates outpace intersystem crossing and oxidation. The scaling crossover reflects the evolving balance between collective coupling and nonlinear loss. Emission is linear at low pump, becoming sublinear as near-field coupling induces finite-range phase synchronization and halo formation, and at higher densities exciton–exciton annihilation competes with collective emission. Stronger annihilation at high MB:CB ratios suppresses superlinear growth, whereas reduced annihilation at low concentration (*Ξ* = 0.01) enables it.

Tuning the dipole density within the nanogaps shows universal behaviour in the emission efficiency (photon intensity *I* scaled to incoupled pump intensity *P*), with all input–output curves in Fig. 3a collapsing to the same curve in Fig. 3c, using input intensities scaled by the product of a critical intensity (*P*_cr_) and the square root of the emitter density *Ξ*: as $(I/P)\propto {(P/{P}_{{cr}}\sqrt{\Xi })}^{-1/2}$. The exception to universality occurs where pair annihilation cannot happen, for the *Ξ* = 0.01 case where there is on average only approximately one emitter per nanogap.

Emission spectra (Fig. 3d) at increasing pump powers show no line narrowing, matching the temporally short *g*^(1)^(*R* = 0, *τ*), distinguishing this behaviour from conventional laser transitions. Low-power PL arises from the 0–0 and 0–1 vibronic transitions (modelled by Gaussians at 680 nm and 760 nm), along with sharp surface-enhanced Raman peaks (Supplementary Information section 6). In the sublinear regime, where coherent spatial transport emerges, a new peak appears at 770 nm. At higher powers in the superlinear regime, additional Stokes and anti-Stokes emission emerges at 690 nm and 580 nm, resonant with the phonon energy difference between the 0–0 and 0–1 transitions. These vibrational features in the multipeaked emission spectrum modulate *g*^(1)^ via spectral beating (Supplementary Fig. 21), reflecting the electronic–vibrational interplay intrinsic to molecular emitters and absent in atomic or conventional solid-state platforms. Such stimulated phonon driving has implications for the impact of optical pumping on phonon condensation/lasing^43^, as well as optomechanical pumping of molecular vibrations^44^.

## Coherence spreading and dipole correlations

**Fig. 4:**
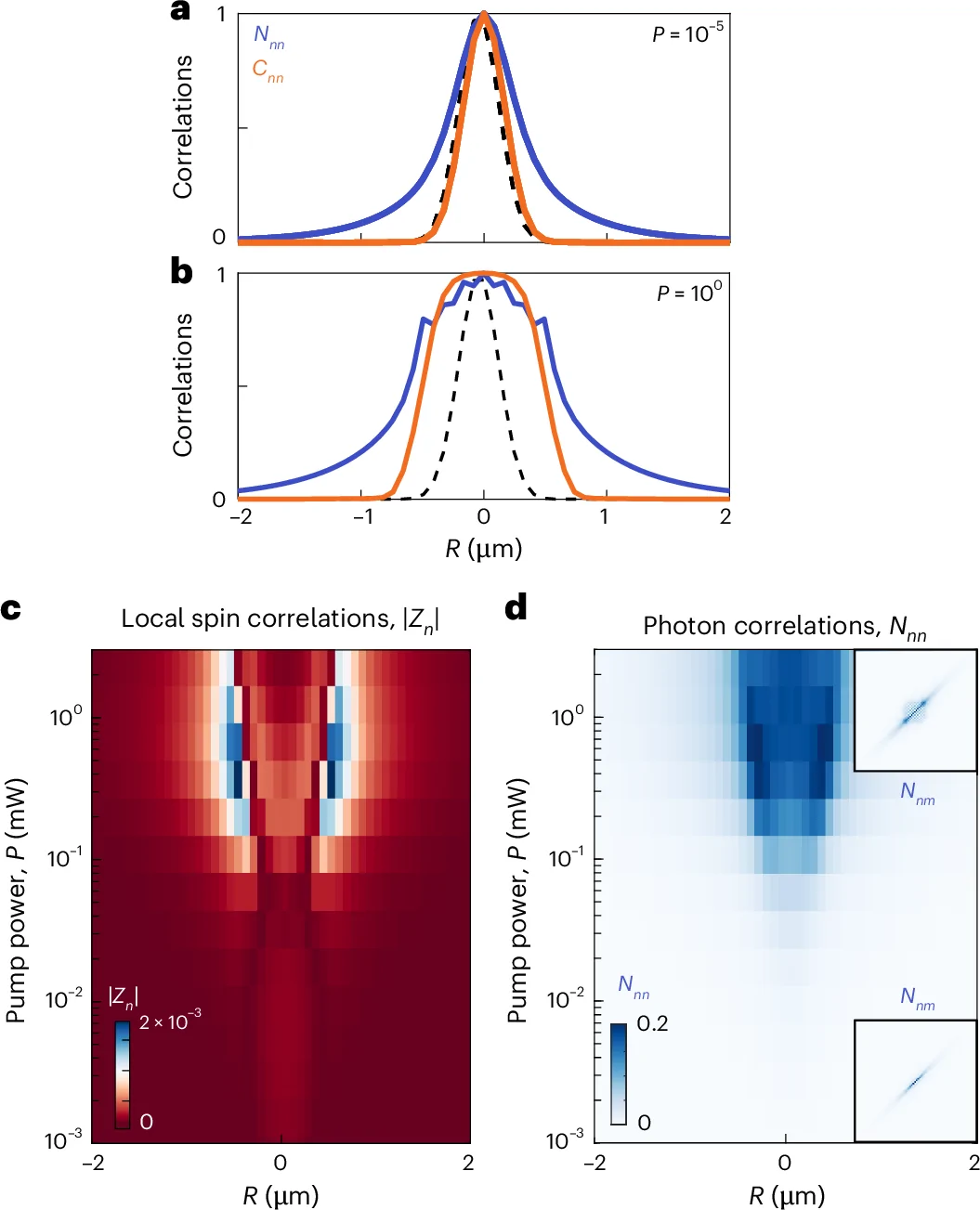
Spreading of correlations and coherence. **a**,**b**, Theoretical model of normalized photon emission on-site correlations (*N*_*nn*_, blue) and on-site dipole correlations (*C*_*nn*_, orange) for pump profile (black) at low pump power *P* = 10^−5^ mW (**a**) and at high pump power *P* = 10^0^ mW (**b**), for *t* = 0.4 eV and *Γ* ^ee^ = 10^−4^ eV. Powers are scaled by a factor *ϕ* = 3 × 10^−4^ to match experimental data in Fig. 3a for MB:CB[7] = 1, and in the model $P=\phi {\varGamma }_{n}^{\uparrow }$, as a function of the excitation rate $P=\phi {\varGamma }_{n}^{\uparrow }$. **c**,**d**, Model emitter–emitter correlations |*Z*_*n*_| (**c**) and photon emission (*N*_*nn*_) (**d**) as a function of pump power and position *R*. Inset shows the normalized off-site photon–photon coherences (*N*_*nm*_) for pump powers in **a** (bottom inset) and **b** (top inset).

Our model shows that the observed halo and coherence spreading arises from competition between light–matter coupling (generating coherence) and dipole–dipole pair annihilation (destroying it). At low pump strengths ${\varGamma }_{n}^{\uparrow }$, stimulated emission into plasmonic modes is insufficient to overcome losses (*κ*, *Γ* ^↓^), so emission stays localized (Fig. 4a) and the dipoles in each gap remain uncorrelated.

As ${\varGamma }_{n}^{\uparrow }$ grows, emitter populations rise and greater pair annihilation saturates the core, while stimulated emission transports coherence outwards via hopping *J*, forming a halo (Fig. 4b). The spatial pump profile creates a core with the highest excitation densities, where annihilation first suppresses emission. Coherence and emission are then redistributed outwards via plasmonic coupling to regions of lower excitation density, forming an emission halo with enhanced width (Fig. 4b, *N*_*nn*_). Without plasmonic coupling (*t* = 0), emission remains localized even at high pump strengths (Fig. 4b, grey). Removing pair annihilation (*Γ* ^ee^ = 0) leads to instability (Supplementary Information section 3) and the lack of any halo.

Although direct modulation of hopping *J* via gap engineering is limited by the molecularly defined nanogap, systematic variation of *Ξ* allows controlled modification of *Γ*_ee_, providing an experimental handle on the nonlinear term in the model. The observed concentration-dependent scaling in Fig. 3c therefore directly supports the theoretical assignment of pair annihilation as a key mechanism governing the collective regime.

Synchronization can be quantified by the rise in spatially remote emitter correlations ($\left|{Z}_{n}\right|$) as ${\varGamma }_{n}^{\uparrow }$ increases (Fig. 4c), indicating phase locking and heralding the transition to the nonlinear regime where a halo appears (Fig. 4d). At the transition, on-site and off-diagonal coherences in the photon–photon correlations *N*_*nm*_ and *g*^(1)^(*R*, *τ* = 0) grow in tandem (Fig. 2d and Fig. 4d, inset), driven by dipole coupling. Here, we thus observe a rapid crossover rather than a true phase transition. Our model shows that local emitter coherence $\left|{Z}_{n}\right|$ rises before photon correlations *N*_*nm*_ spread spatially, establishing phase locking before halo formation. This sequential build-up explains why *g*^(1)^(*R* = 0, *τ* = 0) increases at slightly lower pump intensities (Fig. 2d) than the full spatial expansion (Fig. 1g), reflecting a continuous evolution towards synchronicity. We note that extending this to a 2D model (Supplementary Information section 3.7) gives comparable results.

## Vortices and non-equilibrium behaviour

**Fig. 5:**
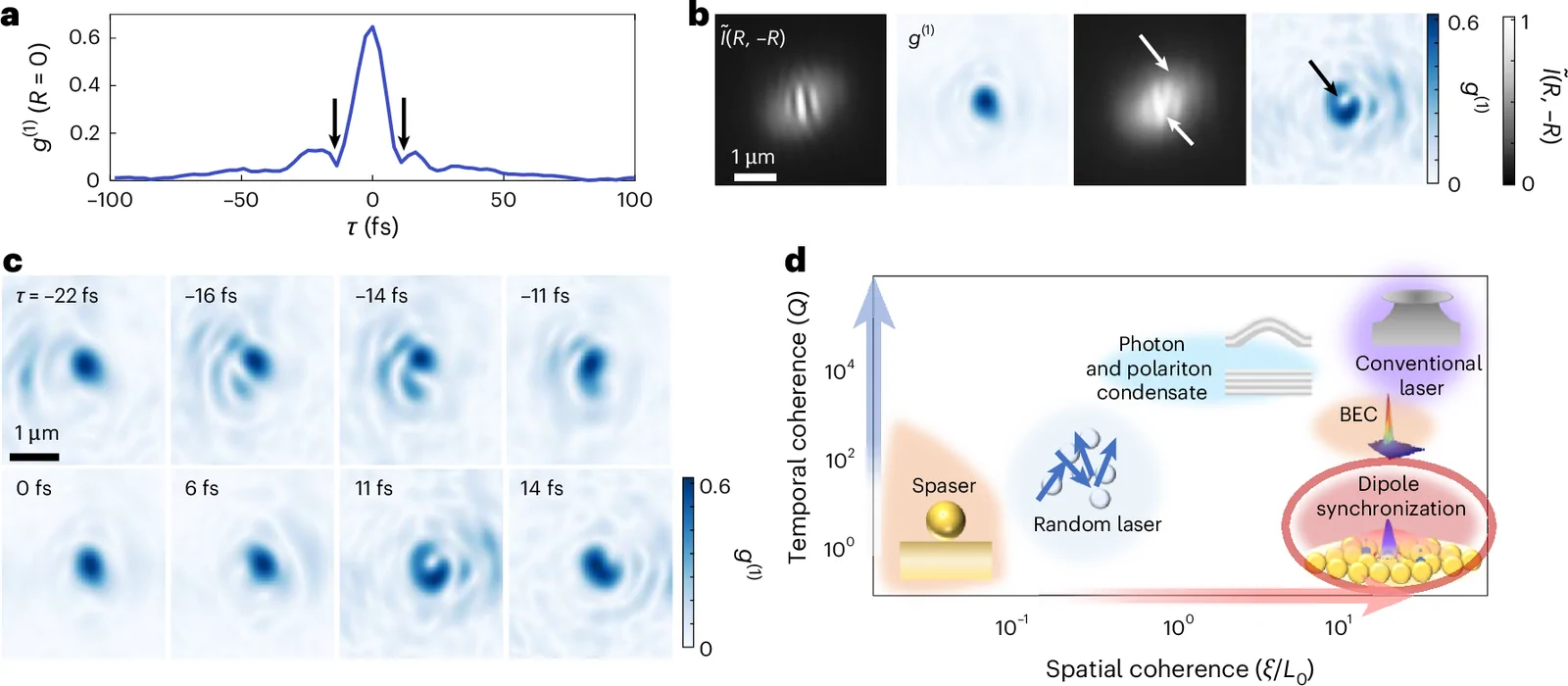
Spatiotemporal vortices forming as a function of delay time at pump power *P* = 0.2 mW for MB:CB = 1 at different time delays *τ*. **a**, *g*^(1)^ (*R* = 0, *τ*) coherence with minima corresponding to images below marked. **b**, Real-space Michelson interferogram intensity profile *I*(*R*, −*R*) and extracted *g*^(1)^ profiles for image with a vortex and without. Vortex indicated by an arrow as a zero in the *g*^(1)^. **c**, Vortex phase dislocation in the *g*^(1)^ (*x*, *y*, *τ*) in space and time, corresponding to zero observed in *g*^(1)^ (*R* = 0, *τ*) coherence trace in **a**. All images share the same scale bar. **d**, Representative coherence properties of diverse synchronized systems (see details in Supplementary Information section 10 and Supplementary Table 2). Temporal coherence is denoted by the quality factor of resonance or trap (*Q*) and spatial coherence by the ratio of coherence length *ξ* to system size *L*.

Spatial coherence arises from dipoles synchronizing their phases through evanescent coupling (set by *J* and *g* in equation (1)) and nonlinear dipole–nanocavity interactions (set by *Γ* ^ee^ in equation (2)). However fast fluctuations from thermal/spontaneous emission noise and Purcell-enhanced decay, cause rapid temporal decoherence. The temporal decoherence manifests in the spatial domain as phase turbulence and slips, appearing as dynamically evolving interference fringes, locally structured emission patterns and vortices (Figs. 1e and 5c). Figure 5b shows vortices appearing as fork-pair defects in the interferograms and as zeros in ${g}^{\left(1\right)}(x,y,\tau )$. Remarkably, Fig. 5c shows that the presence or absence of vortices in the image changes with delay time.

The vortices represent points of zero visibility where the phase of the emission winds by 2π around a central defect (Supplementary Figs. 31 and 32). This is a topological signature that the system has established a degree of global phase order that is susceptible to topological excitations. The vortex here is a phase dislocation between successive frames of the optical field taken 11 fs apart (Fig. 5c). Note that, because ${g}^{\left(1\right)}(x,y,\tau )$ involves time integration (at fixed delay *τ*), this changing vorticity does not simply reflect a vorticity that changes with time; after time integration, such fluctuating vorticity would simply give reduced coherence. Instead, this result indicates time-correlated fluctuations associated with changing vorticity.

These vortices appear only within the coherence time and resemble the time-averaged interferograms previously observed in polariton/photon condensates^45–48^. A vortex with *g*^(1)^ = 0 traverses the central *R* = 0, thus inducing the oscillations in *g*^(1)^ observed in Figs. 2f and 5a. We suggest that this puzzling behaviour of vortex symmetry breaking could be induced by disorder, in both spatial variation of plasmon transport and outcoupling rates, causing transient bursts of correlated emission to circulate as it propagates. Confirmation of the vortex phase winding around 2π, variation across sample positions, and agreement between experimental and simulated interferograms is discussed in Supplementary Information section 7.

## Conclusions

We have realized a continuous-wave-pumped room-temperature synchronized dipole state in a quasi-2D array of plasmonic nanogaps. The coupled nanogap geometry combines ultralow optical mode volumes, high Purcell factors and strong dipole–field interactions, while retaining scalable self-assembly and ambient operation. Emission nonlinearity, halo growth and the onset of spatial coherence indicate finite-range phase locking among optical dipoles. This synchronization manifests as spatial expansion and enhanced first-order coherence rather than beamed emission or spectral linewidth narrowing, distinguishing it from conventional lasers, polariton condensates and random lasers. The absence of linewidth narrowing, together with rapid radiative decay, shows that temporal photon coherence remains short; instead, coherence resides in molecular dipoles coupled through plasmonic near fields. Fast Purcell-enhanced emission limits temporal coherence, while lateral coherence reflects outward propagation of dipole phase correlations, observed experimentally as an expanding emission halo, which scales with a larger pump area (Supplementary Figs. 40 and 41).

Synchronization in driven-dissipative dipole arrays has been theoretically established^7^. Our nanogap system contains the same ingredients—interacting two-level dipoles, collective decay, incoherent pumping and strong dissipation, now implemented in a room-temperature plasmonic architecture. Our model shows that local dipole spin correlations (*Z*_*n*_) establish before the spatial expansion of photon emission, indicating that phase locking precedes ‘halo’ formation. This sequential build-up explains why the onset of coherence appears before the full spatial expansion and shows that the system undergoes a rapid crossover rather than a true phase transition. The concentration-dependent scaling further supports pair annihilation as a key nonlinear process controlling the collective regime. In this extreme bad-cavity limit, phase order arises from near-field dipolar interactions on timescales shorter than dissipation, rather than from long-lived cavity photons, mode selection or equilibrium condensation.

This sequential build-up distinguishes the synchronized dipole state from standard threshold-driven transitions in polaritons. Compared with other emissive systems (Fig. 5d and Supplementary Table 2), synchronization is central: unlike random lasing, spatial coherence appears; unlike conventional lasing or polariton condensation^49^, temporal coherence remains short; and unlike superradiance^50^, operation is continuous-wave. The observation of spatiotemporal vortices further indicates that the synchronized state is intrinsically non-equilibrium. These vortices appear as phase dislocations in the measured first-order *g*^(1)^ coherence and evolve within the ultrashort coherence time, showing that the system supports transient phase turbulence and phase slips alongside locally synchronized regions. This behaviour is reminiscent of driven-dissipative systems such as Josephson junction arrays^51^, laser arrays^52^, ultracold atoms^53^ and non-equilibrium 2D BECs^54^, where chimera-like synchronized and asynchronous domains can coexist. In the present plasmonic nanogap arrays, disorder in local coupling and outcoupling probably promotes this coexistence, producing finite-range phase order rather than global temporal coherence.

This platform opens a route to mesoscopic quantum photonics in strongly dissipative, room-temperature materials. Collective phase order among strongly coupled emitters enables engineered quantum resources^8,55^, phase-coherent readout of embedded emitters such as molecular qubits^56^ or colour centres^57^, and routes to polariton-enhanced exciton transport and chemically active light–matter states^58,59^ beyond conventional lasing or condensation. Long photon lifetimes are not essential: in bad-cavity systems, coherence can reside in emitters rather than the cavity field, improving metrology^60^, while Purcell-enhanced ultrafast emission increases rates and can outpace slow environmental noise^61,62^. Synchronized dipolar dynamics offer a route to collective optical functions in molecular and nanoscale materials where disorder and dissipation are not removed, but become defining features of the operating regime.

## Methods

### Monolayer aggregate self-assembly

Interfacial self-assembly of gold NP aggregates are performed with a previously developed method^31^. MB (1 mM aqueous, Sigma-Aldrich) and cucurbit-7-uril (1 mM aqueous, Sigma-Aldrich) solutions are mixed in varying volume fractions to achieve different concentrations of MB:CB[7] supramolecular complexes. Then, 150 µl of this solution is added to 500 µl of 80-nm citrate-capped Au NP solutions (BBI Solutions) on an aqueous interface with chloroform. The NP phase is then mixed well and forms NP aggregates sandwiching the MB:CB[7] supramolecular complexes that settle to the interface with chloroform. This phase is then concentrated and deposited onto the surface of a gold-coated silicon substrate (50 nm Au thickness with 5 nm Cr adhesion layer). After deposition, the samples are rinsed with distilled water and dried with nitrogen.

### Photoluminescence measurements

The surface-enhanced Raman and scattering spectra measurements are performed in a home-built Python-controlled confocal Raman microscope with a 100× 0.9 numerical aperture (NA) objective and a Shamrock i303 spectrograph and a Newton EMCCD. The set-up allows automatic mapping that provides statistical spectroscopy data from many locations. The integration time for spectra is 1 s. All the measurements are performed with a continuous-wave 633-nm laser (Matchbox, Integrated Optics). The laser power is controlled over several orders of magnitude by a series of fixed neutral density filters and a continuously tuneable reflective neutral-density filter. The 100×, 0.9 NA objective lens results in a focal spot size of approximately 564 nm and a focal spot area of approximately 1 µm^2^.

### First-order coherence measurements

The coherence measurements are performed on a home-built interferometer by sending the emitted light collected by the 100× 0.9 NA objective to a 50:50 non-polarizing beamsplitter. One arm of the interferometer directly reflects the light with a plane mirror, while the other flips the beam in both transverse directions with a retro-reflector on a motorized delay stage. The two arms are then sent to a lens (Thorlabs, *f* = 60 cm) which images the emission on a complementary metal–oxide–semiconductor camera (Prime BSI sCMOS, Photometrics). The separation between the two beams on the lens sets the width of the interference fringes observed. The motorized delay stage (Thorlabs PT1/M-Z9) is then scanned to equalize the path length of the interferometer and determine the first-order temporal coherence of the emission. This is referenced with white light reflecting off a mirror to find time zero.

### Theoretical dipole synchronization model

An open quantum systems approach was used to model the dipole synchronization, with all details provided in Supplementary Information section 3. The model can be reproduced in Python with files available via GitHub at https://github.com/piperfw/srdop/tree/plasmon.

### Electromagnetic simulations

Finite-difference time-domain simulations were performed with Lumerical FDTD, with details provided in Supplementary Information section 2.

## Online content

Any methods, additional references, Nature Portfolio reporting summaries, source data, extended data, supplementary information, acknowledgements, peer review information; details of author contributions and competing interests; and statements of data and code availability are available at https://doi.org/10.1038/s41565-026-02242-w.

## Supplementary information

Supplementary Information Supplementary Figures 1–41, Supplementary Discussion and Supplementary Tables 1 and 2.

Peer Review File

## Data Availability

All the data in this Article are available via the Cambridge Apollo repository at https://doi.org/10.17863/CAM.131678.
