# Supplementary material for: Coherent room-temperature dipole synchronization in plasmonic nanocavities
Source: Nat Nanotechnol. 2026 Jul 30;21(9):1230–7. doi: 10.1038/s41565-026-02242-w (PMC13569432; doi:10.1038/s41565-026-02242-w)
Supplement: Supplementary file 1 [file 41565_2026_2242_MOESM1_ESM.pdf]

nature nanotechnology

Supplementary information https://doi.org/10.1038/s41565-026-02242-w

Coherent room-temperature dipole synchronization in plasmonic nanocavities

In the format provided by the authors and unedited

Supplementary Information

Contents

S1 Experimental Methods & Materials **2**

S2 Electromagnetic simulations of plasmonic chains **3**

S3 Theoretical model and calculations **4**
S3.1 Model 4
S3.2 Method 6
S3.3 Observables 8
S3.4 Tight-binding and full plasmonic chain dispersion 11
S3.5 Instability with zero pair annihilation rate 12
S3.6 Pair/exciton-exciton annihilation 12
S3.7 2D Model 13
S3.8 Limitations of theoretical model 17

S4 Interferometer calibration and coherence measurement protocol **18**

S5 Power dependence and input power scaling **26**

S6 Surface-enhanced vibronic and Raman spectra **28**

S7 Polariton vortex simulations and experiments **32**
S7.1 Vortex and phase gradient (compressible and incompressible) components 33

S8 Polarization and k-space measurements **39**

S9 Pump spot size dependence and imaging **42**

S10 Comparison of dipole synchronization systems **44**

SI Section S1. Experimental Methods & Materials

Monolayer aggregate self-assembly. Interfacial self-assembly of gold nanoparticle aggregates are performed with a previously developed method [1]. Methylene-blue (1mM aqueous, Sigma-Aldrich) and cucurbit-7-uril (1mM aqueous, Sigma-Aldrich) solutions are mixed in varying volume fractions to achieve different concentrations of MB:CB[7] supramolecular complexes. 150 µL of this solution is then added to 500 µL of 80 nm citrate-capped Au nanoparticle solutions (BBI Solutions) on an aqueous interface with chloroform. The nanoparticle phase is then mixed well and forms nanoparticle aggregates sandwiching the MB:CB[7] supramolecular complexes which settle to the interface with chloroform. This phase is then concentrated and deposited onto the surface of a gold-coated silicon substrate (50 nm Au thickness with 5 nm Cr adhesion layer). After deposition, the samples are rinsed with distilled water and dried with nitrogen.

Photoluminescence measurements. The SERS and scattering spectra measurements are performed in a home-built Python-controlled confocal Raman microscope with a 100X 0.9 N.A. objective and a Shamrock i303 spectrograph and a Newton EMCCD, as discussed in detail in previous studies [2–4]. The setup allows automatic mapping that provides statistical spectroscopy data from many locations. The integration time for spectra are 1s. All the measurements are performed with a continuous-wave 633nm laser (Matchbox, Integrated Optics). The laser power is controlled over several orders-of-magnitude by a series of fixed neutral density filters and a continuously tuneable reflective neutral-density filter. The 100X, 0.9 NA objective lens results in a focal spot size of approximately 500 nm.

First-order coherence measurements. The coherence measurements are performed on a home-built interferometer by sending the emitted light collected by the 100X 0.9 N.A. objective to a 50:50 non-polarizing beamsplitter. One arm of the interferometer directly reflects the light with a plane mirror, while the other flips the beam in both transverse directions with a retro-reflector on a motorized delay stage. The two arms are then sent to a lens (Thorlabs, $f = 60$ cm) which images the emission on a CMOS camera (Prime BSI sCMOS, Photometrics). The separation between the two beams on the lens sets the width of the interference fringes observed. The motorized delay stage (Thorlabs PT1/M-Z9) is then scanned to equalize the path length of the interferometer and determine the first-order temporal coherence of the emission. This is referenced with white light reflecting off a mirror to find time-zero.

Electromagnetic simulations. Finite-difference time-domain simulations are performed with Lumerical FDTD. Chains of spherical Au nanoparticles are simulated with a 1 nm gap, modeled with a small mesh refinement around the gaps, and excited by a broadband dipole source oriented along the long-axis of the nanoparticle chains. Gold's permittivity was taken from Johnson & Christy [5]. The optical scattering spectrum under plane wave illumination for increasing the number of nanoparticles in the chain was performed with the multiple sphere T-matrix code MSTM49, with the Au dielectric function of Johnson & Christy [5].

SI Section S2. Electromagnetic simulations of plasmonic chains

The plasmonic modes of locally ordered but globally disordered nanoparticle arrays on a substrate are complex [6], but can be approximated in the first instance, when excited by a polarized laser source, as fractal aggregates composed of linear chain segments. Supplementary Figure **S1**a,b show how the modes of a nanoparticle chain (diameter, D = 40 nm, gap separation, d = 1 nm) redshift to the near-infrared regime, when tuned from one to 40 particles in a linear chain. The fundamental collective plasmonic mode saturates in energy past approximately 10 particles in a chain, and broadens in spectral linewidth as the bright coupled mode becomes superradiant and undergoes strong radiative losses [1]. This spectrally broad mode extends throughout much of the visible and near-infrared, enabling efficient coupling with most broadband organic emitters.

Placing a dipole excitation source within the centre of this chain (Supplementary Figure **S1**c) and calculating the field propagating along the chain from the centre outwards shows how light couples from one nanogap to the next. The field shows an enhancement within each nanogap (Supplementary Figure **S1**d) and a spectral response which varies from the innermost to outermost gaps (from blue dot labelled nanogap to red dot, as in Supplementary Figure **S1**d,e). This is primarily due to the attenuation of lower wavelengths due to absorption loss as the dipole excites propagating plasmonic modes.

The coupling between plasmonic nanocavities results in a spatial decay of the optical field which is slower than in free-space, showing exponential decay (Supplementary Figure **S1**f) as opposed to the $1/r^3$ decay expected of a dipole in vacuum. The fitted exponential decay length is 83 nm, which strongly motivates the nearest-neighbour coupling utilized in SI Section S3 to approximate the plasmonic nanocavity coupling.

The coupling between plasmonic nanocavities is robust to geometric distortions of the chain, as shown in Supplementary Figure **S1**a where we bend the chain up to 30°and the majority of optical energy still propagates along the chain. The optical field only decays with a slightly smaller exponential decay length of 51 nm (Supplementary Figure **S1**b), still enabling adequate nearest-neighbour plasmonic coupling.

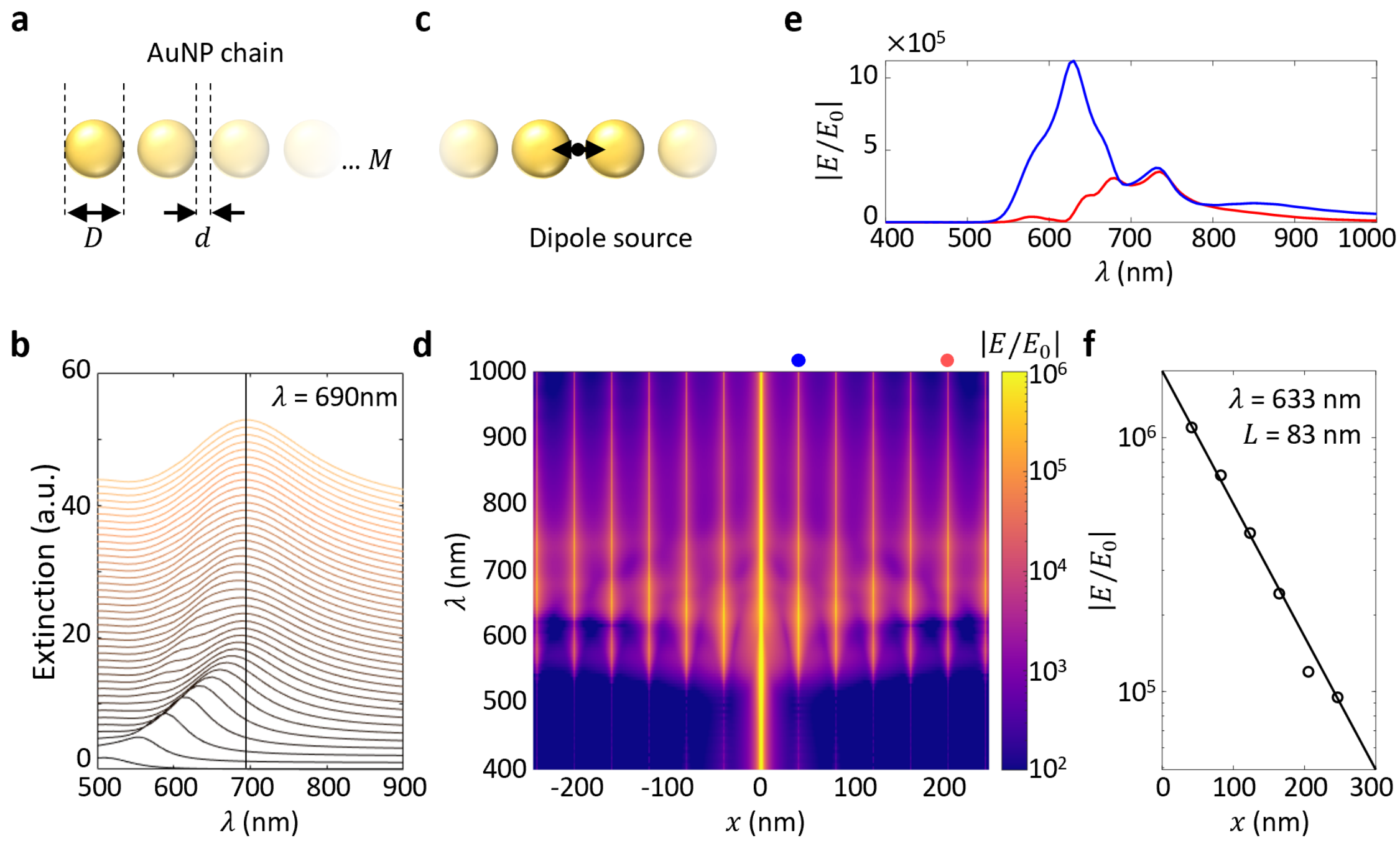

Supplementary Figure S1: **FDTD simulations of plasmonic nanoparticle chains. a,** Schematic of linear 1D gold nanoparticle (AuNP) chains composed of M nanoparticles of diameter D and inter-particle gap spacing d. **b,** Simulated optical extinction spectrum of chains of (D = 80 nm) varying number of AuNPs in the chain from $M = 1,\ldots, 39$ (from the bottom to the top, offset by 1) under plane-wave excitation polarized parallel to the long axis of the 1D linear chain. Vertical line indicates the centre emission wavelength of methylene blue. **c,** Schematic of linear 1D gold nanoparticle (AuNP) chains excited by a broadband dipole source in the centre gap of the chain, polarized along the long-axis of the chain. **d,** Optical near field magnitude $|E|$ spectrum, normalized to a flat E_0 field of 1 V/m, along the central axis of the 1D chain. Blue and red dots indicate the nanocavities closest to and most distant from the central dipole source, whose spectrum is respectively depicted in **e,**. **f,** Decay length of near field magnitude from the nanogap closest to the dipole source to the furthest nanogap. Solid line is an exponential fit $E = E_0 \exp(-x/L)$ to the decay with decay length L.

SI Section S3. Theoretical model and calculations

In this section, we detail the tight-binding model, the second-order cumulant expansion, and the computation of the theoretical curves shown in Figs. 1–4.

S3.1 Model

As described in the article, we develop a tight-binding model for the plasmonic system, where M plasmonic modes interact with Ξ identical quantum dipoles localized in the M gaps between

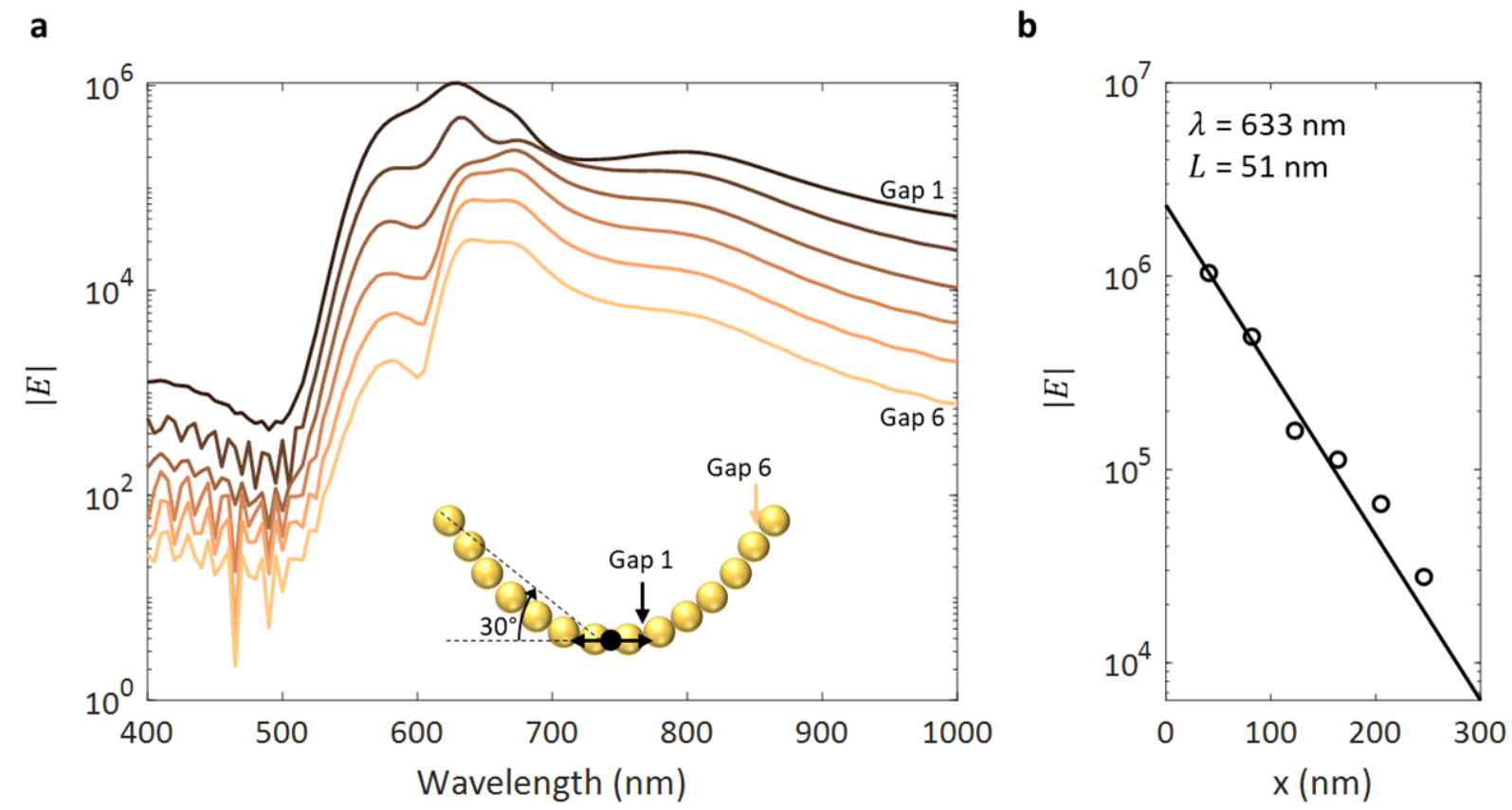

Supplementary Figure S2: **Optical near field with bent chain. a,** Optical near field magnitude E spectrum under dipole excitation aligned parallel to the central nanogap as depicted, for nanogap 1 closest to the dipole source to nanogap 6 furthest away. The nanoparticle chain is bent at an angle of 30°. **b,** Decay of E with nanogap position along the bent chain at $\lambda = 633$ nm pump excitation, with an exponential fit $E = E_0 \exp(-x/L)$ to the decay with decay length L.

nanoparticles (Supplementary Figure S3). Focusing on the radial dependence of observables, we restrict our analysis to a one-dimensional chain with periodic boundary conditions.

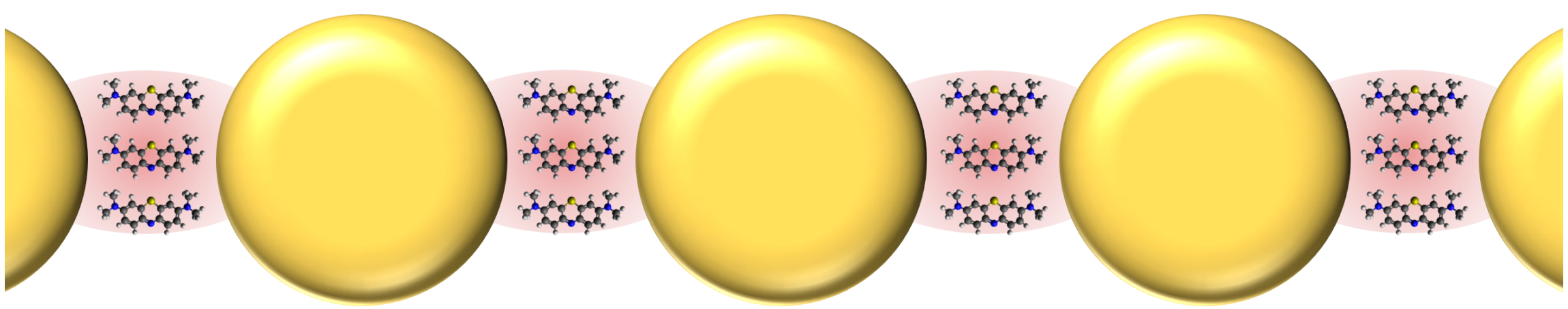

Supplementary Figure S3: **Schematic for the plasmonic chain**, showing collections of MB particles confined to gaps between gold nanoparticles. Interactions with local plasmonic fields facilitate the redistribution of localised input excitation within a pump spot along the chain in a tight-binding model.

We define bosonic operators a_n for the plasmonic field at site $n = 0, \dots, M-1$ (position R_n) and Pauli operators $\sigma^{\alpha}_{n\mathrm{x}}$ for the dipoles, where x labels the distinct dipoles at a given site, with $\mathrm{x} = 0, \dots, \Xi - 1$ for Ξ dipoles per site ($\Xi = 2$, 23, 225). The system Hamiltonian, working in a

frame rotating at the cavity frequency, can be written as

$$H = \sum_{n,\mathrm{x}} \frac{\Delta}{2}\sigma^z_{n\mathrm{x}} - J\sum_n \left(a^\dagger_{n+1}a_n + \mathrm{H.c.}\right) + \sum_{n,\mathrm{x}} g\left(a_n\sigma^+_{n\mathrm{x}} + \mathrm{H.c.}\right). \tag{1}$$

Here, g is the individual dipole-plasmon coupling, J the hopping strength, and $\Delta = \omega_0 - \omega_c$ the detuning between the dipole frequency and the centre of the plasmonic band. We set $\Delta = 0$ and note that $g_{\mathrm{site}} = g\sqrt{\Xi}$ characterizes the collective light-matter coupling within a single nanoparticle gap. Note that in contrast to bulk systems eg. organic microcavities, where g scales inversely with the square root of the total mode volume and, therefore, the total number of emitters [7], we consider a fixed gap volume and so individual coupling g.

In addition to the unitary dynamics governed by Eq. (1), we consider incoherent processes describing plasmonic loss at rate κ as well as dipole pump $\Gamma^\uparrow_n$, decay $\Gamma^\downarrow$, dephasing Γ^z, and pair annihilation Γ^{ee} within each gap. The excitation rate $\Gamma^\uparrow_n$ encodes the spatial profile of the pump, while the other rates are taken to be independent of the site index n. Each process is incorporated as a Markovian term in the master equation for the total density operator $\rho = \rho(t)$,

$$\partial_t\rho = -i[H,\rho] + \kappa\sum_n L[a_n] + \sum_{n,\mathrm{x}} \Gamma^\uparrow_n L[\sigma^+_{n\mathrm{x}}] + \Gamma^\downarrow\sum_{n,\mathrm{x}} L[\sigma^-_{n\mathrm{x}}] + \Gamma^z\sum_{n,\mathrm{x}} L[\sigma^z_{n\mathrm{x}}] + \Gamma^{\mathrm{ee}}\sum_{n,\mathrm{x},\mathrm{y}} L[\sigma^-_{n\mathrm{x}}\sigma^-_{n\mathrm{y}}], \tag{2}$$

where $L[X] = X\rho X^\dagger - \{X^\dagger X, \rho\}/2$.

S3.2 Method

We now outline the method [8] for solving the dynamics governed by Eq. (2), enabling the calculation of relevant observables in the steady state. Our approach proceeds in two steps. First, we derive Heisenberg equations of motion from Eq. (2) for the expectation values of the operator pairs $\langle a^\dagger_n a_m\rangle$, $\langle a_m\sigma^+_{n\mathrm{x}}\rangle$, $\langle\sigma^+_{n\mathrm{x}}\sigma^-_{m\mathrm{y}}\rangle$, and $\langle\sigma^z_{n\mathrm{x}}\sigma^z_{n\mathrm{y}}\rangle$, along with an equation for the single-site expectation $\langle\sigma^z_{n\mathrm{x}}\rangle$. The diagonal elements of $\langle a^\dagger_n a_n\rangle$ and $\langle\sigma^z_{n\mathrm{x}}\rangle$ describe the average plasmon population and dipole inversion at site n, respectively, and the remaining terms describe correlations.

We define the total emitter linewidth as $\Gamma_n = \Gamma^\uparrow_n + \Gamma^\downarrow - 4\Gamma^z$, the effective pair annihilation rate as $\Gamma^\Xi = (\Xi - 1)\Gamma^{\mathrm{ee}}$ for Ξ dipoles per gap, and $S_0 = (\Gamma^\uparrow_n - \Gamma^\downarrow - \Gamma^\Xi)/(\Gamma_n - 4\Gamma^z + 2\Gamma^\Xi)$. With these

definitions, the equations of motion are

$$\begin{aligned}
\partial_t\langle a_n^\dagger a_m\rangle &= -\kappa\langle a_n^\dagger a_m\rangle + ig\Xi\left(\langle a_m\sigma_n^+\rangle - \overline{\langle a_n\sigma_m^+\rangle}\right)\\
&\quad - it\left(\langle a_{n+1}^\dagger a_m\rangle + \langle a_{n-1}^\dagger a_m\rangle - \langle a_n^\dagger a_{m-1}\rangle - \langle a_n^\dagger a_{m+1}\rangle\right)
\end{aligned}\tag{3}$$

$$\partial_t\langle\sigma_n^z\rangle = -\left(\Gamma_n - 4\Gamma^z + 2\Gamma^\Xi\right)\left(\langle\sigma_n^z\rangle - S_0\right) + 4g\ \mathrm{Im}\langle a_n\sigma_n^+\rangle - \Gamma^\Xi\langle\sigma_n^z\sigma_n^z\rangle \tag{4}$$

$$\begin{aligned}
\partial_t\langle a_m\sigma_n^+\rangle &= \left(i\Delta - \frac{\Gamma_n + \kappa + \Gamma^\Xi}{2}\right)\langle a_m\sigma_n^+\rangle - \frac{\Gamma^\Xi}{2}\langle a_m\sigma_n^+\sigma_n^z\rangle + it\left(\langle a_{m-1}\sigma_n^+\rangle + \langle a_{m+1}\sigma_n^+\rangle\right)\\
&\quad - ig\left[\left(\langle a_n^\dagger a_m\sigma_n^z\rangle + \frac{\delta_{nm}}{2}\langle\sigma_n^z\rangle\right) + \frac{\delta_{nm}}{2} + \left(\Xi - \delta_{nm}\right)\langle\sigma_n^+\sigma_m^-\rangle\right]
\end{aligned}\tag{5}$$

$$\begin{aligned}
\partial_t\langle\sigma_n^+\sigma_m^-\rangle &= -\left(\frac{\Gamma_n + \Gamma_m + 2\Gamma^\Xi}{2}\right)\langle\sigma_n^+\sigma_m^-\rangle - \frac{\Gamma^\Xi}{2}\left(\langle\sigma_n^+\sigma_m^-\sigma_m^z\rangle + \langle\sigma_n^+\sigma_m^-\sigma_n^z\rangle\right)\\
&\quad + \Gamma^{\mathrm{ee}}\delta_{nm}\left(\langle\sigma_n^+\sigma_n^-\rangle + \langle\sigma_n^+\sigma_n^-\sigma_n^z\rangle\right) + ig\left(\langle a_m\sigma_n^+\sigma_m^z\rangle - \overline{\langle a_n\sigma_m^+\sigma_n^z\rangle}\right)
\end{aligned}\tag{6}$$

$$\begin{aligned}
\partial_t\langle\sigma_n^z\sigma_n^z\rangle &= -2(\Gamma_n - 4\Gamma^z)\langle\sigma_n^z\sigma_n^z\rangle + 2(\Gamma_n^\uparrow - \Gamma^\downarrow)\langle\sigma_n^z\rangle + 8g\ \mathrm{Im}\langle a_n\sigma_n^+\sigma_n^z\rangle\\
&\quad - 2\Gamma^{\mathrm{ee}}(\Xi - 2)\langle\sigma_n^z\sigma_n^z\sigma_n^z\rangle,
\end{aligned}\tag{7}$$

where, as explained below, we have dropped the second dipole index, and an overline denotes complex conjugation. Additionally, we observe that $\langle\sigma_n^z\rangle$ couples only to the diagonal elements of $\langle\sigma_n^z\sigma_m^z\rangle$. Hence we derived an equation for $\langle\sigma_n^z\sigma_m^z\rangle$ when $n = m$ only.

To obtain Eqs. (3) to (7), we performed sums over dipoles within each gap, using the fact that dipoles in the same gap share identical on-site properties. Thus, $\langle\sigma_n^z\rangle = \langle\sigma_{n\mathrm{x}}^z\rangle$ can be defined unambiguously for any dipole at n, and the second dipole index was dropped from $\langle a_m\sigma_n^+\rangle$ in the same fashion. The same applies to the dipole-dipole correlations. However, note $\langle\sigma_n^+\sigma_n^-\rangle$ refers to correlations between *distinct* dipoles within the same gap, i.e., $\langle\sigma_{n\mathrm{x}}^+\sigma_{n\mathrm{y}}^-\rangle$ for $\mathrm{x} \neq \mathrm{y}$, not $\langle\sigma_{n\mathrm{x}}^+\sigma_{n\mathrm{x}}^-\rangle = \frac{1}{2}(\langle\sigma_{n\mathrm{x}}^z\rangle + 1)$.

Next, we apply a second-order cumulant expansion [9], which factorizes moments involving more than two operators by setting third-order cumulants to zero, effectively neglecting higher-order correlations. When applying this expansion, we also neglect expectations that do not conserve the total number of excitations (plasmonic plus dipole). The U(1) symmetry of the model ensures that if these expectations are initially zero, they will remain zero for all times.

For example,

$$\langle a_m\sigma_n^+\sigma_m^z\rangle = \langle a_m\sigma_n^+\rangle\langle\sigma_m^z\rangle + \langle a_m\sigma_m^z\rangle\langle\sigma_n^+\rangle + \langle a_m\rangle\langle\sigma_n^+\sigma_m^z\rangle - \langle a_m\rangle\langle\sigma_n^+\rangle\langle\sigma_m^z\rangle \tag{8}$$

follows from $\langle\langle a_m\sigma_n^+\sigma_m^z\rangle\rangle \approx 0$, where double angled brackets denote a cumulant, and then

$$\langle a_m\sigma_n^+\sigma_m^z\rangle = \langle a_m\sigma_n^+\rangle\langle\sigma_m^z\rangle, \tag{9}$$

since $\langle\sigma_n^+\rangle = \langle a_m\rangle = \langle a_m\sigma_n^z\rangle = \langle\sigma_n^+\sigma_m^z\rangle = 0$ on account of the U(1) symmetry.

Similarly, we have $\langle a_n^\dagger a_m \sigma_n^z\rangle \approx \langle a_n^\dagger a_m\rangle\langle\sigma_m^z\rangle$, $\langle\sigma_n^+\sigma_m^-\sigma_n^z\rangle \approx \langle\sigma_n^+\sigma_m^-\rangle\langle\sigma_n^z\rangle$, $\langle a_m\sigma_n^+\sigma_m^z\rangle \approx \langle a_m\sigma_n^+\rangle\langle\sigma_n^z\rangle$, whilst

$$\langle\sigma_n^z\sigma_n^z\sigma_n^z\rangle \approx \langle\sigma_n^z\rangle + 2\langle\sigma_n^z\sigma_n^z\rangle + \langle\sigma_n^z\rangle\left(3\langle\sigma_n^z\sigma_n^z\rangle - 2\langle\sigma_n^z\rangle^2\right). \tag{10}$$

Substituting these expansions into Eqs. (3) to (7) produces a closed set of equations:

$$\begin{aligned}
\partial_t\langle a_n^\dagger a_m\rangle &= -\kappa\langle a_n^\dagger a_m\rangle + ig\Xi\left(\langle a_m\sigma_n^+\rangle - \overline{\langle a_n\sigma_m^+\rangle}\right)\\
&\quad - it\left(\langle a_{n+1}^\dagger a_m\rangle + \langle a_{n-1}^\dagger a_m\rangle - \langle a_n^\dagger a_{m-1}\rangle - \langle a_n^\dagger a_{m+1}\rangle\right)
\end{aligned} \tag{11}$$

$$\partial_t\langle\sigma_n^z\rangle = -\left(\Gamma_n - 4\Gamma^z + 2\Gamma^\Xi\right)\left(\langle\sigma_n^z\rangle - S_0\right) + 4g\ \mathrm{Im}\langle a_n\sigma_n^+\rangle - \Gamma^\Xi\langle\sigma_n^z\sigma_n^z\rangle \tag{12}$$

$$\begin{aligned}
\partial_t\langle a_m\sigma_n^+\rangle &= \left(i\Delta - \frac{\Gamma_n+\kappa+\Gamma^\Xi}{2}\right)\langle a_m\sigma_n^+\rangle - \frac{\Gamma^\Xi}{2}\langle a_m\sigma_n^+\rangle\langle\sigma_n^z\rangle + it\left(\langle a_{m-1}\sigma_n^+\rangle + \langle a_{m+1}\sigma_n^+\rangle\right)\\
&\quad - ig\left[\langle\sigma_n^z\rangle\left(\langle a_n^\dagger a_m\rangle + \frac{\delta_{nm}}{2}\right) + \frac{\delta_{nm}}{2} + \left(\Xi - \delta_{nm}\right)\langle\sigma_n^+\sigma_m^-\rangle\right]
\end{aligned} \tag{13}$$

$$\begin{aligned}
\partial_t\langle\sigma_n^+\sigma_m^-\rangle &= -\left(\frac{\Gamma_n+\Gamma_m+2\Gamma^\Xi}{2}\right)\langle\sigma_n^+\sigma_m^-\rangle - \frac{\Gamma^\Xi}{2}\langle\sigma_n^+\sigma_m^-\rangle\left(\langle\sigma_n^z\rangle + \langle\sigma_m^z\rangle\right)\\
&\quad + \Gamma^{\mathrm{ee}}\delta_{nm}\langle\sigma_n^+\sigma_n^-\rangle\left(1 + \langle\sigma_n^z\rangle\right) + ig\left(\langle a_m\sigma_n^+\rangle\langle\sigma_m^z\rangle - \overline{\langle a_n\sigma_m^+\rangle}\langle\sigma_n^z\rangle\right)
\end{aligned} \tag{14}$$

$$\begin{aligned}
\partial_t\langle\sigma_n^z\sigma_n^z\rangle &= -2(\Gamma_n - 4\Gamma^z)\langle\sigma_n^z\sigma_n^z\rangle + 2(\Gamma_n^\uparrow - \Gamma^\downarrow)\langle\sigma_n^z\rangle + 8g\langle\sigma_n^z\rangle\ \mathrm{Im}\langle a_n\sigma_n^+\rangle\\
&\quad - 2\Gamma^{\mathrm{ee}}(\Xi - 2)\left(\langle\sigma_n^z\rangle + 2\langle\sigma_n^z\sigma_n^z\rangle + \langle\sigma_n^z\rangle\left[3\langle\sigma_n^z\sigma_n^z\rangle - 2\langle\sigma_n^z\rangle^2\right]\right).
\end{aligned} \tag{15}$$

Denoting $n_{nm} = \langle a_n^\dagger a_m\rangle$ the plasmon-plasmon correlation between sites, $S_n = \langle\sigma_n^z\rangle$ the dipole inversion ($S_n \in [-1, 1]$), $P_{mn} = \langle a_m\sigma_n^+\rangle$ the plasmon-dipole correlations, $D_{nm} = \langle\sigma_n^+\sigma_m^-\rangle$ the dipole-dipole correlations, and $Z_n = \langle\sigma_n^z\sigma_n^z\rangle$ the spin-spin correlations, we obtain quantities presented in the article.

S3.3 Observables

We now explain how observables were calculated from Eqs. (11) to (15) for the theoretical data presented in Figs. 1-4 of the article. The parameter values used in these calculations are summarized in Table 1.

Description	Symbol	Value
Nanoparticle number	M	123
Dipoles per gap	Ξ	2, 23, 225
Chain length	L	9963 μ m
Detuning	Δ	0
Dipole-plasmon coupling	g	10 meV ($\hbar = 1$)
Hopping strength	J	0.45 eV ($\hbar = 1$)
Pump strength	$\Gamma_n^{\uparrow}$	Varies
Pump width	σ	2 gaps
Dipole Decay	$\Gamma^{\downarrow}$	10^{-4} eV = 0.15 ps^{-1}
Pure Dephasing	Γ^z	0
Pair annihilation	Γ^{ee}	10^{-4} eV = 0.15 ps^{-1}
Plasmonic decay	κ	10^{-1} eV = 0.15 fs^{-1}

Table 1: Model Parameters.

All calculations were based on the steady-state properties of the model, obtained by numerically evolving Eqs. (11) to (15) from initial conditions $\langle \sigma_n^z \rangle = -1$, $\langle \sigma_n^z \sigma_n^z \rangle = 1$, and all other expectations zero, until the expectations reached a stationary value. To evaluate the dependence on pump strength, the steady-state was obtained for Gaussian pump profiles $\Gamma_n^{\uparrow} = Pe^{-n^2/(2\sigma^2)}$, with different powers P. The centre of the chain is at $n = 0$, and the fixed pump width is $\sigma = 2$ with a linewidth/full-width-at-half-maximum (FWHM) of $2\ln(2) \cdot 2 = 5$ gaps or 405 nm.

For the emission profile in Fig. 1g, we plot (solid curves) the steady-state plasmon populations $N_{nn} = \langle a_n^{\dagger} a_n \rangle$ as a function of distance R_n from the center ($n = 0$) for two pump powers $P = \Gamma^{\downarrow}$ below and above the threshold $P_{\text{th}} \approx 10^4 \Gamma^{\downarrow}$. Note that these curves are normalized to the emission at $R_n = 0$.

The steady-state emission profiles for various pump powers P were calculated for Fig. 3a, where the total emission $\sum_n N_{nn}$ across all sites is plotted against P. Fig. 4 further shows the steady-state emission N_{nn} and spin-spin correlations D_{nn} and Z_n as functions of position R_n and pump magnitude P (panels C,D).

For the first-order coherence function $g^{(1)}(R, \tau = 0)$, plotted in Fig. 2c, we calculate

$$g^{(1)}(R_n) = \frac{\langle a_n^{\dagger} a_{-n} \rangle}{\sqrt{\langle a_n^{\dagger} a_n \rangle \langle a_{-n}^{\dagger} a_{-n} \rangle}}. \tag{16}$$

In Fig. 2c, $g^{(1)}$ is plotted as a function of R_n for the target power $P = 10^4 \Gamma^{\downarrow} = 0.13$~mW and two dipole concentrations per gap ($\Xi = 2$, red thin line, and $\Xi = 225$, blue thin line). To estimate the core coherence $g^{(1)}(R = 0)$ shown in Fig. 2d, we averaged $g^{(1)}(R_n, \tau = 0)$ over 3 pairs of sites around zero:

$$g^{(1)}(R = 0) = \frac{1}{3}\left(g^{(1)}(\Delta R) + g^{(1)}(2\Delta R) + g^{(1)}(3\Delta R)\right). \tag{17}$$

We note that this definition means the measured $g^{(1)}(R=0,\tau=0)$ is obtained over a diffraction-limited region containing multiple nanogap emitters. In the absence of mutual coherence, spatial averaging reduces the measured visibility below unity. The increase of $g^{(1)}(R=0,\tau=0)$ with pump therefore reflects the development of inter-emitter phase correlations rather than a deviation from the formal definition of first-order coherence.

We note that the calculated dynamics provide single-time expectations only. Evaluating quantities such as the first-order coherence as a function of delay, $g^{(1)}(R,\tau)$ in Figs. 2E,F, requires calculating two-time correlations. The correct calculation of two-time correlations and spectra following a second-order cumulant expansion is an interesting problem but beyond the scope of our current model.The main observables for the 1D model for the 0.01 MB:CB7 nanocavity array are shown in Supplementary Figure S4 below.

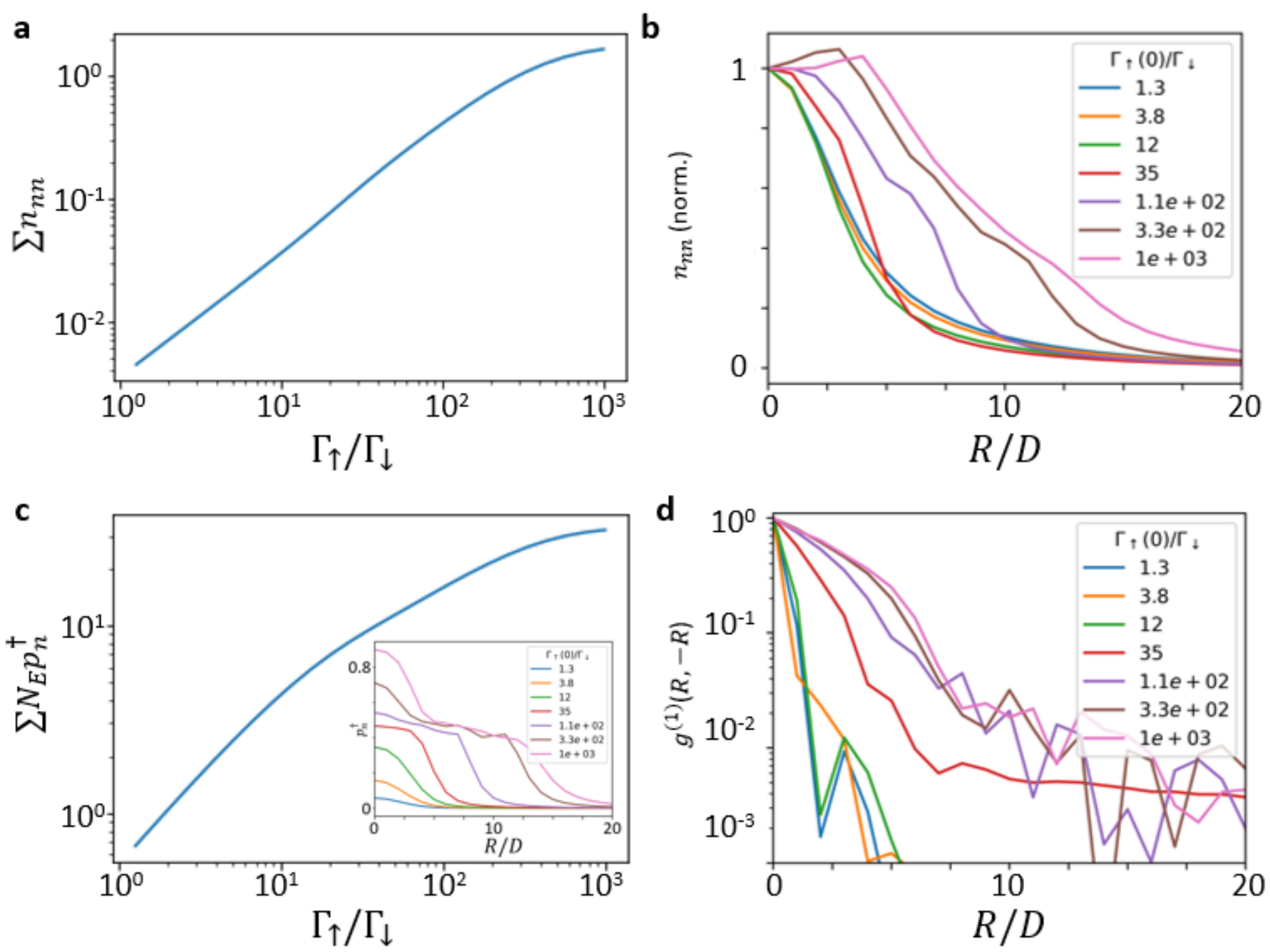

Supplementary Figure S4: **1D tight-binding model coherence and photon correlation. a,** Input-output relation for total summed photon number n_{nn} against pumping rate $\Gamma_\uparrow$ normalized with non-radiative decay $\Gamma_\downarrow$ **b,** Spatial profile of photon number n_{nn} against position R/D where D is the nanoparticle diameter for different pumping rates. **c,** Sum of emitter polarization against pumping rate $\Gamma_\uparrow$ normalized with non-radiative decay $\Gamma_\downarrow$. Inset shows the polarization as a function of position R/D for different pumping rates, with colors indicated by the legend in b. **d,** Spatial dependence of $g^{(1)}(R,-R)$ coherence for different pumping rates, showing a spatial spreading and growth in coherence.

S3.4 Tight-binding and full plasmonic chain dispersion

The effective dispersion of the tight-binding model is very similar to the modes of the exact 1D plasmonic chain [10] including plasmon-photon coupling to second-order perturbation theory, as shown in (Supplementary Figure **S5**) below. The exact dispersion is reproduced below and compared to the approximate tight-binding dispersion used in the model above. Both dispersion relations correspond well to each other, enabling the use of the real-space tight binding model above, which is more tractable to computational solution with the cumulant equations and yields further insight in real space v.s. reciprocal space.

Below, $\tilde{\omega}_k^\sigma$ is the exact plasmonic mode energy, ω_p is the plasmon energy resonant frequency set at 1.87 eV, $\Omega = \frac{\omega_0}{2}(\frac{a}{d})^3$ is the long-range dipolar coupling strength between plasmons, $a = 40$ nm is the nanoparticle radius, d=1 nm is the nanoparticle gap-size, c is the speed of light, $\Delta r = 2a + d$ is the centre-to-centre particle spacing, and k is the wavevector from $-\pi M/L$ to $\pi M/L$, where M is the number of particles in the chain and $L = M\Delta r$ is the length of the chain.

$$\tilde{\omega}_k^\sigma = \omega_k^\sigma + \delta_k^\sigma, \tag{18}$$

$$\omega_k^\sigma = \omega_p\sqrt{1 + 2\frac{\Omega}{\omega_0}f_q^\sigma}, \tag{19}$$

$$f_q^\sigma = \eta^\sigma\left[\mathrm{Li}_3(e^{ik\Delta r}) + \mathrm{Li}_3(e^{-ik\Delta r})\right] \tag{20}$$

$$\delta_k^\sigma = \frac{\eta^\sigma}{2}\frac{\omega_p^2}{\omega_k^\delta}\frac{k^2a^3}{\Delta r}\Theta\left(1/a - |k|\right)\left\{\ln\left(\frac{1}{ka}\right) + \frac{1}{2}\left[1 + \mathrm{sgn}(\eta^\sigma)\left(\frac{\omega_k^\sigma}{kc}\right)^2\right]\ln\left(\frac{\left|(kc)^2 - (\omega_k^\sigma)^2\right|}{(c/a)^2 - (\omega_k^\sigma)^2}\right)\right\} \tag{21}$$

Here $\eta^\sigma = 1$ (2) for transverse (longitudinal) modes, $\mathrm{Li}_3(z) = \sum_{n=1}^{\infty} z^n/n^3$ is the polylogarithm function. Note $k_pa \sim 0.38$ and the cutoff $1/a = 0.025$ nm^{-1} with the above parameters.

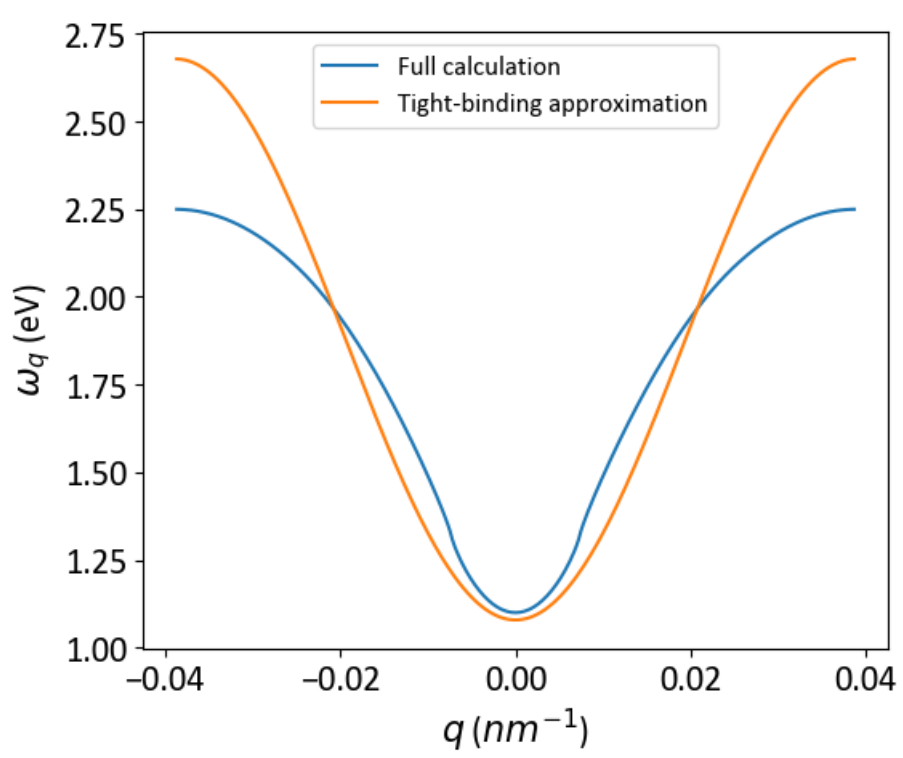

Supplementary Figure S5: **Tight-binding v.s. full analytical ($\tilde{\omega}_k^\sigma$) dispersion relation for a 1D plasmonic chain**. Parameters for calculation in SI Section S3.4 and in the main text.

S3.5 Instability with zero pair annihilation rate

The presence of a two-body interaction producing pair-annihilation parameterised by Γ^{ee} is critical to enable the spreading of correlations and the suppression of instability in the photon number at high pumping rates. This is seen in Supplementary Figure S6b, where changing to Γ^{ee}=0 induces an irregular photon number profile in the dotted blue line. In addition to the irregular dependence on position, the photon distribution in this case does not reach a steady state, showing fluctuations and irregular jumps in Supplementary Figure S6c.

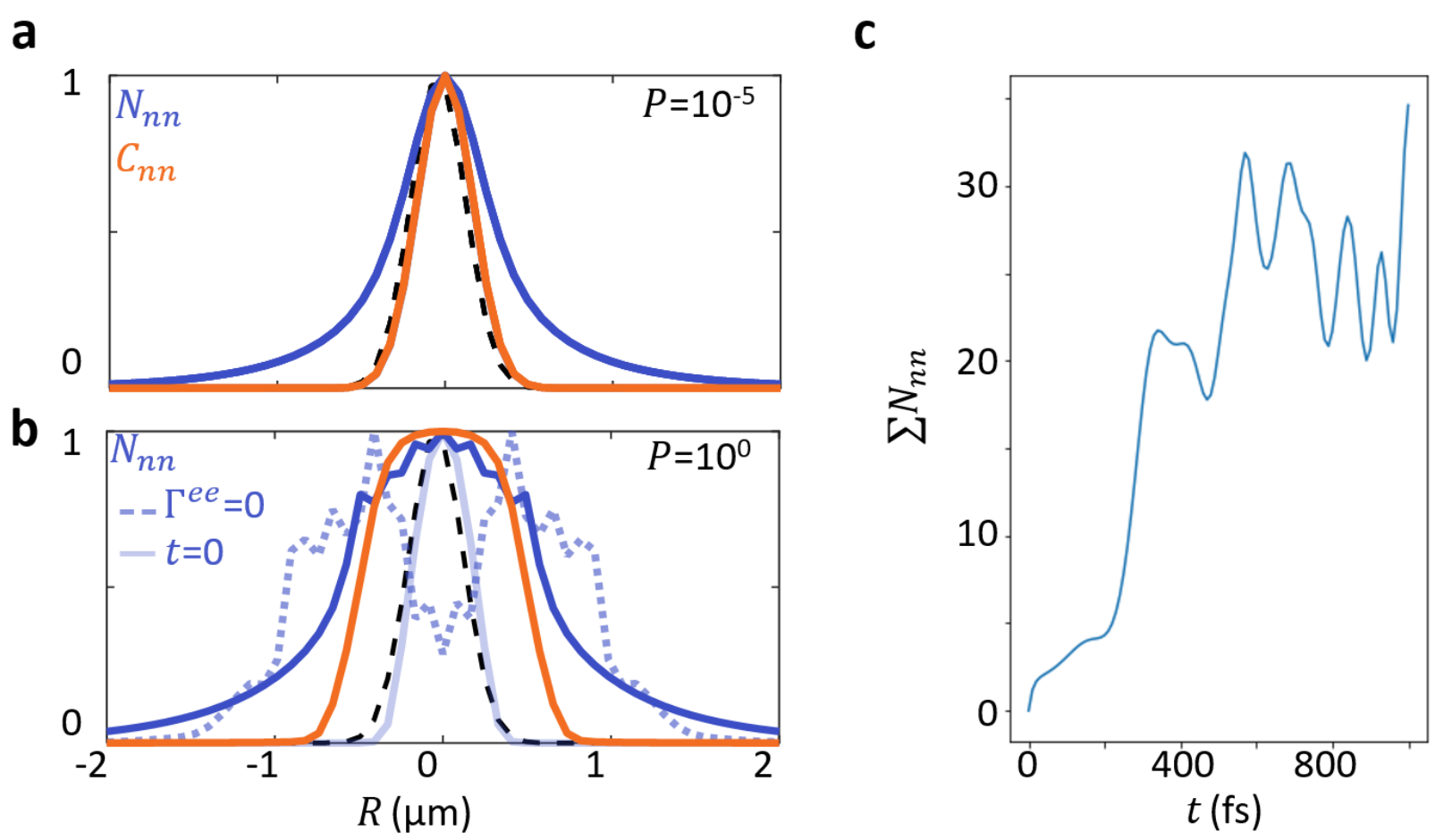

Supplementary Figure S6: **Effect of pair annihilation rates (Γ^{ee})** normalized photon-emission profile (N_{nn}, blue) and on-site dipole correlations (C_{nn}, orange) for pump profile (black) at **a,** low pump power $P = 10^{-5}$ and **b,** at high pump power $P = 100$, for $t = 0.45$ eV and $\Gamma^{ee} = 0$ or 10^{-4} eV, for Ξ=100. **c,** Sum of N_{nn} for simulation in b with Γ^{ee}=0 with time of simulation showing lack of convergence of the photon number.

S3.6 Pair/exciton-exciton annihilation

Under strong laser illumination, two singlet excitons may be found in adjacent molecules within a nanogap. In such a case the two excited state excitons in S_1 may non-radiatively annihilate to promote one exciton to the second excited state while de-exciting the first exciton. The resulting state then rapidly decays back down non-radiatively following Kasha's rule, prior to emission of a photon into the nanocavity field. This is a pair annihilation process as it takes two emissive dipoles/excitons, and via this higher order process only retains one, which re-emits a photon back into the cavity. This has been observed in many condensed phase organic molecules within crystals and thin films [11, 12], 2D materials [13], and optical cavities [14]. Within the highly dilute limit observed in our experiments, we expect that the plasmonic field may also be assisting dipole-dipole interactions and hence increasing the strength of the pair annihilation rate Γ^{ee}, following the theoretical treatment by May et al. [15].

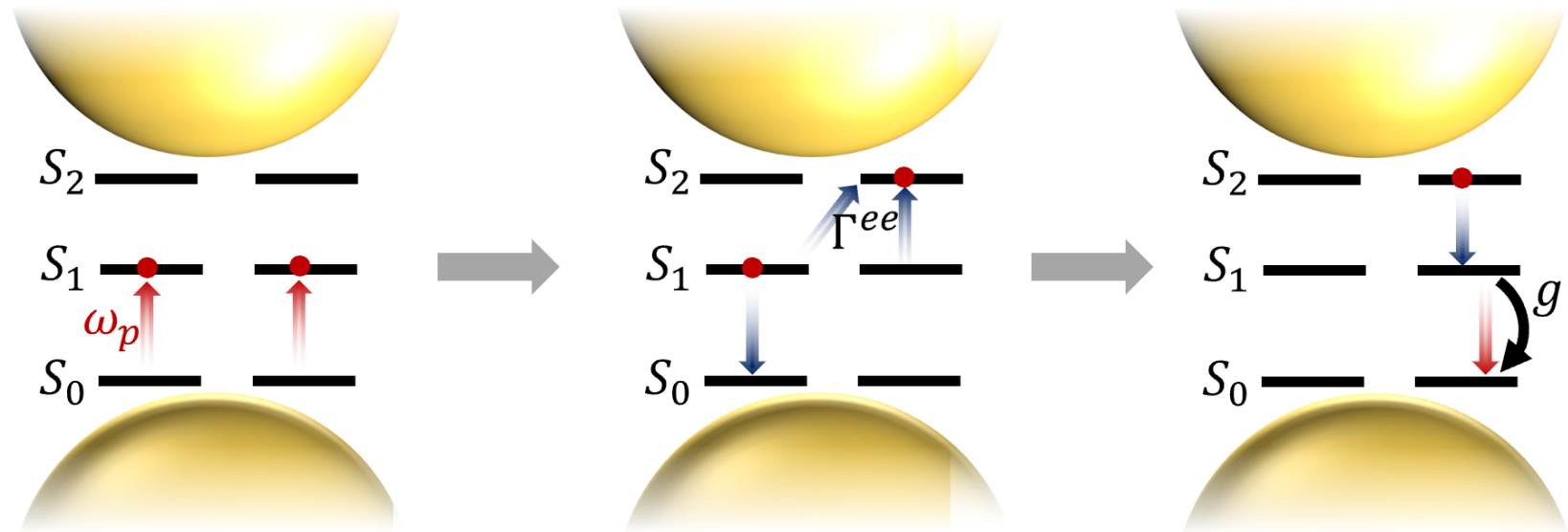

Supplementary Figure S7: **Exciton-exciton pair annihilation schematic within nanogap.** Initially, two adjacent molecules within a nanogap may be excited by an incident pump photon from the singlet exciton ground state S_0 to the first excited state S_1. Under high density of excitons, two S_1 excitons may non-radiatively annihilate with a rate Γ^{ee}, promoting one exciton to the second excited state S_2 while de-exciting the first one. The S_2 state then non-radiatively decays to S_1 prior to emission and coupling to the photonic field (coupling strength g).

S3.7 2D Model

To extend the 1D tight-binding model presented in Section S3.1 to two dimensions, we consider a square lattice with nearest-neighbour interactions such that each site is indexed by a pair $\boldsymbol{n} = (n_x, n_y)$ of integers (position $\boldsymbol{r_n}$) with corresponding wavevectors $\boldsymbol{k} = (k_x, k_y)$ in reciprocal space. The plasmonic dispersion is

$$\omega_{\boldsymbol{k}} = \omega_c - 2t\left(\cos(k_x \Delta r) + \cos(k_y \Delta r)\right), \tag{22}$$

The plasmonic 2D dispersion is shown in Supplementary Figure **S8**a, and the pump spot $\Gamma_n^{\uparrow} \to \Gamma_{\boldsymbol{n}}^{\uparrow} \sim e^{-(n_x^2+n_y^2)/(2\sigma_0^2)}$ becomes a two-dimensional Gaussian centred at $\boldsymbol{r} = \boldsymbol{0}$ (see Supplementary Figure **S8**b).

The derived equations of motion have the same form as before (cf. Eqs. (11) to (15)), and we

sum over all neighbours of sites when determining the hopping terms:

$$\begin{aligned}
\partial_t \langle a^\dagger_{\boldsymbol{n}} a_{\boldsymbol{m}}\rangle &= -\kappa \langle a^\dagger_{\boldsymbol{n}} a_{\boldsymbol{m}}\rangle + ig\Xi \left(\langle a_{\boldsymbol{m}} \sigma^+_{\boldsymbol{n}}\rangle - \overline{\langle a_{\boldsymbol{n}} \sigma^+_{\boldsymbol{m}}\rangle}\right) \\
&\quad - it\left(\langle a^\dagger_{(n_x+1,n_y)} a_{\boldsymbol{m}}\rangle + \langle a^\dagger_{(n_x-1,n_y)} a_{\boldsymbol{m}}\rangle + \langle a^\dagger_{(n_x,n_y+1)} a_{\boldsymbol{m}}\rangle + \langle a^\dagger_{(n_x,n_y-1)} a_{\boldsymbol{m}}\rangle\right) \\
&\quad + it\left(\langle a^\dagger_{\boldsymbol{n}} a_{(m_x+1,m_y)}\rangle + \langle a^\dagger_{\boldsymbol{n}} a_{(m_x-1,m_y)}\rangle + \langle a^\dagger_{\boldsymbol{n}} a_{(m_x,m_y+1)}\rangle + \langle a^\dagger_{\boldsymbol{n}} a_{(m_x,m_y-1)}\rangle\right)
\end{aligned} \tag{23}$$

$$\partial_t \langle \sigma^z_{\boldsymbol{n}}\rangle = -\left(\Gamma_{\boldsymbol{n}} - 4\Gamma^z + 2\Gamma^\Xi\right)\left(\langle \sigma^z_{\boldsymbol{n}}\rangle - S_0\right) + 4g\,\mathrm{Im}\langle a_{\boldsymbol{n}} \sigma^+_{\boldsymbol{n}}\rangle - \Gamma^\Xi \langle \sigma^z_{\boldsymbol{n}} \sigma^z_{\boldsymbol{n}}\rangle \tag{24}$$

$$\begin{aligned}
\partial_t \langle a_{\boldsymbol{m}} \sigma^+_{\boldsymbol{n}}\rangle &= \left(i\Delta - \frac{\Gamma_{\boldsymbol{n}} + \kappa + \Gamma^\Xi}{2}\right)\langle a_{\boldsymbol{m}} \sigma^+_{\boldsymbol{n}}\rangle - \frac{\Gamma^\Xi}{2}\langle a_{\boldsymbol{m}} \sigma^+_{\boldsymbol{n}}\rangle\langle \sigma^z_{\boldsymbol{n}}\rangle \\
&\quad + it\left(\langle a_{(m_x+1,m_y)} \sigma^+_{\boldsymbol{n}}\rangle + \langle a_{(m_x-1,m_y)} \sigma^+_{\boldsymbol{n}}\rangle + \langle a_{(m_x,m_y+1)} \sigma^+_{\boldsymbol{n}}\rangle + \langle a_{(m_x,m_y-1)} \sigma^+_{\boldsymbol{n}}\rangle\right) \\
&\quad - ig\left[\langle \sigma^z_{\boldsymbol{n}}\rangle\left(\langle a^\dagger_{\boldsymbol{n}} a_{\boldsymbol{m}}\rangle + \frac{\delta_{\boldsymbol{nm}}}{2}\right) + \frac{\delta_{\boldsymbol{nm}}}{2} + \left(\Xi - \delta_{\boldsymbol{nm}}\right)\langle \sigma^+_{\boldsymbol{n}} \sigma^-_{\boldsymbol{m}}\rangle\right]
\end{aligned} \tag{25}$$

$$\begin{aligned}
\partial_t \langle \sigma^+_{\boldsymbol{n}} \sigma^-_{\boldsymbol{m}}\rangle &= -\left(\frac{\Gamma_{\boldsymbol{n}} + \Gamma_{\boldsymbol{m}} + 2\Gamma^\Xi}{2}\right)\langle \sigma^+_{\boldsymbol{n}} \sigma^-_{\boldsymbol{m}}\rangle - \frac{\Gamma^\Xi}{2}\langle \sigma^+_{\boldsymbol{n}} \sigma^-_{\boldsymbol{m}}\rangle\left(\langle \sigma^z_{\boldsymbol{n}}\rangle + \langle \sigma^z_{\boldsymbol{m}}\rangle\right) \\
&\quad + \Gamma^{\mathrm{ee}}\delta_{\boldsymbol{nm}}\langle \sigma^+_{\boldsymbol{n}} \sigma^-_{\boldsymbol{n}}\rangle\left(1 + \langle \sigma^z_{\boldsymbol{n}}\rangle\right) + ig\left(\langle a_{\boldsymbol{m}} \sigma^+_{\boldsymbol{n}}\rangle\langle \sigma^z_{\boldsymbol{m}}\rangle - \overline{\langle a_{\boldsymbol{n}} \sigma^+_{\boldsymbol{m}}\rangle}\langle \sigma^z_{\boldsymbol{n}}\rangle\right)
\end{aligned} \tag{26}$$

$$\begin{aligned}
\partial_t \langle \sigma^z_{\boldsymbol{n}} \sigma^z_{\boldsymbol{n}}\rangle &= -2\left(\Gamma_{\boldsymbol{n}} - 4\Gamma^z\right)\langle \sigma^z_{\boldsymbol{n}} \sigma^z_{\boldsymbol{n}}\rangle + 2\left(\Gamma^\uparrow_{\boldsymbol{n}} - \Gamma^\downarrow\right)\langle \sigma^z_{\boldsymbol{n}}\rangle + 8g\langle \sigma^z_{\boldsymbol{n}}\rangle\,\mathrm{Im}\langle a_{\boldsymbol{n}} \sigma^+_{\boldsymbol{n}}\rangle \\
&\quad - 2\gamma^{\mathrm{ee}}(\Xi - 2)\left(\langle \sigma^z_{\boldsymbol{n}}\rangle + 2\langle \sigma^z_{\boldsymbol{n}} \sigma^z_{\boldsymbol{n}}\rangle + \langle \sigma^z_{\boldsymbol{n}}\rangle\left[3\langle \sigma^z_{\boldsymbol{n}} \sigma^z_{\boldsymbol{n}}\rangle - 2\langle \sigma^z_{\boldsymbol{n}}\rangle^2\right]\right)
\end{aligned} \tag{27}$$

The observables $n_{nm} = \langle a^\dagger_n a_m\rangle$, $S_n = \langle \sigma^z_n\rangle$, $P_{mn} = \langle a_m \sigma^+_n\rangle$, $D_{nm} = \langle \sigma^+_n \sigma^-_m\rangle$ and $Z_n = \langle \sigma^z_n \sigma^z_n\rangle$ directly generalise to 2D.

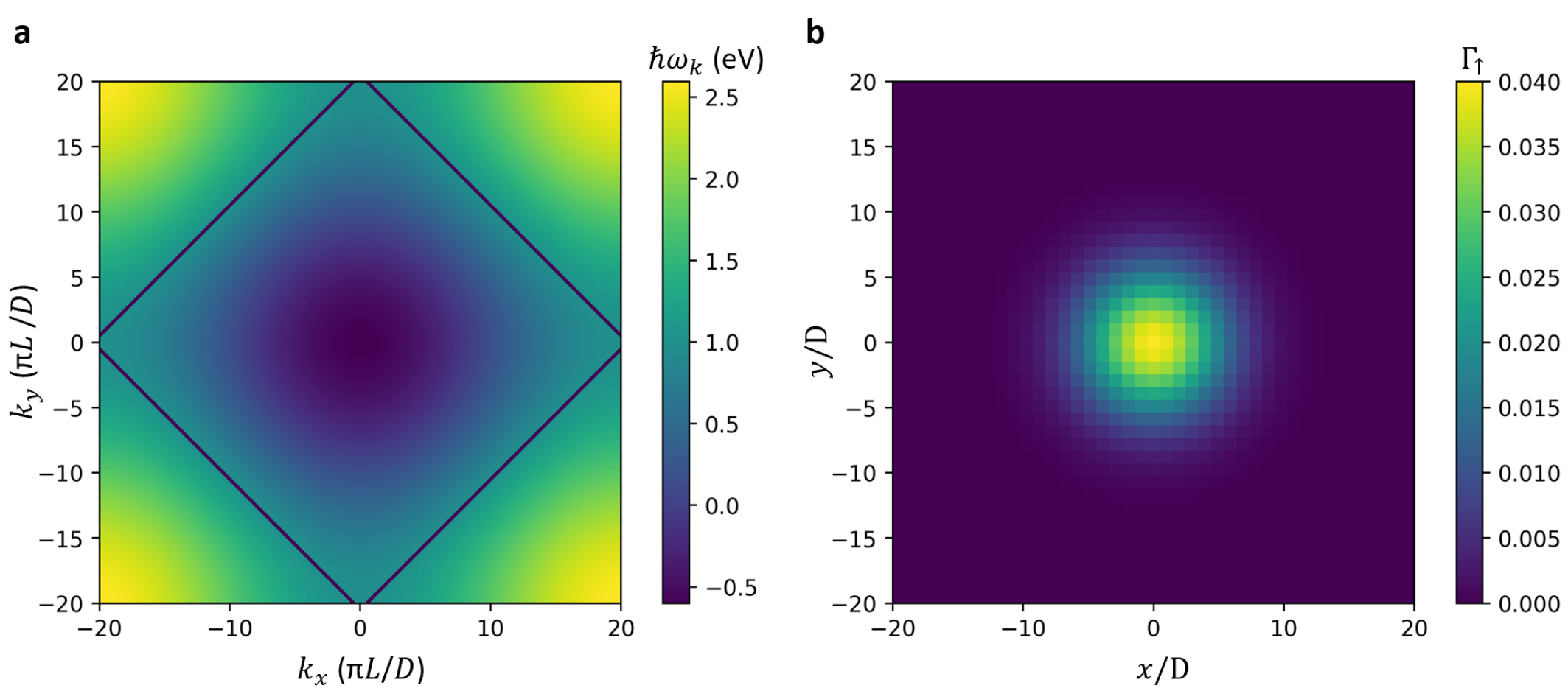

Supplementary Figure S8: **2D tight-binding model. a,** Dispersion Eq. (22) of the 2D plasmonic modes of energy $\hbar\omega_{\boldsymbol{k}}$ as a function of in-plane momentum k for a square lattice of an array of 41×41 nanoparticles ($L = 41\Delta r$). The dark contour indicates $\hbar\omega_{\boldsymbol{k}} = \hbar\omega_0$. **b,** Real-space profile of the pumped region of the nanoparticle array with incoherent pumping magnitude $\Gamma_\uparrow$.

The resulting input-output curves for a range of pump powers are shown in Supplementary Figure S9a, with a clear saturation behaviour at higher powers. The photon number profile spreads out spatially, with increasing pump powers (Supplementary Figure S9b), and the resulting sum of on-site polarization also increases spatially and in magnitude (Supplementary Figure S9c). This results in an increased first order coherence function $g^{(1)}(R, \tau = 0)$, which is calculated as an average over all antipodal pairs of sites a distance R away from the centre $\boldsymbol{r}_n = \mathbf{0}$ of the lattice. The photon number distribution in real and k-space is also shown in Supplementary Figure S10, displaying an increase in the spatial size in 2D. These observations clearly show similar behaviour to the 1D case, shown in the main text figures, and provides confidence that the 1D approximation captures the relevant physics of the problem. All the parameters remain the same as for the 1D case, as shown in Table 1.

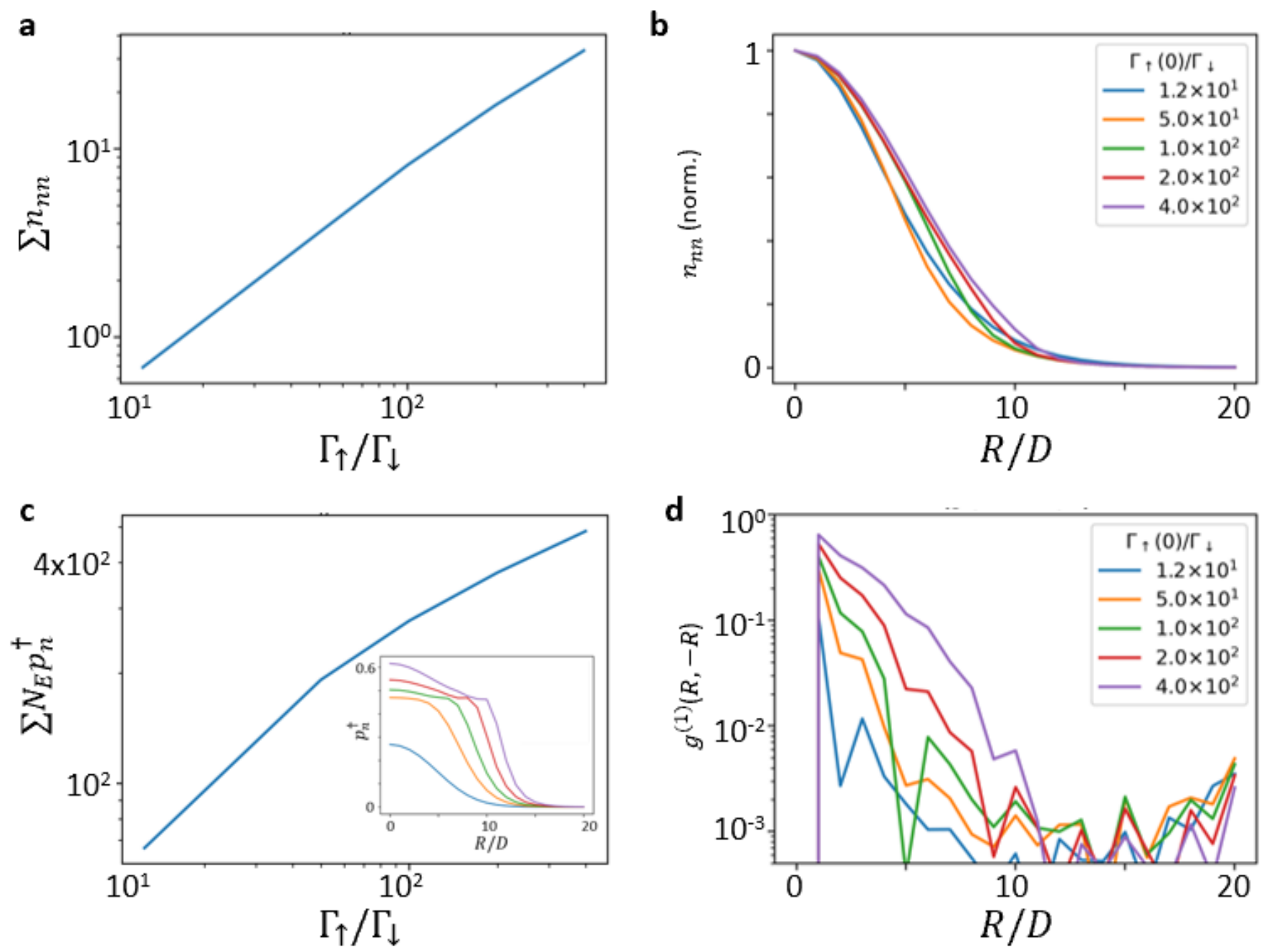

Supplementary Figure S9: **2D tight-binding model coherence and photon correlation.** **a,** Input-output relation for total summed photon number n_{nn} against pumping rate $\Gamma_\uparrow$ normalized with non-radiative decay $\Gamma_\downarrow$ **b,** Spatially averaged radial profile of photon number n_{nn} against position R/D where D is the nanoparticle diameter for different pumping rates. **c,** Sum of emitter polarization against pumping rate $\Gamma_\uparrow$ normalized with non-radiative decay $\Gamma_\downarrow$. Inset shows the polarization as a function of position R/D for different pumping rates, with colors indicated by the legend in b. **d,** Spatial dependence of $g^{(1)}(R, -R)$ coherence for different pumping rates, showing a spatial spreading and growth in coherence. This figure can be compared directly to Fig. S4 which shows analogous results for the 1D case.

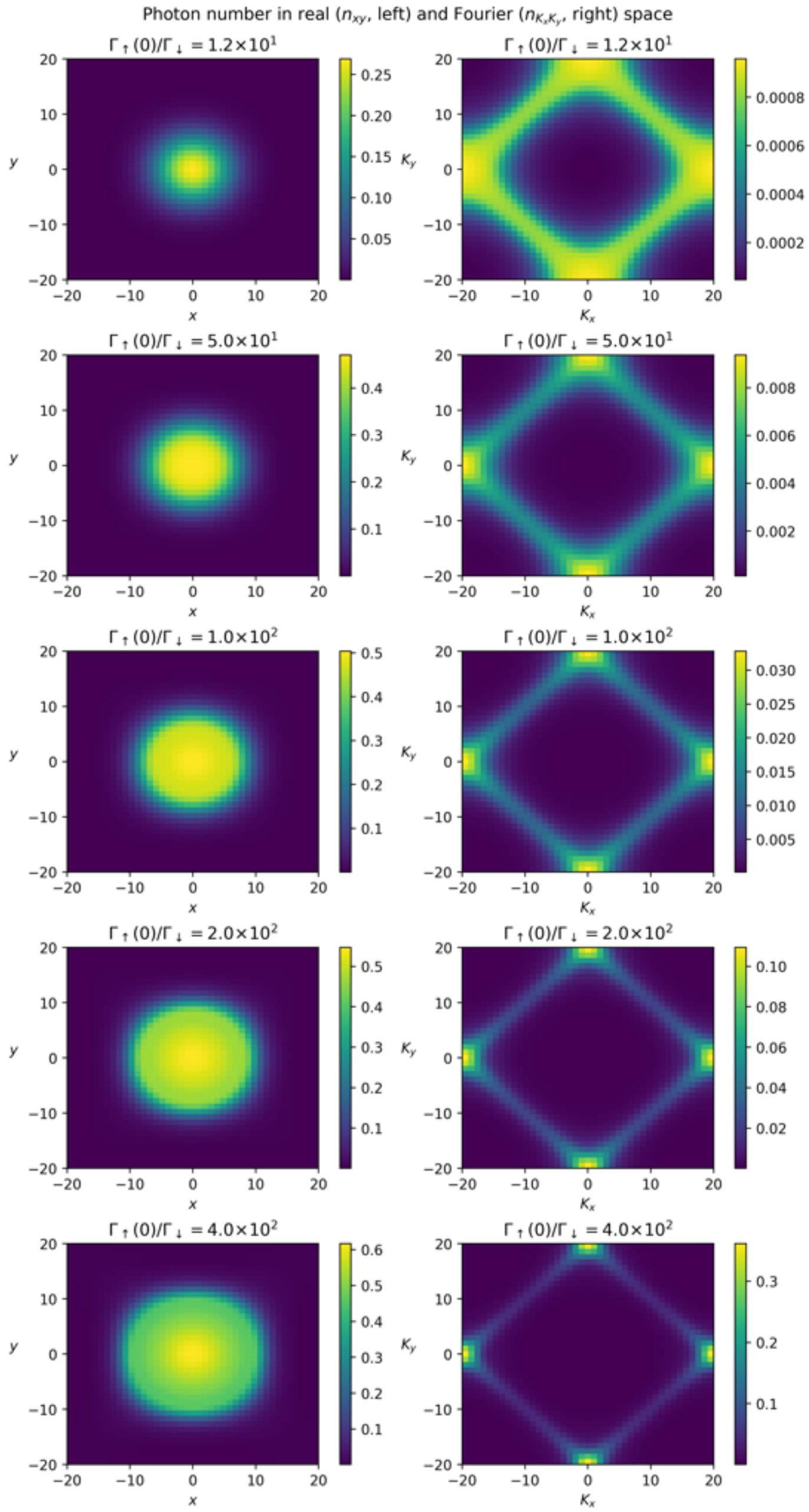

Supplementary Figure S10: **Photon number distribution.** In real space on the left in units of R/D, and in k-space on the right in units of $\pi L/D$ for a range of pumping strengths.

S3.8 Limitations of theoretical model

The theoretical calculations shown do not fit all our data perfectly. The model presented is a simple model intended to capture the key features of the physics. We note here some aspects of the physics of the experiment that may be missed by the simplified theoretical model:

- Plasmonic mode approximations: We represent the collective nanoplasmonic modes in a tight-binding model and parabolic band. The actual system may have more than one relevant plasmonic mode per nanoparticle. Only nearest-neighbour interactions are considered, when next-nearest neighbour and longer-range interactions may play a role.
- Emitter approximations: The complex organic molecule is represented as a singlet exciton and two-level system, which does not account for the role of the triplet exciton dark states [16].
- Cumulant expansion solution scaling with system size: The full quantum model is very costly to solve exactly, and hence we utilize the cumulant expansion method with a finite number of emitters. The finite number of emitters may lead to notable differences to the cumulant results which should become accurate in the thermodynamic limit $\Xi \to \infty$.
- Numerical instability at high pump powers: These methods are limited in that they are prone to become unstable at high pumping rates (Supplementary Figure **S6**).

SI Section S4. Interferometer calibration and coherence measurement protocol

A Michelson interferometer in the mirror-retroreflector configuration was used to measure the spatio-temporal coherence of light emission as a function of pump power and emitter density in the sample. Supplementary Figure **S11** shows a schematic of the optical configuration, with the retroreflector mounted on a motorized delay line, as used extensively in the polariton physics community [17–22]. Each arm of the interferometer was calibrated by a laser, which only showed coherent interference fringes when overlapped by a lens on the imaging plane of a CMOS camera. The interference fringe spacing was set by the distance between the two laser spots on the entrance pupil of the imaging lens (which imposes a phase difference $\Delta\Phi$ as in Eq. (28)). At each time delay on the motorized stage, an interferogram is collected, where the depth and spatial extent of fringes can be analyzed to extract the visibility as a function of time and space, from which the first-order coherence $g^{(1)}(R,\tau)$ can be inferred, where $R = \sqrt{x^2+y^2}$ is the radial coordinate and τ is the time delay. The zero-delay time was calibrated by sending white light from a broadband white light illuminator on the Olympus BX51 microscope, or the incoherent emission from a 2D photoluminescent semiconductor (WSe_2) to the interferometer and measuring the interferogram with time.

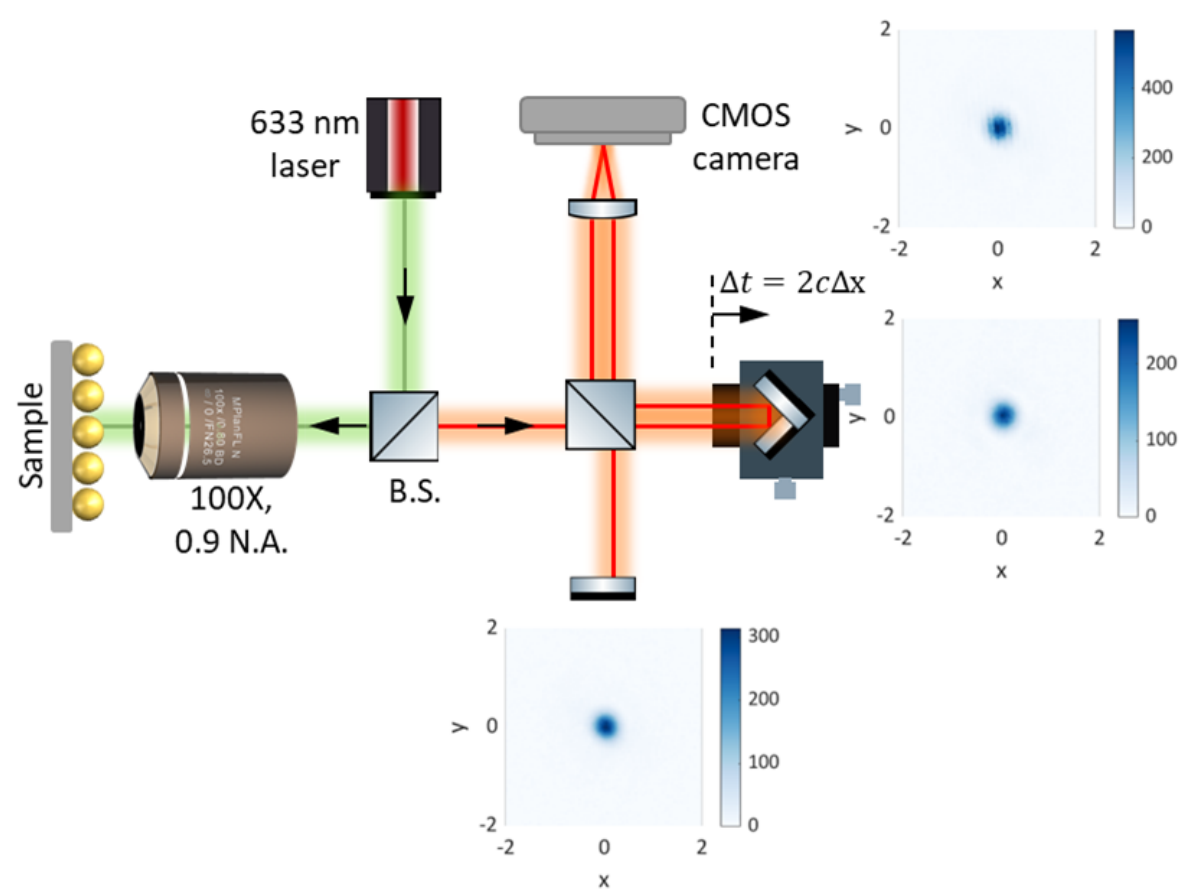

Supplementary Figure S11: **Michelson interferometer.** Schematic of the Michelson interferometer setup with sample measured with microscope objective sent to automated delay stage and overlapped with a lens on the imaging plane. Insets show the intensity (in counts/s) image of the pump laser from each arm with each arm blocked to observe the intensity within each arm prior to overlap and interference at time-zero.

The visibility of fringes can be observed for the 1 MB:CB emitter density at a pump power above threshold in Supplementary Figure **S12**a,b below, which clearly has a larger spatial extent than the corresponding spatial coherence of the pump laser in Supplementary Figure **S12**c,d. The interferograms in Supplementary Figure **S12** were all collected at zero time delay (τ=0). While the synchronized excitons have a larger spatial range of coherence, the depth of the fringe visibility for the pump laser is larger, as expected for a highly spatially and temporally coherent system.

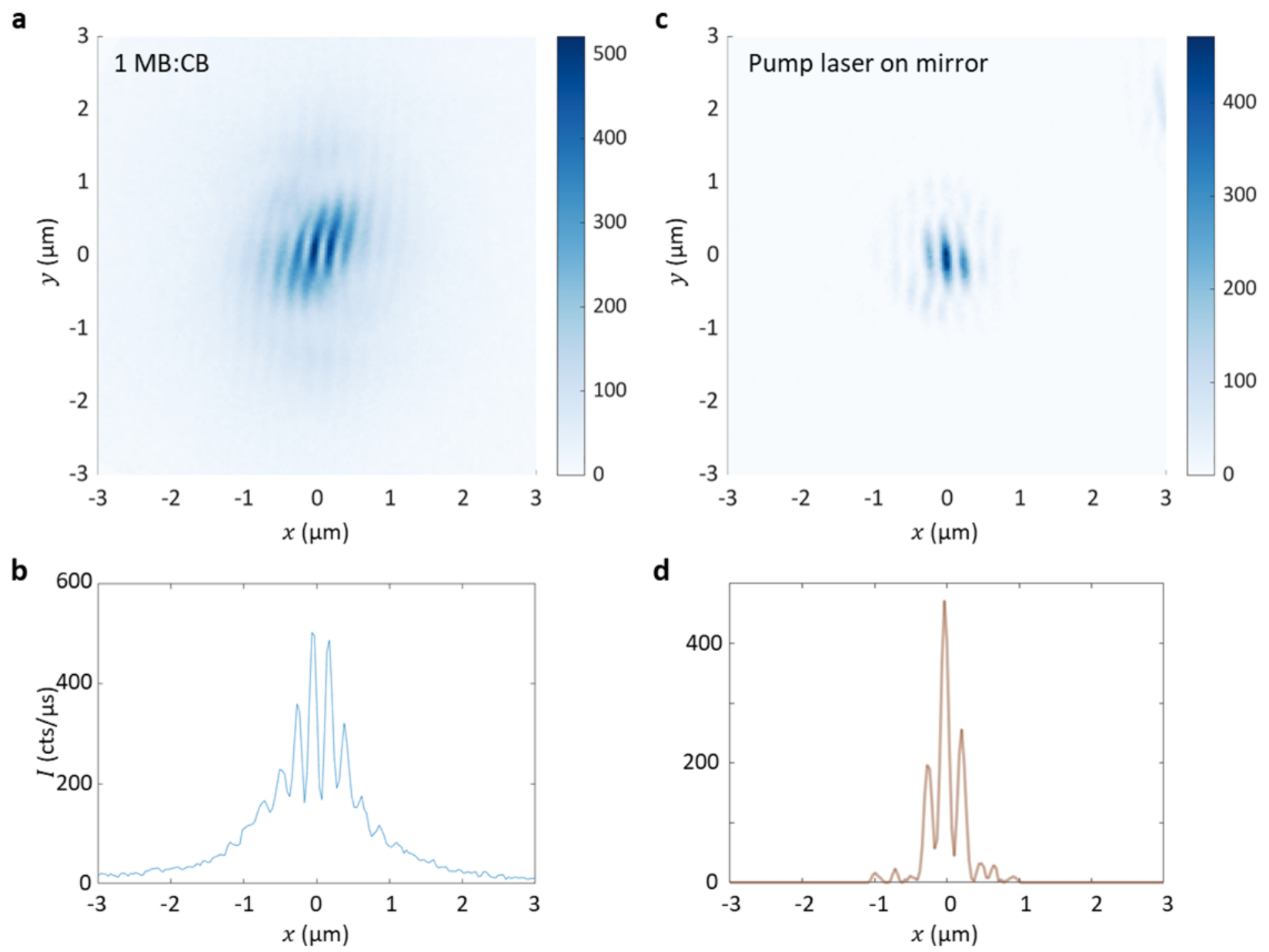

Supplementary Figure S12: **Interferogram of sample emission and pump laser at time-zero (τ=0). a,** Emission interferogram (in counts/s) from the synchronized excitons with 1 MB:CB emitter density, and **b,** Line-cut at y=0 of the emission interferogram. **c,** Pump laser (λ = 633 nm) interferogram (in counts/s), and **d,** Line-cut at y=0 of the pump laser interferogram. Scale for images in a, c are in counts on the CMOS detector. Pump laser intensity of 200 $\mu W/\mu m^{-2}$.

In order to extract the visibility and $g^{(1)}(R,\tau)$ from the interferograms I_{tot}, the real-space interferogram was processed using Fourier filtering. As one arm of the interferometer is a corner-cube retroreflector, the image on that arm is inverted about its centre, i.e. $x, y \to -x, -y$ or $R \to -R$. Hence the interferogram as a function of time delay τ is represented by [18]:

$$I_{tot} = \frac{1}{4}[I(R) + I(-R) + 2\sqrt{I(R)I(-R)}|g^{(1)}(R,\tau)|\cos(\Delta\Phi)] \tag{28}$$

where $\Delta\Phi$ is the relative phase between the two interfered beams due to the angle between them after the imaging lens and $I(R) = \langle|E(r,t)|^2\rangle_t$ is the time-averaged emission intensity. The first-order correlation function can then be approximated by measuring the visibility, $V = (I_+ - I_-)/(I_+ + I_-)$ where I_+ and I_- are the upper and lower envelopes of the measured interferogram. The visibility is exactly related to the first-order coherence via [23]:

$$V(R,\tau) = \frac{2\sqrt{I(R)I(-R)}}{I(R) + I(-R)}|g^{(1)}(R,\tau)| \tag{29}$$

where the factor $2(\sqrt{I(R)I(-R)})/(I(R) + I(-R))$ accounts for the difference between the intensity in each arm of the interferometer, which is approximately equal due to the nominal 50:50

beamsplitter used.

The visibility is extracted by Fourier filtering of the interferogram I_{tot} of its 2D Fourier transform in Supplementary Figure S13 a,b. By masking a circle in the center and reverse-Fourier transforming back to real-space (Supplementary Figure S13c,d), the intensity of the lower envelope and the unmodulated background component can be extracted as I_{BG}. By masking a circle at the first-diffracted order and reverse-Fourier transforming back to real-space (Supplementary Figure S13e,f), half the intensity of the modulated component can be extracted as I_{mod}. Dividing the two images, we obtain $V(R,\tau) = |g^{(1)}(R,\tau)| = 2I_{mod}/I_{BG}$.

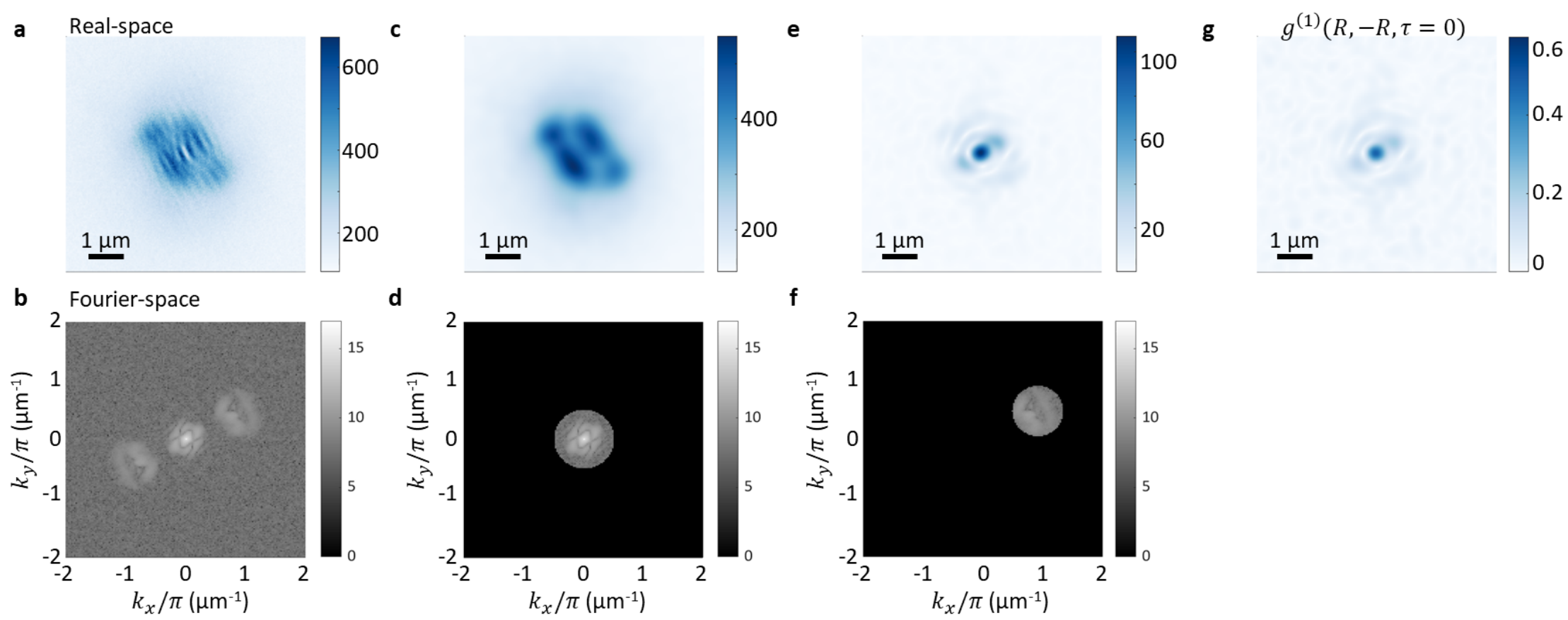

Supplementary Figure S13: **Analysis of Michelson interferogram and extraction of first order spatial coherence of emitted light** $g^{(1)}(R,-R)$**. a,** Real-space interferogram of emission from pumped 1 MB:CB sample (in counts/s). **b,** 2D Fourier-transform of image in a. Filtered data representing the un-modulated incoherent emission component: **c,** Real-space image of filtered image in **d,** at the centre of the Fourier domain. Filtered data representing the modulated coherent emission component: **e,** Real-space image at the first-order displaced mode filtered at **f,**. Both filter radii adjusted to capture only the centre and modulated components. **g,** $g^{(1)}(R,-R)$ map obtained by dividing the images in e and c: 2e/c. Pump laser intensity of 200 $\mu W/\mu m^{-2}$.

As further control experiments, we test the coherence from 2D materials inside and outside of plasmonic nanocavity systems, and compare them to the coherence from the coupled nanocavity system and the pump laser.

This is intended as a control for the coherence expected for a single point scatterer/point source at the focus of the imaging setup. Supplementary Figure S14a and b show the emission interferogram at zero time delay from a 2D layer of WSe_2 on gold and a nanodecahedra-on-mirror nanocavity containing WSe_2 (fabricated as in Ref. [24]). Supplementary Figure S14c shows line profile across the centre of both interferograms showing the limited visibility compared to the synchronized exciton state in the 1 MB:CB sample and the pump laser (Supplementary Figure S14d). Similarly, the directly extracted visibility can be compared to the emission from a broadband white light reflecting off a mirror and that from a 0.01 MB:CB synchronized exciton sample in Supplementary Figure S15.

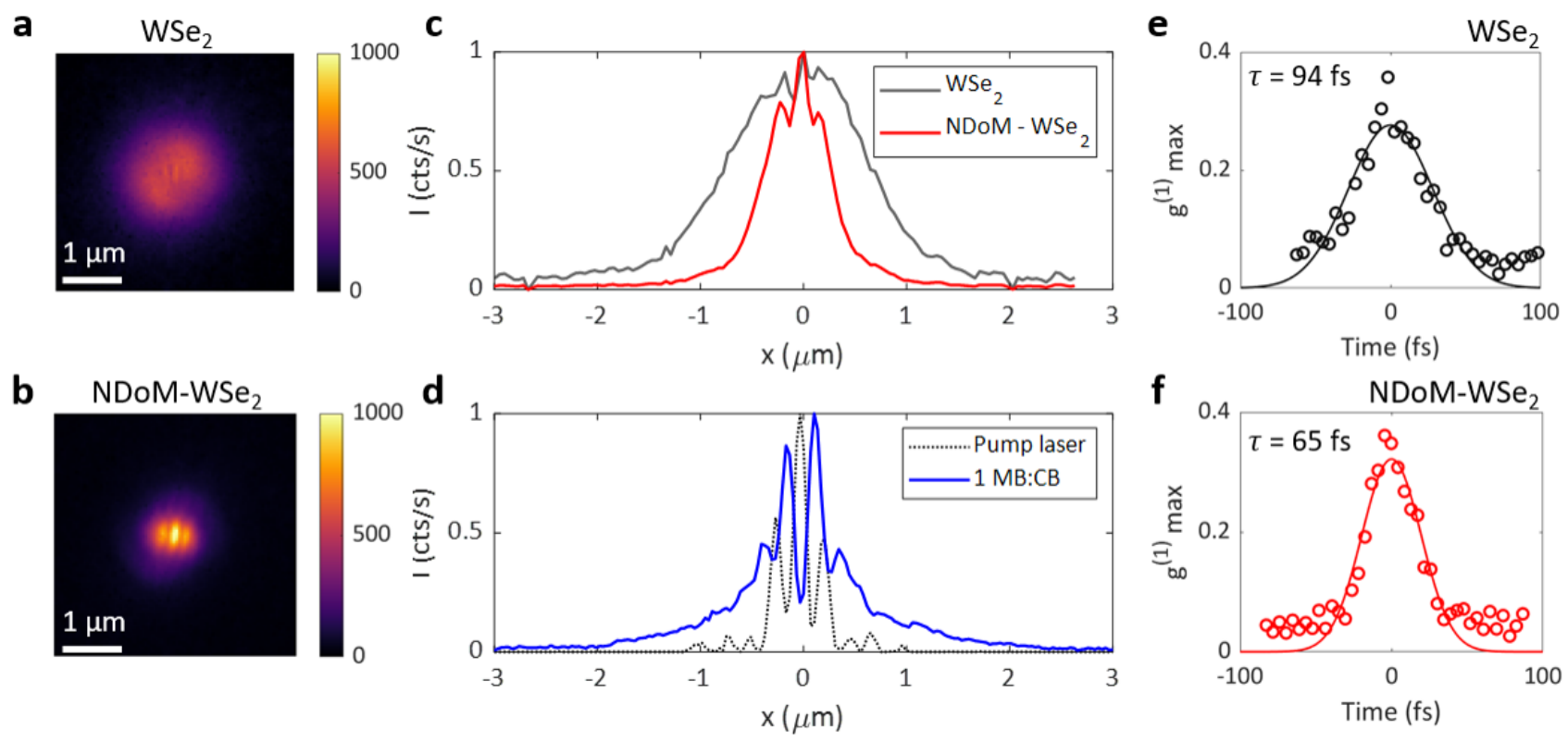

Supplementary Figure S14: **Control Michelson interferograms (in counts/s) from 2D material emitters and single-nanogap systems pumped with a 633 nm laser at 200 $\mu W/\mu m^{-2}$ intensity. a,** Interferogram of emission from WSe_2 monolayer on gold substrate at zero time delay. **b,** Interferogram of emission from WSe_2 monolayer inside a nanodecahedron-on-mirror (NDoM) nanocavity at zero time delay. **c,** Normalipatial line-cut along the centre of the emission pattern of the WSe_2 monolayer and WSe_2 monolayer inside NDoM. **d,** Line-cut along centre of emission from 1 MB:CB synchronized emitters and the pump laser.

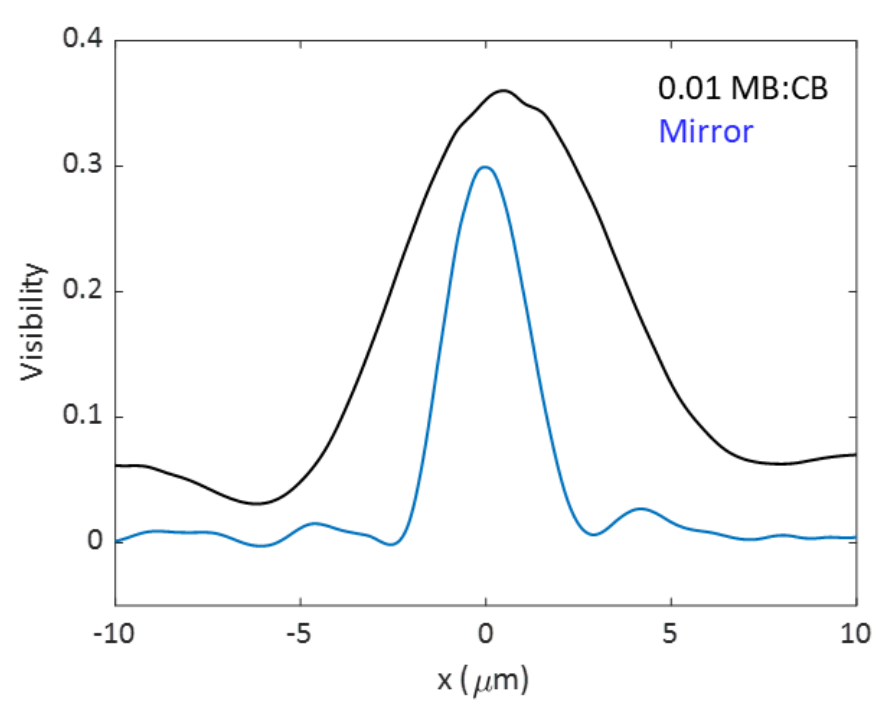

Supplementary Figure S15: **Control Michelson** $g^{(1)}(R, -R, \tau = 0)$ from broadband white light reflecting of a mirror and 0.01 MB:CB synchronized exciton sample. Pump laser intensity of 100 $\mu W/\mu m^{-2}$.

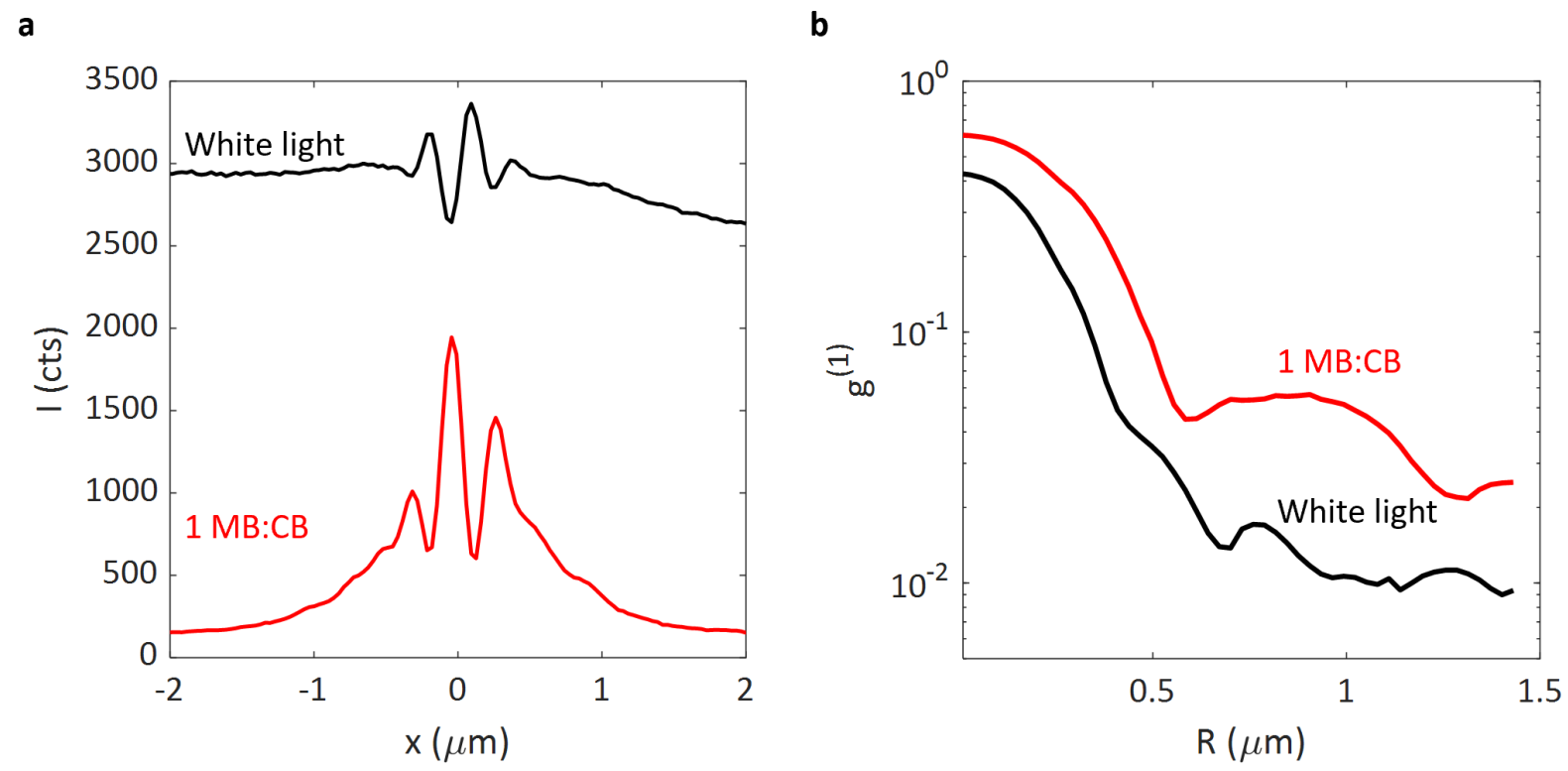

Supplementary Figure S16: **Interferograms for white light reflection off a mirror and MB:CB=1. a,** Interferogram for white light reflecting of a mirror, and 1 MB:CB at pump power of 357 $\mu W \mu m^{-2}$ and **b,** Extracted $g^{(1)}(R, \tau = 0)$ for the corresponding interferograms in a.

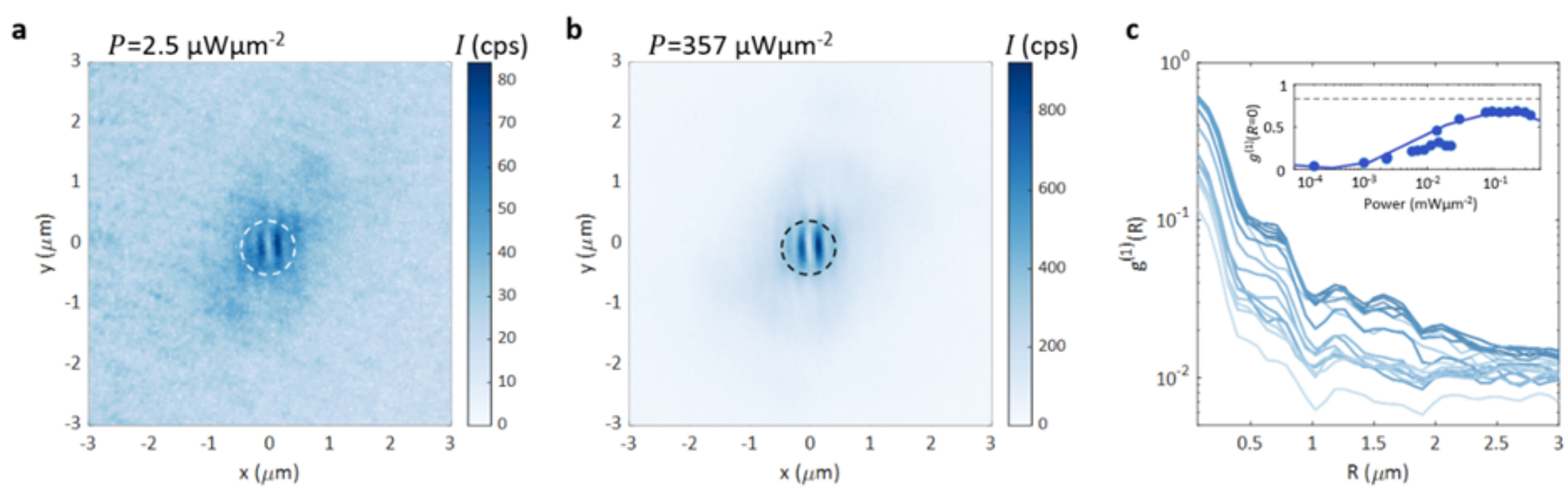

Supplementary Figure S17: **Interferograms at low and high $g^{(1)}$ coherence for MB:CB=1. a,** Interferogram at low pump power of 2.5 $\mu W \mu m^{-2}$ and **b,** at high pump power of 357 $\mu W \mu m^{-2}$. c, Extracted $g^{(1)}(R, \tau = 0)$ for pump powers indicated in the inset reproduced from main text Fig. 2d.

By stepping across the 1 MB:CB sample, we can explore the spatial homogeneity of the interferogram. Supplementary Figure S18 shows that the interference pattern is consistently spread out across the sample, over a range of 12 μm, and comparable in intensity. We highlight the repeatability of the phenomenon and its persistence despite the self-assembled and locally-ordered nature of the nanogap arrays. The $g^{(1)}(R = 0, \tau = 0)$ is plotted against pump power across 8 different locations on the sample in the new Supplementary Figure S19 below, showing similar values to that shown in the main text Fig. 2d. The $g^{(1)}(R, \tau = 0)$ images are also shown across the film at 8 different locations in Supplementary Figure S20, at the corresponding maximum power shown in Supplementary Figure S19.

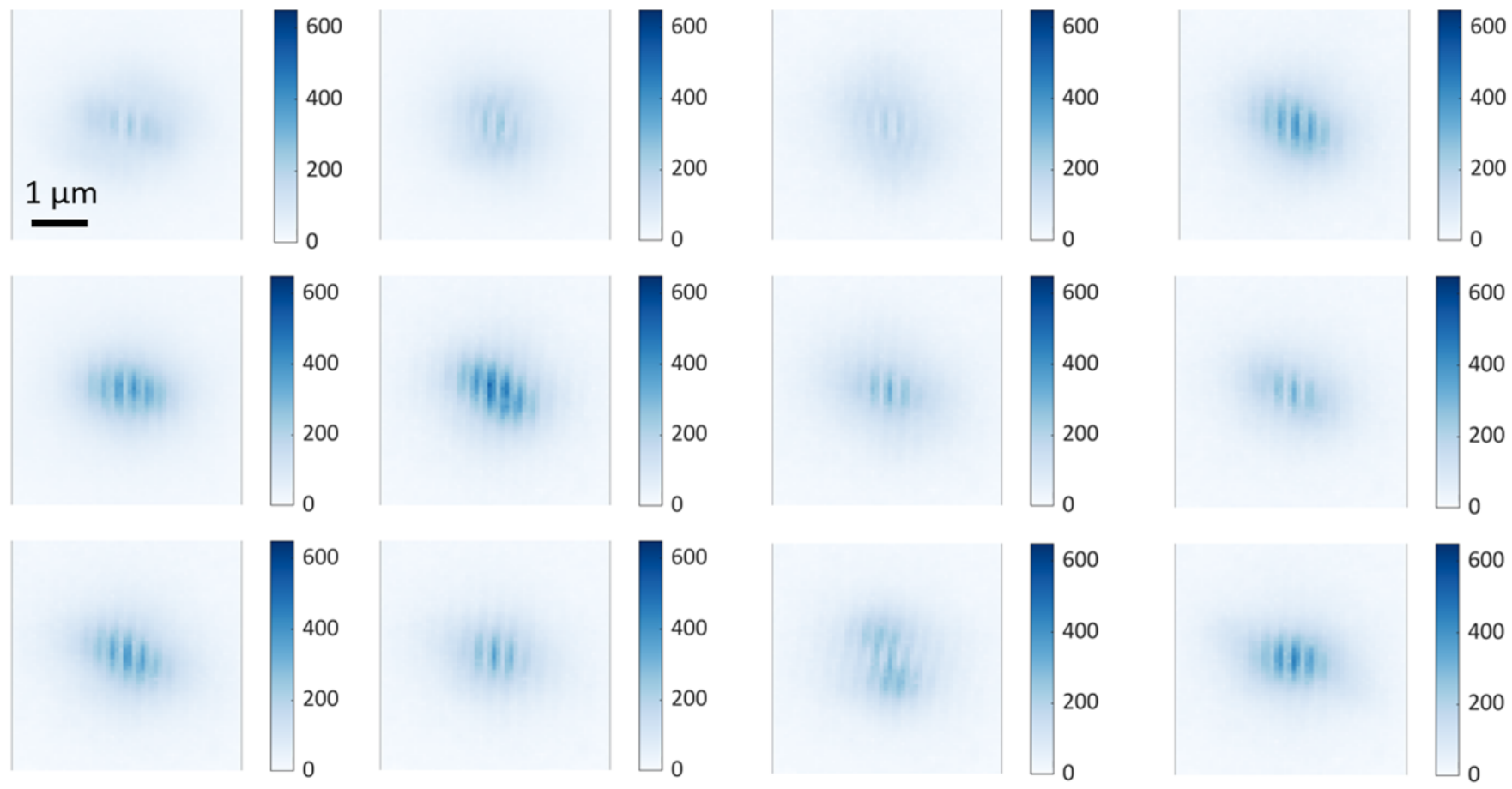

Supplementary Figure S18: **Spatial consistency of Michelson interferogram for 1 MB:CB sample** by translating the sample across laterally by 1 μm and acquiring an interferogram every subsequent image from left to right and top to bottom. Interferogram intensity in counts/s. Pump laser intensity of 200 $\mu W/\mu m^{-2}$.

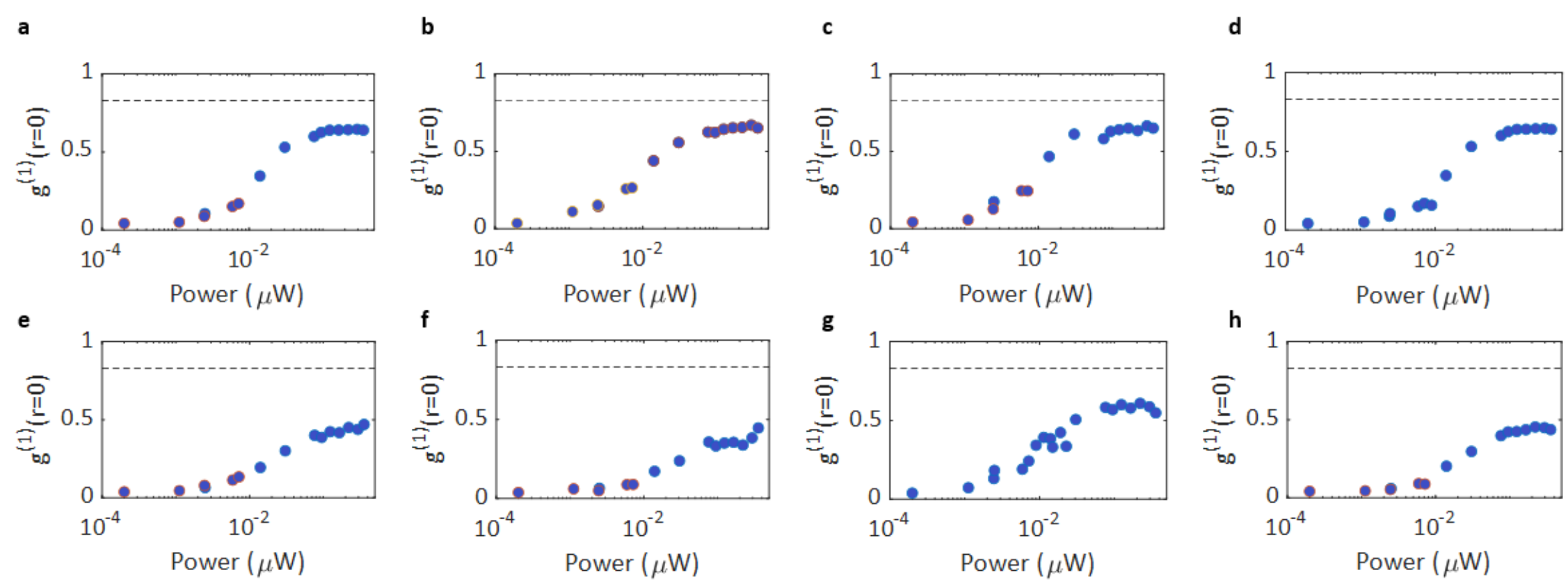

Supplementary Figure S19: **Pump power dependence of** $g^{(1)}(R=0,\tau=0)$ **for MB:CB=1 across different positions. a-h**

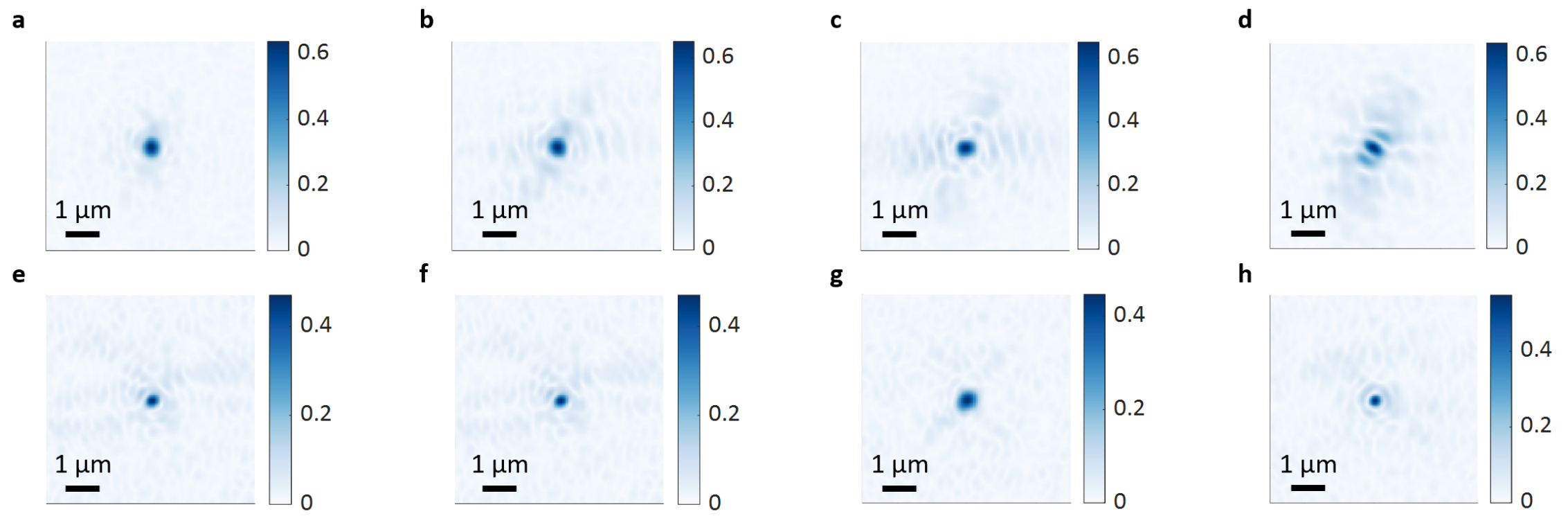

Supplementary Figure S20: **Extracted $g^{(1)}$ for MB:CB=1 at different positions. a-h** at pump power of 357 $\mu W \mu m^{-2}$.

The initial rapid decay of $g^{(1)}(R = 0, \tau)$ reflects the ultrafast radiative decay associated with strong Purcell enhancement. The subsequent collapse-revival-like oscillation arises primarily from spectral beating between distinct components of the emission spectrum. As shown in Supplementary Figure S21, fitting the emission with a broad bi-Gaussian model representing the main electronic transition and vibronic shoulder does not reproduce the observed oscillatory structure in $g^{(1)}(R = 0, \tau)$ when Fourier transformed (Supplementary Figure S21d, black solid line). Only when enhanced resonance-Raman features are included in the spectral model does the Fourier transform yield an oscillatory behaviour consistent with the measured secondary rise (black dotted line). The Fourier transform of the full measured spectrum (blue dotted line) reproduces some features of the observed revival amplitude and period.

However, spectral beating alone does not account for all features, particularly the spatial vortex nucleation observed near the first minimum of $g^{(1)}$. As shown in Supplementary Section S7, phase mapping reveals the emergence of phase winding at this delay. We therefore interpret the collapse-revival feature as arising from two coupled effects: (i) temporal beating between spectral components and (ii) transient reconfiguration of spatial phase domains associated with vortex formation.

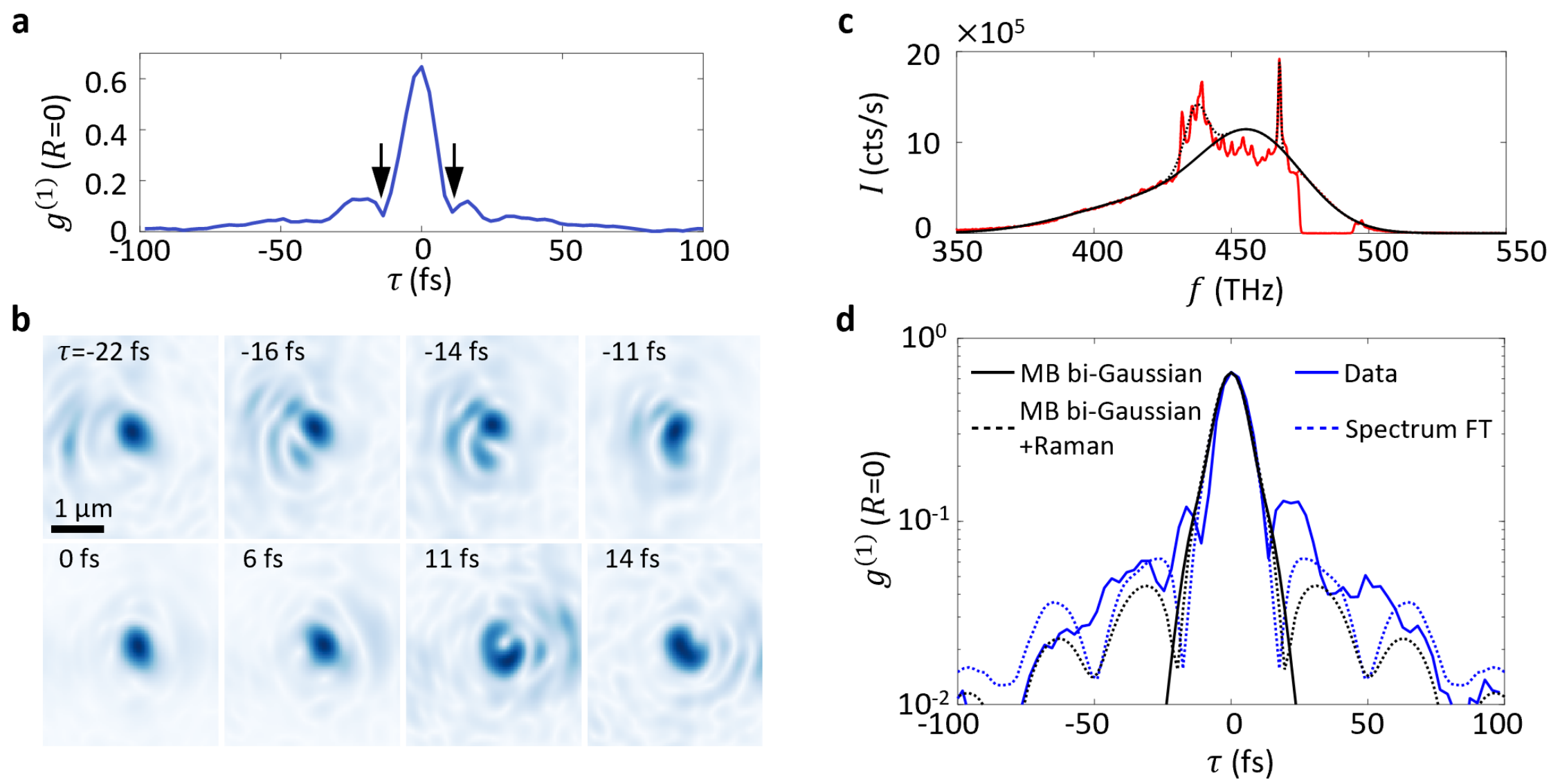

Supplementary Figure S21: **Collapse-revival analysis in $g^{(1)}$. a,** Decay of $g^{(1)}(R = 0, \tau)$ for MB:CB = 1 at pump power P = 0.2 mW, with arrows indicating formation of vortices degrading the central $g^{(1)}$ at labelled interferometer time delay τ. **b,** Vortex phase dislocation in the $g^{(1)}(x, y, \tau)$ in space and time, corresponding to zero observed in $g^{(1)}(R = 0, \tau)$ coherence trace in **a**. All images share the same scale bar. **c,** Emission spectrum of MB:CB = 1 corresponding to **a**, with solid black lines showing bi-Gaussian fit for MB main emission peak and vibronic side-band. Narrower Gaussian peaks show surface-enhanced resonant Raman peaks of MB. **d,** Decay of $g^{(1)}(R = 0, \tau)$ compared to Fourier-transform of the fitted MB emission peak (MB bi-Gaussian), the fitted MB emission and resonance Raman (MB bi-Gaussian + Raman), the entire spectrum in c (Spectrum FT), and the observed data in a (Data).

SI Section S5. Power dependence and input power scaling

In order to correct for the effect of slight differences in local nanoparticle configuration affecting the amount of pump laser intensity actually coupled from the far-field to the emitters in the near-field, a scaling procedure was performed using the method developed in [25]. To allow comparison across positions on a single sample with a certain emitter MB:CB density, we apply a normalization procedure based on their emission at the lowest input power.

For each position i, we define its coupling efficiency η_i by measuring the integrated emission signal $S_i(\omega_{\nu_1}, I_0)$ under the lowest incident power I_0:

$$\eta_i = \frac{S_i(\omega_{\nu_1}, I_0)}{I_0} \tag{30}$$

We then compute the average coupling efficiency across all particles:

$$\bar{\eta} = \frac{1}{N}\sum_{i=1}^{N} \eta_i \tag{31}$$

To correct for differences in in-coupling efficiency, we define the normalized in-coupled intensity $I_{\text{in}}^{(i)}$ for each position on the sample as:

$$I_{\text{in}}^{(i)} = I_\ell \cdot \frac{\eta_i}{\bar{\eta}} \tag{32}$$

Here, I_ℓ is the nominal laser power used during measurement. The units of $I_{\text{in}}^{(i)}$ are integrated counts per second per μW, and this normalization ensures that at the same low laser power, all positions produce the same normalized emission signal.

This normalization procedure is essential for comparing the nonlinear synchronized emission response across an ensemble of nanostructures. Its effectiveness is demonstrated in Supplementary Figure **S22**: without normalization, the emission intensity at fixed laser power varies across different positions. After correction using $I_{\text{in}}^{(i)}$, a consistent power-dependent trend is recovered, enabling quantitative comparison across the dataset and samples with different emitter densities.

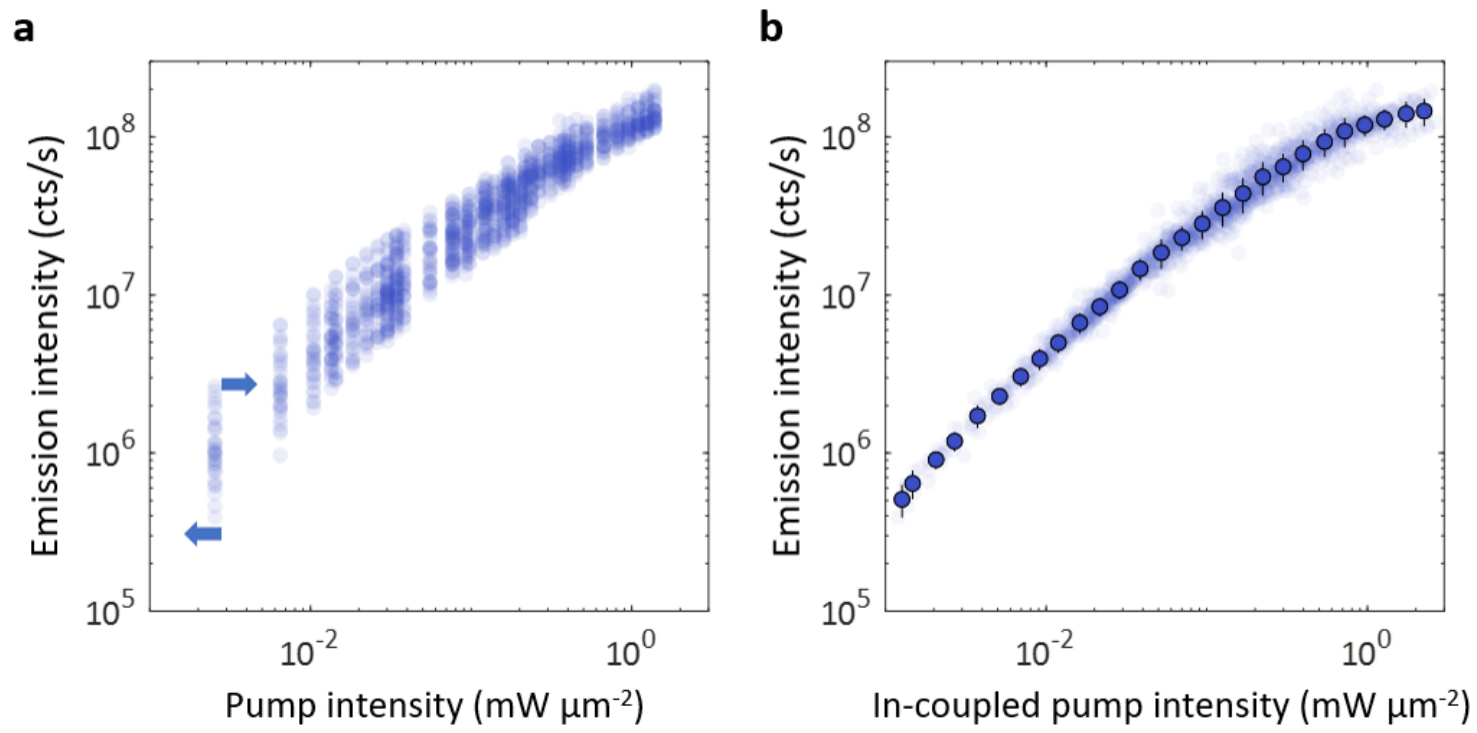

Supplementary Figure S22: **Correction of incident pump power for nanocavity in-coupling. a,** Integrated emission intensity from 1 MB:CB sample across ∼50 positions, pumped by a 633 nm laser. Arrows show direction of correction of in-coupling efficiency by normalizing the laser intensity at each position with the emission intensity at the lowest pump intensity. **b,** Integrated emission intensity from 1 MB:CB sample across ∼50 positions against corrected intensity scaled for the in-coupling efficiency ($I_{\rm in}^{(i)}$). Using method developed in [25]

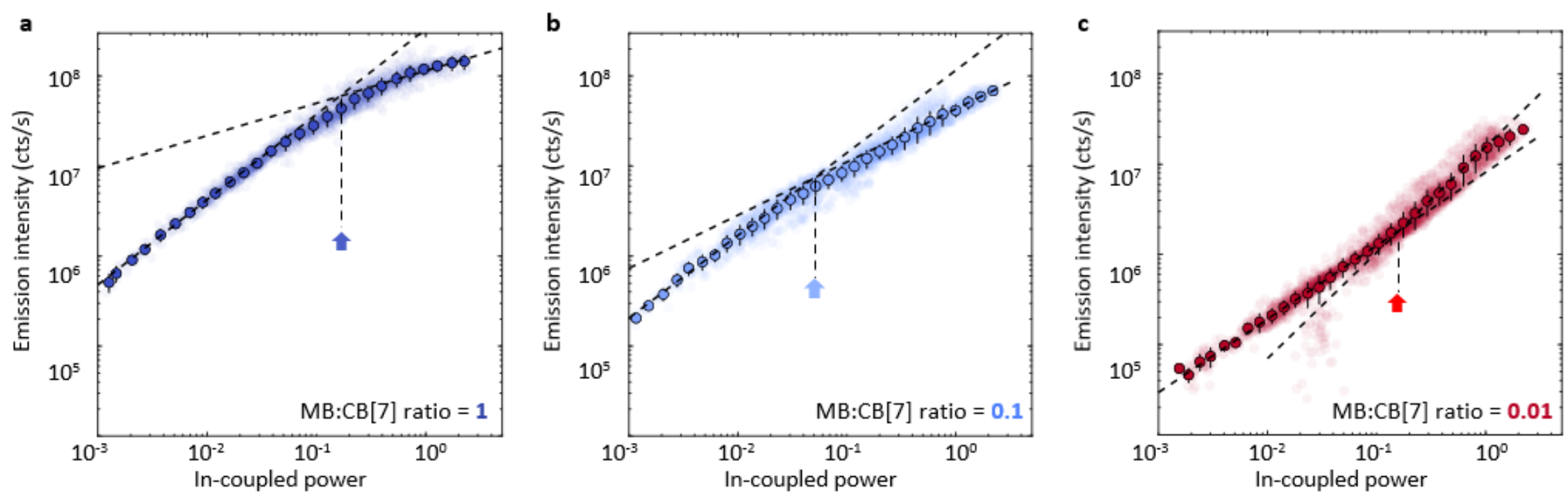

Supplementary Figure S23: **Input-output relation for nanocavity system.** Integrated intensity of MB emission vs in-coupled power for increasing dye concentration ($\propto P^{\alpha}$), showing deviations from linear scaling for different regimes. **a,** MB:CB = 1, dotted lines show a transition from linear (α=0.95±0.02) to sublinear (α=0.36 ±0.1) power dependence. **b,** MB:CB = 0.1, transition from linear (α=0.92±0.1) to sublinear (α=0.59±.05) power dependence. **c,** MB:CB = 0.01, transition from linear (α=0.81±0.1) to superlinear (α=1.2±0.1) power dependence. The cross-over point between the regimes varies from 0.16, 0.05 and 0.03 for MB:CB = 1, 0.1, and 0.01 respectively.

SI Section S6. Surface-enhanced vibronic and Raman spectra

The absorption-emission spectrum of methylene blue (MB) displays a small Stokes shift and hence significant overlap between the absorption and emission profiles (Supplementary Figure S24a). This enables Forster-enhanced resonance energy transfer (FRET) to occur via near-field coupling processes, whose efficiency scales with the overlap integral between the normalized absorption-emission spectrum [26]. The emission spectrum of the 0.01 MB:CB synchronized excitonic state also changes depending on incident pump power, blueshifting and displaying a significant anti-Stokes emission (at energies higher than the pump laser energy) in Supplementary Figure S24b.

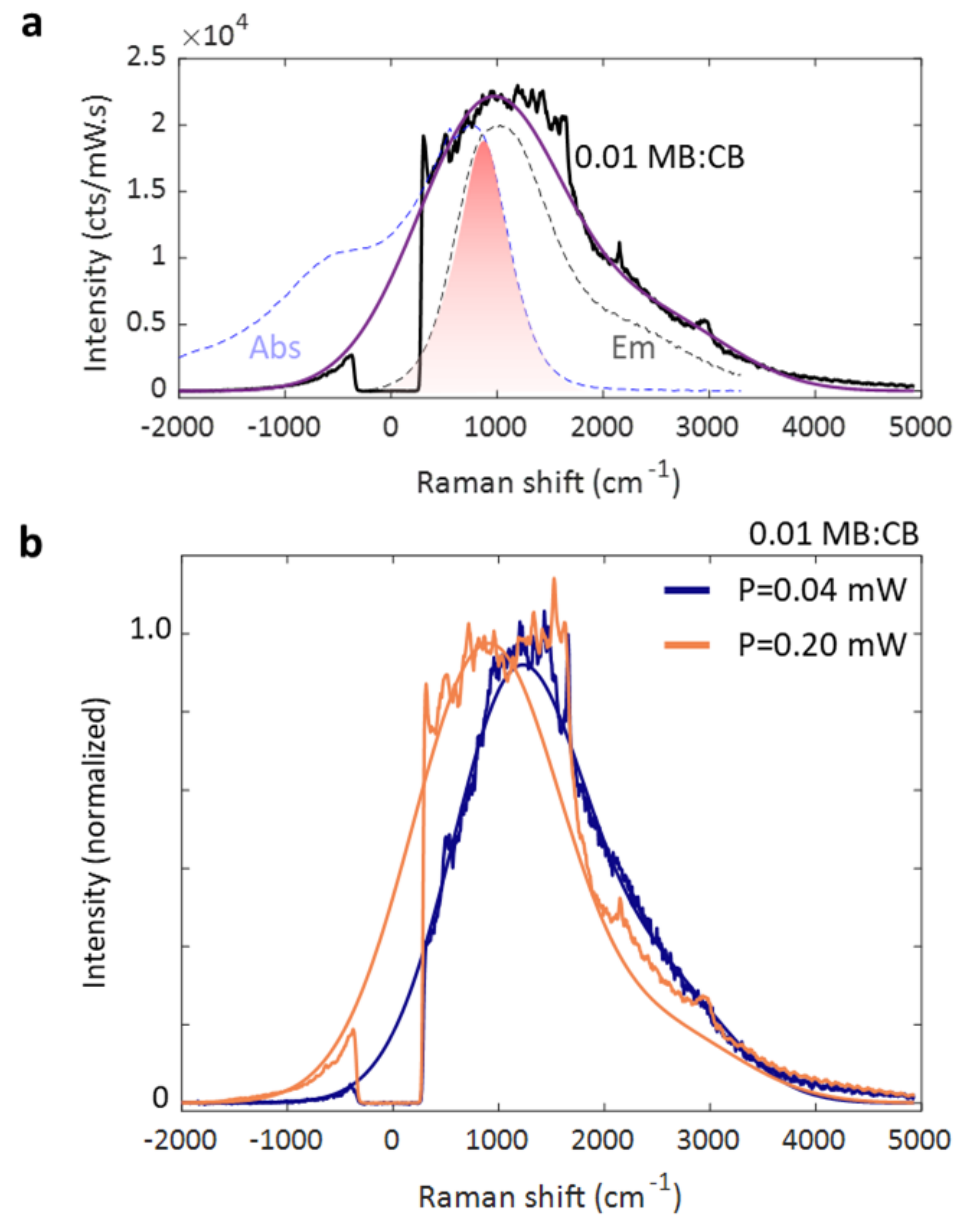

Supplementary Figure S24: **Emission spectral linewidth broadening of synchronized excitons and Forster overlap. a,** Emission spectrum of the 0.01 MB:CB sample overlapped with the normalized absorption and emission spectrum of aqueous methylene blue solution. **b,** Normalized intensity spectrum of 0.01 MB:CB at low pump power (0.04 $mW/\mu m^{-2}$) under linear input-output relation conditions, and above threshold (0.20 $mW/\mu m^{-2}$) at nonlinear input-output conditions.

Supplementary Figure S25 shows the emission spectrum for 0.01 MB:CB samples with the main surface-enhanced resonant Raman peaks (at 530, 1650, 2160, and 2960 cm^{-1}) labelled with arrows. The two vibronic emission transitions of MB are also fitted with Gaussian profiles, corresponding to the emission from the excited electronic state to the ground electronic state (0-0 transition) and a vibronically excited ground state (0-1 transition).

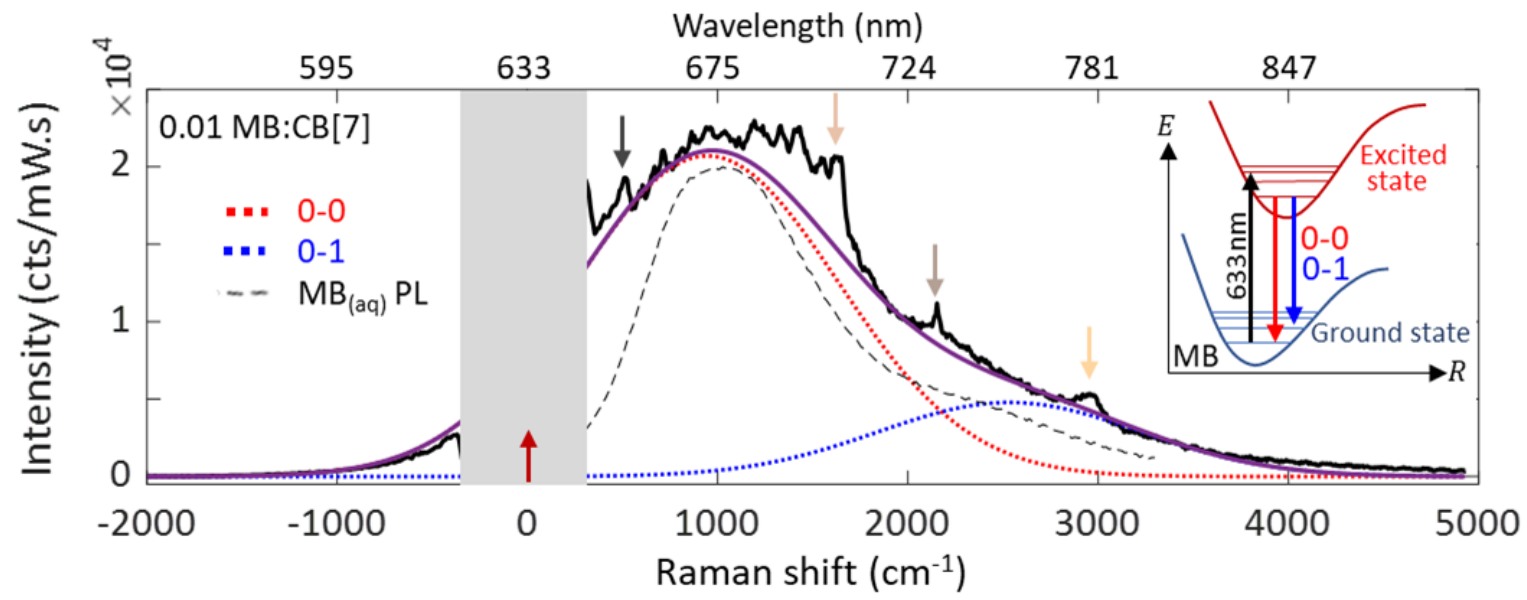

Supplementary Figure S25: **Emission and surface-enhanced resonant Raman spectrum of 0.01 MB:CB.** Emission spectrum of 0.01 MB:CB films, pumped at 633 nm (indicated by arrow at Raman shift of 0 cm^{-1}), overlaid with the emission spectrum of aqueous MB solution ($MB_{(aq)}$), and fitted with two Gaussian peaks. The red and blue Gaussian peak corresponds to the vibronic emission from the excited electronic state to the ground electronic state (0-0 transition) and a vibronically excited ground state (0-1 transition) respectively. Each process is indicated by coloured emission arrows on the Jablonski diagram inset. Coloured arrows indicate Stokes surface-enhanced resonant Raman peaks. Raman shift measured from the pump laser centered at 633 nm.

Supplementary Figure **S26** show the pump power dependence of the intensity of each of the surface-enhanced resonance Raman spectroscopy (SERRS) peaks indicated in Supplementary Figure **S25**a. The background photoluminescence was subtracted prior to analysis to obtain the signal from just the SERRS process. While the 530, 2160, and 2960 cm^{-1} molecular vibrations display the linear dependence expected of SERRS, the 1650 cm^{-1} vibration shows an initial linear power dependence, transitioning to almost a quadratic dependence. The nonlinear Stokes and anti-Stokes SERRS of the 1650 cm^{-1} line is shown in Supplementary Figure **S27**a, with the anti-Stokes also showing a nonlinear power dependence once the signal rises above the noise floor indicated. This may be attributed to vibrations being pumped into the MB molecule via the vibronic emission process, as the energy difference between the (0-0) and (0-1) peaks corresponds to molecular vibrations at 1650 cm^{-1}. The 1650 cm^{-1} vibration is related to a local C=C stretch mode in the core of the MB molecule [27, 28], which is predicted to have strong Franck-Condon overlap and hence a high resonant Raman cross-section as well [26]. This vibrational pumping process mediated by photoluminescent emission is depicted in Supplementary Figure **S27**b.

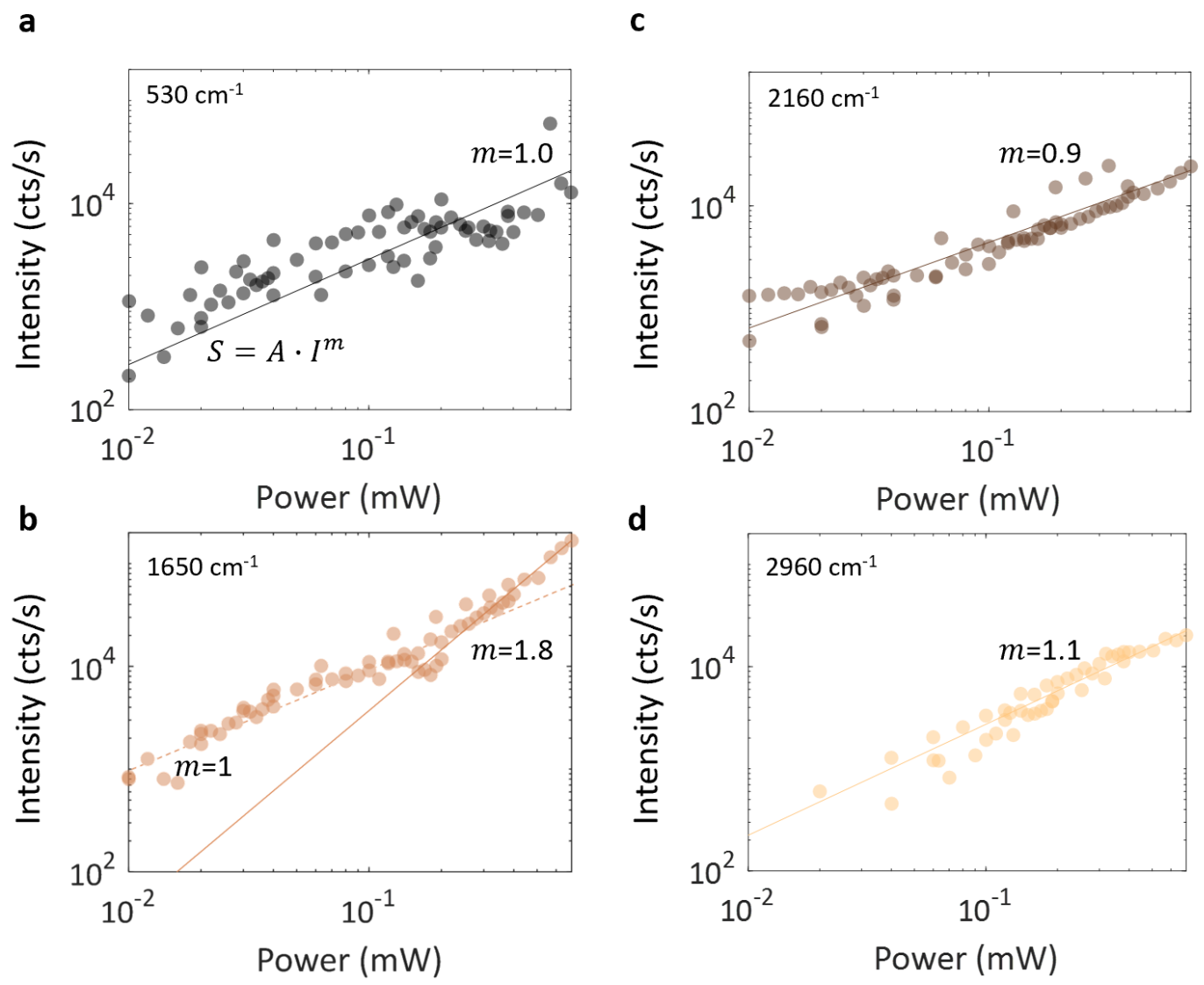

Supplementary Figure S26: **Pump power dependence of Raman vibrational transitions of 0.01 MB:CB.** Power law $S = A \cdot I^m$ with exponent m fitted for Raman vibrations of frequency: **a,** 530 cm^{-1}, **b,** 1650 cm^{-1}, **a,** 2160 cm^{-1}, and **a,** 2960 cm^{-1}, where S is the integrated intensity of the Raman peak, A is a proportionality constant, and I is the pump laser power. Each Raman transition indicated by an arrow in Figure S24a

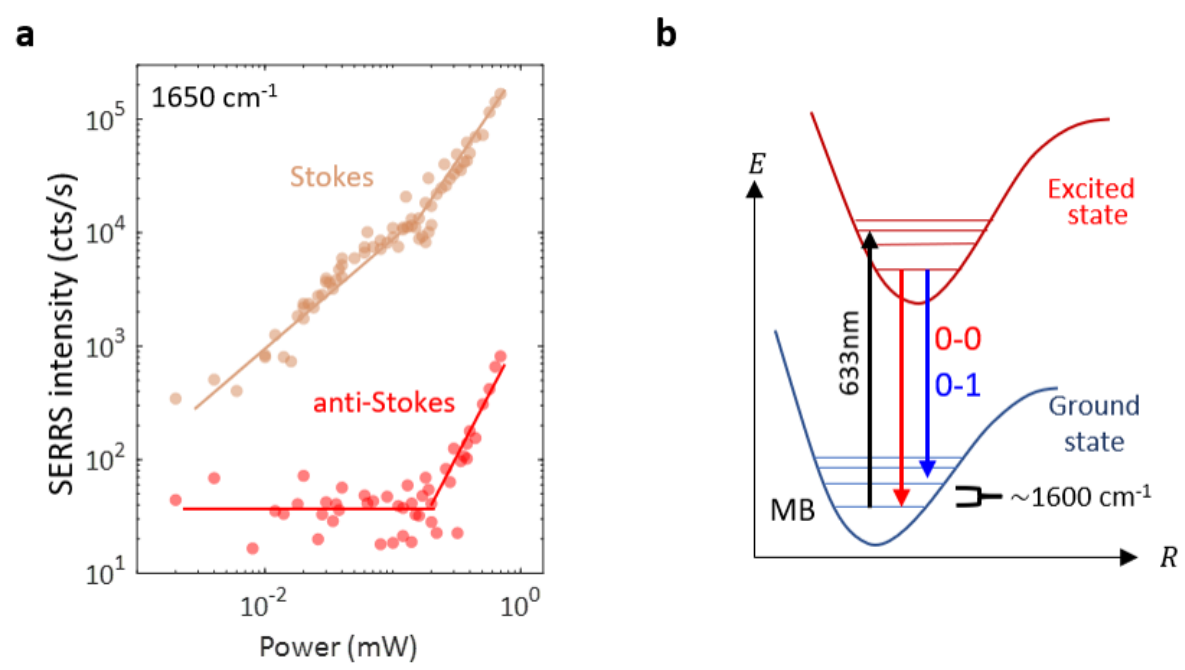

Supplementary Figure S27: **Nonlinear Raman of the vibronic line of 0.01 MB:CB. a,** Stokes and anti-Stokes power dependence of the 1650 cm^{-1} transition. Noise floor indicated by the horizontal red line for the anti-Stokes data. **b,** Jablonski diagram of the ground and excited state of MB emitters, showing the role of the vibronic photoluminescent transitions and the main vibration at 1650 cm^{-1} involved, pumped by the photoluminescence process.

The plasmonic nanogap array speeds up radiative emission, thus the excited state only exists for a short time, preventing bond disassociation and photochemical bleaching or transfer to dark

triplet states and hence reaction with singlet oxygen. This has also been shown by other groups in recent work [29–31]. Heating may be an issue with CW measurements, however we use anti-Stokes electronic Raman scattering (ERS) thermometry, as in Ref. [32, 33], to show that the system is not significantly heating, in Supplementary Figure S28 below. The anti-Stokes ERS spectrum (I_{ERS}) is fitted with a Boltzmann model $I_{ERS} \propto \exp(-\Delta E/k_B T)$ to extract out an effective temperature. Strictly speaking, it is the temperature of the electrons in the plasmonic nanogap array; however, we assume equilibrium between such temperatures and the temperature of the molecules hosted within the nanogap.

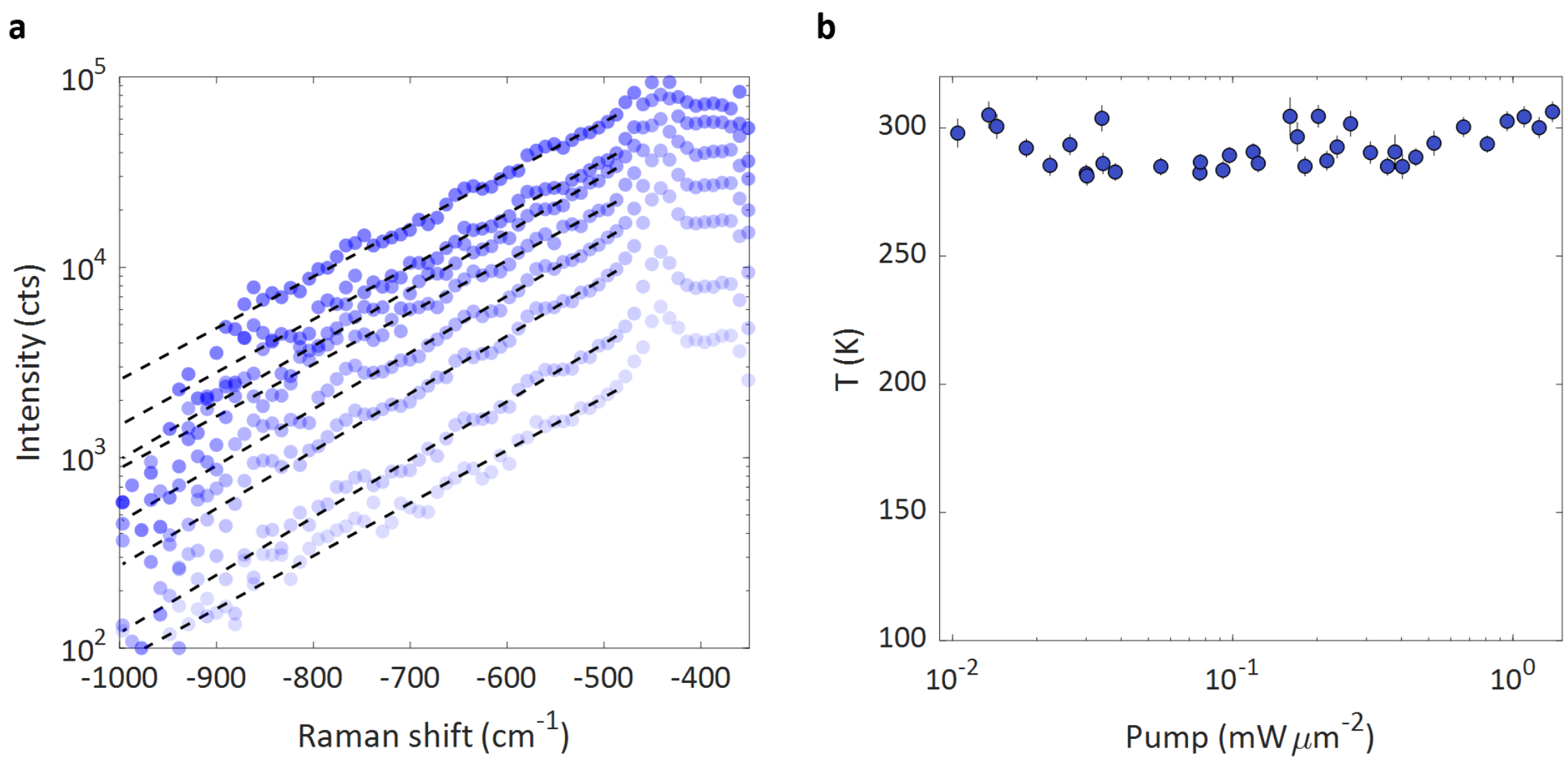

Supplementary Figure S28: **Anti-Stokes Electronic Raman scattering measurements. a,** Anti-Stokes electronic Raman scattering spectrum from MB:CB=1 over a range of powers from 0.01 to 2 mWμm^{-2}, dotted lines shows fits to Boltzmann tail extracting temperature. **b,** Extracted anti-Stokes electronic temperature vs pump power.

SI Section S7. Polariton vortex simulations and experiments

Simulation of the polariton interferograms using Eq. (28) enables us to identify the presence of vortices in the interferogram. If we represent the synchronized exciton/polariton condensate emission's complex electric field as:

$$E(R,\theta) = A(R) \cdot e^{i\Phi(R,\theta)} \tag{33}$$

where $A(R) = \exp\left(-R^2/w_0^2\right)$ is the amplitude of the condensate (Supplementary Figure **S29**a), with w_0 being the Gaussian beam waist and $R = \sqrt{x^2+y^2}$ being the radial coordinate. $\Phi(R,\theta) = m.\theta$ is the phase of the condensate (Supplementary Figure **S29**b), which contains a topological charge or vortex winding number m and can be located at angular position $\theta = \arctan\left(\frac{y-y_0}{x-x_0}\right)$ offset from the centre by Cartesian coordinates x_0, y_0. To obtain the interferogram, the electric field in the plane mirror arm is summed with the electric field in the retroreflector arm, which is spatially inverted as $E(R) \to E(-R)$ or $E(x,y) \to E(-x,-y)$, with an additional phase to account for the finite angle ϕ between the two beams and also the path length difference ΔL between the two arms in units of the central emitted wavelength λ. We assume that there is only a tilt in the x direction, thus interference in that direction only.

$$I_{tot}(R,\theta) = |E_1(R,\theta) + E_2(-R,\theta) \cdot e^{-i(k_x x)} \cdot e^{-i(\frac{2\pi\Delta L}{\lambda})}|^2 \tag{34}$$

Matching the experimental measurements requires the introduction of two new physical parameters, a finite coherence time in the field and a finite coherence length. The polychromatic field is represented by a Gaussian spectral distribution centered at angular frequency ω_0 and spectral width $\Delta\omega$. The final field is then a coherent summation across frequencies:

$$E_{\text{total}}(\mathbf{r}) = \int d\omega\, S(\omega) \left[E_1(R) + E_2(-R) \cdot e^{-i(k_x x)} \cdot e^{i\omega\Delta\tau}\right] \tag{35}$$

where $S(\omega) = \exp\left(-\frac{(\omega-\omega_0)^2}{2\Delta\omega^2}\right)$ is the spectral weight and $\Delta\tau = \Delta L/c$ is the delay induced by a path length difference ΔL. Visibility is reduced for large path length offsets, $\Delta L \gg L_{\text{coh}}$. The final intensity pattern is

$$I(x,y) = |E_{\text{total}}(x,y)|^2 \tag{36}$$

The resulting interference pattern faithfully reproduces the experimentally measured vortex interferograms in the main text (Supplementary Figure **S29**) and also shows a clear finite temporal coherence time ($\tau_c = 1/\Delta\omega$) in (Supplementary Figure **S30**). The decrease in fringe visibility can be compared to experimental measurements in the inset of Supplementary Figure **S30**c.

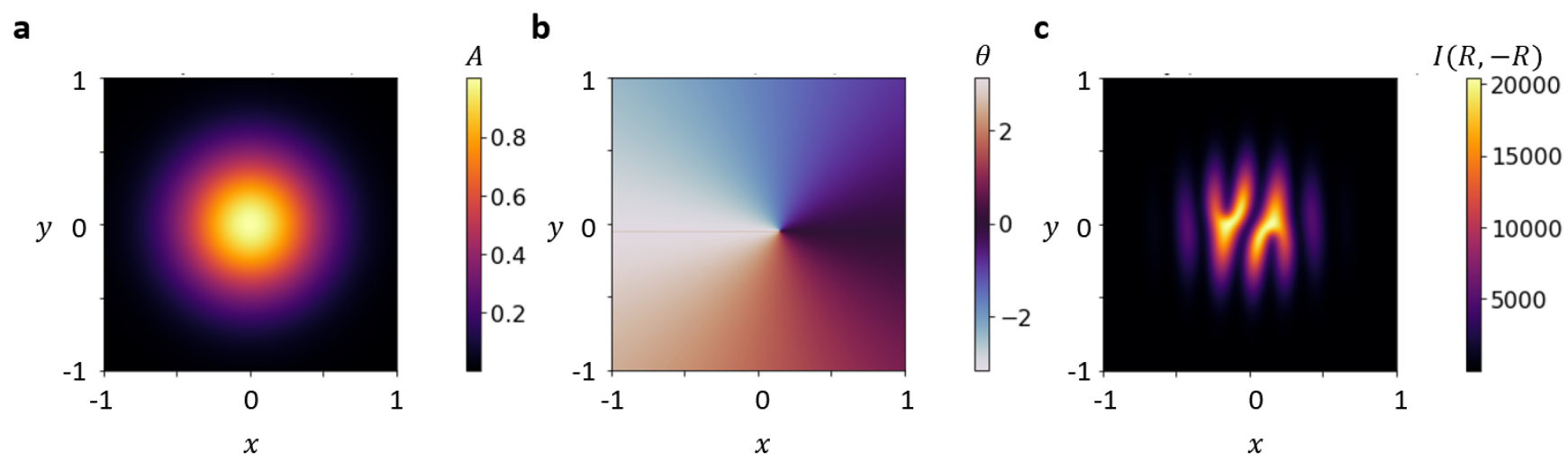

Supplementary Figure S29: **Simulated polariton interferogram with a single vortex offset from the centre. a,** Amplitude of complex condensate (normalized). **b,** Phase of complex state in radians. **c,** Intensity interferogram of the complex condensate overlapping R with $-R$.

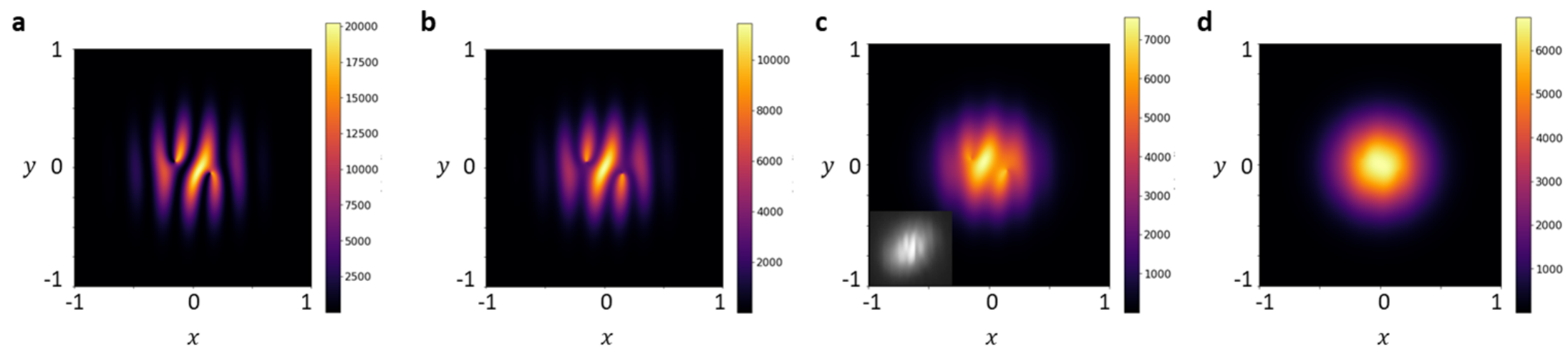

Supplementary Figure S30: **Simulated polariton vortex interferograms with a single vortex at different Michelson delay times/path-length differences. a,** $\Delta L = 1/4(2\pi/\tau_c)$. **b,** $\Delta L = 1/2(2\pi/\tau_c)$. **c,** $\Delta L = 3/4(2\pi/\tau_c)$ (inset is an experimental interferogram at close to time-zero). **d,** $\Delta L = (2\pi/\tau_c)$. Where ΔL is the path length difference and τ_c is the coherence time of the emission. Parameters in units of $\omega_0 = 1$ and $\Delta\omega = \omega_0/2$. Color bars indicate the simulated intensity.

S7.1 Vortex and phase gradient (compressible and incompressible) components

The vortex formed in main text Figure 5 is not a vortex traditionally observed in a condensate, but is a vortex in the $g^{(1)}(R, -R, \tau)$ coherence, which probes the emission coherence at point R with the point $-R$, for a continuous-wave excited emission at relative time delays of the field τ. This means a vortex is showing a phase slip of the field when correlations are probed with a delay time τ, but with averaging over the observation times of the continuously emitting system.

The phase of this vortex can be retrieved from the Fourier transform itself by subtracting the d.c. component in the Supplementary Figure S31, as performed in Ref. [34]. While Mach-Zehnder interferometry and interferometry with a reference phase from an expanded homogeneous part of the emission has been used [21], this is difficult in our system due to the inherent fluctuation of the plasmonic system and the inhomogeneous emission pattern (Fig. 1f, main text). Hence, expansion of a region of the emissive region will not guaranty a flat phase for interference.

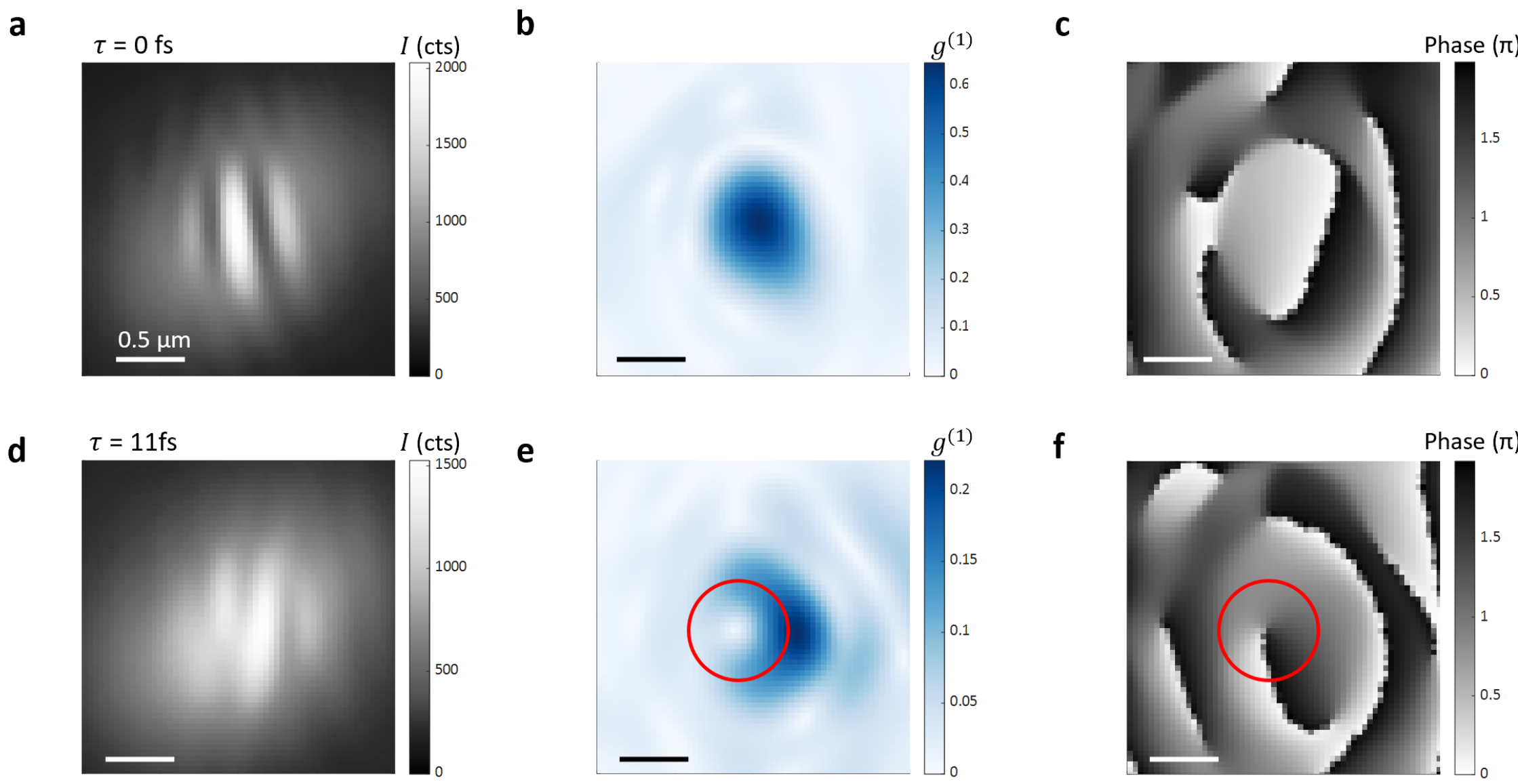

Supplementary Figure S31: **Phase retrieval for vortex formation in $g^{(1)}(R, -R, \tau)$ for MB:CB=1.** At zero time delay $\tau = 0$ fs: **a,** Bare interferogram. **b,** Extracted $g^{(1)}(R, -R, \tau)$ using techniques in Supplementary Figure S13. **c,** Extracted phase profile showing flat phase in the centre of the emission region. At time delay $\tau = 11$ fs: **d,** Bare interferogram showing phase fork. **e,** Extracted $g^{(1)}(R, -R, \tau)$ showing vortex with null $g^{(1)}$ coherence circled red. **f,** Extracted phase profile showing vortex with phase winding around 2π.

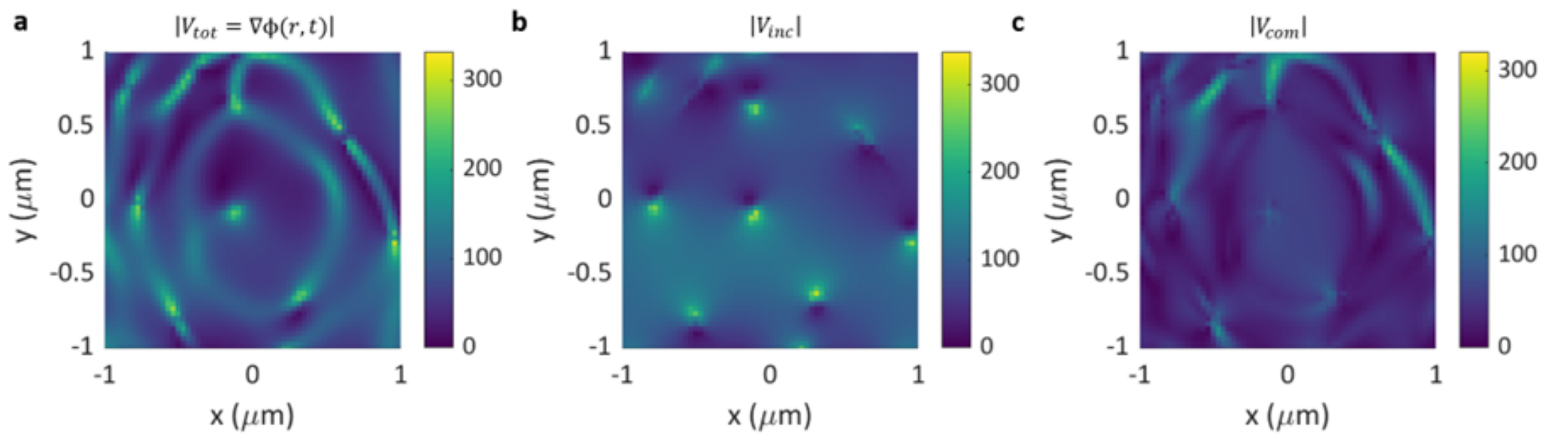

Supplementary Figure S32: **Flow analysis of phase velocity field.** At time delay $\tau = 11$ fs: **a,** Magnitude of the total phase velocity ($|v_{tot}|$) extracted from Supplementary Figure **S31**f and its decomposition into the **b,** incompressible field ($|v_{inc}|$) and **c**, compressible field ($|v_{com}|$) components.

Following the hydrodynamic description of quantum fluids [34, 35], the velocity field is defined as the gradient of the phase of the macroscopic wavefunction, $\boldsymbol{v}_{tot}\,(R,\tau) = \frac{\hbar}{m}\,\nabla\phi(R,\tau)$. To ensure numerical stability and correct handling of topological phase singularities (2π wraps) present in polariton vortices, the velocity was calculated using the complex form $\boldsymbol{v}_{tot} = \text{Im}\left\{\psi^*\nabla\psi\right\}/\left|\psi\right|^2$, which preserves the rotational nature of the flow at the vortex cores, depicted below in Supplementary Figure **S32**a.

The velocity field can be decomposed into an incompressible/solenoidal/divergence-free ($\boldsymbol{v}_{inc}$) and compressible/longitudinal/curl-free ($\boldsymbol{v}_{com}$) field. These fields are extracted with a conventional approach, first by applying the 2D-Fourier transform (2D-FFT) onto $\boldsymbol{v}\,(\boldsymbol{R},\tau)$ to form $\boldsymbol{v}\,(\boldsymbol{k},\tau)$. As the 2D-FFT is periodic, we mitigate edge/finite boundary effects by subtracting the mean/bulk average velocity and applying a 2D Hann-window to smooth the velocity to zero at the boundaries.

Subsequently, the incompressible field can be defined by projecting the Fourier coefficient into the direction perpendicular to the wavevector $\boldsymbol{k} = (k_x, k_y)$:

$$\boldsymbol{v}_{com} = \frac{\boldsymbol{k}(\boldsymbol{k}\cdot\boldsymbol{v}_{tot})}{\left|k\right|^2} \tag{37}$$

$$\boldsymbol{v}_{inc} = \boldsymbol{v}_{tot} - \boldsymbol{v}_{com} \tag{38}$$

The fields are then transformed back into real-space with the inverse 2D-FFT and de-windowing. The final output shows the solenoidal field showing swirls or incompressible flow and vortices in Supplementary Figure **S32**b, and the longitudinal field showing flow from sources or sinks in Supplementary Figure **S32**c. It is evident that there is a bona-fide vortex close to the centre of the image, and additional vortices initially not observed in the interferogram, which may be due to low signal-to-noise.

We also show additional interferogram images below in Supplementary Figures **S33** to **S35** with vortices forming and traversing the system, with corresponding space-time coherence plots to show variation across the sample plane in the origin point and propagation of these vortices.

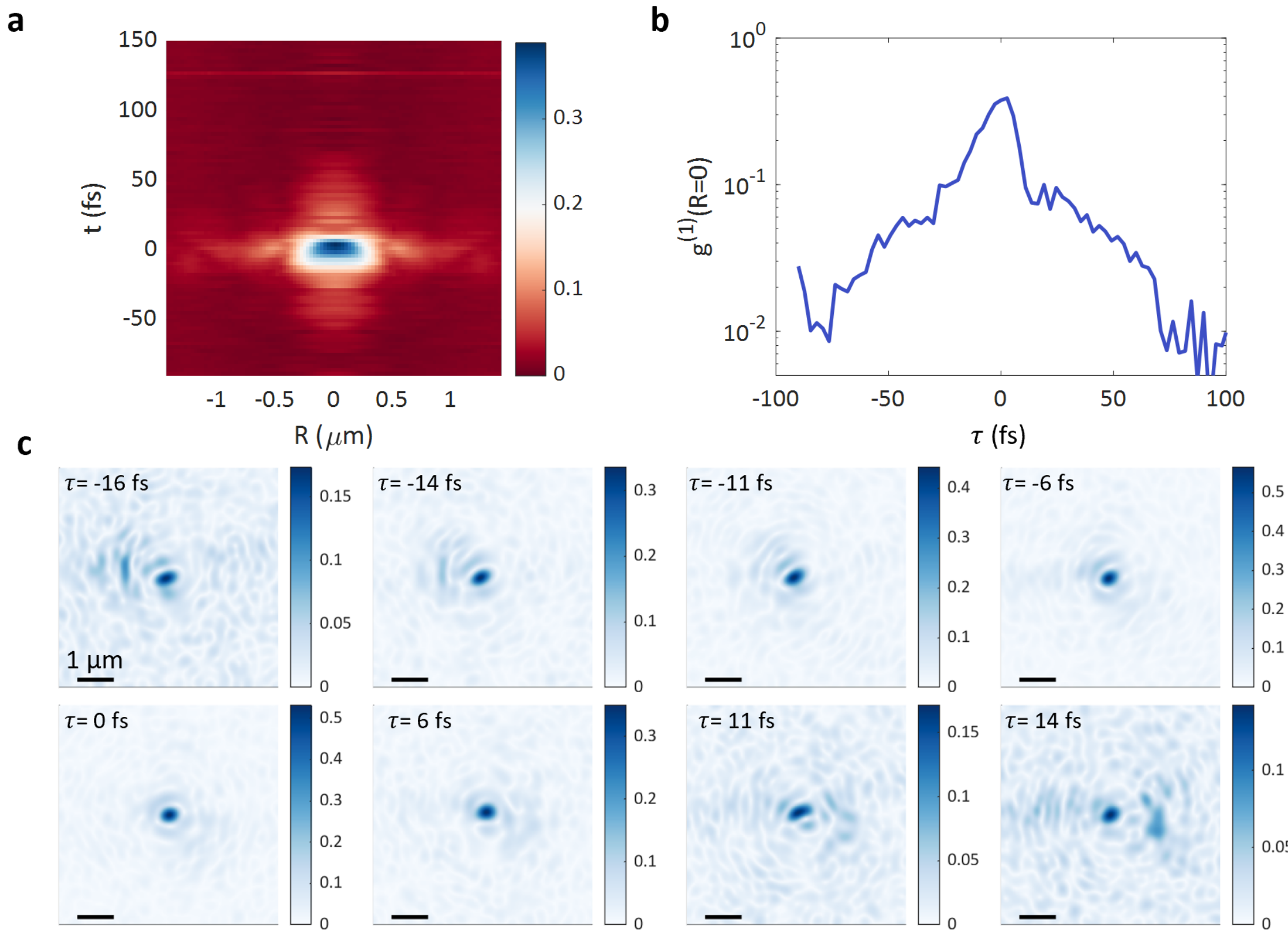

Supplementary Figure S33: **Vortex interferograms and analysis for MB:CB=1. a,** Spatio-temporal $g^{(1)}(R,\tau)$ decay for MB:CB $=1$. **b,** Extracted time decay at the centre of the pumped region $g^{(1)}(R=0,\tau)$ for pump power P=0.2 mW. **c**, Vortex phase dislocation in the $g^{(1)}\,(x,y,\tau)$ in space and time, corresponding to zero observed in $g^{(1)}\,(R=0,\tau)$ coherence trace in (a). All images share the same scale bar.

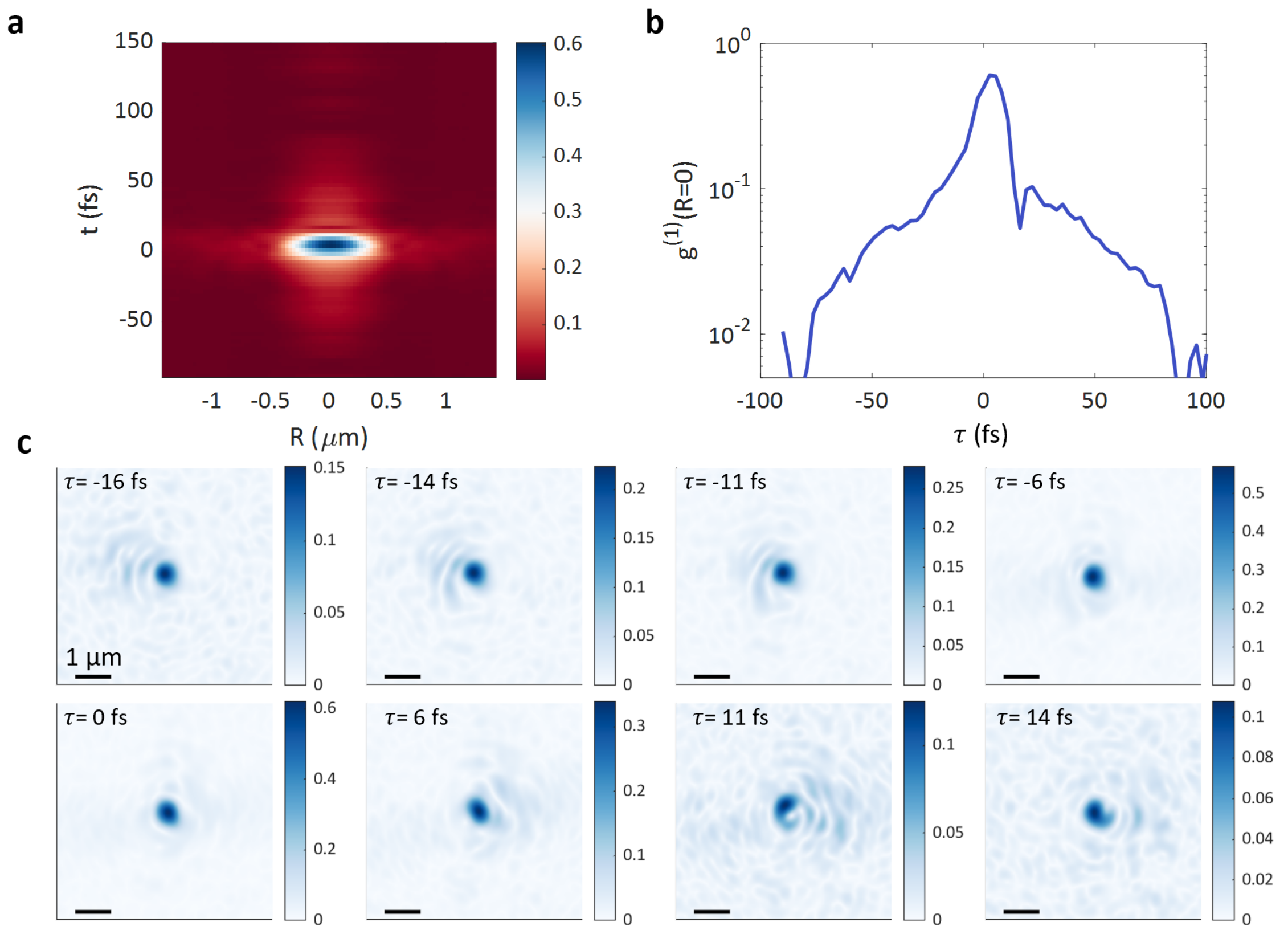

Supplementary Figure S34: **Vortex interferograms and analysis for MB:CB=1. a,** Spatio-temporal $g^{(1)}(R,\tau)$ decay for MB:CB $= 1$. **b,** Extracted time decay at the centre of the pumped region $g^{(1)}(R = 0,\tau)$ for pump power P=0.2 mW. **c**, Vortex phase dislocation in the $g^{(1)}\,(x, y, \tau)$ in space and time, corresponding to zero observed in $g^{(1)}\,(R = 0, \tau)$ coherence trace in (a). All images share the same scale bar.

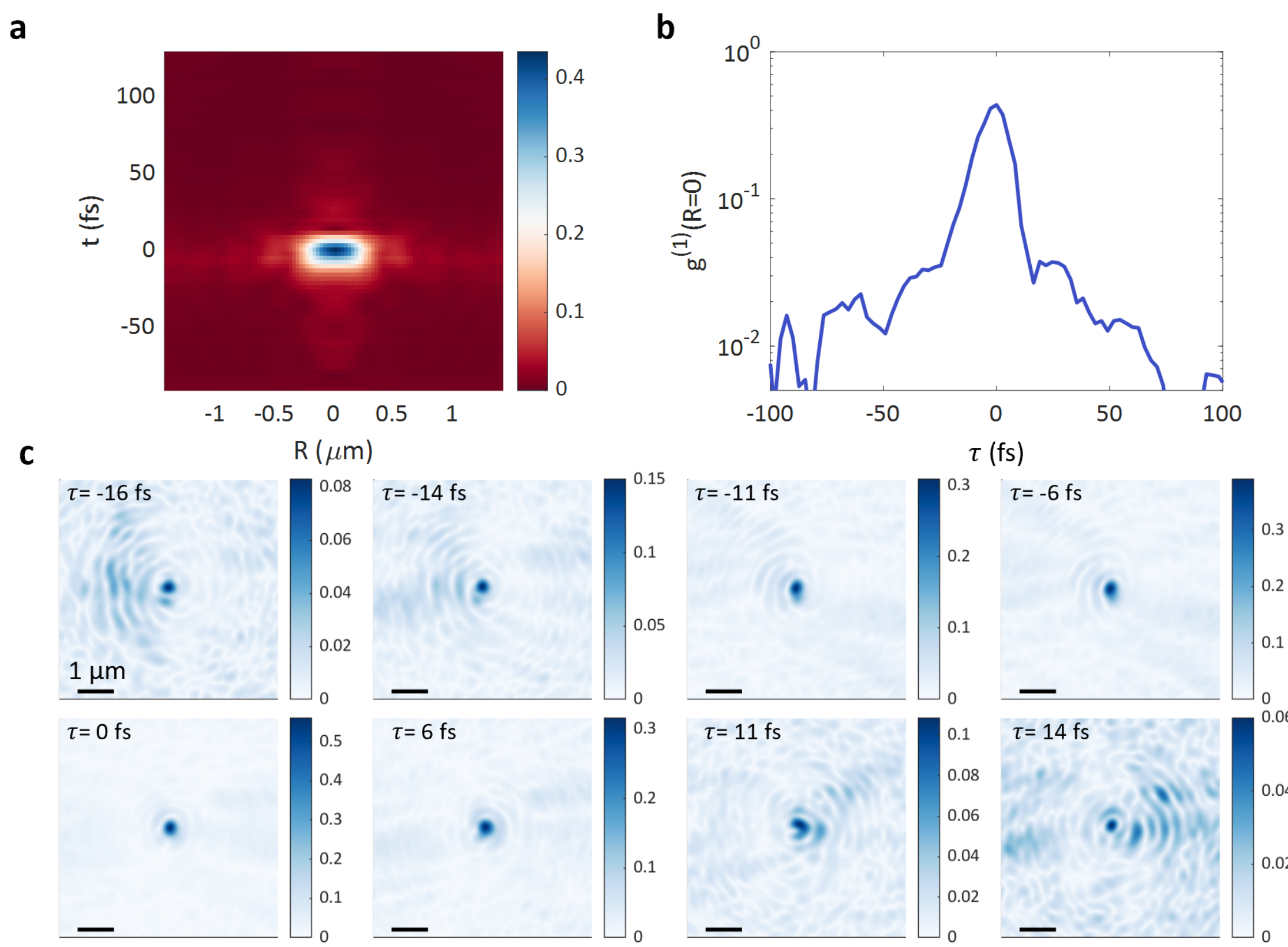

Supplementary Figure S35: **Vortex interferograms and analysis for MB:CB=1. a,** Spatio-temporal $g^{(1)}(R,\tau)$ decay for MB:CB $=$ 1. **b,** Extracted time decay at the centre of the pumped region $g^{(1)}(R = 0, \tau)$ for pump power P=0.2 mW. **c**, Vortex phase dislocation in the $g^{(1)}(x, y, \tau)$ in space and time, corresponding to zero observed in $g^{(1)}(R = 0, \tau)$ coherence trace in (a). All images share the same scale bar.

SI Section S8. Polarization and k-space measurements

The polarization state does not change with pump power, even in the regime where synchronization occurs. We note that the polarization state is less strong than that of the initial highly polarized pump laser due to the modes of the plasmonic nanogaps depolarizing the emission. The modes of plasmonic chains and aggregates possess different polarization components [6, 36] which may result in the emitted light possessing a different orientation to the pump laser beam. This is also further indicative that the emission is not coherent with the pump laser and does not fully inherit the properties of the pump laser.

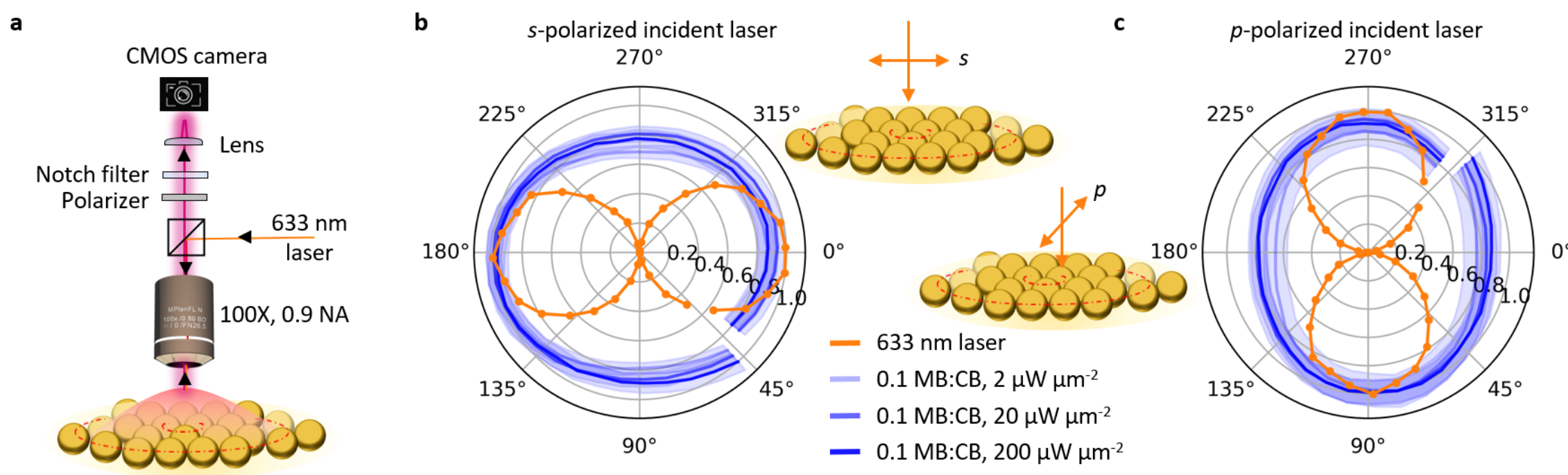

Supplementary Figure S36: **Emission polarization measurements. a,** Emission polarization setup: wire-grid polarizer measures emission polarization on CMOS camera. **b,c,** Total normalized emission intensity vs polarizer angle for the pump laser control for (b) s-polarized and (c) p-polarized excitation. MB:CB=0.1 sample at three different pump intensities below/above synchronisation, over 10 locations (shaded area = standard deviation).

Measurement of the emission polarization in a k-space measurement configuration was performed by imaging the total emitted light in the back-focal plane of the illumination objective lens (Supplementary Figure **S37**a). Supplementary Figure **S37**b,c show that the total emission intensity follows the same trend with pump power, and the emission k-space does not significantly change, with only a slight increase at higher wave-vectors/emission angles.

We have a tightly focused pump laser which excites the system in a range of k-vectors from the normal up to the maximum NA of the objective, which is shown in the fall-off of signal in Supplementary Figure **S37**c below. The film composed of near-field coupled nanoparticle arrays scatters the incident light field into a range of directions, shown by the k-space emission pattern even under normal incidence in Supplementary Figure **S38**. Despite the excitation being at normal incidence after the pump laser passes through the Bertrand lens and being focused on the back-focal plane of the objective lens (Supplementary Figure **S38**a), the emission pattern remains almost spatially even, with only an increase towards higher angles at higher pump powers (Supplementary Figure **S38**b).

Hence, the combined emission pattern in Supplementary Figure **S37**c contains both contributions: the range of incident angles and $k_{\parallel}$, and also the scattered angular range of emitted light

under each angle of incidence. This makes it harder to determine any clear angular narrowing, like in the case of a microcavity polariton which has a clear wavelength scale angular dispersion. In contrast, the $g^{(1)}$ measurements are not necessarily correlated to the angular range, especially for sub-wavelength scattering or emitting objects. It is a more sensitive probe, as it purely looks at field correlations from points R to $-R$ about the centre of the image.

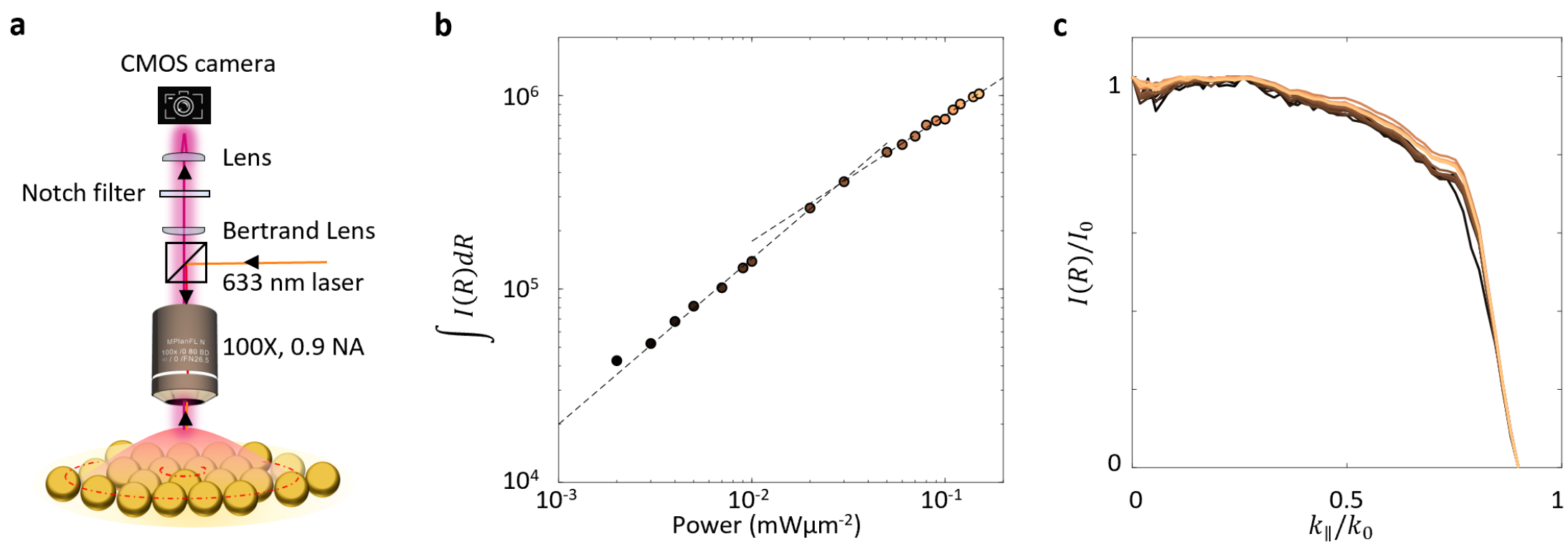

Supplementary Figure S37: **k-space emission measurements. a,** Emission k-space setup, where a k-space/Bertrand lens images the back-focal plane of the objective lens and the reciprocal/k-space information is imaged to a CMOS camera. **b,** Total k-space emission intensity (in cts/s) pump power increases for MB:CB=0.1 samples averaged over 5 positions. **c,** Normalized k-space emission averaged over 5 positions for a range of powers with colours indicated in b.

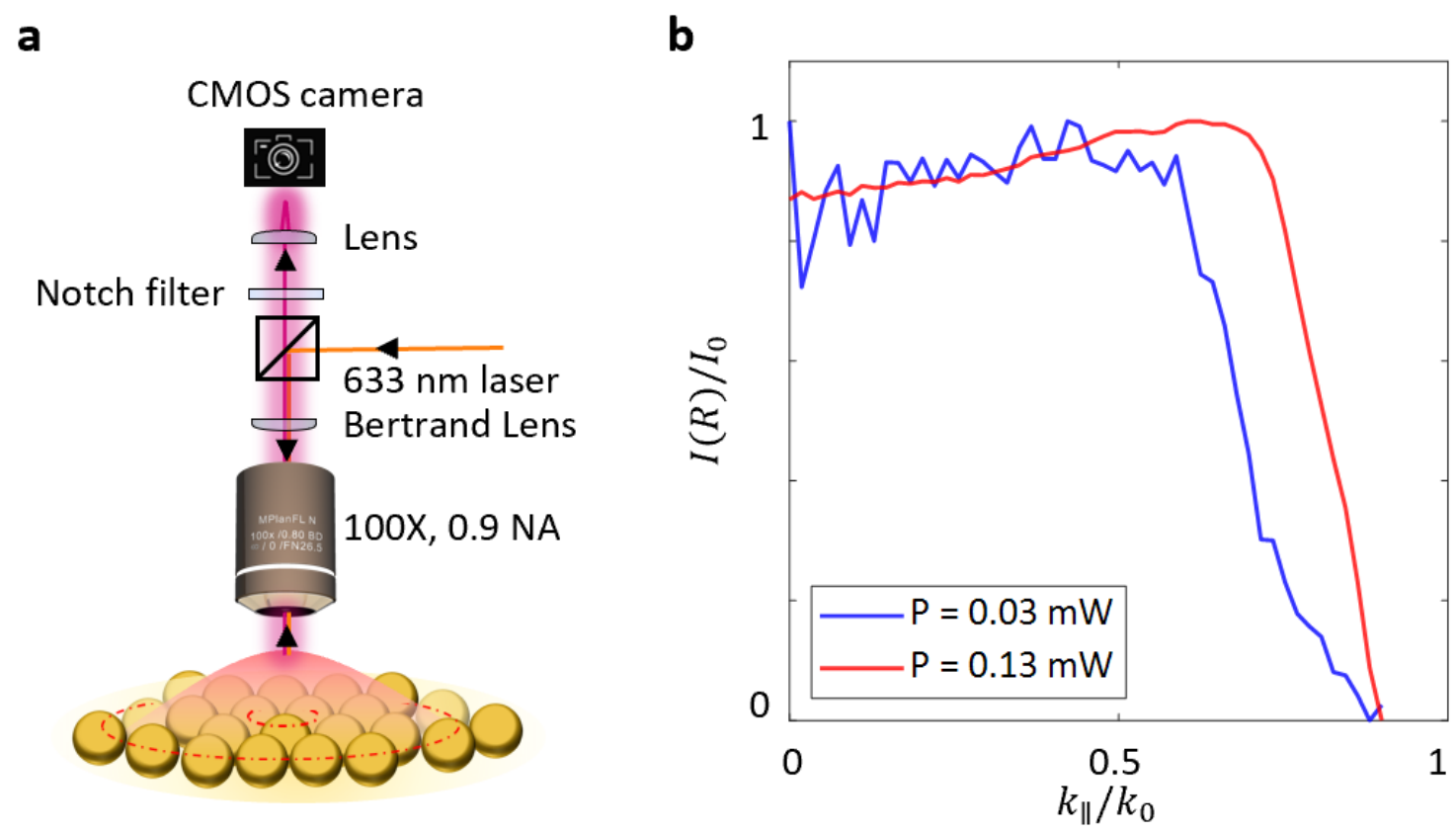

Supplementary Figure S38: **k-space emission measurements under normal incidence laser excitation. a,** Emission k-space setup, where a k-space/Bertrand lens images the back-focal plane of the objective lens and the reciprocal/k-space information is imaged onto a CMOS camera. The Bertrand lens is placed after the beamsplitter in-coupling the laser, hence laser illumination is now normal to the sample. **b,** Total k-space emission intensity as pump power increases for MB:CB=0.1 samples averaged over 5 positions for two different pump powers. Note the focal spot of the laser is restricted to normal incidence with the Bertrand lens, giving larger areas and reducing the intensity.

SI Section S9. Pump spot size dependence and imaging

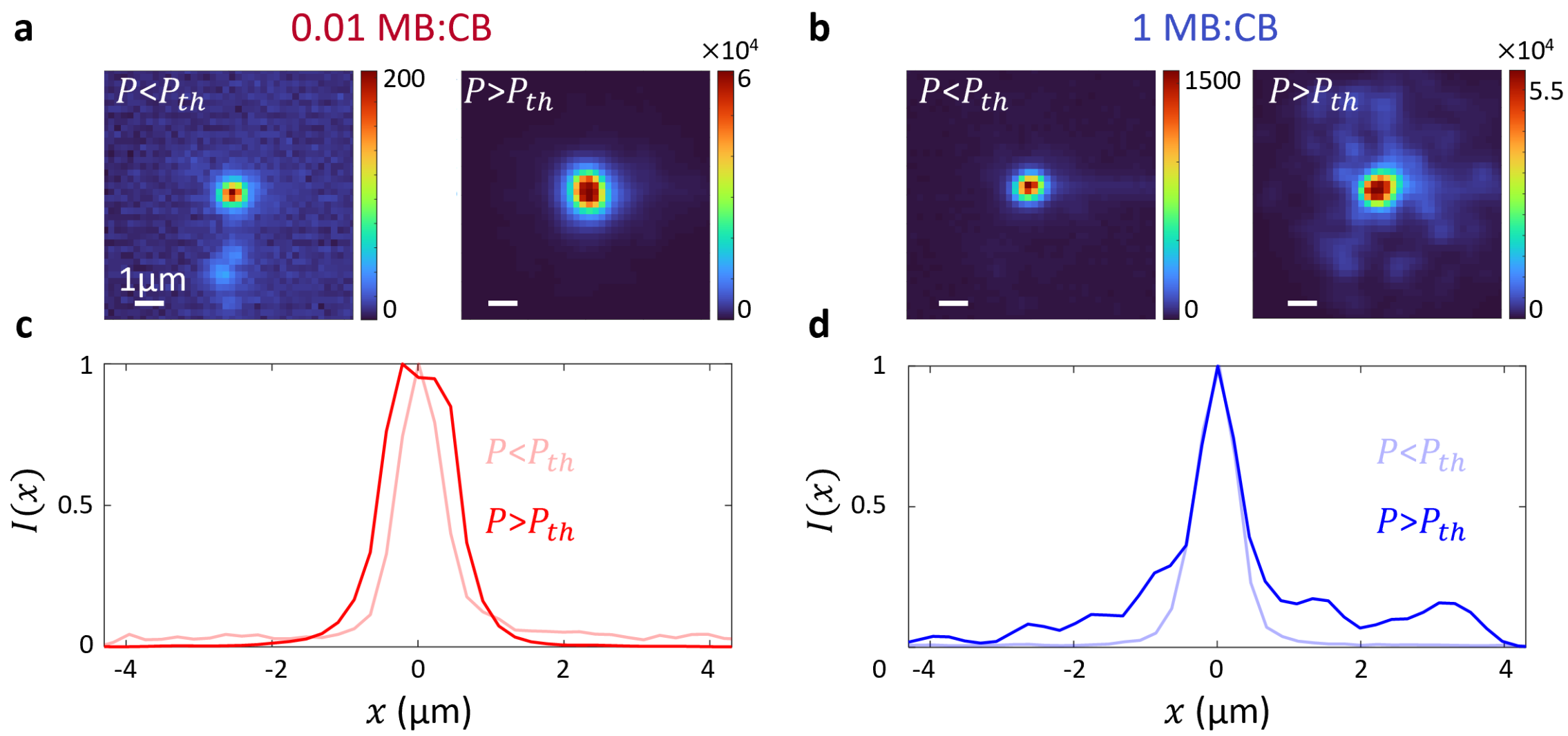

Supplementary Figure S39: **Line profiles for halo formation. a,b,** Imaged emission intensity maps (in cts/s) below (P=2μW) and above (P=130μW) for (a) 0.01 and (b) 1 MB:CB ratios. All images share the same scale bar. **c,d,** Normalized horizontal cross-sections of the images in (a,b) through their centres.

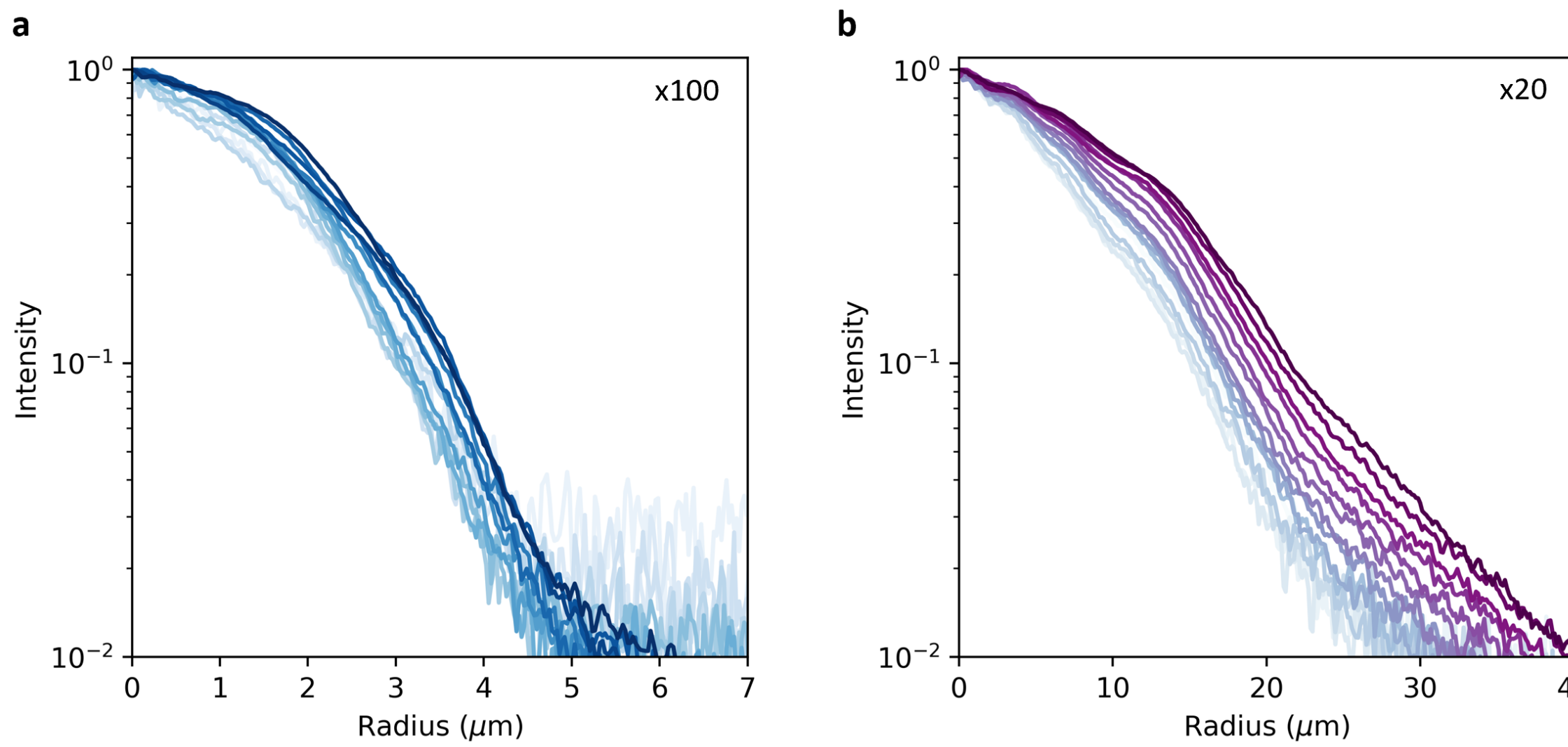

Supplementary Figure S40: **Measurements comparing x20 and x100 objectives.** **a,** Radially averaged normalized emission spatial pattern for MB:CB=0.1 over a range of pump powers with a 100X 0.9NA objective lens. **b,** Same as (a) with instead a 20X, 0.4NA objective lens. Colours corresponding to powers shown in Supplementary Figure S41 below.

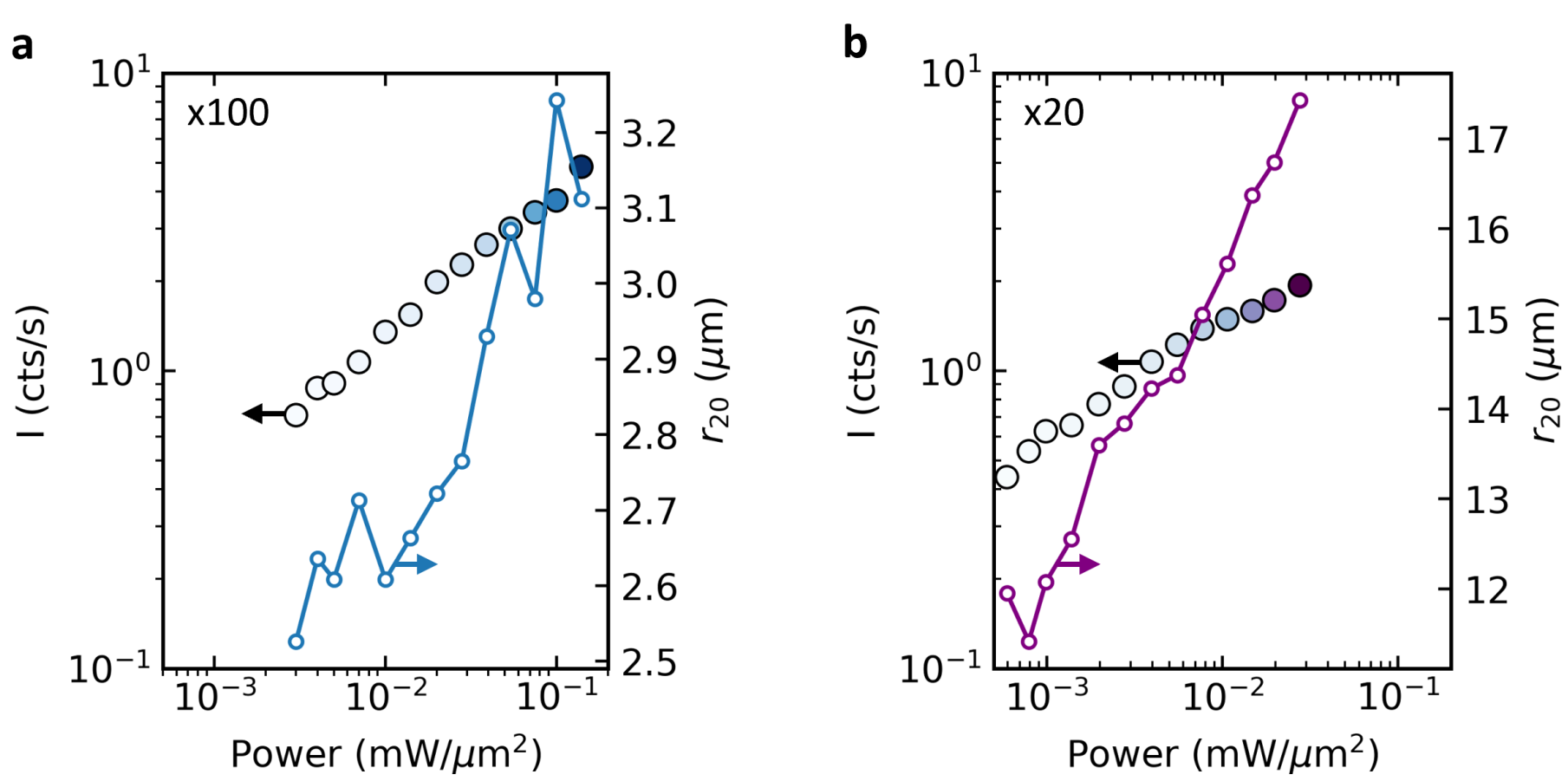

Supplementary Figure S41: **Intensity and halo size comparing x20 and x100 objectives.** **a,** Total emission intensity and spatial size (radial position where normalized intensity drops to 20%) vs pump power for MB:CB=0.1 with a 100X 0.9NA objective lens. **b,** Same as (a) with instead a 20X, 0.4NA objective lens. Colours corresponding to spatial profiles shown in Supplementary Figure S40.

SI Section S10. Comparison of dipole synchronization systems

Below, we provide the references and value ranges used to construct the schematic in Fig. 5d.

**System**	**Temporal Coherence (Q)**	**Mode Volume V** (λ^3)	**Q/V** (λ^{-3})	**Spatial Coherence Index**
Conventional laser [37]	10^5–10^6	~ 1	10^5–10^6	10–100
Random laser [38, 39]	10^2–10^3	~ 1	10^2–10^3	0.5–2
Spaser [40]	10–100	10^{-3}–10^{-2}	10^4–10^5	0.1–1
Superluminescence [41–43]	10–100	~ 1	10–100	1–5
Photon BEC [44]	10^3–10^4	~ 100	10–100	5–20
Exciton-polariton condensate [17]	10^2–10^3	~ 10	10–100	2–10
Atomic BEC [45, 46]	10^1–10^3	~ 1	~ 1	10–100
**Synchronized dipoles (this work)**	10–50	10^{-3}	10^4–5×10^4	2–10

Table 2: **Representative coherence properties of diverse synchronized systems.** Optical values are referenced to a wavelength $\lambda = 700\,\text{nm}$. Where applicable, mode volume V is given in units of λ^3. Spatial coherence is expressed as the ratio of system size to coherence length.

References

(1) Arul, R.; Grys, D.-B.; Chikkaraddy, R.; Mueller, N. S.; Xomalis, A.; Miele, E.; Euser, T. G.; Baumberg, J. J. *Light: Science & Applications* **2022**, *11*, 281.

(2) Arul, R.; Menghrajani, K.; Rider, M. S.; Chikkaraddy, R.; Barnes, W. L.; Baumberg, J. J. *Physical Review Letters* **2023**, *131*, 126902.

(3) Horton, M. J.; Ojambati, O. S.; Chikkaraddy, R.; Deacon, W. M.; Kongsuwan, N.; Demetriadou, A.; Hess, O.; Baumberg, J. J. *Proceedings of the National Academy of Sciences* **2020**, *117*, 2275–2281.

(4) Chikkaraddy, R.; Arul, R.; Jakob, L. A.; Baumberg, J. J. *Nature Photonics* **2023**, *17*, 865–871.

(5) Johnson, P. B.; Christy, R.-W. *Physical Review B* **1972**, *6*, 4370.

(6) Esteban, R.; Taylor, R. W.; Baumberg, J. J.; Aizpurua, J. *Langmuir* **2012**, *28*, 8881–8890.

(7) Feist, J.; Galego, J.; Garcia-Vidal, F. J. *ACS Photonics* **2018**, *5*, 205–216.

(8) Fowler-Wright, P. Mean-Field and Cumulant Approaches to Modelling Organic Polariton Physics, Thesis, PhD Doctor of Philosophy, St Andrews UK: University of St Andrews, 2024, 217 pp.

(9) Kubo, R. *J. Phys. Soc. Jpn.* **1962**, *17*, 1100–1120.

(10) Downing, C. A.; Mariani, E.; Weick, G. *J. Phys.: Condens. Matter* **2018**, *30*, 025301.

(11) Mikhnenko, O. V.; Blom, P. W.; Nguyen, T.-Q. *Energy & Environmental Science* **2015**, *8*, 1867–1888.

(12) Kumar, S.; Dunn, I. S.; Deng, S.; Zhu, T.; Zhao, Q.; Williams, O. F.; Tempelaar, R.; Huang, L. *Nature Chemistry* **2023**, *15*, 1118–1126.

(13) Uddin, S. Z.; Higashitarumizu, N.; Kim, H.; Yi, J.; Zhang, X.; Chrzan, D.; Javey, A. *ACS nano* **2022**, *16*, 8005–8011.

(14) Sortino, L.; Gülmüs, M.; Tilmann, B.; de S. Menezes, L.; Maier, S. A. *Light: Science & Applications* **2023**, *12*, 202.

(15) May, V. *The Journal of Chemical Physics* **2014**, *140*, 054103.

(16) Khazanov, T.; Gunasekaran, S.; George, A.; Lomlu, R.; Mukherjee, S.; Musser, A. J. *Chemical Physics Reviews* **2023**, *4*, 041305.

(17) Bloch, J.; Carusotto, I.; Wouters, M. *Nature Reviews Physics* **2022**, *4*, 470–488.

(18) Fontaine, Q.; Squizzato, D.; Baboux, F.; Amelio, I.; Lemaître, A.; Morassi, M.; Sagnes, I.; Le Gratiet, L.; Harouri, A.; Wouters, M., et al. *Nature* **2022**, *608*, 687–691.

(19) Christmann, G.; Tosi, G.; Berloff, N. G.; Tsotsis, P.; Eldridge, P. S.; Hatzopoulos, Z.; Savvidis, P. G.; Baumberg, J. J. *New Journal of Physics* **2014**, *16*, 103039.

(20) Tosi, G.; Christmann, G.; Berloff, N.; Tsotsis, P.; Gao, T.; Hatzopoulos, Z.; Savvidis, P.; Baumberg, J. *Nature Physics* **2012**, *8*, 190–194.

(21) Lagoudakis, K. G.; Wouters, M.; Richard, M.; Baas, A.; Carusotto, I.; André, R.; Dang, L. S.; Deveaud-Plédran, B. *Nature Physics* **2008**, *4*, 706–710.

(22) Orfanakis, K.; Tzortzakakis, A.; Petrosyan, D.; Savvidis, P.; Ohadi, H. *Physical Review B* **2021**, *103*, 235313.

(23) Damm, T.; Dung, D.; Vewinger, F.; Weitz, M.; Schmitt, J. *Nature Communications* **2017**, *8*, 158.

(24) Mueller, N. S.; Arul, R.; Kang, G.; Saunders, A. P.; Johnson, A. C.; Sánchez-Iglesias, A.; Hu, S.; Jakob, L. A.; Bar-David, J.; de Nijs, B., et al. *Nature Communications* **2023**, *14*, 5726.

(25) Jakob, L. A.; Deacon, W. M.; Zhang, Y.; de Nijs, B.; Pavlenko, E.; Hu, S.; Carnegie, C.; Neuman, T.; Esteban, R.; Aizpurua, J., et al. *Nature Communications* **2023**, *14*, 3291.

(26) Köhler, A.; Bässler, H., *Electronic processes in organic semiconductors: An introduction*; John Wiley & Sons: 2015.

(27) Kostjukova, L. O.; Leontieva, S. V.; Kostjukov, V. V. *Journal of Molecular Liquids* **2021**, *336*, 116369.

(28) Abou Taka, A.; Lu, S.-Y.; Gowland, D.; Zuehlsdorff, T. J.; Corzo, H. H.; Pribram-Jones, A.; Shi, L.; Hratchian, H. P.; Isborn, C. M. *Journal of Chemical Theory and Computation* **2022**, *18*, 3039–3051.

(29) Munkhbat, B.; Wersäll, M.; Baranov, D. G.; Antosiewicz, T. J.; Shegai, T. *Science Advances* **2018**, *4*, eaas9552.

(30) Kéna-Cohen, S.; Wiener, A.; Sivan, Y.; Stavrinou, P. N.; Bradley, D. D.; Horsfield, A.; Maier, S. A. *ACS Nano* **2011**, *5*, 9958–9965.

(31) Cang, H.; Liu, Y.; Wang, Y.; Yin, X.; Zhang, X. *Nano Letters* **2013**, *13*, 5949–5953.

(32) Lee, A.; Wu, S.; Yim, J. E.; Zhao, B.; Sheldon, M. T. *ACS Nano* **2024**, *18*, 19077–19085.

(33) Boehmke, A.; Bar-David, J.; Sibug-Torres, S.; de Nijs, B.; Ferere, A. B.; Large, N.; Baumberg, J. J. *Nano Letters* **2025**, *25*, 15817–15823.

(34) Panico, R.; Comaron, P.; Matuszewski, M.; Lanotte, A.; Trypogeorgos, D.; Gigli, G.; Giorgi, M. D.; Ardizzone, V.; Sanvitto, D.; Ballarini, D. *Nature Photonics* **2023**, *17*, 451–456.

(35) Carusotto, I.; Ciuti, C. *Reviews of Modern Physics* **2013**, *85*, 299–366.

(36) Carnegie, C.; Chikkaraddy, R.; Benz, F.; De Nijs, B.; Deacon, W. M.; Horton, M.; Wang, W.; Readman, C.; Barrow, S. J.; Scherman, O. A.; Baumberg, J. J. *ACS Photonics* **2017**, *4*, 2681–2686.

(37) Vahala, K. J. *Nature* **2003**, *424*, 839.

(38) Andreasen, J.; Asatryan, A.; Botten, L.; Byrne, M.; Cao, H.; Ge, L.; Labonté, L.; Sebbah, P.; Stone, A.; Türeci, H. E., et al. *Advances in Optics and Photonics* **2010**, *3*, 88–127.

(39) Wiersma, D. S. *Nature physics* **2008**, *4*, 359–367.

(40) Azzam, S. I.; Kildishev, A. V.; Ma, R.-M.; Ning, C.-Z.; Oulton, R.; Shalaev, V. M.; Stockman, M. I.; Xu, J.-L.; Zhang, X. *Light: Science & Applications* **2020**, *9*, 90.

(41) Lu, H.; Alkhazragi, O.; Wang, Y.; Almaymoni, N.; Yan, W.; Gunawan, W. H.; Lin, H.; Park, T.-Y.; Ng, T. K.; Ooi, B. S. *npj Nanophotonics* **2024**, *1*, 9.

(42) Gross, M.; Haroche, S. *Physics reports* **1982**, *93*, 301–396.

(43) Kocharovsky, V. V.; Zheleznyakov, V. V.; Kocharovskaya, E. R.; Kocharovsky, V. V. *Physics-Uspekhi* **2017**, *60*, 345.

(44) Klaers, J.; Schmitt, J.; Vewinger, F.; Weitz, M. *Nature* **2010**, *468*, 545–548.

(45) Sinatra, A.; Castin, Y.; Witkowska, E. *Physical Review A* **2009**, *80*, 033614.

(46) Hagley, E. W.; Deng, L.; Kozuma, M.; Trippenbach, M.; Band, Y.; Edwards, M.; Julienne, P.; Helmerson, K.; Rolston, S.; Phillips, W. *Physical Review Letters* **1999**, *83*, 3112.
