# Supplementary material for: Coherent room-temperature dipole synchronization in plasmonic nanocavities
Source: Nat Nanotechnol. 2026 Jul 30;21(9):1230–7. doi: 10.1038/s41565-026-02242-w (PMC13569432; doi:10.1038/s41565-026-02242-w)
Supplement: Supplementary file 2 [file 41565_2026_2242_MOESM2_ESM.pdf]

nature portfolio

Peer Review File

Coherent room-temperature dipole synchronization in plasmonic nanocavities

Corresponding Author: Professor Jeremy Baumberg

This file contains all reviewer reports in order by version, followed by all author rebuttals in order by version.

A version of this paper was originally rejected for publication by Nature Nanotechnology, however that decision was reconsidered after appeal by the authors.

Version 0:

Reviewer comments:

Reviewer #1

(Comments for the Author)
Detailed comments attached

Reviewer #2

(Comments for the Author)
In this work the authors report the formation of room temperature synchronized molecular emission by coupling to plasmonic nanoparticles under incoherent CW pumping. A detailed theoretical model is developed to explain the results. The idea of generating coherence via interparticle plasmonic coupling is interesting. However, the experimental results presented in this work does not support the main claims and hence I cannot recommend this work for publication in Nature Nanotech.
Below are the main concerns:
1. Why do authors claim incoherent CW pumping? Aren't they using a laser?
2. What is the role of the spatial coherence of the pump laser on the observed spatial coherence? i.e if you were to use a truly incoherent source like white light or an LED can spatial coherence be observed?
3. Can one think of this observation as the result of short radiative lifetime and hence the emission doesn't have enough time to undergo decoherence? i.e essentially the photons retain the coherence information only because the lifetime of the emitter is so short in the plasmonic cavity.
4. Observation of coherence from molecular emitters coupled to plasmonic structures (without lasing or condensation) has been reported before (Phys. Rev. Lett. 108, 066401 (2012)).
5. How does the spatial coherence vary across the samples? The authors need to show this effect at least at a few different pump locations and show the variation across the sample.
6. As per Fig. 1 h , I, it appears that the threshold is around 10^-2 mW for the MB:CB of 1 and almost no clear threshold behavior for the MB:CB of 0.01. However, in Fig. 2, where g1 is shown, the threshold where the coherence sets in seems to be 10^-3 mW. Also, what is the collection of datapoints in Fig. 2d around 10^-2 that does not lie near the theory graph?
7. The authors state the distinction of the present system to be absence of spectral narrowing and rapid decay of temporal coherence. I don't see any spectral data! And why would one want short temporal coherence?
8. What is the polarization state of the light before and after synchronization? Does it depend on the polarization of the laser, and does it also become synchronized in space?
9. What happens under pulsed excitation? Can this system transition to conventional lasing in that regime? What about heating in the CW regime?
10. The authors mention "without directional emission" in abstract, but this statement is never supported in the manuscript.
11. Also, in the abstract the authors claim "scalable ambient operation." This requires clarification, what type of scalability do the authors have in mind?
12. In Fig. 1e,f there are no color bars, and the central signal appears saturated. In Fig. 1h the caption is missing an explanation of what the arrows indicate. In addition, for the ratio of 0.1 the threshold is not clearly visible, why is this the case?
13. Is strong focusing of the laser spot required for the synchronization to occur? Would it still appear for a larger spot, e.g., with a 2 um diameter, if provided power density is comparable? Then, how does the core/halo diameter scale?

14. What is the source of the revival of coherence observed in Fig. 2e?
15. In Fig. 3a, for a ratio of 0.01 the flattening appears very unconvincing. Moreover, the authors should explicitly refer to the SI Section 5 explaining how the in-coupled power is calculated.
16. How are the thresholds estimated?
17. What is the purpose of the Raman measurement? They point to the emergence of a new peak, but do not discuss what is the origin and how that has anything to do with the coherence in this system.
18. The purpose of the WSe2 experiments is not clear. Why is the WSe2 a control sample? Wont the excitons in the WSe2 couple via the plasmonic modes similar to the dye? And why is it compared to 0.01MB:Cb system and not the MB:CB = 1 or 0.1 system?
19. The authors talk about enhanced coherence in the conclusion. Is it really enhanced or it is just showing up in the photons emitted because of the short lifetime? And of so what use is the coherence if it is so short lived?

Minor comments:

1. In the Methods section, the authors should explicitly state that the laser is continuous-wave and that the spot size is 0.5 um.
2. There are numerous small editorial errors, e.g., inconsistent spacing between citations, figure numbers, and units, also in SI.
3. In the SI section Electromagnetic simulation there is a typo "nnaoparticle".
4. The results from SI Sections S7 and S8 are not discussed in the main text.

The most interesting data was the time resolved vortex formation (shown in Fig. 5). Does that alone warrant publication in Nature Nanotech? Finally the introduction and the discussion section of the paper are very well written making some interesting connections to other fields.

Reviewer #3

(Comments for the Author)
The manuscript by Arul et al. claims the experimental observation of dipole synchronisation in a plasmonic nanogap array under cw pumping.

The justification of their claim is based on the observation of a halo above some power threshold combined with short temporal coherence. Moreover, they claim that the system features long range spatial coherence.

I think that the topic of this work is definitely of interest and that it would be suitable for publication in Nature Nanotechnology if I were convinced of the validity of their claims.

There are however several points that should be clarified to me before I can accept the manuscript.

1) The short temporal coherence is seen as a feature that supports the synchronisation of the dipoles. This is counterintuitive to me. When a large number of dipoles synchronise, they form a macroscopic polarisation that should behave classical, i.e. have some robustness against fluctuations much in the same way as the phase of a coherent optical field. The absence of an increase of the temporal coherence time therefore seems to cast doubt on the true synchronisation of the dipoles.
2) In the abstract it is stated that there is a spread of g^(1) coherence without directional emission. This seems to contradict the relation between near and far field emission. When there is spatial coherence, the directional emission should be a direct consequence. I do not see how truly synchronised dipoles can emit non directed. The light emission should inherit the coherence properties of the dipoles, no?
3) Fig. 2d shows g^(1)(R=0,tau=0), which by definition should always be equal to one, no? Such a strong variation rather casts doubt on the experiment than show a transition to a synchronised regime.

I also have some further more technical questions

1) What is the physical origin of the pair annihilation mechanism in the model?
2) In Fig. 2.c, the spatial coherence in the theory decays quickly in both regimes. Is there really a synchronisation transition in the theory?
3) line 230: You say: “Spatial coherence arises from dipoles synchronizing their phases through evanescent coupling and nonlinear dipole-nanocavity interactions.” Can you point out which terms in the model represent this physics?
4) line 242: you say that the observed vortices are related to the Kibble-Zurek mechanism. That is rather for systems with a quench, no? And those vortices are spontaneous and should not be visible in time-averaged interferrograms.
5) line 243: you write “The vortex with $g(1)$=0 traverses the centre R=0”. What does this mean?line 268 mentions Ref. (7). This reference is on interacting dipoles. It is not clear to me how this theory work supports the system that you are studying.
6) line 265: you write “constrained lateral coherence reflects phase diffusion as near-fields propagate outward from the core pumped region.” Which phase diffuses here? Do you mean that there is diffusion of excitations due to the propagation?
7) The connection to the cold atom reference (45) seems inappropriate. This work is about the phase fluctuations in a 1D thermalised system, which is very different from the driven-dissipative physics under study here.

In general, it is my impression that the authors have sacrificed clarity for trying to highlight the great impact of the work, using technical terms not very carefully.

It is from the current discussion actually not clear to me how much synchronisation is claimed. For example in lines 275, they write that there is “localised coherence”. What does this imply for the length scale of the synchronisation of the dipoles. Is it only between neighbouring cavities? Do they mean that there are short-lived patches of dipoles that synchronise? Does this correspond to the “synchronised” state in the usual sense? I would rather say that it corresponds to a desynchronised regime in the usual meaning of the word.

In conclusion, I think that the paper needs a careful rewriting, making sure that technical terms are used appropriately and that the claims are made precise. I actually doubt that there is very good synchronisation in this system.

Version 1:

Reviewer comments:

Reviewer #1

(Comments for the Author)
I have carefully reviewed the response and revised version. The authors have made improvements and addressed my earlier concerns in the revised version. I would recommend its publication in its present form.

Reviewer #2

(Comments for the Author)
I have read the rebuttal by the authors to the comments raised by the three reviewers. I must confess that I am now even more confused about the claim of synchronization!
The paper and the rebuttal is packed with jargons and as the Reviewer 3 rightly points out: "the authors have sacrificed clarity for trying to highlight the great impact of the work, using technical terms not very carefully".
The biggest concern is - why does it matter if there is synchronization at ultrashort timescales?

More specifically here are some comments on the rebuttal:
1. The authors claim that the 2D model shows similar trend as experiments and 1D model. However the presented data does not validate that!
2. What is the physical interpretation of vortex formation in g1? Doesn’t it imply loss of long range spatial order? How many such vortices do you observe in the experiments?
3. For my second question, the authors discuss the coherence of white light source. That is not what I was asking. My question was: if you used a LED to pump the sample at the same intensity, would you observe spatial coherence as observed under CW laser pump? This is important to show as it will negate any role of the pump laser or its scattering on the observed coherence.
4. My third comment also seems to have been misunderstood. I was asking about the short radiative/non-radiative excited state lifetime due to the plasmonic coupling contributing to the observation. i.e. the excited state lifetime is so short due to plasmonic coupling that there is not enough time to undergo decoherence.
5. The polarization experiments (Fig. S35) seem to indicate that it follows the incident laser polarization although the degree of polarization is lower which is expected. However the emission is clearly not isotropic. If the laser polarization is rotated, does the PL also follow?
6. For the question on scalability, the authors comment: "This is the key nanotechnology advance: a soft matter, self-assembled platform showing synchronization of quantum emitters." Once again maybe I am not aware, what could one do with such short lived synchronization?
7. I am glad the authors repeated the experiments with lower magnification objective (20X) and have shown the data in Fig. S39. However, I do not see the "halo" formation here. Am I missing something?
8. The authors suggest two mechanisms for the collapse-revival feature of coherence. The second one sounds interesting - i.e. transient reconfiguration of spatial phase domains. However, what is puzzling is the periodicity of this collapse-revival phenomena (Fig. 2e). I would have imagined the latter effect to result in random locations for such collapse-revival to happen.

In summary, I am still not convinced that this work represents the kind of advancement that warrants publication in Nature Nanotechnology. The authors tend to use esoteric and complex theoretical ideas to interpret their experimental results. In the end what the authors seem to have is a fluctuation-dominated regime where the phase coherence is destroyed both spatially and temporarily in short time scales.

Reviewer #3

(Comments for the Author)
The rebuttal by Arul et al. is very extensive and answers many of the questions that were raised by the referees. I am still somewhat unsure about several of the claims, such as for example the interpretation of the vortices (referee #2 found this very interesting) in the g^1 map, which seems confusing (also for referee #2). Also the spectrum that is now shown in the sup mat is more complicated than I can expect to follow from the theoretical model. It is stated by the authors that computing the

different time correlators is beyond the current scope.

Looking again at the experimental data and interpretation on the growth of the g^1(R=0), I do now indeed agree that it indicates the growth of spatial coherence. I do not think that the argument on the spatial averaging is entirely correct though. I think that it is rather the size of the interference fringes (on about the same scale) that limits g^1(R=0).

I think that in the current state and with the extended discussions, the paper is now sufficiently sound for publication in Nature Nanotechnology since it can stimulate further experimental and theoretical research in this direction.

Version 2:

Reviewer comments:

Reviewer #2

(Comments for the Author)
Although the explanation of the experimental observation is still not convincing, I am willing to recommend publishing this work since it will likely create interest in this topic.

Open Access This Peer Review File is licensed under a Creative Commons Attribution 4.0 International License, which permits use, sharing, adaptation, distribution and reproduction in any medium or format, as long as you give appropriate credit to the original author(s) and the source, provide a link to the Creative Commons license, and indicate if changes were made.
In cases where reviewers are anonymous, credit should be given to 'Anonymous Referee' and the source.
The images or other third party material in this Peer Review File are included in the article's Creative Commons license, unless indicated otherwise in a credit line to the material. If material is not included in the article's Creative Commons license and your intended use is not permitted by statutory regulation or exceeds the permitted use, you will need to obtain permission directly from the copyright holder.
To view a copy of this license, visit https://creativecommons.org/licenses/by/4.0/

The work by R. Arul et al. demonstrates a room-temperature synchronized dipole phase in locally-ordered plasmonic nanogap 2D arrays in the bad-cavity regime. The CB[7] molecular force the MB dipoles to be perpendicular to the metal facets, leading to the formation of the plasmonic nanocavity with large cavity-dipole coupling and high loss rates. Under an incoherent CW pumping, the authors observe a synchronized dipole phase inside this system. Different from other conventional synchronized phases, this synchronized dipole phase exhibits very fast temporal coherence decay, but without linewidth narrowing or directional emission, which is explained by the 1D driven-dissipative lattice model. These results and the underlying physics are interesting. However, it is not clear what kind of advances it can bring to the field of nanotechnology and future photonic quantum technologies. I think the current findings are more suitable for Nature Communications after addressing the detailed comments below:

1. The central finding of this work is the observation of a synchronized dipole phase that shows extended spatial coherence. However, from Fig. 2, one can find that both the spatial and temporal coherence are quite short, compared with other systems, such as photon/polariton condensation, BEC, etc. It is not clear why such a synchronized dipole phase is important and how it can bring further advances to the field of nanotechnology. The discussion around lines 287-291 introduce some possibilities but remains too broad and insufficiently justified.

2. The authors use a 1D tight-binding model to explain the emergence of the 'halo', incorporating dipole-plasmon coupling g and multiple decay channels. However, the hopping strength term J appears oversimplified, as only nearest-neighbor hopping is included. In addition, the model is one-dimensional whereas the experimental system is evidently two-dimensional. Also, from figure 1c, the nanoparticles are not arranged in good order, suggesting that the coupling between different nanoparticles are different.

3. In Fig. 2c, the theoretical decay of $g^{(1)}(R,-R)$ mismatches with the experimental data outside the core area (R>0.5μm). Please explain this mismatch and, if possible, quantify the behavior.

4. From Fig. 2e and Fig. 2f, the ultrashort coherence time of this unique synchronized phase is evident, and the authors attribute this ultrafast dephasing to the large Purcell enhancement (>1000). However, $g^{(1)}(R=0)$ shows a secondary increase after the initial rapid decay (a collapse-revival like feature), what is the physical origin of this behavior?

5. Figure 3 further examines the threshold behavior of this synchronized phase. However, the change of slope in Figure 3a is quite marginal. It could also be fitted by a linear fitting with error bars. Also, the theoretical calculation does not fit the experimental well for all the three concentrations. At low MB:CB ratios (0.01), the origin of the transitions from linear-sublinear-superlinear is not clear. It would be helpful to clarify the underlying mechanism and the conditions under which these regimes emerge.

6. The author theoretically analyzes the spreading of correlations and coherence, emphasizing that the plasmonic coupling (t) and the pair annihilation (Γ_{ee}) play an important role in the formation of this phase. Is there an experimental route to validate these assignments? (For example, tune the plasmonic coupling (t) by slightly increasing interparticle spacing or introducing spacer layers.)

7. Regarding the vortex features, a well-defined vortex core appears only at ~11 fs in Fig. 5c, while other delays do not show clear vortices, with no apparent trend. In addition, interference images in Fig. S8a and Fig. S11 display fringe bending and splitting without a consistent pattern, suggesting that vortex occurrence in this system may be random. It would be helpful to use a Mach-Zehnder interferometer to retrieve the real-space phase and track its evolution versus time delay for systematically analyzing the vortices emerging mechanism.

8. Figure 1 compares below and above threshold behavior across different MB:CB ratios and clearly shows the emergence of the 'halo' with intensity increasing with Ξ. However, Fig. 1e and 1f do not state the corresponding excitation power they used. The author should label these figures with the corresponding pump power and link them unambiguously to the data points plotted in Fig. 1h. In addition, the arrows in Fig. 1h are not defined.

9. Related to the former comment, if the blue arrow in Fig.1h denote the transition threshold, they appear offset by an order of magnitude from the threshold-like behavior shown in Fig. 2d (the evolution of $g^{(1)}(R = 0)$. Please explain the origin of this offset and determine the transition threshold in this work. Additionally, to further validate the phase transition, it would be helpful to demonstrate the real-space emission and the corresponding interference fringe images under different pumping density from below and above the threshold.

Response to reviewers:

We are delighted that the reviewers find "*these results and the underlying physics are interesting*" with "*the observation of a synchronized dipole phase that shows extended spatial coherence*" (Reviewer #1), that the "*idea of generating coherence via interparticle plasmonic coupling is interesting*" and "*make some interesting connections to other fields*" (Reviewer #2), and that "*the topic of this work is definitely of interest and that it would be suitable for publication in Nature Nanotechnology if I were convinced of the validity*" (Reviewer #3). They bring up many of the same issues. We have thus performed additional measurements and addressed all their points below, which has significantly strengthened the manuscript and justified our claims.

Reviewer #1

1. The central finding of this work is the observation of a synchronized dipole phase that shows extended spatial coherence. However, from Fig. 2, one can find that both the spatial and temporal coherence are quite short, compared with other systems, such as photon/polariton condensation, BEC, etc. It is not clear why such a synchronized dipole phase is important and how it can bring further advances to the field of nanotechnology. The discussion around lines 287-291 introduce some possibilities but remains too broad and insufficiently justified.

> We agree that the spatial and temporal coherence lengths in Fig.2 are short compared to photon/polariton condensates or BEC systems. Crucially, however, our platform operates in the extreme bad-cavity, deeply sub-wavelength plasmonic regime where long-lived coherence and linewidth narrowing is fundamentally impossible due to ultrafast radiative and non-radiative decay. The significance of our observation is therefore not the *magnitude* of coherence, but its *existence at all* under these conditions, and we now extend this discussion as suggested.

In particular, by contrast to condensates or lasers where coherence is seen through mode occupation and spectral narrowing, here we observe phase synchronization of emitters mediated by near-field plasmonic coupling on timescales shorter than dissipation, without linewidth narrowing, directional emission, or lasing. This demonstrates a qualitatively different route to optical phase order that is **compatible with lossy, broadband, and nanoscale architectures**.

The relevant nanotechnology advance here is that such collective phase order emerges in a **self-assembled, room-temperature** plasmonic network made from ubiquitous materials, **without high-Q cavities, epitaxy, cryogenics, or vacuum infrastructure**. This enables scalable architectures that are compatible with sensing and spectroscopy, rather than a route to long-lived coherence or narrow-line lasers. High-Q cavities often fail as sensors under disorder, roughness, temperature drift, but our cavities are robust to these.

We demonstrate a robust, large-area, self-assembled plasmonic network ('MLaggs', [1] below) with reproducible nanoscale hotspots. These can be used in sensing chips, coatings, and heterogeneous integration [2, 3]. In this context, collective phase order provides new functional observables (eg. spatial $g^{(1)}$ maps and vortex statistics) that are sensitive to coupling and dephasing and can be exploited alongside established near-field sensing and spectroscopy modalities. Because MLaggs are already known to host strong IR near-fields and vibrational coupling [2], these collective states provide a plausible route to *phase-based* detection of IR-driven changes (eg. IR absorption modifying local dephasing/coupling), complementing intensity-only enhanced absorption/Raman spectroscopies.

More broadly, short temporal coherence and bad-cavity operation can be advantageous rather than detrimental: in bad-cavity/superradiant lasers, coherence is stored primarily in the emitters and cavity

pulling is strongly suppressed, improving robustness against cavity noise for metrology [4, 5]. Likewise, Purcell-shortened lifetimes can improve practical performance by increasing emission rates [6] and by extracting photons on timescales shorter than slow environmental noise such as spectral wandering, improving photon indistinguishability [7].

The self-assembled platform we have developed shows synchronization of quantum emitters. The field of mesoscopic physics of few quantum emitters is developing [8] and poised to push advances in physics and chemistry - our new platform realizes this and explores new polariton physics. Conventional microcavity polaritons already have a host of applications in switching [9], optical computing [10], study of nonequilibrium physics [11, 12], and control of chemistry [13]. We thus have revised the introduction and discussion to clarify this distinction and to focus on concrete nanotechnology implications: the possibility of engineering spatially coherent emission patterns, nonlinear upconversion, and collective light-matter response in 2D systems where traditional cavity-based coherence is unattainable.

2. The authors use a 1D tight-binding model to explain the emergence of the ‘halo’, incorporating dipole-plasmon coupling g and multiple decay channels. However, the hopping strength term J appears oversimplified, as only nearest-neighbor hopping is included. In addition, the model is one dimensional whereas the experimental system is evidently two-dimensional. Also, from figure 1c, the nanoparticles are not arranged in good order, suggesting that the coupling between different nanoparticles are different.

> This is a helpful suggestion. We now show results of a new 2D model showing similar behaviour as the 1D case in SI Section S3.6. We do not explore the full parameter space as the computational cost of this 2D simulation increases significantly (converged simulations at a single power for 40x40 sites take 30 mins). The effect of disorder is important, however we have shown in previous work that the disorder is mitigated by an effective medium coupling (Ref. [14] below and Ref [31] of the main text), and detailed study of the impact of disorder in nanoparticle arrangement on the optical field confinement is highly detailed and under active study for future work.

3. In Fig. 2c, the theoretical decay of $g(1)(R, -R)$ mismatches with the experimental data outside the core area (R>0.5μm). Please explain this mismatch and, if possible, quantify the behavior.

> We note first that as Fig. 2(c) is on a logarithmic scale, the mismatch occurs in a region where g(1) has already fallen to a small fraction of its peak, so this mismatch refers to discrepancies in a region where the correlation function is very small.

At larger distances, our model indeed does not capture the full complexity of the data. However the purpose of the current model is to provide a simple model which allows us to understand the types of behaviour (phases) possible and their key ingredients and physical origin. While it would be possible to construct a more complex model with multiple fitting parameters and thus fit the data closely, such an approach would obscure the key physics that we believe underlies the observed behaviour. We have thus avoided a model with additional fitting parameters.

4. From Fig. 2e and Fig. 2f, the ultrashort coherence time of this unique synchronized phase is evident, and the authors attribute this ultrafast dephasing to the large Purcell enhancement (>1000). However, $g(1)(R = 0)$ shows a secondary increase after the initial rapid decay (a collapse-revival like feature), what is the physical origin of this behavior?

> Indeed the reviewer highlights an interesting behaviour of this synchronized state. The initial rapid decay of $g^{(1)}(R\text{=}0,\tau)$ reflects the ultrafast radiative decay associated with strong Purcell enhancement. We believe the subsequent collapse-revival-like oscillation arises primarily from spectral beating between distinct components of the emission spectrum.

As shown in new SI Fig. S20 (below), fitting the emission with a broad bi-Gaussian model which captures the main electronic transition and its vibronic shoulder does not reproduce the observed oscillatory structure in $g^{(1)}(R\text{=}0,\tau)$ when Fourier transformed (Fig. S20d, black solid line). Only when enhanced resonance-Raman features are included in the spectral model does the Fourier transform yield an oscillatory behaviour consistent with the measured secondary rise (black dotted line). Indeed the Fourier transform of the full measured spectrum (blue dotted line) reproduces these features of the observed revival amplitude and period.

Spectral beating alone does not account for all features here, particularly the spatial vortex nucleation observed near the first minimum of $g^{(1)}$. As shown in SI Fig. S20b, phase mapping reveals the emergence of phase winding at this delay. We therefore interpret the collapse-revival feature as arising from the interplay of two effects: (i) temporal beating between spectral components and (ii) transient reconfiguration of spatial phase domains associated with vortex formation. We have clarified this interpretation in the revised manuscript and added the new analysis to the SI.

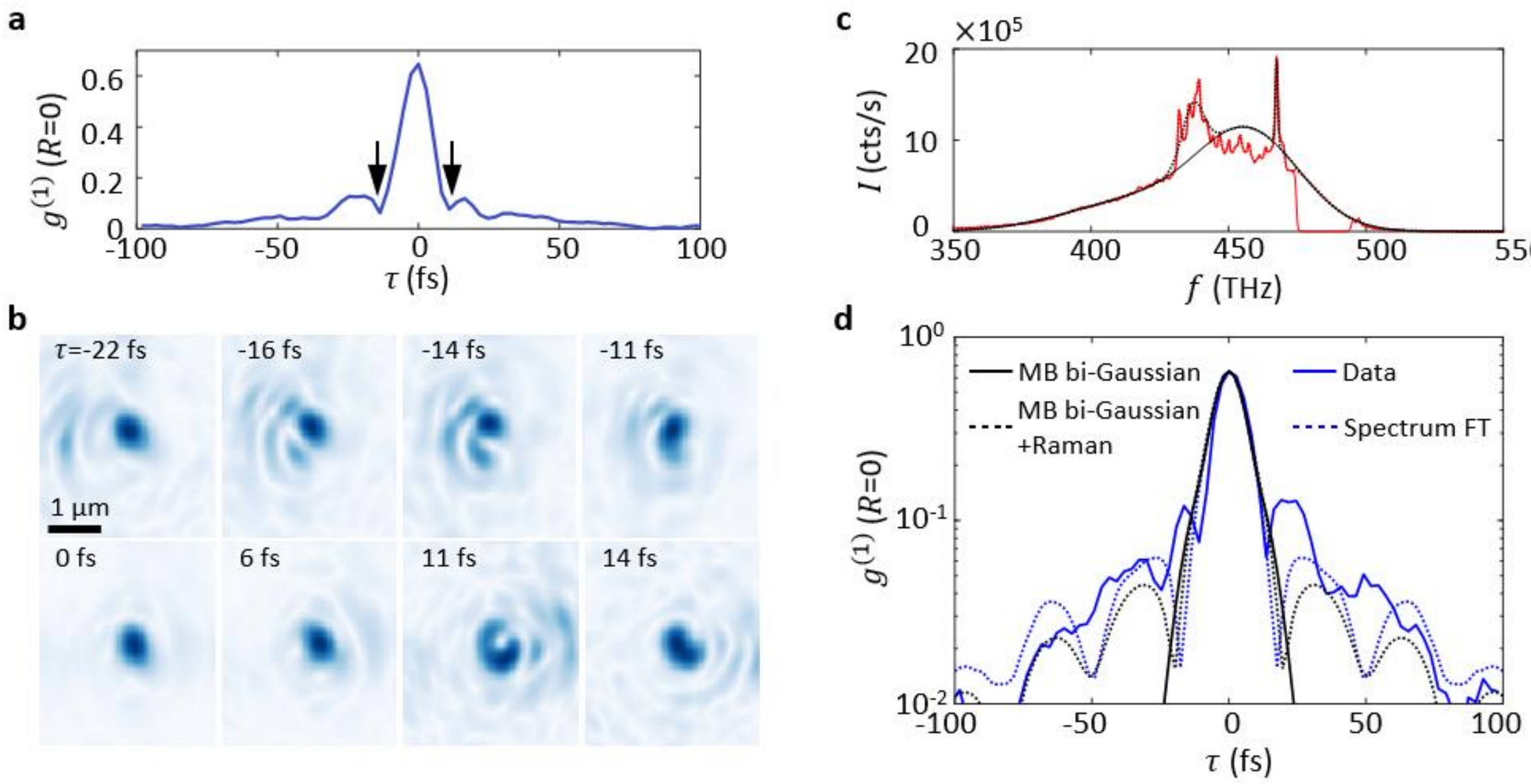

Supplementary Figure S20: **Collapse-revival analysis of $\mathbf{g^{(1)}}$**. **a**, Decay of $g^{(1)}(R\text{=}0,\ \tau)$ for MB:CB = 1 at pump power P=0.2 mW, with arrows indicating formation of vortices degrading the central $g^{(1)}$ at labelled interferometer time delay τ. **b**, Vortex phase dislocation in the $g^{(1)}(x,y,\tau)$ in space and time, corresponding to zero observed in $g^{(1)}(R=0,\tau)$ coherence trace in (a). All images share the same scale bar. **c**, Emission spectrum of MB:CB = 1 corresponding to (a), with solid black lines showing bi-Gaussian fit for MB main emission peak and vibronic side-band. Narrower Gaussian peaks show surface-enhanced resonant Raman peaks of MB. **d**, Decay of $g^{(1)}(R\text{=}0,\ \tau)$ compared to Fourier-transform of the fitted MB emission peak (MB bi-Gaussian), the fitted MB emission and resonance Raman (MB bi-Gaussian + Raman), the entire spectrum in c (Spectrum FT), and the observed data in a (Data).

5. Figure 3 further examines the threshold behavior of this synchronized phase. However, the change of slope in Figure 3a is quite marginal. It could also be fitted by a linear fitting with error bars.

> The way we plotted this previously did not make the change of slope clear, since it is the average data (which has small error bars) that we are fitting. In the new SI Fig. S22 (and below) we show that

the change in slope is not marginal, and clearly goes from a linear to a sublinear dependence (for MB:CB=1, 0.1) and superlinear dependence (MB:CB=0.01).

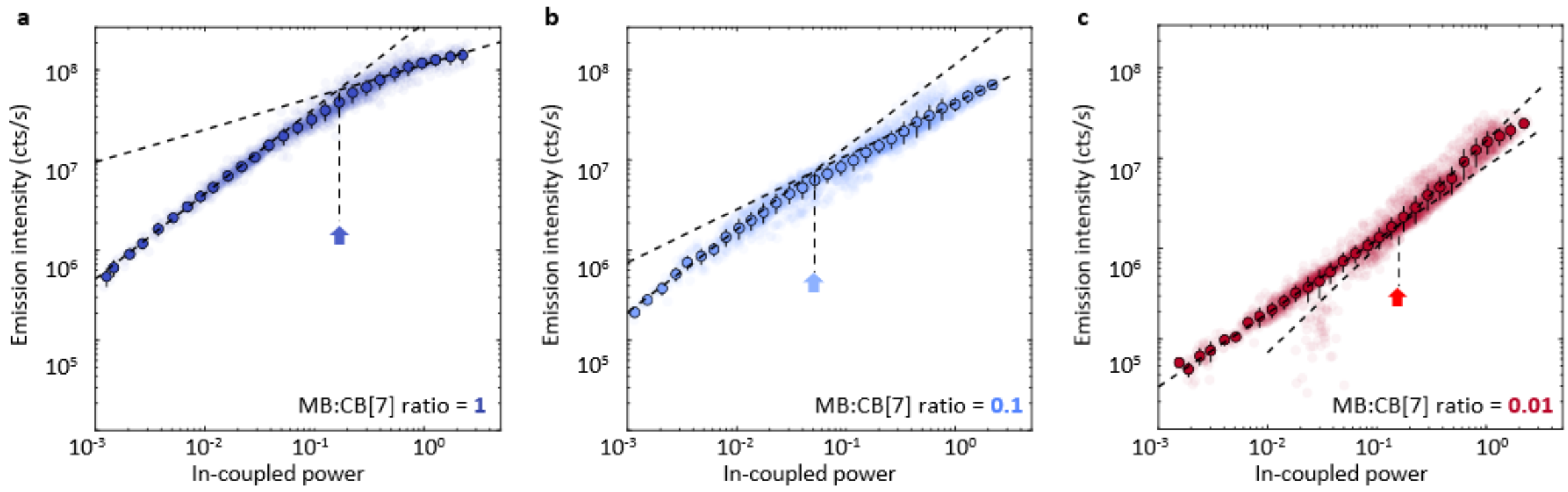

Supplementary Figure S22: **Input-output relation for nanocavity system**. Integrated intensity of MB emission *vs* in-coupled power for increasing dye concentration ($\propto P^{\alpha}$), showing deviations from linear scaling for different regime. **a**, MB:CB = 1, dotted lines show a transition from linear (α=0.95±0.02) to sublinear (α=0.36 ±0.1) power dependence. **b**, MB:CB = 0.1, transition from linear (α=0.92±0.1) to sublinear (α=0.59±.05) power dependence. **c**, MB:CB = 0.01, transition from near linear (α=0.81±0.1) to superlinear (α=1.2±0.1) power dependence. The crossover in-coupled power between the regimes shifts from 0.16, 0.05 and 0.03 for MB:CB = 1, 0.1, and 0.01 respectively.

Also, the theoretical calculation does not fit the experimental well for all the three concentrations. At low MB:CB ratios (0.01), the origin of the transitions from linear-sublinear-superlinear is not clear. It would be helpful to clarify the underlying mechanism and the conditions under which these regimes emerge.

> We believe the transition is not abrupt because it is a continuous evolution between linear and sublinear scaling regimes, as shown by our theory. This transition between the regimes is discussed in the main text ("*Coherence spreading and dipole-correlations*" section) as the point where a spatial emission halo emerges and the phases between adjacent excitons synchronize.

At low pump powers, emission scales linearly because dipoles act independently and the excitation density is low. For increasing pumping, near-field plasmon-mediated coupling establishes finite-range phase synchronization between adjacent nanocavities, leading to the emergence of the spatial emission halo and a redistribution of emission pathways. This collective regime produces sublinear scaling. At higher excitation densities, exciton-exciton annihilation (EEA) becomes significant. For higher MB:CB ratios (larger Ξ), the increased number of emitters per cavity enhances the probability of pair annihilation, introducing strong nonlinear loss that suppresses the emission intensity. This is only possible when there is more than one emitter on average at each nanocavity site, hence this behaviour shows strong universal scaling (Fig. 3c, main text) for all Ξ except for Ξ=0.01. For this low concentration (Ξ = 0.01), reduced EEA weakens this nonlinear loss channel, enabling the superlinear scaling. We now rewrite the main text to make this point clearer.

We now include this discussion in SI Section S3.8 detailing the limitations of our theoretical model. With such considerations, it is a remarkable that the simple theoretical model matches the experiments so well, which we believe captures the essential physics of the system. Further details are the subject of current and future work, requiring significantly pushing the frontier of theoretical simulations of organic molecules in complex nanophotonic cavities.

6. The author theoretically analyzes the spreading of correlations and coherence, emphasizing that the plasmonic coupling (t) and the pair annihilation (Γee) play an important role in the formation of this phase. Is there an experimental route to validate these assignments? (For example, tune the plasmonic coupling (t) by slightly increasing interparticle spacing or introducing spacer layers.)

> This is a great suggestion. In our MLagg platform, the interparticle spacing is templated by the supramolecular complex formed between the emitter (MB) and cucurbit-7-uril (CB[7]), which defines the ~1 nm nanogaps [15] (Fig. 1b). This molecular templating is essential for achieving the extreme field enhancement and strong plasmon-exciton coupling [16] required for observing collective effects. Because the nanogap size is fixed by the CB[7] binding geometry, direct tuning of the plasmonic coupling parameter J via spacer-layer variation is experimentally non-trivial in this system.

However, we can experimentally probe the role of the pair annihilation rate Γ_{ee} by varying the emitter loading Ξ through control of the MB:CB ratio. As shown in Fig. 3a and SI Fig. S22, the power-law scaling changes systematically with Ξ: for average occupancies >1 emitter per nanogap, nonlinear exciton–exciton annihilation modifies the emission scaling, whereas for $\Xi \leq 0.01$, annihilation is suppressed and the scaling behaviour differs markedly. This concentration-dependent scaling provides experimental validation of the role of Γ_{ee} in shaping the collective regime. We now highlight this key experimental control in the main text.

7. Regarding the vortex features, a well-defined vortex core appears only at ~11 fs in Fig. 5c, while other delays do not show clear vortices, with no apparent trend. In addition, interference images in Fig. S8a and Fig. S11 display fringe bending and splitting without a consistent pattern, suggesting that vortex occurrence in this system may be random. It would be helpful to use a Mach-Zehnder interferometer to retrieve the real-space phase and track its evolution versus time delay for systematically analyzing the vortices emerging mechanism.

> We now provide additional Michelson data showing further vortices. Indeed the nucleation time for vortex formation is not fully deterministic, which is characteristic for most polaritonic systems [17]. We discuss this further in a new SI Section S7.1. In brief the vortex formed in Fig.5 is not a traditional condensate vortex, but is a vortex in the $g^{(1)}(R, -R, \tau)$ coherence, which correlates the emission coherence at point $\boldsymbol{R}$ with the point $-\boldsymbol{R}$, at different relative time delays τ of the field excited by a continuous-wave pump. Any vortex must thus have a phase slip of the field for correlations probed at every τ delay of the continuously emitting system. This phase can be retrieved from the Fourier transform itself by subtracting the d.c. component (below, SI Figure S30), as performed in Ref.[17]. While Mach-Zehnder interferometry and interferometry using a reference phase from an expanded homogeneous part of the emission has been used [18], this is difficult in our system due to the inherent fluctuation of the plasmonic system and the inhomogeneous emission pattern (Fig. 1f, main text). Hence, an expansion of a region of the emissive region will not guarantee a flat phase for interference.

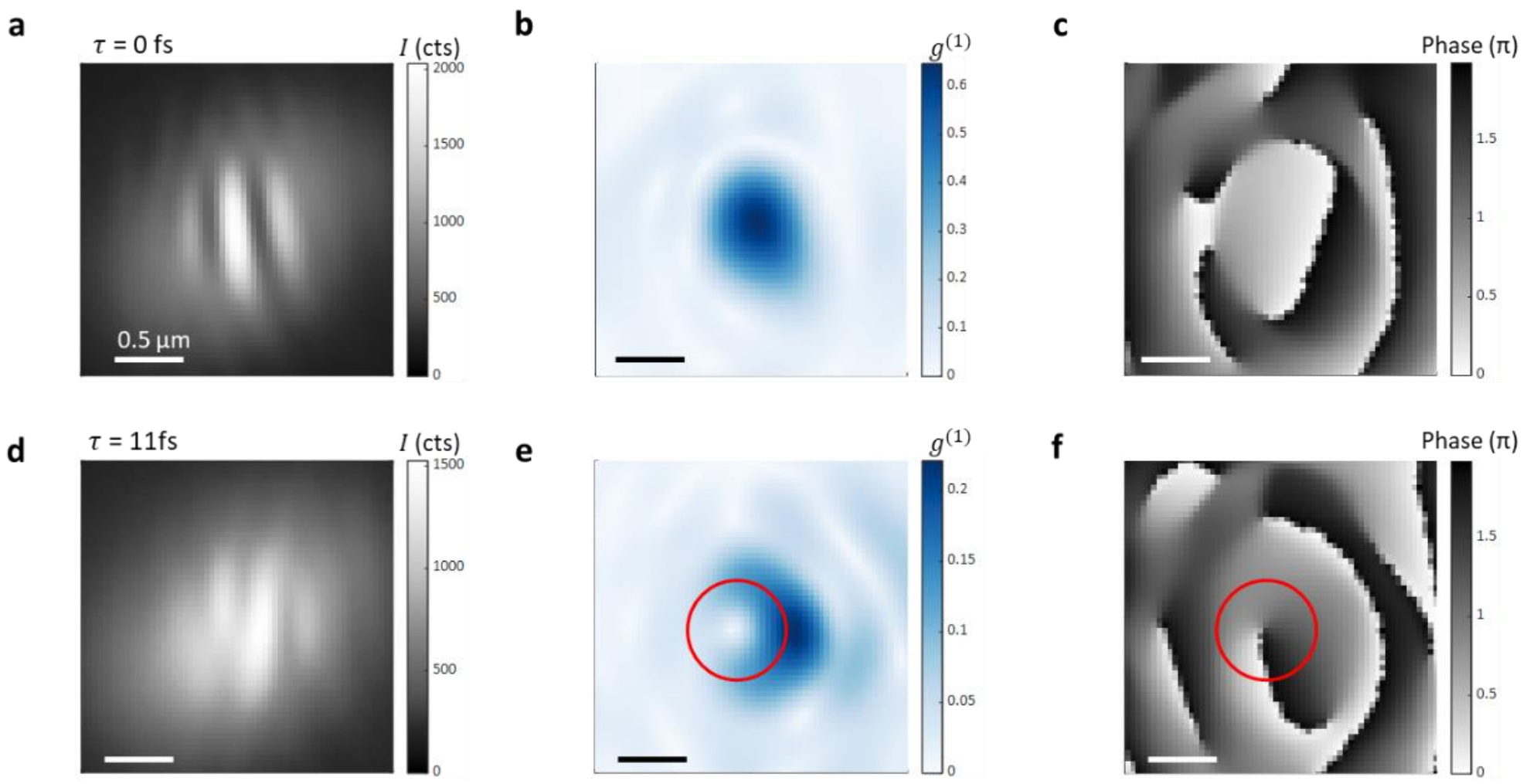

Supplementary Figure S30: **Phase retrieval for vortex formation in $g^{(1)}(R, -R, \tau)$ for MB:CB=1**. At zero time delay τ = 0 fs: **a**, Bare interferogram. **b**, Extracted $g^{(1)}(R, -R, \tau)$ using techniques in SI Figure S12. **c**, Extracted phase profile showing flat phase in the centre of the emission region. At time delay τ = 11 fs: **d**, Bare interferogram showing phase fork. **e**, Extracted $g^{(1)}(R, -R, \tau)$ showing vortex with null $g^{(1)}$ coherence circled red. **f**, Extracted phase profile showing vortex with phase winding around 2π.

Following the hydrodynamic description of quantum fluids [17, 19], the velocity field is defined as the gradient of the phase of the macroscopic wavefunction, $v_{\text{tot}}(R, \tau) = \frac{\hbar}{m} \nabla\phi(\text{R}, \tau)$. To ensure numerical stability and correct handling of topological phase singularities (2π wrapping) present in polariton vortices, the velocity is calculated using the complex form $v_{\text{tot}} = Im\{\psi^* \nabla\psi\}/|\psi|^2$, which preserves the rotational nature of the flow at the vortex cores (below, SI Figure S31a). The velocity field can be decomposed into an incompressible (solenoidal/divergence-free, v_{inc}) and compressible (longitudinal/curl-free, v_{com}) fields. These are extracted, first by applying the 2D-Fourier transform (2D-FFT) to $v(R, \tau)$ to form $v(k, \tau)$. As the 2D-FFT is periodic, we mitigate edge/finite boundary effects by subtracting the mean/bulk average velocity and applying a 2D Hann-window to smooth the velocity to zero at the boundaries. Subsequently, the incompressible field can be defined by projecting the Fourier coefficient into the direction perpendicular to the wavevector $k = [k_x, k_y]$ as $\hat{v}_{\text{inc}} = \hat{v}_{\text{tot}} - \hat{v}_{\text{com}}$ with $\hat{v}_{\text{com}} = k(k \cdot \hat{v}_{\text{tot}})/|k|^2$. The fields are then transformed back into real-space with an inverse 2D-FFT, and de-windowing then performed. This gives the solenoidal field showing swirls of incompressible flow and vortices (SI Figure S31b), and the longitudinal field showing flow from sources or sinks (SI Figure S31c). It is evident that there is a bona-fide vortex close to the centre of the image, while additional vortices (not observed in the interferogram) may be due to low signal-to-noise.

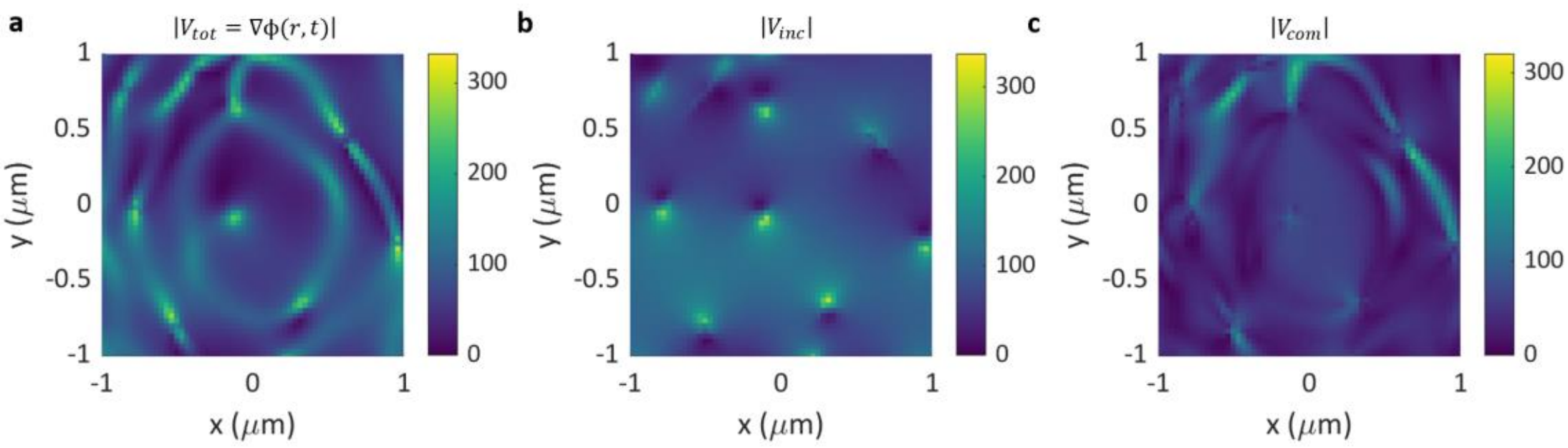

Supplementary Figure S31: **Flow analysis of phase velocity field**. **a**, Magnitude of the total phase velocity ($|v_{\text{tot}}|$) extracted from SI Figure S30f and its decomposition into the **b**, incompressible field ($|v_{\text{inc}}|$) and **c**, compressible field ($|v_{\text{com}}|$) components.

We now also show three additional interferogram images in the SI Section S7.1 with vortices forming and traversing the system, with corresponding space-time coherence plots to show variation across the sample plane at the origin, and propagation of these vortices.

8. Figure 1 compares below and above threshold behavior across different MB:CB ratios and clearly shows the emergence of the 'halo' with intensity increasing with Ξ. However, Fig. 1e and 1f do not state the corresponding excitation power they used. The author should label these figures with the corresponding pump power and link them unambiguously to the data points plotted in Fig. 1h. In addition, the arrows in Fig. 1h are not defined.
> The reviewer makes a good suggestion. We now include the pump powers used in the caption to Fig.1. The arrows mark the intersection of the initial fixed halo size with the back-projected nonlinear rise, for each dipole density/MB:CB ratio, as now noted in the caption.

9. Related to the former comment, if the blue arrow in Fig.1h denote the transition threshold, they appear offset by an order of magnitude from the threshold-like behavior shown in Fig. 2d (the evolution of $g(1)(R = 0)$. Please explain the origin of this offset and determine the transition threshold in this work.
> For this MB:CB ratio of 1, the arrow in Fig. 1h corresponds to $P\sim 6$ μW μm^{-2}, while $g^{(1)}$ in Fig. 2d begins to increase at $P\sim 3$ μW μm^{-2}. Given the width of this transition, as reproduced in our model, these are indeed comparable within experimental uncertainty and reflect different observables probing related but distinct aspects of the synchronization process.

In particular, local phase correlations (measured by $g^{(1)}$) increase before the spatial emission halo becomes evident. This behaviour is consistent with the theoretical results shown in Fig. 4c, where the spin coherence $|Z_n|$ grows prior to the spatial spreading of photon population N_{nn}. Thus, the apparent offset reflects the sequential emergence of local coherence followed by spatial expansion, rather than distinct transition points. We now clarify this in the revised manuscript.

Additionally, to further validate the phase transition, it would be helpful to demonstrate the real space emission and the corresponding interference fringe images under different pumping density from below and above the threshold.
> The real space images above and below threshold are now provided in the SI Fig.16, as suggested.

Reviewer #2:

1. Why do authors claim incoherent CW pumping? Aren't they using a laser?
> We clarify that although pumped with a CW laser (at 1.96eV), energy relaxation to the emitting state (at 0.19eV lower energy) through vibrational relaxation (via Kasha's rule) breaks all coherence in the emission. This is a key issue, which explains most other questions of the reviewer. This is also evidenced by the change in emission polarization compared to the pump laser, now added in new SI Figure S35. Referring to such a configuration as incoherent pumping is standard terminology within the microcavity polariton field, to distinguish from coherent pumping where one resonantly pumps the mode that is emitting. We thus amend the manuscript to state this clearly.

2. What is the role of the spatial coherence of the pump laser on the observed spatial coherence? i.e if you were to use a truly incoherent source like white light or an LED can spatial coherence be observed?

> The reviewer highlights a key aspect which should be clarified. Indeed the pump laser is a highly spatially coherent source, with spatially narrower and larger $g^{(1)}$ than the emission from our coupled nanogap arrays (SI Fig. S7b). However (see point 1) this coherence is not imparted onto the emission, whose coherence still spreads out further than the half-width of the laser pump (SI Fig. S7a). We now also include controls in SI Fig S15 (see below) showing the coherence of a brightfield white light source reflecting off a mirror in our setup, and show that it has much lower coherence, and spatially falls off faster than emission from the 1 MB:CB system.

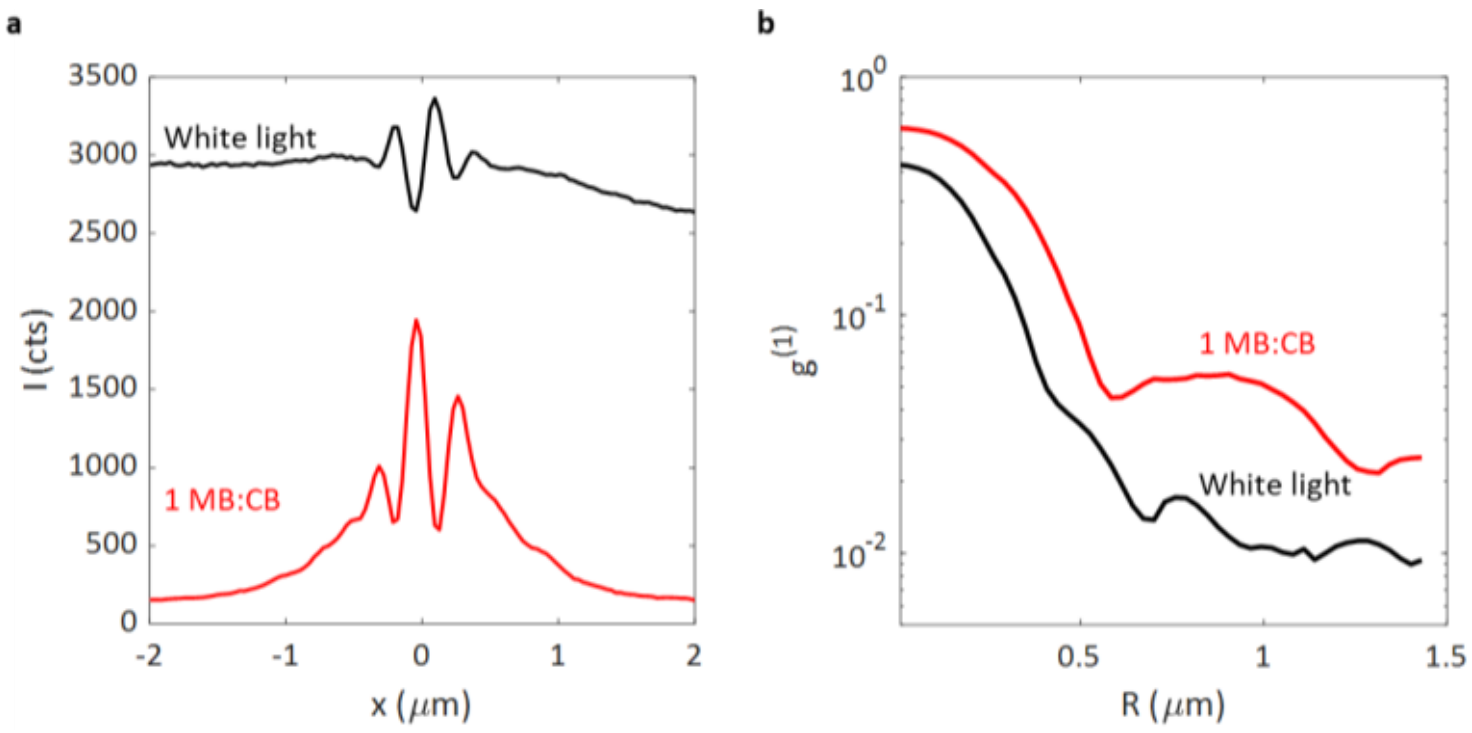

Supplementary Figure S15: **Interferograms for white light reflection off a mirror and MB:CB=1**. **a**, Interferogram for white light reflecting of a mirror, and from 1 MB:CB at pump power of 360 μWμm^{-2}. **b**, Extracted $g^{(1)}(R, \tau = 0)$ for the corresponding interferograms in (a).

3. Can one think of this observation as the result of short radiative lifetime and hence the emission doesn't have enough time to undergo decoherence? i.e essentially the photons retain the coherence information only because the lifetime of the emitter is so short in the plasmonic cavity.

> This is an interesting point. However, because the emission spectrum is similar to that from MB outside the plasmonic cavity (Fig.S23a), we do not believe it arises from rapid reradiation of the pump due to plasmonic speed-up, but instead from relaxation to the lowest excited state before reradiation (photoluminescence). This is confirmed by the growth of coherence with increasing pump power (Fig. 2d), which would not be expected in the reviewer's scenario. We thus clarify this in the main text.

4. Observation of coherence from molecular emitters coupled to plasmonic structures (without lasing or condensation) has been reported before (Phys. Rev. Lett. 108, 066401 (2012)).

> We thank the referee for bringing this paper (Guebrou *et al*) to our attention. We highlight several key issues and differences with our work:

- The interference observed in Guebrou *et al* (now [20]) likely originates from **spatial filtering of the emission in k-space** rather than being an intrinsic property of the material state. As described in their experimental setup (Fig. R1a), a 'beam block' was inserted in the Fourier plane to select a specific wave-vector direction (the k_{up} configuration). According to the Van Cittert–Zernike theorem [21, 22], restricting the angular range of wave-vectors in the far-field (k-space) fundamentally increases the spatial coherence length in the near-field (real-space). By filtering their emitted light, they effectively place a pinhole in k-space (Fig. R1a), which artificially restricts the range of wave-vectors able to interfere on their detector. This creates a filtered coherence that is indicative of the optical aperture used rather than the actual polaritonic or synchronized

state of the sample (Fig. R1b). In contrast, our measurements are performed without such k-space filtering, ensuring that the $g^{(1)}$ coherence we report is a genuine signature of the emerging driven-dissipative synchronization.

- Our spatial coherence emerges because of **power-dependent synchronization**, whereas k-space filtering would produce coherence that is independent of pump power. Guebrou *et al* do not report any nonlinear pump-power dependence of the spatial extent of the emission or total emission intensity. The formation of an emission halo is a novel indicator of transport mediated by dipole synchronization, and is not a feature of the linear strong-coupling regime described by the reference cited.
- Here we study the behaviour of **localized coupled nanogap systems** forming a collective state. This contrasts with emission from propagating surface plasmon polaritons which is fundamentally different. In standard polaritonic or laser systems (as Guebrou *et al* who operate in the strong coupling limit), spatial coherence is typically accompanied by temporal coherence (line narrowing). Our observation of spatial coherence *without* temporal coherence is a distinct signature of synchronization in a highly lossy environment (the ‘bad-cavity’ limit).
- We provide a **many-body theoretical treatment** of how synchronization spreads through a disordered nanogap lattice, which goes beyond the "independent emitter" vs "hybridized state" comparison used by Guebrou *et al*.

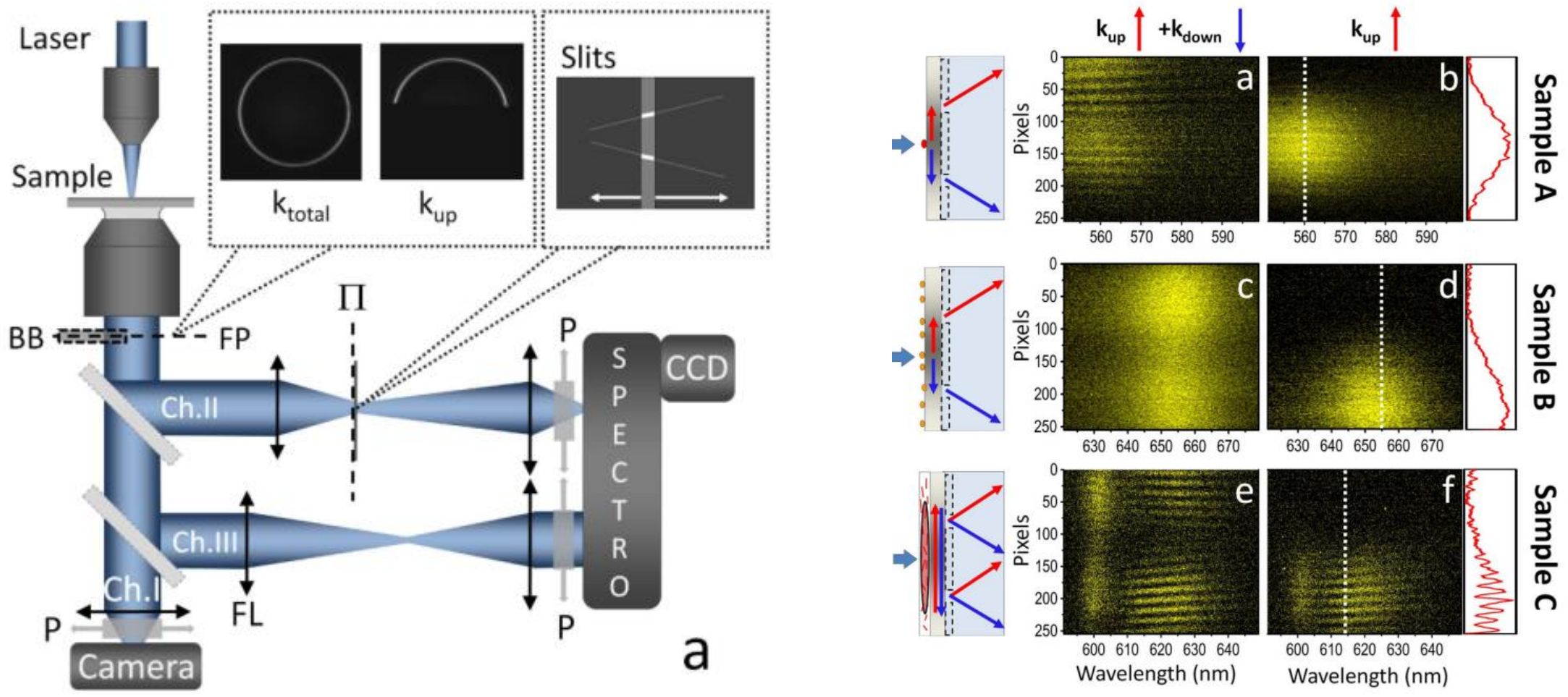

Fig. R1 Reproduced from Guebrou et. al. [20] Left: “FIG. 1 (a) Experimental setup. A direct imaging is performed on channel I. Channel II is devoted to coherence measurements by inserting slits in an intermediate image plane of the sample surface. Channel III is used for dispersion measurements, by imaging the Fourier plane of the microscope objective on the entrance slit of the spectrometer. A beam block (BB) can be inserted in the Fourier plane (FP) to select a given wave vector direction for all the channels.” Right: “FIG. 3. Interference pattern recorded for samples A, B, and C: without selection on the wave vector [panels (a), (c), and (e), respectively] and with only the upwards propagation [panels (b), (d), and (f), respectively]. Layouts of different propagation mechanisms are drawn on the left side of the figure for each of the samples. For the (k_{up}) configuration, the intensity profile measured along the white dotted line is drawn on the right side of the figure.”

Regardless, we cite this work as an example of how such systems have been studied for some time, but that self-assembly and strong light-matter coupling within the nanogaps now enables new synchronization phenomena to emerge.

5. How does the spatial coherence vary across the samples? The authors need to show this effect at least at a few different pump locations and show the variation across the sample.

> In the SI we now add additional spatial interferograms (Fig. S11), and highlight the repeatability of the phenomenon and its persistence despite the self-assembled and locally-ordered nature of the nanogap arrays. The new SI Figure S18 (see below) shows $g^{(1)}(R\text{=}0, \tau\text{=}0)$ plotted against pump power across 8 different locations on the sample, showing similar values to that of Fig. 2d. In each case, a similar nonlinear rise in coherence is observed. Images of $g^{(1)}(R, \tau\text{=}0)$ are also shown across the film at 8 different locations in SI Figure S17, at the maximum power.

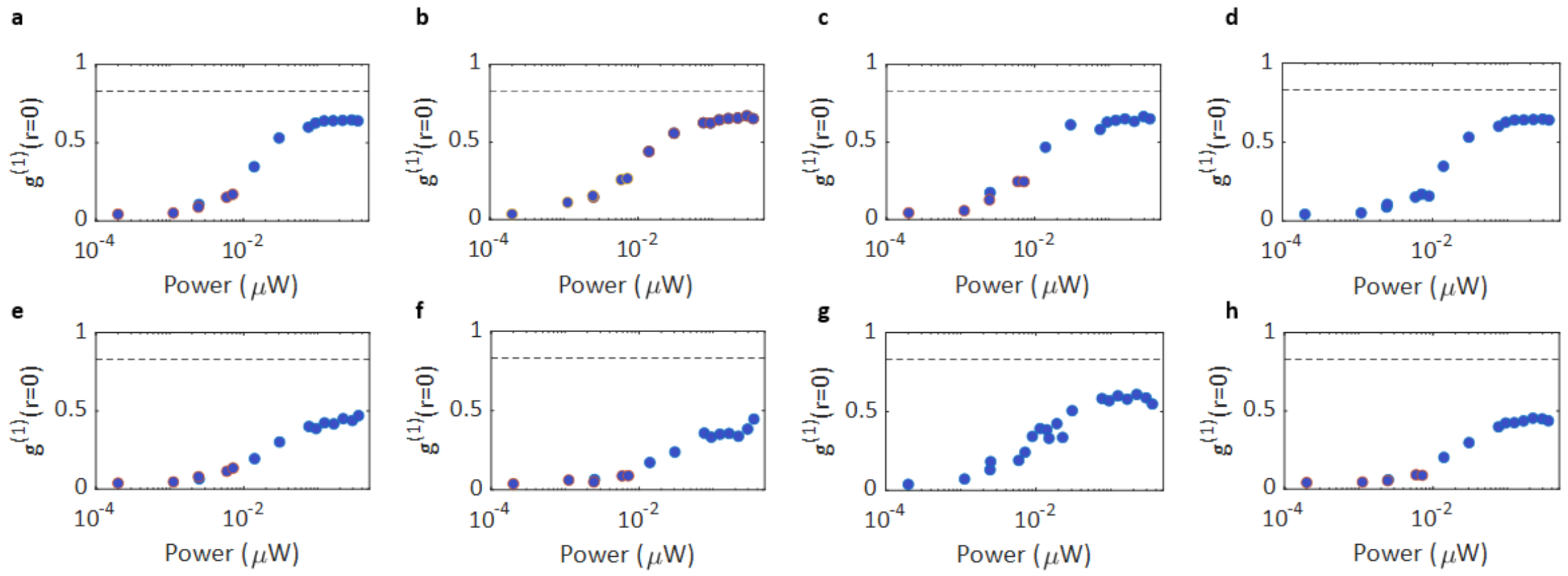

Supplementary Fig. S18: **Pump power dependence of $g^{(1)}(R\text{=}0, \tau\text{=}0)$ for MB:CB=1 for different positions a-h.**

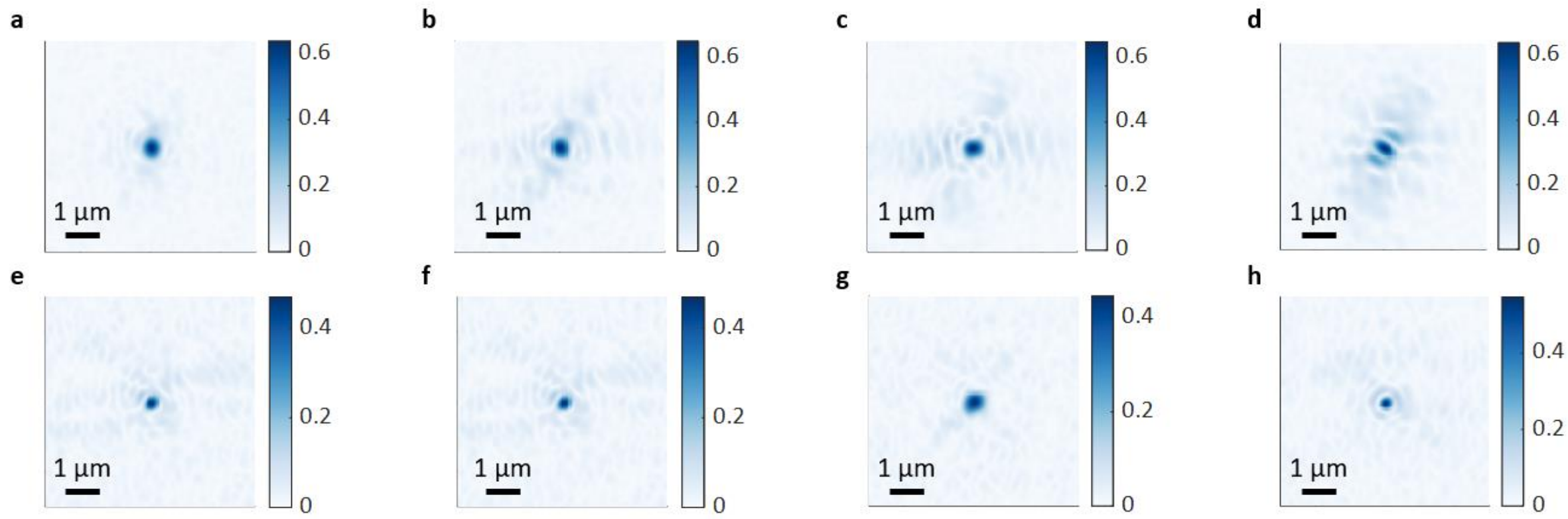

Supplementary Figure S17: **Interferograms for MB:CB=1 at different positions**. a-h at pump power of 357 Wm^{-2}

6. As per Fig. 1 h , I, it appears that the threshold is around 10^-2 mW for the MB:CB of 1 and almost no clear threshold behavior for the MB:CB of 0.01. However, in Fig. 2, where g1 is shown, the threshold where the coherence sets in seems to be 10^-3 mW. Also, what is the collection of datapoints in Fig. 2d around 10^-2 that does not lie near the theory graph?

> The arrow in Fig. 1h corresponds to $P\sim6$ μW μm^{-2}, while $g^{(1)}$ in Fig. 2d begins to increase at $P\sim3$ μW μm^{-2}. Given the width of this transition, as reproduced in our model, these are indeed comparable within experimental uncertainty and reflect different observables probing related but distinct aspects of the synchronization process. In particular, local phase correlations (measured by $g^{(1)}$) increase before the spatial emission halo becomes evident. This behaviour is consistent with the theoretical results shown in Fig. 4c, where the spin coherence $|Z_n|$ grows prior to the spatial spreading of photon population N_{nn}. Thus, the apparent offset reflects the sequential emergence of local coherence followed by spatial expansion, rather than distinct transition points. We now clarify this in the revised manuscript. We also believe the transition is not abrupt because it is a continuous evolution between linear and sublinear scaling regimes, as shown by our theory. This transition between the regimes is

discussed in the main text ("*Coherence spreading and dipole-correlations*" section) as the point where a spatial emission halo emerges and the phases between adjacent excitons synchronize.

As for the collection of points not lying exactly on the theory prediction, we include a new discussion in SI Section S3.8 detailing the limitations of our theoretical model. With such considerations, it is a remarkable that the full quantum theory matches the experiments so well, and we believe it captures the essential physics of the system.

7. The authors state the distinction of the present system to be absence of spectral narrowing and rapid decay of temporal coherence. I don't see any spectral data! And why would one want short temporal coherence?

> Emission spectra are shown in Fig. 3d and in SI Section S6, and discussed in the main text. We agree that the temporal coherence in Fig. 2 is short compared to photon or polariton condensates. However, long temporal coherence is not the relevant performance metric for the regime explored here, as we also answer to point 1 of reviewer #1. The MLagg platform operates in an extreme bad-cavity limit with ultrafast plasmonic decay, where long photon lifetimes are fundamentally inaccessible. In this context, short temporal coherence reflects rapid emission and strong light–matter coupling rather than a lack of collective behaviour. Indeed, fast radiative decay can be advantageous: it enables high repetition rates, improve practical performance by increasing emission rates [6] and photon indistinguishability [7], suppresses cavity-induced spectral pulling [4, 5], and allows collective phase synchronization to occur on timescales shorter than dissipation without requiring narrow-line operation. The key advance we have observed is the emergence of spatial phase order and synchronized emission in a highly dissipative nanophotonic environment, not the realization of long-lived cavity coherence. Hence, we have revised the introduction and discussion to clarify this distinction and to focus on concrete nanotechnology implications: the possibility of engineering spatially coherent emission patterns, nonlinear upconversion, and collective light-matter response in 2D systems where traditional cavity-based coherence is unattainable.

8. What is the polarization state of the light before and after synchronization? Does it depend on the polarization of the laser, and does it also become synchronized in space?

> We now performed measurements of the emission polarization (new SI Fig. S35, below). The polarization state does not change with pump power, even in the regime where synchronization occurs. We note that the polarization state is far weaker than the initial linearly-polarized pump, due to the 2D modes of coupled plasmonic nanogaps depolarizing the emission. Plasmonic chains and aggregates support both polarization components [23, 24] emitting light of different orientation to the pump laser. This further indicates that the emission is not simply scattered laser light.

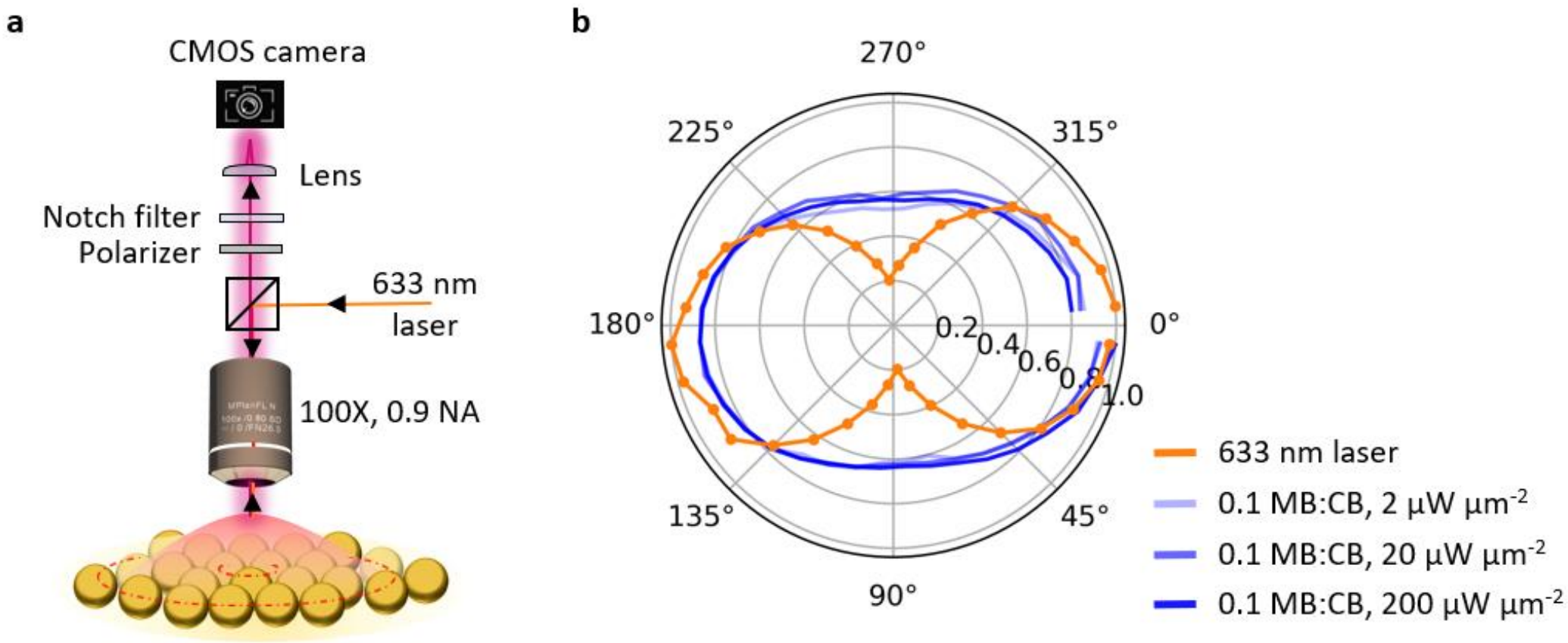

Supplementary Figure S35: **Emission polarization measurements**. **a**, Emission polarization setup, with wire-grid polarizer used to measure the emission polarization on a CMOS camera. **b**, Total normalized emission intensity as a function of polarizer angle for the pump laser control, and MB:CB=0.1 sample at three different pump intensities below/above synchronisation.

9. What happens under pulsed excitation? Can this system transition to conventional lasing in that regime? What about heating in the CW regime?

> Here we explicitly avoid pulsed excitation, as many works concentrated on this (while interesting, it is outside the scope of this work). We highlight the key advantage here is that it works with CW pumping, without needing ultrashort pulsed lasers to achieve high peak powers, because the plasmonic nanogaps provide optical field enhancement that yield synchronisation even with CW fields. This has been previously shown by our group (and others) to enable other nonlinear optical processes such as visible-midIR sum frequency generation with CW light sources [25-27]. The plasmonic nanogaps speed up radiative emission, so the excited state relaxes before bond disassociation, photochemical bleaching, or transfer to dark triplet states (and hence reaction with singlet oxygen). This has also been shown by other groups in recent work [28-30].

Heating may be an issue with CW measurements, however we use anti-Stokes electronic Raman scattering (ERS) thermometry, as in Refs. [31, 32], to show that the system is not significantly heated (new SI Fig. S27 and below). The anti-Stokes ERS spectrum (I_{ERS}) is fitted with a Boltzmann model $I_{ERS} \propto \exp(-\Delta E/k_B T)$ to extract an effective temperature (of the electrons in the plasmonic nanogaps).

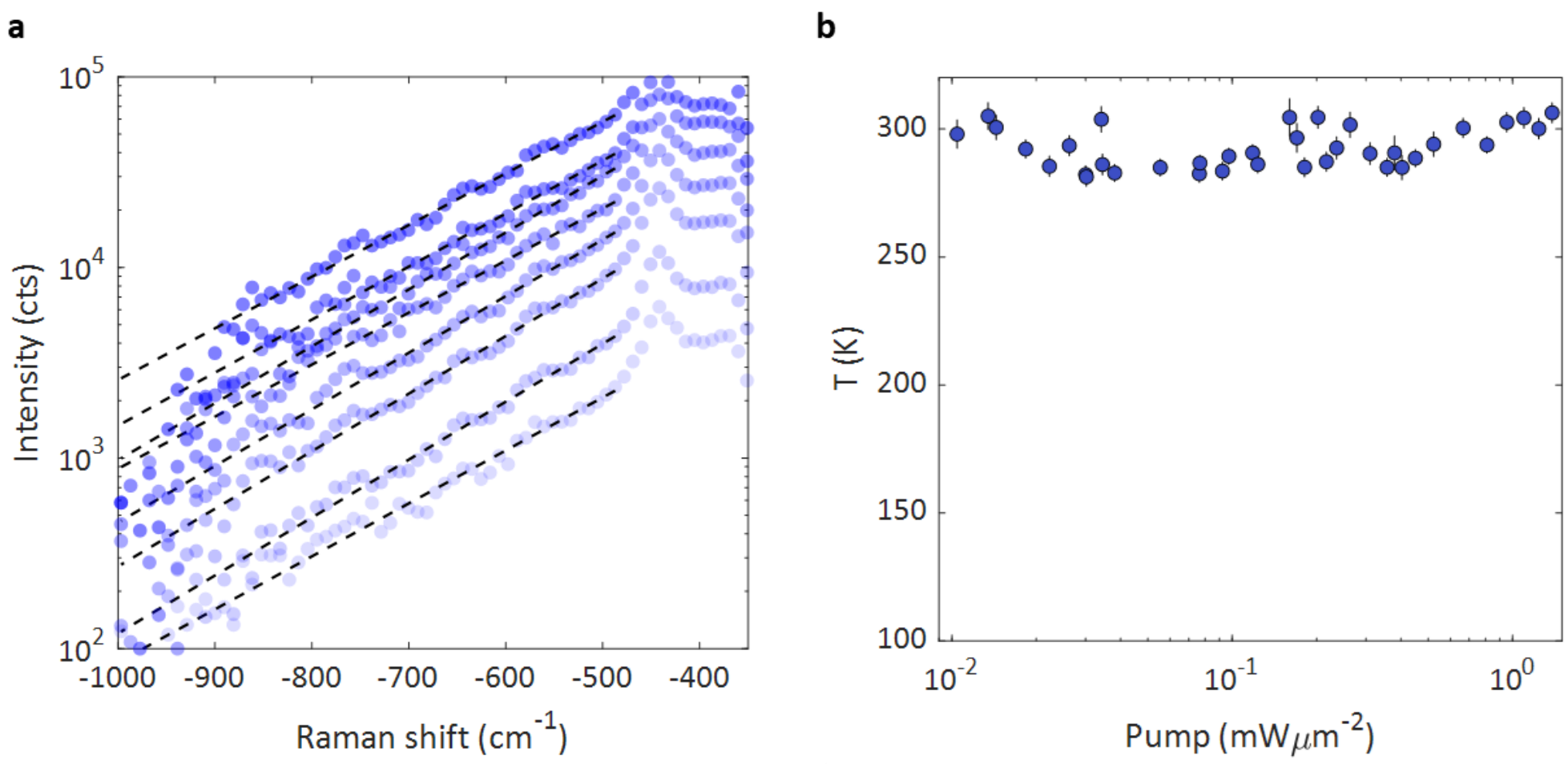

Supplementary Figure S27: **Estimated temperature from Raman**. **a**, Anti-Stokes electronic Raman scattering spectrum from MB:CB=1 over a range of powers from 0.01 to 2 mWμm^{-2}, dotted lines shows fits to Boltzmann tail extracting temperature. **b**, Extracted anti-Stokes electronic temperature *vs* pump power.

10. The authors mention "without directional emission" in abstract, but this statement is never supported in the manuscript.

> The reviewer helpfully catches our oversight. We now evidence this claim through the emission in k-space when imaging the total emitted light in the back-focal plane of the objective lens (new SI Fig. S36a). We show (new SI Fig. S36b,c) that the total emission intensity follows the same trend with pump power as before, and the k-space emission does not significantly change, with only a slight increase at higher wave-vectors/emission angles.

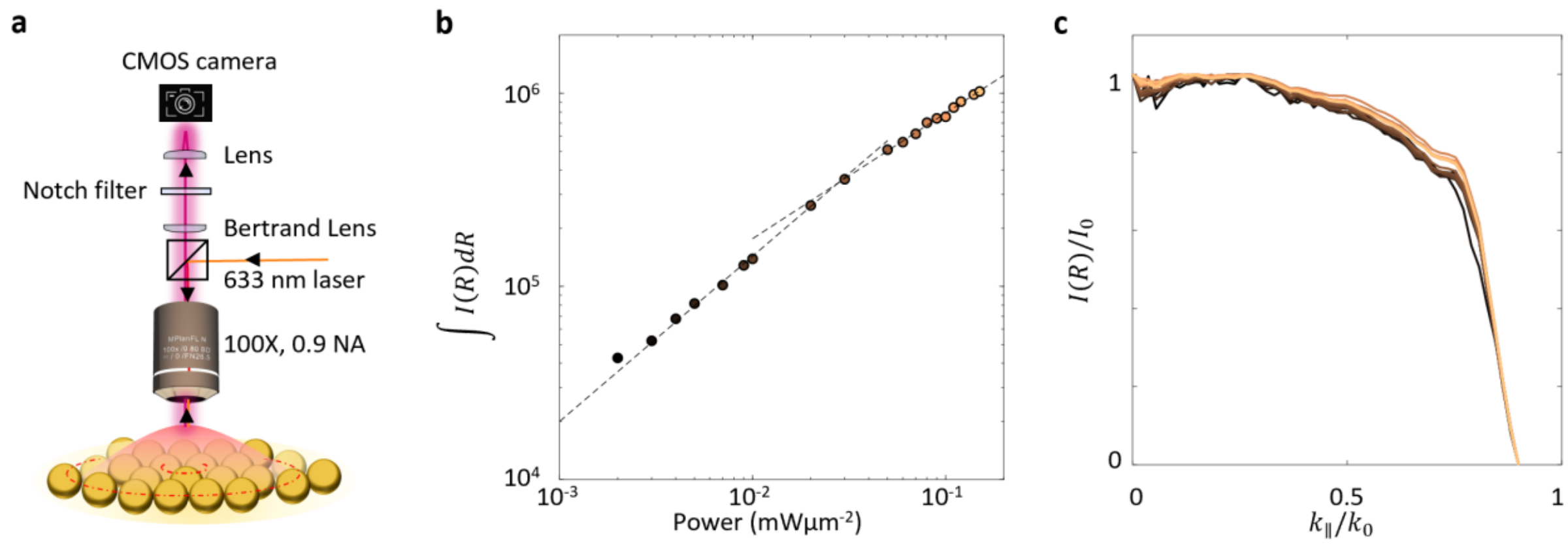

Supplementary Figure S36: **k-space emission measurements**. **a**, Emission k-space setup, where a k-space/Bertrand lens images the back-focal plane of the objective lens and the reciprocal/k-space information is imaged to a CMOS camera. **b**, Total k-space emission intensity pump power increases for MB:CB=0.1 samples averaged over 5 positions. **c**, Normalized k-space emission averaged over 5 positions for a range of powers with colors indicated in b.

11. Also, in the abstract the authors claim "scalable ambient operation." This requires clarification, what type of scalability do the authors have in mind?

> Scalability comes from collective phase order emerging in a self-assembled, room-temperature plasmonic network made from ubiquitous materials, without high-Q cavities, epitaxy, cryogenics, or vacuum infrastructure. This enables scalable architectures that are compatible with sensing and spectroscopy. We have previously demonstrated robust, large-area, self-assembled plasmonic networks ('MLaggs', see [1]) with reproducible nanoscale hotspots. These are transferable to sensing chips, coatings, and heterogeneous integration [2, 3]. This is the key nanotechnology advance: a soft matter, self-assembled platform showing synchronization of quantum emitters.

12. In Fig. 1e,f there are no color bars, and the central signal appears saturated. In Fig. 1h the caption is missing an explanation of what the arrows indicate. In addition, for the ratio of 0.1 the threshold is not clearly visible, why is this the case?

> This is a fair question. We add a colour bar in Fig.1e and show that the image is clearly not saturated below. Likely the reviewer is misled by our plotting on a logarithmic scale. We show now the full range on a linear-scale line profile in new SI Fig. S38a,b below. We also extract the normalized line profile on a linear scale, which shows that the image is not saturated (SI Fig. S38c,d, also below).

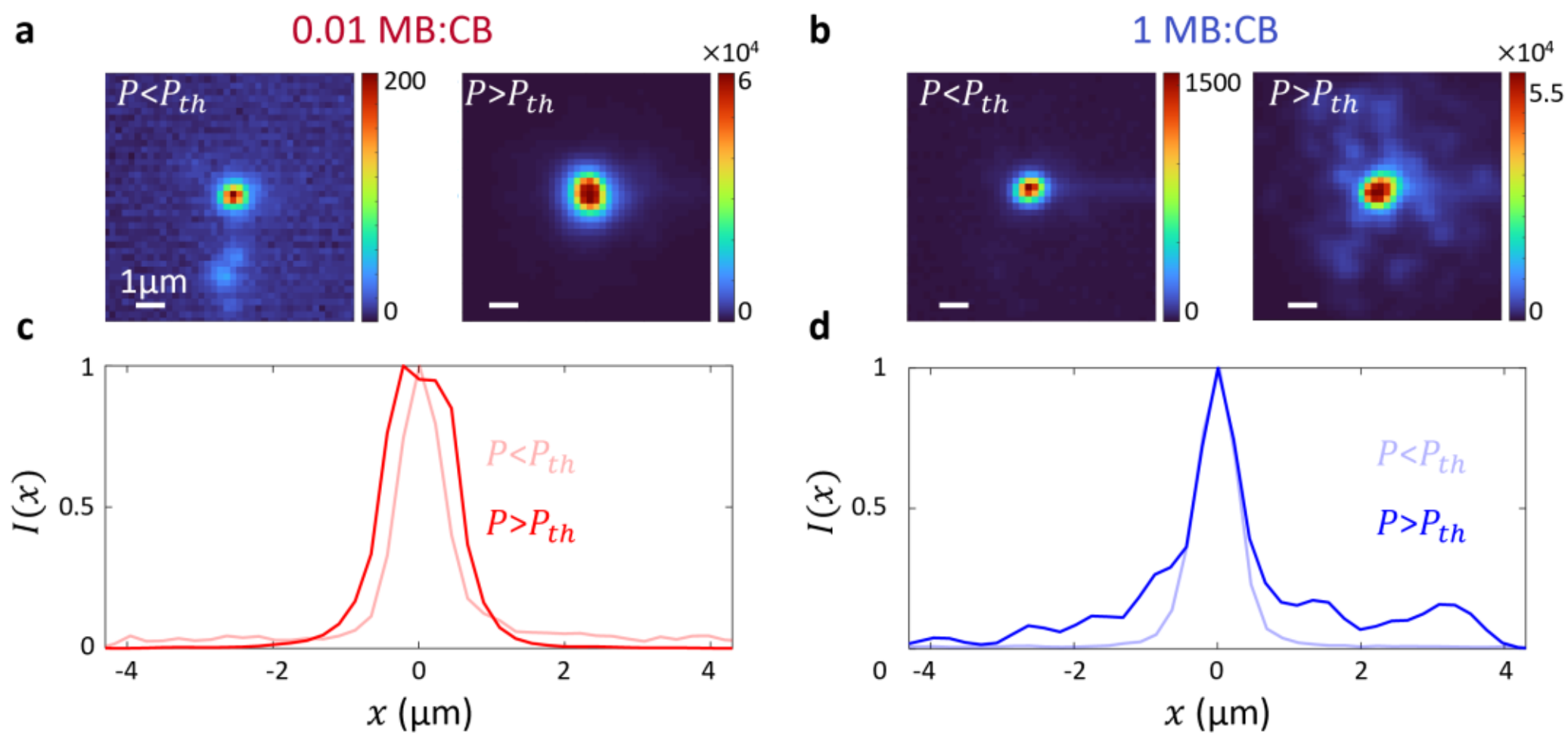

Supplementary Figure S38: **Line profiles for halo formation**. **a,b**, Imaged emission intensity maps below (P=2µW) and above (P=130µW) for (a) 0.01 and (b) 1 MB:CB ratios. All images share the same scale bar. **c,d**, Horizontal cross-sections of the images in (a,b) through their centres.

We now also clarify the origin of the arrows in the Fig. 1 caption: arrows mark the intersection of the initial fixed halo size with the back-projected nonlinear rise, for each dipole density/MB:CB ratio. While a specific transition point is less clear in the MB:CB=0.1 sample, as also shown in our model a continuous evolution towards synchronicity is observed. This likely is affected by the local arrangement of nanogap arrays, the varying density of emitters. Future work will address these questions.

13. Is strong focusing of the laser spot required for the synchronization to occur? Would it still appear for a larger spot, e.g., with a 2 um diameter, if provided power density is comparable? Then, how does the core/halo diameter scale?

> This is an excellent suggestion. A lower magnification objective (20X, 0.4 NA) results in a larger pump spot size of $\approx 0.61\lambda/NA$ = 970 nm and hence a pump intensity which is decreased by a factor of $(965/429)^2$ = 5.1x vs the more tightly focused 100X 0.9NA objective used previously. These new results are shown in the new SI Fig. S39 (below), comparing 100X excitation with 20X excitation. We cover some of the range of pump intensities where the total emission intensity becomes nonlinear, however this is limited as the laser source used has a maximum power of 50 mW. Regardless, we observe similar features of emission spatial spot size expansion, and a halo region which extends much further out to several tens of microns in diameter. The shape of the emission profile is slightly different to the 100X, which we attribute to the less tightly focused pump region, and the collection NA of the objective lens changing the image observed. This provides evidence that larger spatial synchronized regions can indeed be created by changing the pumping configurations, as previously in conventional polariton condensates observed by others [33, 34].

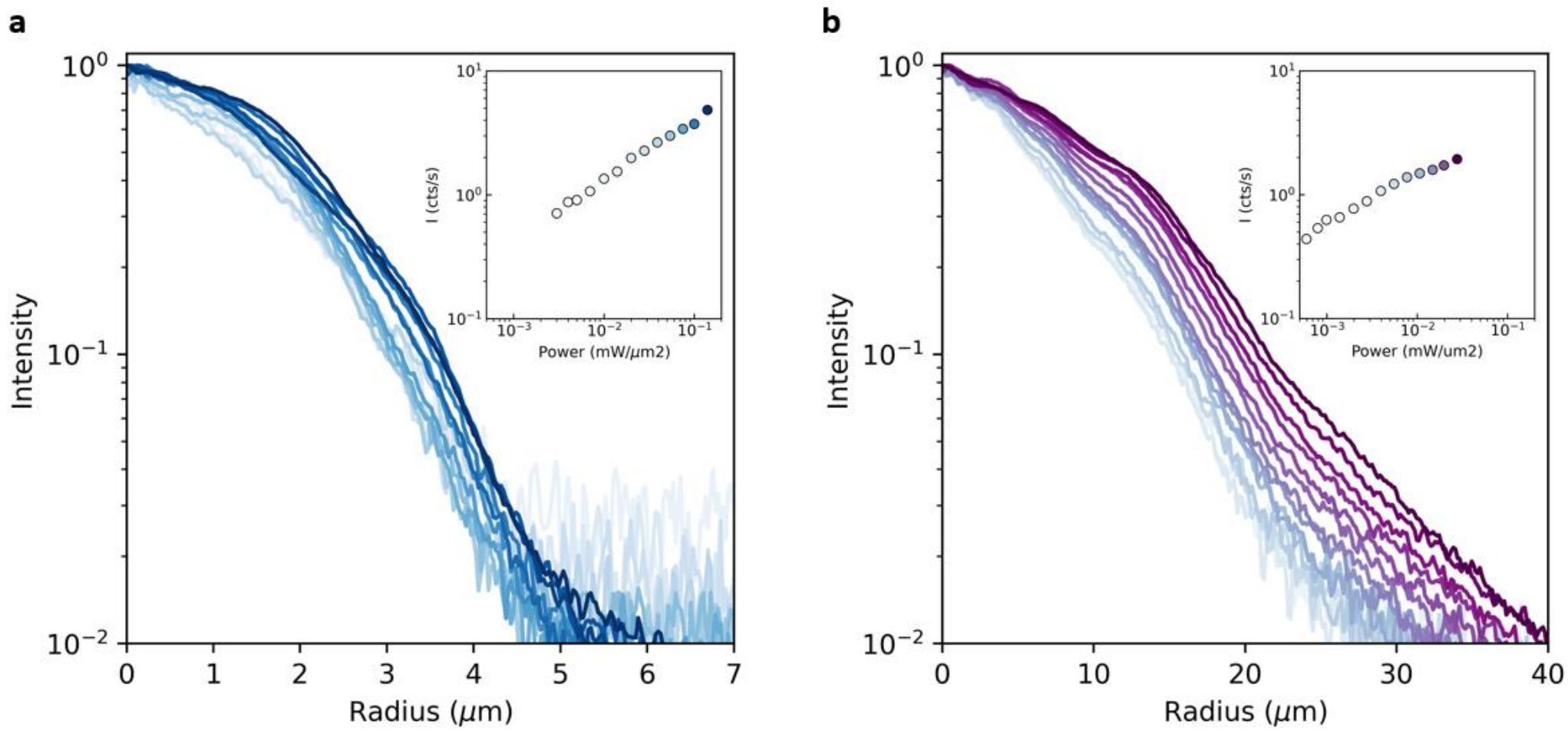

Supplementary Figure S39: **Measurements comparing x20 and x100 objectives**. **a**, Radially averaged normalized emission spatial pattern for MB:CB=0.1 over a range of pump powers with a 100X 0.9NA objective lens. **b**, Same as (a) with instead a 20X, 0.4NA objective lens. Insets indicate the total emission intensity vs pump power with colours corresponding to spatial profiles shown in the main plots.

14. What is the source of the revival of coherence observed in Fig. 2e?

> Again the reviewer raises an interesting question. The initial rapid decay of $g^{(1)}(R{=}0,\tau)$ reflects the ultrafast radiative decay associated with strong Purcell enhancement. We believe the subsequent collapse-revival-like oscillation arises primarily from spectral beating between distinct components of the emission spectrum.

In new SI Fig. S20 (below), fitting the emission spectrum with a broad bi-Gaussian model which captures the main electronic transition and its vibronic shoulder does not reproduce the observed oscillatory structure in $g^{(1)}(R{=}0,\tau)$ when Fourier transformed (Fig. S20d, black solid line). Only when enhanced resonance-Raman features are included in the spectral model does the Fourier transform yield an oscillatory behaviour consistent with the measured secondary rise (black dotted line). Spectral beating alone does not account for all features here, particularly the spatial vortex nucleation observed near the first minimum of $g^{(1)}$. As shown in SI Fig. S20b, phase mapping reveals the emergence of phase winding at this delay. We therefore interpret the collapse-revival feature as arising from the interplay of two effects: (i) temporal beating between spectral components and (ii) transient reconfiguration of spatial phase domains associated with vortex formation. We have clarified this interpretation in the revised manuscript and added the new analysis to the SI.

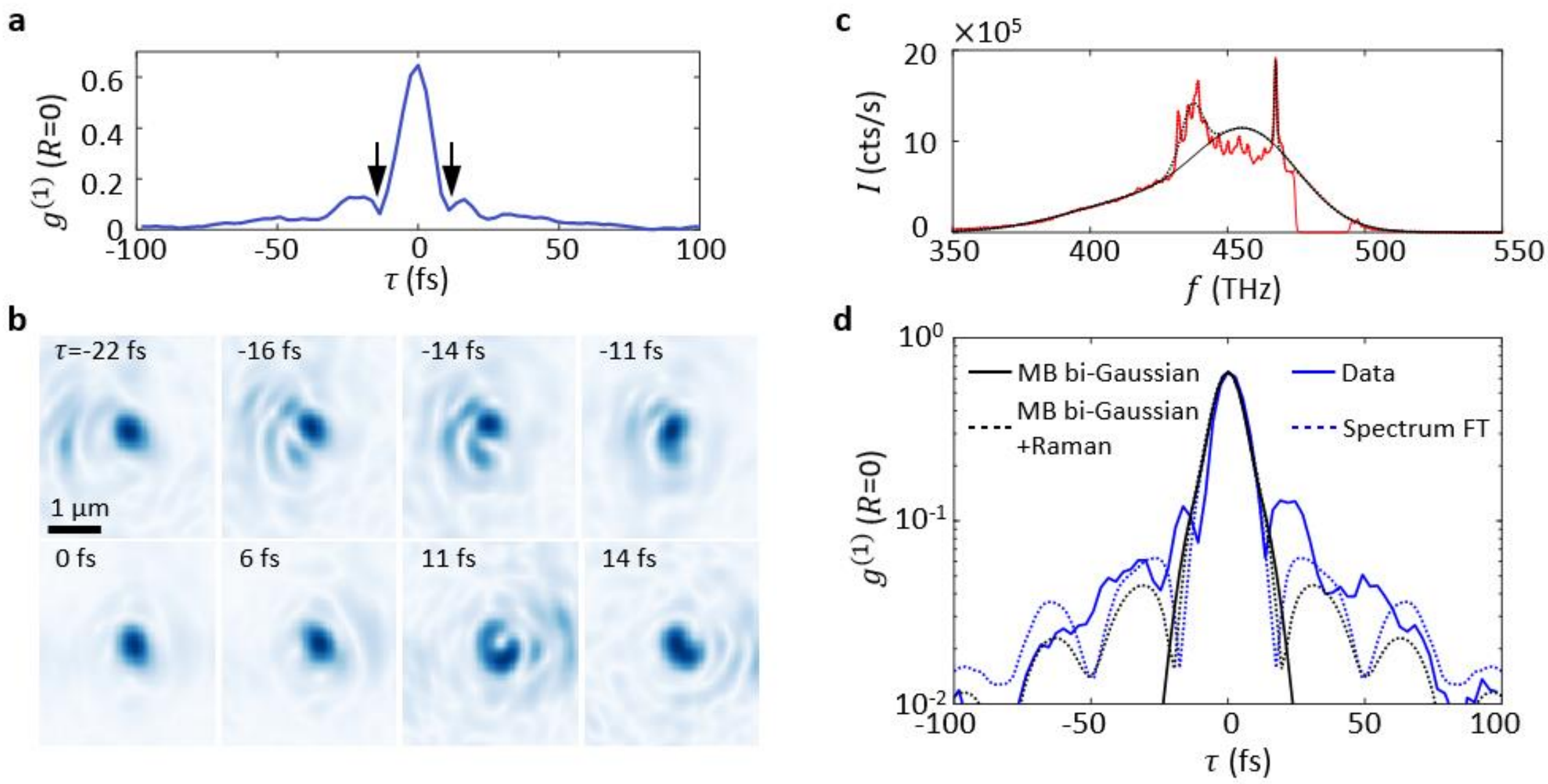

Supplementary Figure S20: **Collapse-revival analysis of $\mathbf{g^{(1)}}$**. **a**, Decay of $g^{(1)}(R$=0, $\tau)$ for MB:CB = 1 at pump power P=0.2 mW, with arrows indicating formation of vortices degrading the central $g^{(1)}$ at labelled interferometer time delay τ. **b**, Vortex phase dislocation in the $g^{(1)}(x,y,\tau)$ in space and time, corresponding to zero observed in $g^{(1)}(R=0,\tau)$ coherence trace in (a). All images share the same scale bar. **c**, Emission spectrum of MB:CB = 1 corresponding to (a), with solid black lines showing bi-Gaussian fit for MB main emission peak and vibronic side-band. Narrower Gaussian peaks show surface-enhanced resonant Raman peaks of MB. **d**, Decay of $g^{(1)}(R$=0, $\tau)$ compared to Fourier-transform of the fitted MB emission peak (MB bi-Gaussian), the fitted MB emission and resonance Raman (MB bi-Gaussian + Raman), the entire spectrum in c (Spectrum FT), and the observed data in a (Data).

15. In Fig. 3a, for a ratio of 0.01 the flattening appears very unconvincing. Moreover, the authors should explicitly refer to the SI Section 5 explaining how the in-coupled power is calculated.

> We show below in the new SI Fig. S22 that the flattening is only present for the MB:CB=1 and 0.1 samples, with low density MB:CB=0.01 samples showing not a sublinear but superlinear transition. We believe this is due to the saturation/flattening being due to pair annihilation processes, which are not possible in the limit where there is only one emitter per nanogap. Hence, the input-output relation calculated theoretically also shows a superlinear dependence and not a flattening (solid line in Fig. 3a of theoretical fit). This also explains why the MB:CB=0.01 sample deviates from the full scaling relation in Fig. 3c, which is obeyed in all other cases for MB:CB = 0.015, 0.02, 0.05, 0.1, 1. We also now make an explicit reference in the caption of Fig. 3 to the SI Section S5 which explains the calculation of in-coupled power, and add a discussion to the main text.

16. How are the thresholds estimated?

> We extract the threshold from the transition between two emission slopes (see new SI Figure S22). We show that the change in slope is not marginal, and clearly goes from a linear to a sublinear dependence (for MB:CB=1, 0.1) and superlinear dependence (MB:CB=0.01), and arrows mark the transition points below.

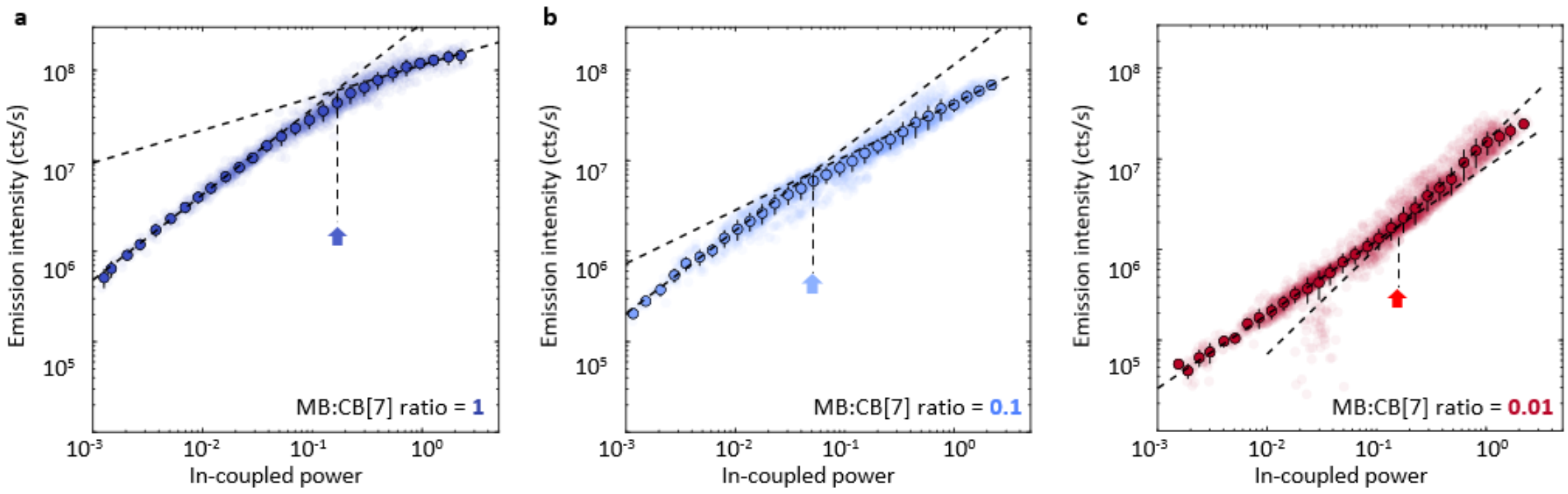

Supplementary Figure S22: **Input-output relation for nanocavity system**. Integrated intensity of MB emission *vs* in-coupled power for increasing dye concentration ($\propto P^{\alpha}$), showing deviations from linear scaling for different regime. **a**, MB:CB = 1, dotted lines show a transition from linear (α=0.95±0.02) to sublinear (α=0.36 ±0.1) power dependence. **b**, MB:CB = 0.1, transition from linear (α=0.92±0.1) to sublinear (α=0.59±.05) power dependence. **c**, MB:CB = 0.01, transition from near linear (α=0.81±0.1) to superlinear (α=1.2±0.1) power dependence. The crossover in-coupled power between the regimes shifts from 0.16, 0.05 and 0.03 for MB:CB = 1, 0.1, and 0.01 respectively.

17. What is the purpose of the Raman measurement? They point to the emergence of a new peak, but do not discuss what is the origin and how that has anything to do with the coherence in this system.

> The Raman measurements (SI Section S6) were performed primarily to identify the origin of the sharp spectral features emerging at higher pump powers and to distinguish them from possible lasing peaks. The analysis confirms that these features correspond to resonance-enhanced Raman transitions of the molecular emitter, rather than the onset of stimulated emission or cavity lasing. While the Raman peaks do not directly generate the spatial coherence observed, they contribute to the multi-component emission spectrum that influences the temporal structure of $g^1(\tau)$ through spectral beating. We have clarified this in the revised manuscript and placed the Raman analysis explicitly in the context of ruling out lasing and understanding the spectral contributions to the coherence dynamics. We also point to its implications for "optical pumping on phonon condensation/lasing (36), as well as optomechanical pumping of molecular vibrations (37)".

18. The purpose of the WSe2 experiments is not clear. Why is the WSe2 a control sample? Wont the excitons in the WSe2 couple via the plasmonic modes similar to the dye? And why is it compared to 0.01MB:Cb system and not the MB:CB = 1 or 0.1 system?

> The excitons in WSe2 do not couple similarly to the plasmonic modes, as there is just a single nanoparticle on top of a mirror, and not an extended plasmonic mode as in the rest of the experiments. This was intended as a control for the coherence expected for a single point scatterer/point source at the focus of the imaging setup. We have edited the SI to make this clearer. We also now show new control experiments measuring the coherence of a brightfield white light source reflecting of a mirror surface, see answer to point 2 above.

19. The authors talk about enhanced coherence in the conclusion. Is it really enhanced or it is just showing up in the photons emitted because of the short lifetime? And of so what use is the coherence if it is so short lived?

> It seems clear that as a function of pump power, the coherence is enhanced (Fig.2d, and the multiple repeats in point 5 above). We agree that the temporal coherence length is short compared to photon/polariton condensates or BEC systems, but we do not claim otherwise. Crucially, however, our

system operates in the bad-cavity deeply sub-wavelength plasmonic regime where long-range or long-lived coherence is fundamentally impossible due to ultrafast radiative and non-radiative decay. The significance of our observation is therefore not the magnitude of coherence times, but its existence under these conditions. We also discuss this in the response to reviewer #1 point 1.

Short temporal coherence and bad-cavity operation can be advantageous rather than detrimental: in bad-cavity/superradiant lasers, coherence is stored primarily in the emitters and cavity pulling is strongly suppressed, improving robustness against cavity noise for metrology [4, 5]. Likewise, Purcell-shortened lifetimes can improve practical performance by increasing emission rates [6] and by extracting photons on timescales shorter than slow environmental noise such as spectral wandering, improving photon indistinguishability [7].

We have developed a soft, self-assembled platform showing synchronization of quantum emitters where collective phase order emerges in a self-assembled, room-temperature plasmonic network made from ubiquitous materials, without high-Q cavities, epitaxy, cryogenics, or vacuum infrastructure. The field of mesoscopic physics of few quantum emitters is a developing one [8] and poised to push advances in physics and chemistry - we provide a new platform to realize this and to explore new polariton physics. Conventional microcavity polaritons already have a host of applications in switching [9], optical computing [10], to study nonequilibrium physics [11, 12], and to control chemistry [13]. Hence, we have revised the introduction and discussion to clarify this distinction and to focus on the nanotechnology implications: engineering spatially coherent emission patterns, nonlinear upconversion, and collective light-matter response in 2D systems where traditional cavity-based coherence is unattainable.

20. In the Methods section, the authors should explicitly state that the laser is continuous-wave and that the spot size is 0.5 um.

> We now include this information in the Methods, as suggested.

21. There are numerous small editorial errors, e.g., inconsistent spacing between citations, figure numbers, and units, also in SI.

> We endeavoured to correct all such errors as encouraged by the reviewer.

22. In the SI section Electromagnetic simulation there is a typo "nnaoparticle".

> We thank the reviewer for pointing this out and corrected it.

23. The results from SI Sections S7 and S8 are not discussed in the main text.

> We have now appropriately referred to SI Section S7 and S8 in the main text.

24. The most interesting data was the time resolved vortex formation (shown in Fig. 5). Does that alone warrant publication in Nature Nanotech? Finally the introduction and the discussion section of the paper are very well written making some interesting connections to other fields.

> We appreciate this reviewer finding Fig.5 most interesting. We thank them for their comments which have helped to strengthen the significance of our work. Our coherence observations cannot be due to simple fast re-emission, and thus in addition to our observation of vortex formation in the coherence, we believe the emergence of synchronization of emitters with power on timescales shorter than dissipation, without linewidth narrowing, directional emission, or lasing is a significant result.

Reviewer #3:

1. The short temporal coherence is seen as a feature that supports the synchronisation of the dipoles. This is counterintuitive to me. When a large number of dipoles synchronise, they form a macroscopic polarisation that should behave classical, i.e. have some robustness against fluctuations much in the same way as the phase of a coherent optical field. The absence of an increase of the temporal coherence time therefore seems to cast doubt on the true synchronisation of the dipoles.

> This is an astute point but identifies the clear novelty of our work. While in most systems, synchronization is accompanied by an increased temporal coherence, which results in a linewidth narrowing in the frequency response, we do not have linewidth narrowing. Instead, the spatial coherence $g^{(1)}$ increases to values comparable to other polariton condensate systems, while decaying within a short amount of time.

As our system operates in the bad-cavity and deeply sub-wavelength plasmonic regime, long-range or long-lived coherence is fundamentally impossible due to ultrafast radiative and non-radiative decay. The significance of our observation is therefore not the magnitude of coherence times, but its existence under these conditions. In contrast to condensates or lasers, where coherence emerges through mode occupation and spectral narrowing, we observe phase synchronization of emitters mediated by near-field plasmonic coupling on timescales shorter than dissipation, without linewidth narrowing, directional emission, or lasing. This demonstrates a qualitatively different route to optical phase order that is compatible with lossy, broadband, and nanoscale architectures. We change the main text to highlight this.

This is not without precedent, as similar synchronization phenomena occur in superradiant/ superfluorescent emission when coupled dipoles are excited with a pulse. The dipoles synchronize and emit light in a burst (Burnham-Chiao ringing [35, 36]), which is often accompanied by a linewidth broadening in the spectral domain.

2. In the abstract it is stated that there is a spread of g^(1) coherence without directional emission. This seems to contradict the relation between near and far field emission. When there is spatial coherence, the directional emission should be a direct consequence. I do not see how truly synchronised dipoles can emit non directed. The light emission should inherit the coherence properties of the dipoles, no?

> This is an excellent point. We thus perform new measurements of the k-space emission pattern (new SI Fig. S36 below) which shows only a subtle change in the k-space emission distribution at high-k. We attribute this to the structure of the nanogap array and the pumping geometry. We have a tightly focused pump laser which excites the system across a range of k-vectors from normal up to the maximum NA of the objective (as seen in SI Fig. S36c).

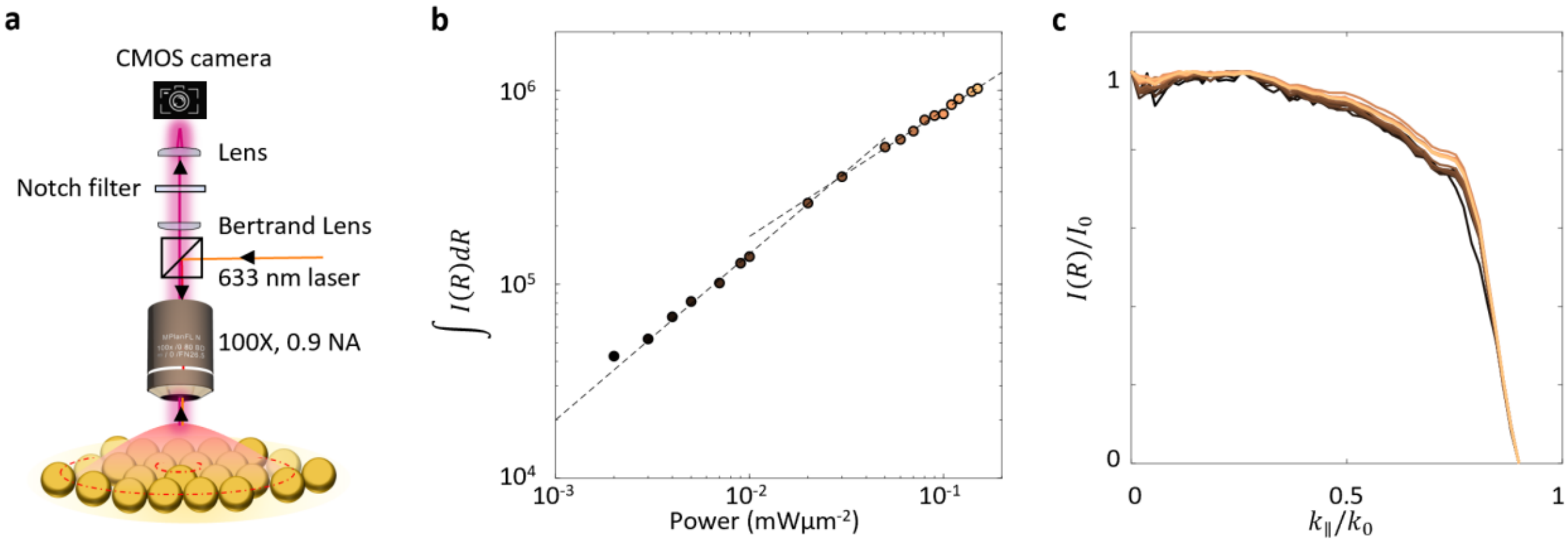

Supplementary Figure S36: **k-space emission measurements**. **a**, Emission k-space setup, where a k-space/Bertrand lens images the back-focal plane of the objective lens and the reciprocal/k-space information is imaged to a CMOS camera. **b**, Total k-space emission intensity pump power increases for MB:CB=0.1 samples averaged over 5 positions. **c**, Normalized k-space emission averaged over 5 positions for a range of powers with colors indicated in b.

Even when the excitation is at only normal incidence (by passing the pump laser through a Bertrand lens which focusses it on the back-focal plane of the objective lens, SI Fig. S37a), the emission pattern remains almost uniform angularly. The film composed of near-field coupled nanoparticle chains that scatter incident light field into a range of directions. There is a slight increase towards higher angles at higher pump powers (SI Fig. S37b). The combined emission pattern in SI Fig. S36c thus contains two contributions: the range of incident angles, and the scattered angular range of emitted light for each angle of incidence. This makes a clear observation of angular narrowing harder to see. By contrast, the $g^{(1)}$ measurement is independent of the angular range. It is a more sensitive probe, as it purely looks at field correlations between R and $-R$ about the image centre. Indeed, the literature has examples where an array of dipoles does not emit directionally even when driven into a superradiant state, eg. superfluorescence in Rb is omnidirectional due to variations in excitation angle [37].

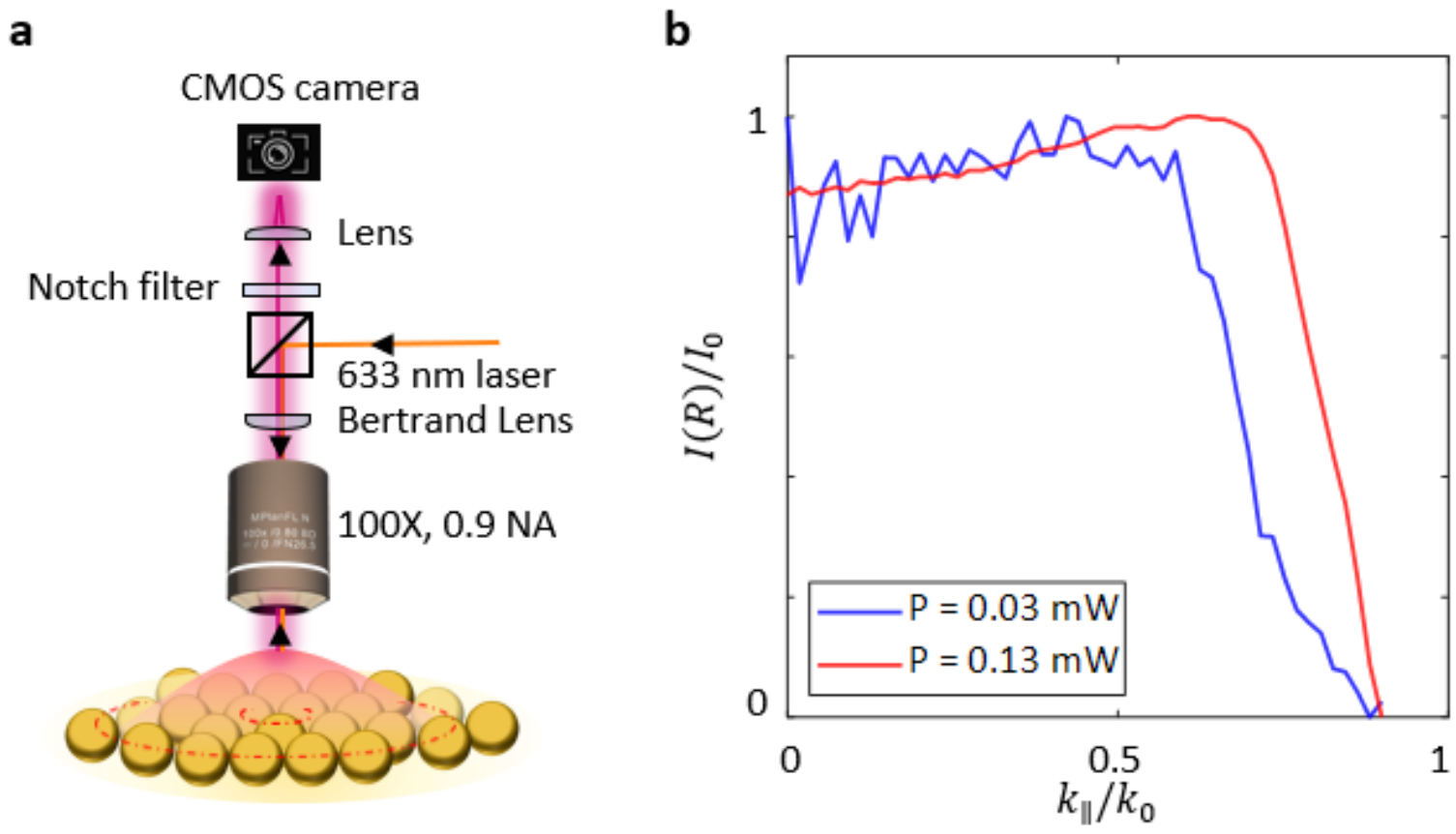

Supplementary Figure S37: **k-space emission measurements under normal incidence laser excitation**. **a**, Emission k-space setup, where a k-space/Bertrand lens images the back-focal plane of the objective lens and the reciprocal/k-space information is imaged onto a CMOS camera. The Bertrand lens is placed after the beamsplitter in-coupling the laser, hence laser illumination is now normal to the sample. **b**, Total k-space emission intensity as pump power increases for MB:CB=0.1 samples averaged over 5 positions for two different pump powers. Note the focal spot of the laser is restricted to normal incidence with the Bertrand lens, giving larger areas and reducing the intensity.

3. Fig. 2d shows g^(1)(R=0,tau=0), which by definition should always be equal to one, no? Such a strong variation rather casts doubt on the experiment than show a transition to a synchronised regime.

> We agree with the reviewer that this needs clarification. For an ideal single spatial mode, $g^{(1)}(R=0,\tau=0) = 1$, by definition. This is indeed the case in our theoretical model, where the correlation is evaluated for individual dipoles.

Experimentally, however, the Michelson measurement probes a diffraction-limited region (~500 nm) which contains multiple nanogap emitters (≈35 sites given the ~80 nm interparticle spacing). The measured $g^{(1)}(R=0,\tau=0)$ therefore reflects a spatial average over partially correlated emitters rather than the autocorrelation of a single dipole. When dipoles emit independently, this averaging reduces the measured visibility below unity. As phase synchronization develops, mutual coherence between emitters increases, and the measured $g^{(1)}(R=0,\tau=0)$ correspondingly rises. We have clarified this point in the SI (Section S3) and now explicitly discuss it in the main text to avoid ambiguity.

4. What is the physical origin of the pair annihilation mechanism in the model?

> The pair annihilation mechanism is exciton-exciton annihilation, which we now expand upon in the SI Section S3.6:

Pair annihilation processes: In SI Fig. S6 below, we show schematically that under strong laser illumination, two singlet excitons may be created in adjacent molecules within a nanogap. Under a high density of excitons, two excited state excitons in S_1 may non-radiatively annihilate to promote one exciton to the second excited state while de-exciting the first exciton. The resulting state then rapidly decays back down non-radiatively following Kasha's rule, prior to emission of a photon into the nanocavity field. This is a pair annihilation process as it takes two emissive dipoles/excitons, and via this higher order process only retains one, which re-emits a photon back into the cavity. This has been observed in many condensed phase organic molecules within crystals and thin films [38, 39], 2D materials [40], and optical cavities [41] have been shown to alter its behaviour. Within the highly dilute limit observed in our experiments, we expect that the plasmonic field is also assisting dipole-dipole interactions and hence increasing the strength of the pair annihilation rate Γ^{ee}, following the theoretical treatment by May et. al. [42].

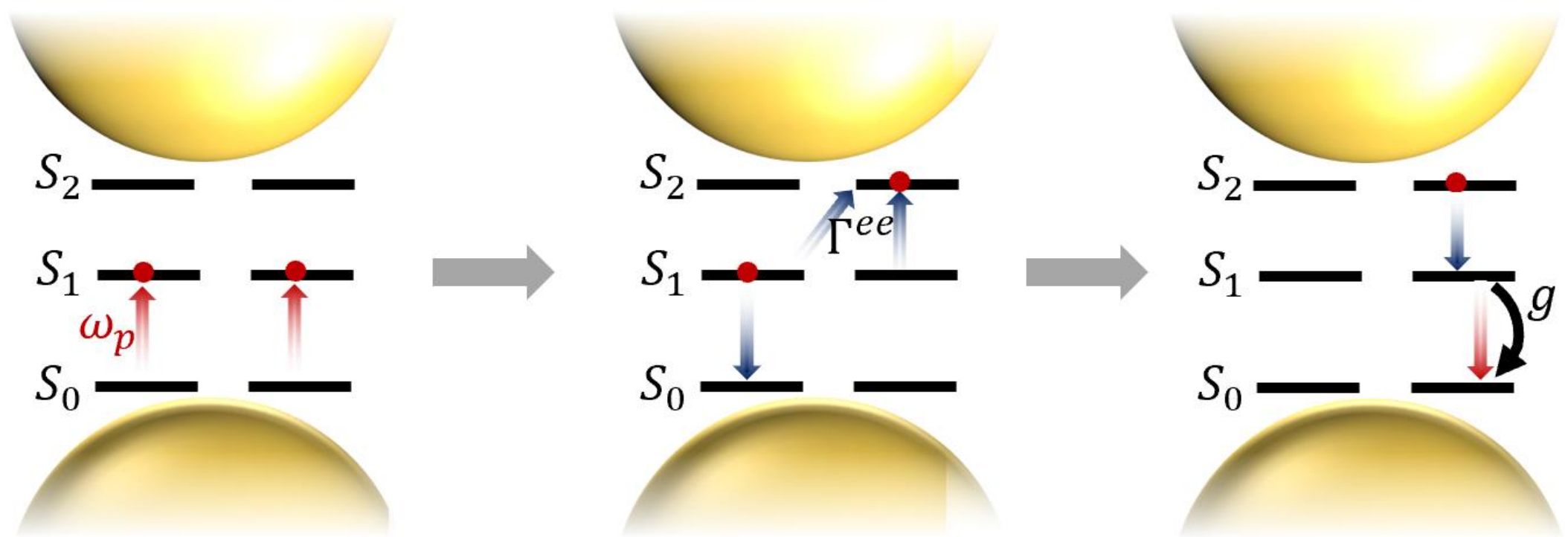

Supplementary Figure S6: **Exciton-exciton pair annihilation schematic within nanogap**. Initially, two adjacent molecules within a nanogap may be excited by an incident pump photon from the singlet exciton ground state S_0 to the first excited state S_1. Under high density of excitons, two S_1 excitons may non-radiatively annihilate with a rate Γ^{ee}, promoting one exciton to the second excited state S_2 while de-exciting the first one. The S_2 state then non-radiatively decays to S_1 prior to emission and coupling to the photonic field (coupling strength g).

5. In Fig. 2.c, the spatial coherence in the theory decays quickly in both regimes. Is there really a synchronisation transition in the theory?

> As shown in Fig. 1g&h halo growth and Fig. 2d $g^{(1)}$ increase, synchronisation increases above a threshold power. This is supported by the theory showing the evolution of emitter- emitter correlations increasing with pump power in a smooth evolution above a threshold, and the growth of photon number from the dipole synchronization in Fig. 4.

The spatial decay of the coherence observed is a function also of the pumping geometry, as we show in the new SI Fig. S39. Using a lower magnification objective (20X, 0.4 NA) results in a larger pump spot size of $\approx 0.61\lambda/NA$ = 970 nm. We observe similar features of emission spatial spot size expansion, and a halo region which extends much further out to several tens of microns in diameter. The shape of the emission profile is slightly different to the 100X, which we attribute to the less tightly focused pump region, and the collection NA of the objective lens changing the image observed. This provides evidence that larger spatial synchronized regions can indeed be created by changing the pumping configurations, as previously in conventional polariton condensates observed by others [40, 41].

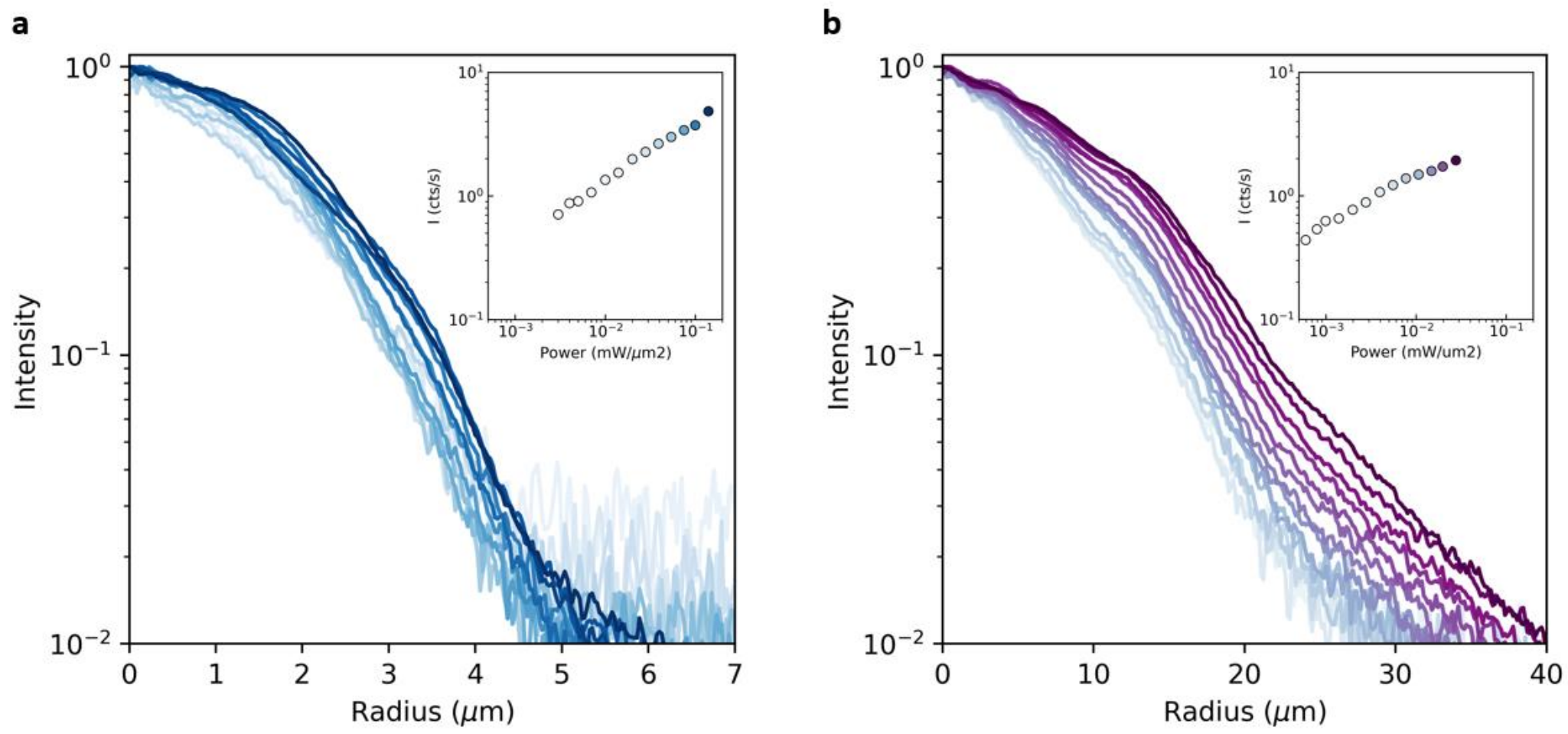

Supplementary Figure S39: **Measurements comparing x20 and x100 objectives**. **a**, Radially averaged normalized emission spatial pattern for MB:CB=0.1 over a range of pump powers with a 100X 0.9NA objective lens. **b**, Same as (a) with instead a 20X, 0.4NA objective lens. Insets indicate the total emission intensity vs pump power with colours corresponding to spatial profiles shown in the main plots.

6. line 230: You say: "Spatial coherence arises from dipoles synchronizing their phases through evanescent coupling and nonlinear dipole-nanocavity interactions." Can you point out which terms in the model represent this physics?

> The terms involving evanescent coupling between nanogap sites appears in the tight-binding model Hamiltonian (main text and SI equation 1) as:

$$-J\sum_{n}(a_{n+1}^{\dagger}a_n + H.c.) + g\sum_{n,x}(a_n\sigma_{nx}^{+}e^{-ikr_n} + H.c.)$$

where J is the hopping strength of photons from one nanocavity site to another, and g is the strength of the individual dipole-plasmon coupling, and the rest of the parameters are defined in the main text. The nature of the tight-binding model (see SI Section S3.4) is an effective nearest-neighbour hopping of photons between sites, ie. the field is short range (and lossy), and only coupling adjacent cavities. Dipoles also only interact with their local cavity field.

The nonlinear interactions involving pair annihilation arises as Markovian terms in the master equation (main text and SI equation 2) as:

$$\Gamma^{ee} \sum_{n,x,y} L\left[\sigma_{nx}^{-}\sigma_{ny}^{-}\right]$$

where the relevant parameter is the pair annihilation rate within each gap Γ^{ee}. However, we note that even for a linear Hamiltonian for a model including two-level systems, a mean-field approximation can produce nonlinear equations of motion [19]. We thank the referee for the opportunity to clarify this and now highlight it in the main text.

7. line 242: you say that the observed vortices are related to the Kibble-Zurek mechanism. That is rather for systems with a quench, no? And those vortices are spontaneous and should not be visible in time-averaged interferrograms.

> We agree that the vortices may not have a common origin to the Kibble-Zurek mechanism, and that most such spontaneous vortices are invisible in time-averaged interferograms, unless the vortices obey a well-defined path due to the local potential landscape as in other polariton studies, which are also time-averaged and not single shot experiments [17, 18, 43]. Additionally, work by Malpuech [44], Witkowska [45], Liew and Ostrovskaya [46] have argued that "the establishment of a steady state by nonresonant pumping in an initially empty system cannot be an adiabatic process and is therefore equivalent to a quenching of the parameters of the system, leading to the appearance of topological defects" [44]. This leaves some room for interpretation; however, we agree it is speculative. We also believe that the vortices observed need not form via the Kibble-Zurek mechanism, and instead may have other origins such as phase fluctuations in the Kardar Parisi Zhang universality class as observed by Fontaine & Bloch [47]. We thus remove these claims from the main text.

8. line 243: you write "The vortex with g(1)=0 traverses the centre R=0". What does this mean?

> In Fig. 5c, we show the vortex forming at the bottom left corner of the coherent region (white spot) at delay time τ=-14 fs and seeming to traverse across the centre at positive times out toward the top right by time delay τ=14 fs. We indicate this now with an arrow for the reviewer in Fig. R2 below.

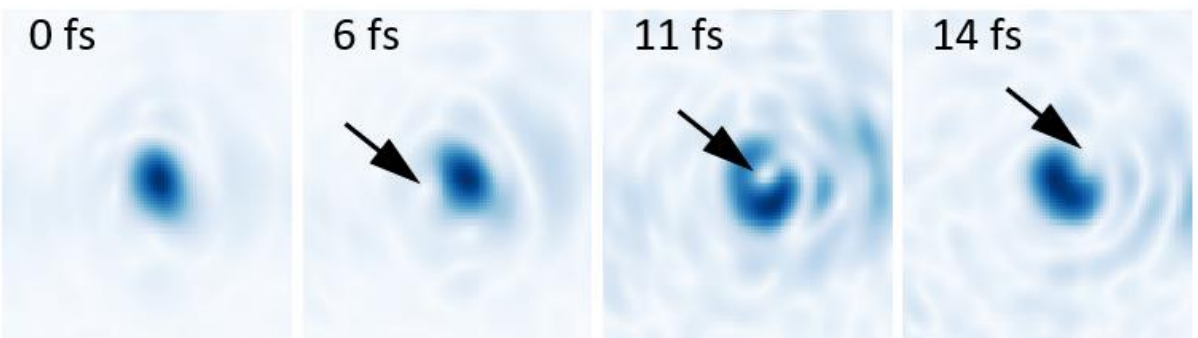

Figure R2: **Spatio-temporal vortex**. Vortex phase dislocation in the $g^{(1)}(x, y, \tau)$ in space and time. All images share the same scale, arrow marks position of vortex.

9. line 268 mentions Ref. (7). This reference is on interacting dipoles. It is not clear to me how this theory work supports the system that you are studying.

> This is a useful clarification. [7] develops a master-equation framework for synchronization in driven–dissipative arrays of interacting quantum dipoles subject to incoherent repumping and collective emission. In their model, elastic and inelastic dipole-dipole interactions, together with incoherent pumping, lead to a synchronized steady state exhibiting macroscopic phase coherence without lasing or cavity-mediated mode selection.

Our nanogap system contains the same fundamental ingredients: (i) two-level dipolar emitters, (ii) interactions mediated by electromagnetic coupling (in our case plasmon-enhanced near-field

interactions), (iii) collective radiative decay, and (iv) incoherent optical pumping. Although our implementation differs substantially, being strongly dissipative, plasmonic, and self-assembled rather than a cold-atom array, the underlying mechanism of synchronization in a driven-dissipative dipole ensemble is directly analogous. We have revised the manuscript to make this connection explicit and to clarify that Ref. (7) provides conceptual support for the synchronization mechanism rather than a direct quantitative model of our platform.

10. line 265: you write "constrained lateral coherence reflects phase diffusion as near-fields propagate outward from the core pumped region." Which phase diffuses here? Do you mean that there is diffusion of excitations due to the propagation?

> By 'phase diffusion' we refer to the diffusion of the collective dipole phase encoded in the spin-spin correlations $C_{nm,xy}= \langle \sigma_{nx}^{+} \sigma_{my}^{-} \rangle$ and $Z_n = \langle \sigma_n^z \sigma_n^z \rangle$, not to spatial diffusion of excitations or population transport. As shown in Fig. 4 and discussed in the section "Coherence spreading and dipole-correlations," the spatially resolved dipole–dipole coherence decreases with increasing separation from the pumped core. This reflects the outward propagation of phase correlations via near-field coupling, while dissipation and dephasing progressively degrade phase locking, resulting in a finite lateral coherence length. The population itself does not diffuse, it is the phase coherence between dipoles that decays with distance. We have clarified this distinction in the revised manuscript.

11. The connection to the cold atom reference (45) seems inappropriate. This work is about the phase fluctuations in a 1D thermalised system, which is very different from the driven-dissipative physics under study here.

> We are attempting to make a connection to fields where such driven phenomena are relevant but agree that a better connection to driven-dissipative atomic systems is more appropriate, hence now we cite instead: JK Bohnet, et al. *Nature* **484**, 78 (2012). We note that Reviewer #2 appreciates that we "make some interesting connections to other fields".

12. In general, it is my impression that the authors have sacrificed clarity for trying to highlight the great impact of the work, using technical terms not very carefully. It is from the current discussion actually not clear to me how much synchronisation is claimed. For example in lines 275, they write that there is "localised coherence". What does this imply for the length scale of the synchronisation of the dipoles. Is it only between neighbouring cavities? Do they mean that there are short-lived patches of dipoles that synchronise? Does this correspond to the "synchronised" state in the usual sense? I would rather say that it corresponds to a desynchronised regime in the usual meaning of the word. In conclusion, I think that the paper needs a careful rewriting, making sure that technical terms are used appropriately and that the claims are made precise. I actually doubt that there is very good synchronisation in this system.

> The reviewer raises an important point regarding terminology. We do not claim global phase locking across the entire nanogap array. Instead, our data demonstrate finite-range dipole phase synchronization, characterized by a non-zero dipole-dipole correlation $C_{nm,xy}$ that extends over multiple coupled cavities but decays with spatial separation.

As shown in Fig. 4 and in the section "Coherence spreading and dipole-correlations," the correlation length increases with pump power and spans several adjacent cavities but does not extend across the entire sample. Thus, the system exhibits spatially localized regions of phase locking coexisting with less

correlated regions. In this sense, the observed behaviour is more closely related to chimera-like states in driven-dissipative oscillator networks, where synchronized and desynchronized domains coexist, rather than to global synchronization in the Kuramoto mean-field limit [48]. We have revised the manuscript to clarify that by "localized coherence" we refer to finite-range phase synchronization characterized by a measurable correlation length, rather than global phase locking.

We disagree that there is no synchronisation in this system, and show below how in various systems where synchronisation and condensation is claimed, that the spatial coherence $g^{(1)}$ and the temporal coherence times are comparable to our system. This is a more detailed version of the summary table shown in SI Table S2. Our system is however different in that the spatial coherence arises on a very fast timescale and correspondingly decoheres quickly as well.

System	$g^{(1)}$(R=0,=0)	Spatial coherence length	Temporal coherence length	Coherence mechanism	Ref.
**Extreme bad-cavity**: Plasmonic nanocavity dipole synchronization	0.7	1-10 µm	30 fs	Near-field-mediated dipole phase alignment + driven-dissipative dynamics	This work
**Bad cavity**: Superfluorescence in perovskite nanolattices	0.6	2-3 µm	140 fs		[36]
**Polaritonic good cavity-limit:**					
GaAs quantum well microcavity	0.94	100 µm	100 ps		[49]
	0.8	100 µm	100 ps		[47]
	0.6	10-100 µm	280 ps		[50]
	0.6-0.9	10-35 µm	50-210 ps		[51]
	0.8	10-50 µm	100 ps		[52]
	0.9	10-100 µm	29 ps		[53]
	0.9	10 µm	2-4 ps		[54]
	0.9-1.0	2-3 µm	30 ps		[55]
Organic microcavity polariton	0.7	1 µm	156 ps		[56]
	0.7	10 µm	0.81 ps		[57]
	0.7	10 µm	30-230 ps		[58]
	0.25	20 µm	2 ps		[59]
Plasmonic lattice organic dye	0.3	100 µm	0.3 ps		[60]
	0.7	120 µm	1.7 ps		[61]
Polariton condensate (BIC polariton, WS_2)	0.3	4.9 µm	2 ps from linewidth	Condensation/stimulated scattering into a mode	[62]
Polariton condensate (WSe_2)	0.25	8 um, pump size 3 µm	4.5 ps		[63]
	0.5	5.6 µm trap size	4.8 ps	Trap and condensation	[64]
Photon gas / BEC in dye microcavity		7-22 µm	129 fs	Thermalization & Bose statistics; long-range	[65]

				order emerges as $N \rightarrow N_c$	
	0.6	-	10 ps		[66]
Atomic BECs	0.5-0.7	>0.7 µm	-	Equilibrium off-diagonal long-range order below T_c (phase coherence)	[67]

References:

1. Sibug-Torres, S.M., et al., *In situ electrochemical regeneration of nanogap hotspots for continuously reusable ultrathin SERS sensors.* Nature Communications, 2024. **15**(1): p. 2022.
2. Arul, R., et al., *Giant mid-IR resonant coupling to molecular vibrations in sub-nm gaps of plasmonic multilayer metafilms.* Light: Science & Applications, 2022. **11**(1): p. 281.
3. Grys, D.-B., et al., *Controlling Atomic-Scale Restructuring and Cleaning of Gold Nanogap Multilayers for Surface-Enhanced Raman Scattering Sensing.* ACS Sensors, 2023. **8**(7): p. 2879-2888.
4. Zhang, J., et al., *An extremely bad-cavity laser.* npj Quantum Information, 2024. **10**(1): p. 87.
5. Shi, T., D. Pan, and J. Chen, *Realization of phase locking in good-bad-cavity active optical clock.* Optics Express, 2019. **27**(16): p. 22040-22052.
6. Hoang, T.B., G.M. Akselrod, and M.H. Mikkelsen, *Ultrafast room-temperature single photon emission from quantum dots coupled to plasmonic nanocavities.* Nano letters, 2016. **16**(1): p. 270-275.
7. Liu, F., et al., *High Purcell factor generation of indistinguishable on-chip single photons.* Nature Nanotechnology, 2018. **13**(9): p. 835-840.
8. Bloch, J., et al., *Strongly correlated electron–photon systems.* Nature, 2022. **606**(7912): p. 41-48.
9. Zasedatelev, A.V., et al., *A room-temperature organic polariton transistor.* Nature Photonics, 2019. **13**(6): p. 378-383.
10. Zasedatelev, A.V., et al., *Single-photon nonlinearity at room temperature.* Nature, 2021. **597**(7877): p. 493-497.
11. Helluin, F., et al., *Phase diagram and universal scaling regimes of two-dimensional exciton–polariton Bose–Einstein condensates.* Physical Review Research, 2025. **7**(3): p. 033103.
12. Comaron, P., et al., *Dynamics of onsager vortex clustering in decaying turbulent polariton quantum fluids.* Physical Review Research, 2025. **7**(2): p. L022006.
13. Rashidi, K., et al., *Efficient and tunable photochemical charge transfer via long-lived bloch surface wave polaritons.* Nature Nanotechnology, 2025. **20**(11): p. 1618-1624.
14. Spiesshofer, N., et al., *Tailoring ultrahigh index plasmonic combinatorial metamaterials for SEIRA and SERS by tuning the fill fraction.* Optica, 2025. **12**(9): p. 1357-1366.
15. Taylor, R.W., et al., *Precise subnanometer plasmonic junctions for SERS within gold nanoparticle assemblies using cucurbit [n] uril "glue".* ACS Nano, 2011. **5**(5): p. 3878-3887.
16. Chikkaraddy, R., et al., *Single-molecule strong coupling at room temperature in plasmonic nanocavities.* Nature, 2016. **535**(7610): p. 127-130.
17. Panico, R., et al., *Onset of vortex clustering and inverse energy cascade in dissipative quantum fluids.* Nature Photonics, 2023. **17**(5): p. 451-456.

18. Lagoudakis, K.G., et al., *Quantized vortices in an exciton–polariton condensate.* Nature Physics, 2008. **4**(9): p. 706-710.
19. Carusotto, I. and C. Ciuti, *Quantum fluids of light.* Reviews of Modern Physics, 2013. **85**(1): p. 299-366.
20. Aberra Guebrou, S., et al., *Coherent Emission from a Disordered Organic Semiconductor Induced by Strong Coupling with Surface Plasmons.* Physical Review Letters, 2012. **108**(6): p. 066401.
21. Born, M. and E. Wolf, *Principles of optics: electromagnetic theory of propagation, interference and diffraction of light*. 2013: Elsevier.
22. Hyde IV, M.W., *Controlling the spatial coherence of an optical source using a spatial filter.* Applied Sciences, 2018. **8**(9): p. 1465.
23. Carnegie, C., et al., *Mapping SERS in CB: Au plasmonic nanoaggregates.* ACS Photonics, 2017. **4**(11): p. 2681-2686.
24. Esteban, R., et al., *How chain plasmons govern the optical response in strongly interacting self-assembled metallic clusters of nanoparticles.* Langmuir, 2012. **28**(24): p. 8881-8890.
25. Xomalis, A., et al., *Detecting mid-infrared light by molecular frequency upconversion in dual-wavelength nanoantennas.* Science, 2021. **374**(6572): p. 1268-1271.
26. Chikkaraddy, R., et al., *Single-molecule mid-infrared spectroscopy and detection through vibrationally assisted luminescence.* Nature Photonics, 2023. **17**(10): p. 865-871.
27. Xie, Z., et al., *Continuous-wave, high-resolution, ultra-broadband mid-infrared nonlinear spectroscopy with tunable plasmonic nanocavities.* arXiv preprint arXiv:2508.12097, 2025.
28. Munkhbat, B., et al., *Suppression of photo-oxidation of organic chromophores by strong coupling to plasmonic nanoantennas.* Science Advances, 2018. **4**(7): p. eaas9552.
29. Kéna-Cohen, S., et al., *Plasmonic sinks for the selective removal of long-lived states.* ACS Nano, 2011. **5**(12): p. 9958-9965.
30. Cang, H., et al., *Giant suppression of photobleaching for single molecule detection via the Purcell effect.* Nano Letters, 2013. **13**(12): p. 5949-5953.
31. Lee, A., et al., *Hot Electrons in a Steady State: Interband vs Intraband Excitation of Plasmonic Gold.* ACS nano, 2024. **18**(29): p. 19077-19085.
32. Boehmke, A., et al., *Tracking Molecular Shear at Metal Surfaces Using Enhanced Lamb Wave Scattering in Plasmonic Nanocavities.* Nano Letters, 2025. **25**(44): p. 15817-15823.
33. Berger, B., et al., *Formation dynamics of exciton-polariton vortices created by nonresonant annular pumping.* Physical Review B, 2020. **101**(24): p. 245309.
34. Wang, Y., P.G. Lagoudakis, and H. Sigurdsson, *Enhanced coupling between ballistic exciton-polariton condensates through tailored pumping.* Physical Review B, 2022. **106**(24): p. 245304.
35. Burnham, D.C. and R.Y. Chiao, *Coherent resonance fluorescence excited by short light pulses.* Physical Review, 1969. **188**(2): p. 667.
36. Rainò, G., et al., *Superfluorescence from lead halide perovskite quantum dot superlattices.* Nature, 2018. **563**(7733): p. 671-675.
37. Lvovsky, A., S. Hartmann, and F. Moshary, *Omnidirectional superfluorescence.* Physical Review Letters, 1999. **82**(22): p. 4420.

38. Mikhnenko, O.V., P.W. Blom, and T.-Q. Nguyen, *Exciton diffusion in organic semiconductors.* Energy & Environmental Science, 2015. **8**(7): p. 1867-1888.
39. Kumar, S., et al., *Exciton annihilation in molecular aggregates suppressed through quantum interference.* Nature Chemistry, 2023. **15**(8): p. 1118-1126.
40. Uddin, S.Z., et al., *Enhanced neutral exciton diffusion in monolayer WS2 by exciton–exciton annihilation.* ACS nano, 2022. **16**(5): p. 8005-8011.
41. Sortino, L., et al., *Radiative suppression of exciton–exciton annihilation in a two-dimensional semiconductor.* Light: Science & Applications, 2023. **12**(1): p. 202.
42. May, V., *Kinetic theory of exciton–exciton annihilation.* The Journal of Chemical Physics, 2014. **140**(5).
43. Lagoudakis, K.G., et al., *Probing the dynamics of spontaneous quantum vortices in polariton superfluids.* Physical Review Letters, 2011. **106**(11): p. 115301.
44. Solnyshkov, D.D., A.V. Nalitov, and G. Malpuech, *Kibble-Zurek mechanism in topologically nontrivial zigzag chains of polariton micropillars.* Physical Review Letters, 2016. **116**(4): p. 046402.
45. Matuszewski, M. and E. Witkowska, *Universality in nonequilibrium condensation of exciton-polaritons.* Physical Review B, 2014. **89**(15): p. 155318.
46. Liew, T.C.H., et al., *Instability-induced formation and nonequilibrium dynamics of phase defects in polariton condensates.* Physical Review B, 2015. **91**(8): p. 085413.
47. Fontaine, Q., et al., *Kardar–Parisi–Zhang universality in a one-dimensional polariton condensate.* Nature, 2022. **608**(7924): p. 687-691.
48. Strogatz, S.H., *From Kuramoto to Crawford: exploring the onset of synchronization in populations of coupled oscillators.* Physica D: Nonlinear Phenomena, 2000. **143**(1-4): p. 1-20.
49. Comaron, P., et al., *Coherence of a non-equilibrium polariton condensate across the interaction-mediated phase transition.* Communications Physics, 2025. **8**(1): p. 94.
50. Spano, R., et al., *Coherence properties of exciton polariton OPO condensates in one and two dimensions.* New Journal of Physics, 2012. **14**(7): p. 075018.
51. Baboux, F., et al., *Unstable and stable regimes of polariton condensation.* Optica, 2018. **5**(10): p. 1163-1170.
52. Caputo, D., et al., *Topological order and thermal equilibrium in polariton condensates.* Nature Materials, 2018. **17**(2): p. 145-151.
53. Alnatah, H., et al., *Coherence measurements of polaritons in thermal equilibrium reveal a power law for two-dimensional condensates.* Science Advances, 2024. **10**(18): p. eadk6960.
54. Roumpos, G., et al., *Power-law decay of the spatial correlation function in exciton-polariton condensates.* Proceedings of the National Academy of Sciences, 2012. **109**(17): p. 6467-6472.
55. Orfanakis, K., et al., *Ultralong temporal coherence in optically trapped exciton-polariton condensates.* Physical Review B, 2021. **103**(23): p. 235313.
56. Betzold, S., et al., *Coherence and interaction in confined room-temperature polariton condensates with Frenkel excitons.* ACS Photonics, 2019. **7**(2): p. 384-392.
57. Daskalakis, K., S. Maier, and S. Kéna-Cohen, *Spatial Coherence and Stability in a Disordered Organic Polariton Condensate.* Physical Review Letters, 2015. **115**(3): p. 035301.
58. Daskalakis, K., et al., *Nonlinear interactions in an organic polariton condensate.* Nature Materials, 2014. **13**(3): p. 271.

59. Plumhof, J.D., et al., *Room-temperature Bose–Einstein condensation of cavity exciton–polaritons in a polymer.* Nature Materials, 2014. **13**(3): p. 247.
60. Väkeväinen, A.I., et al., *Sub-picosecond thermalization dynamics in condensation of strongly coupled lattice plasmons.* Nature Communications, 2020. **11**(1): p. 3139.
61. De Giorgi, M., et al., *Interaction and coherence of a plasmon–exciton polariton condensate.* Acs Photonics, 2018. **5**(9): p. 3666-3672.
62. Sung, J., et al., *Polariton condensate far-detuned from exciton resonance in WS2 bound states in the continuum.* Nature Communications, 2025.
63. Shan, H., et al., *Spatial coherence of room-temperature monolayer WSe2 exciton-polaritons in a trap.* Nature communications, 2021. **12**(1): p. 6406.
64. Luo, Y., et al., *Manipulating nonlinear exciton polaritons in an atomically-thin semiconductor with artificial potential landscapes.* Light: Science & Applications, 2023. **12**(1): p. 220.
65. Damm, T., et al., *First-order spatial coherence measurements in a thermalized two-dimensional photonic quantum gas.* Nature Communications, 2017. **8**(1): p. 158.
66. Walker, B.T., et al., *Driven-dissipative non-equilibrium Bose–Einstein condensation of less than ten photons.* Nature Physics, 2018. **14**(12): p. 1173-1177.
67. Bloch, I., T.W. Hänsch, and T. Esslinger, *Measurement of the spatial coherence of a trapped Bose gas at the phase transition.* Nature, 2000. **403**(6766): p. 166-170.

Response to reviewers:

We thank all reviewers for their comments in improving the clarity and impact of this manuscript, especially the clarifying comments from Reviewer 2, and the recommendations respectively from Reviewers 1 and 3 that '*in the current state and with the extended discussions, the paper is now sufficiently sound for publication in Nature Nanotechnology since it can stimulate further experimental and theoretical research in this direction*' and that they '*recommend its publication in its present form*'. We discuss the further queries of Reviewer 2, and simplify the manuscript to the minimum that accounts for the phenomena.

Reviewer #1:

I have carefully reviewed the response and revised version. The authors have made improvements and addressed my earlier concerns in the revised version. I would recommend its publication in its present form.

> We thank the reviewer for their comments and suggestions for improvements.

Reviewer #2:

I have read the rebuttal by the authors to the comments raised by the three reviewers. I must confess that I am now even more confused about the claim of synchronization!

The paper and the rebuttal is packed with jargons and as the Reviewer 3 rightly points out: "the authors have sacrificed clarity for trying to highlight the great impact of the work, using technical terms not very carefully".

> We have endeavoured to simplify and clarify of our manuscript and believe our edited manuscript (which has satisfied Reviewer 3 after the previous revisions), now explains all jargon and clarifies the synchronization we discuss.

Synchronization has a well-defined meaning in the context of interacting dipoles, which we principally observe as the increase in the emission $g^{(1)}(R$=0,τ=0) coherence with pump power and the growth of the halo region. This is the key piece of evidence. We also use the established theoretical framework developed by Ana Maria Rey's group in Ref [7] of the main text. In our work, synchronization is quantified through inter-site and spin-spin correlations that signal the spontaneous emergence of a fixed phase relationship (phase-locking) among dipoles:

- **Inter-site Emitter Correlations ($C_{nm,xy}$):** defined as $C_{nm,xy}$= $\langle \sigma^{+}_{nx} \sigma^{-}_{my} \rangle$ represent the mutual coherence between dipoles at different spatial locations (x, y). The onset of synchronization is marked by these correlations growing and spreading spatially, which is directly observed as an increase in the **first-order spatial coherence $g^{(1)}$** recorded via Michelson interferometry.
- **Order parameter Z_n:** Synchronization is also shown in Ref [7] by growth in the average pairwise spin-spin correlations, denoted there as Z_Q. We use a related quantity, the local emitter coherence Z_n to show that phase-locking builds up across sites before photon correlations (N_{nm}) spread spatially into the halo (main text Fig. 4c,d). We explicitly show Z_n increases with pump power, and heralds the transition through off-diagonal terms appearing in the photon number correlations N_{nm}. The photon number correlation is experimentally measured by the $g^{(1)}(R, -R)$ coherence, which shows an increase with pump power, that is also reproduced in our theory (Fig. 2d, details in SI Eqns. 16 and 17).

Unlike conventional lasers or polariton condensates, where spatial coherence is accompanied by spectral narrowing (temporal coherence), our system exhibits extended spatial order while temporal

coherence remains suppressed by rapid Purcell-enhanced decay. Our model reveals that local dipole phase-locking (Z_n) establishes itself prior to the spatial expansion of photon emission (the 'halo'). This sequential buildup distinguishes the synchronized dipole state from standard threshold-driven transitions in polaritons. The observation of spatio-temporal vortices indicates a unique regime of phase turbulence and slips that are inherently driven-dissipative and non-equilibrium, coexisting with localized synchronized regions.

We now make the explicit connection between synchronization and the Z_n parameter in the main text in the section "Coherence spreading and dipole-correlations" and in the "Discussion and Outlook".

The biggest concern is - why does it matter if there is synchronization at ultrashort timescales?

> We note the extensive discussion on this in the previous round of reviewing, which satisfies the other two reviewers.

More specifically here are some comments on the rebuttal:

1. The authors claim that the 2D model shows similar trend as experiments and 1D model. However the presented data does not validate that!

> We show our explicit comparison below. While the computational cost of the 2D model is high, simulations on 40x40 sites demonstrate that the qualitative features persist in two dimensions, specifically the emergence of spatial coherence under driving. The 2D model reproduces the characteristic spatial spreading of correlations observed in both the 1D model and experimental $g^{(1)}$ maps. We make this explicit below, by comparing corresponding results from both the 2D and 1D (in a new SI Fig. S4, compared below with Fig.S9). These clearly show nonlinear growth in the total photon number $\sum n_{nn}$ with increasing pump power $\Gamma_\uparrow/\Gamma_\downarrow$, spreading of the halo region, increase in the dipole polarization per site $\sum N_E p_n^\dagger$, and the corresponding increase in $g^{(1)}(R,\tau{=}0)$. All other simulation parameters are the same, details in SI Section S3.7. Some differences in the magnitude of the spread are attributed to the difference between coupling in 2D *vs* 1D, which is the subject of ongoing work.

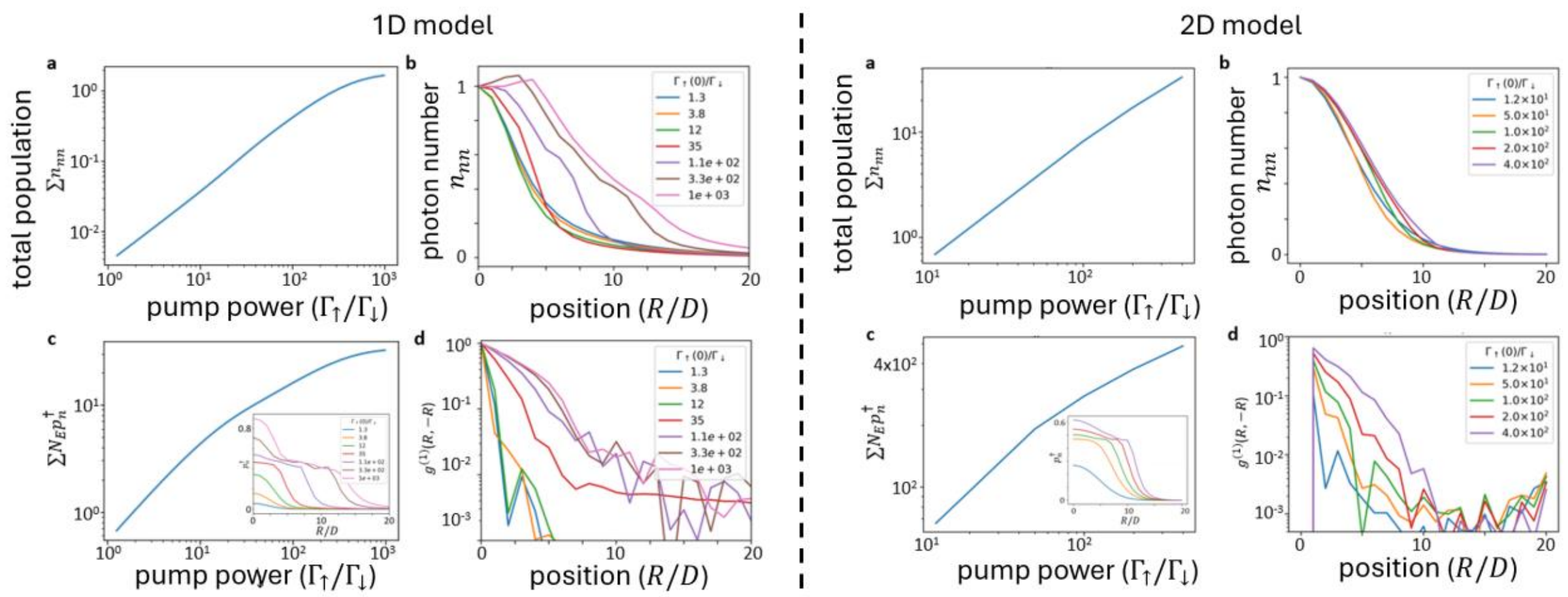

Fig.R1: **Tight-binding model coherence and photon correlation, for (left) 1D model in Fig.S4 and (right) 2D model in Fig.S9. a,** Input-output relation for total summed photon number n_{nn} against pumping rate $\Gamma_\uparrow$ normalized with non-radiative decay $\Gamma_\downarrow$. **b,** Spatially-averaged radial profile of photon number n_{nn} against position R/D for different pumping rates. D is the nanoparticle diameter. **c,** Sum of emitter polarization against normalized pumping rate $\Gamma_\uparrow$. Inset shows polarization *vs* position R/D for different pumping rates, with colors indicated by the legend in b. **d,** Spatial dependence of $g^{(1)}(R, -R)$ coherence for increasing pumping rate, showing spatial spreading & growth in coherence.

2. What is the physical interpretation of vortex formation in g1? Doesn't it imply loss of long range spatial order? How many such vortices do you observe in the experiments?

> Vortices in the $g^{(1)}$ map represent topological phase defects that emerge during the transient reconfiguration of the synchronized state. Rather than implying a total loss of order, their nucleation is a hallmark of phase-ordered systems (like quantum fluids) undergoing dynamical evolution [see main text Refs. 4, 12, 13, 29]. A varying number of spatio-temporal vortices are observed in the $g^{(1)}(x, y, \tau)$ data as marked in SI Fig. S21 and Fig. R2 in point #8 below, but typically only one traverses the central spot. The vortex evolution with measured delay time is highly unusual under continuous-wave pumping. The vortex is a phase dislocation between successive frames of the optical field 11 fs apart (in Fig. 5c). This is distinct from the usual vortices which freely evolve, which are not usually observed in time-averaged pulsed polariton experiments [main text Ref. 13].

3. For my second question, the authors discuss the coherence of white light source. That is not what I was asking. My question was: if you used a LED to pump the sample at the same intensity, would you observe spatial coherence as observed under CW laser pump? This is important to show as it will negate any role of the pump laser or its scattering on the observed coherence.

> We apologize for any previous misunderstanding, but we emphasize that the observed coherence does not originate from the pump laser coherence. We provide three distinct reasons why this is not the case, why we do not consider LED pumping, and why LED pumping would not produce these effects observed.

Observed effects are not due to coherence of the laser pump:

1. **Nonlinear power dependence of halo and $g^{(1)}$:** If our observation was an artifact of elastically scattered pump laser light, the spatial coherence would be observed linearly at all pump powers. However, as shown in our data (main text Fig. 2d and SI Fig. S19), spatial coherence is completely absent at low laser pump powers. The spatial coherence only emerges and spreads nonlinearly as pump power increases, confirming it is a collective density-dependent property of the emitters, not from the laser source or an LED source.

2. **Loss of pump phase memory:** The emission we observe is Stokes-shifted by 190 meV below the 1.96 eV pump (whose linewidth is 2 MHz, so < 0.1 meV). Following Kasha's rule, the molecular Frenkel excitons undergo rapid non-radiative vibrational relaxation before emission. This emission of vibrations is an incoherentdephasing process that fundamentally breaks any phase relationship with the incident pump laser. Furthermore, our detection path actively filters out the 1.96 eV pump with notch filters (spectra in Fig. 3d clearly show laser leak is suppressed by over 4 orders-of-magnitude), ensuring the measured $g^{(1)}$ signal originates entirely from the uncorrelated Stokes-shifted emission.

Experimental feasibility of LED-pumping:

3. **Limits of etendue:** Experimentally, pumping the sample with an LED at the same intensity ($\sim$100 μWμm^{-2}) and the same diffraction-limited spot size as our CW laser is restricted by conservation of etendue. An LED is an extended spatially-incoherent source. Reaching the requisite high-intensity excitation threshold to trigger synchronization in a nanoscale volume is not yet possible with LEDs or commercial superluminescent diodes, as demonstrated by the etendue comparison provided below. A CW laser is a spatially coherent point source with an etendue approaching zero, allowing full power focussing into a 1 μm^2 diffraction-limited spot. By contrast, an LED is an extended Lambertian emitter.

Even when utilizing state-of-the-art >100W commercial LEDs coupled through high-NA objectives, most of the emitted light is rejected by the objective small acceptance etendue (etendue $G \sim 0.19$). The fraction of light that *is* transmitted cannot be focused to a point, but instead fills the entire field of

view. This is because LEDs are an extended source (size $d_{\mathrm{LED}} \sim$ 3 mm) with an inherent divergence angle $\theta \approx d_{\mathrm{LED}}/f_{\mathrm{collimator}}$. Because the beam is not truly parallel, when it enters the back aperture of an objective lens, it is not focussed to a point. Instead, the objective focuses these diverging rays to create an image of the LED chip on the sample. Hence to get a small spot, a large magnification (M=3000) of the LED is needed, to shrink a 3 mm LED down to a 1 μm^2 spot. Since the 100X objective has a fixed focal length ($f_{\mathrm{obj}} \sim$ 2 mm), the collimating lens must have a focal length of 6000 mm, which captures only a tiny portion of the highly divergent LED light. To instead capture the full power of an LED requires sacrificing the spot size and using a short focal length collimator (eg. an aspheric condenser with $f_{\mathrm{collimator}} \sim$16 mm) placed near the LED, giving magnification is M = 16/2 = 8. The image of a 3 mm LED on the sample will thus be ~375 μm wide and completely overfill the field of view of the objective.

We summarize this in **Table R1** below for a range of LED sources. Attempting to pump our system with an ultra-high-power LED through our objectives yields an intensity that is roughly three to four orders-of-magnitude too low to reach the current experimental threshold.

Table R1: Comparison of light sources for pumping

Light Source	Microscope Objective NA	Objective Acceptance Etendue ($mm^2 \cdot sr$)	Source Total Power (W)	Source Diameter (mm)	Source Area (mm^2)	Source Full Angle (deg)	Source Etendue ($mm^2 \cdot sr$)	Source Brightness ($W/mm^2 \cdot sr$)	Theoretically Coupled Power (W)	Minimum Focused Spot Size (Area, μm^2)	Maximum Achievable Intensity ($mW/\mu m^2$)	Notes
Laser (Matchbox VBG SLM 633nm)	100X (0.9 NA)	0.097	0.07	0.01	0.0001	0.057	6.105E-11	1146605093	0.07	1	**70**	633 nm narrow
Cree XHP70.2	100X (0.9 NA)	0.097	29	4	12.57	120	29.609	0.979	0.095	38000	**0.0025**	425 - 730 nm
Luminus CBT-140	100X (0.9 NA)	0.097	100	4.22	13.99	120	32.955	3.034	0.294	38000	**0.0077**	425 - 750 nm
LDLS EQ99	100X (0.9 NA)	0.097	0.3	0.15	0.02	56	0.012	24.518	0.3	4800	**0.0625**	full power, assuming no spectral filter
										(full FOV)		
Laser (VBG SLM 633nm)	20X (0.4 NA)	0.478	0.07	0.01	0.0001	0.057	6.105E-11	1146605093	0.07	4	**18**	633 nm narrow
Cree XHP70.2	20X (0.4 NA)	0.478	29	4	12.57	120	29.609	0.979	0.468	950000	**0.0005**	425 - 730 nm
Luminus CBT-140	20X (0.4 NA)	0.478	100	4.22	13.99	120	32.955	3.034	1.450	950000	**0.0015**	425 - 750 nm
LDLS EQ99	20X (0.4 NA)	0.478	0.3	0.15	0.02	56	0.012	24.518	0.3	24500	**0.0122**	full power, assuming no spectral filter

4. My third comment also seems to have been misunderstood. I was asking about the short radiative/non-radiative excited state lifetime due to the plasmonic coupling contributing to the observation. i.e. the excited state lifetime is so short due to plasmonic coupling that there is not enough time to undergo decoherence.

> We agree that the plasmonic enhanced emission lifetime allows emission before decoherence. However, synchronization here is not merely a result of the emission occurring "too fast to decohere". If the effect were solely due to Purcell-shortened lifetimes, the coherence would be independent of pump power. Instead, we observe a clear power-dependent increase in spatial $g^{(1)}$ in main text Fig. 2d (at all spatial locations), which is a defining signature of a driven synchronization process where dipoles actively lock their phases through coupling. We note further that ultrafast emission on its own would ultimately lead to less coherence due to lifetime broadening.

5. The polarization experiments (Fig. S35) seem to indicate that it follows the incident laser polarization although the degree of polarization is lower which is expected. However the emission is clearly not isotropic. If the laser polarization is rotated, does the PL also follow?

> Emission follows the incident laser polarization because the local nanogaps (hotspots) are preferentially excited when their long axis aligns with the laser E-field. While the degree of polarization is lower due to the random orientation of nanogap segments within the aggregate, the emission follows the rotation of the incident laser polarization, as we show in the new Figure S35 below, with polarization of emission sampled over 10 different locations on the sample.

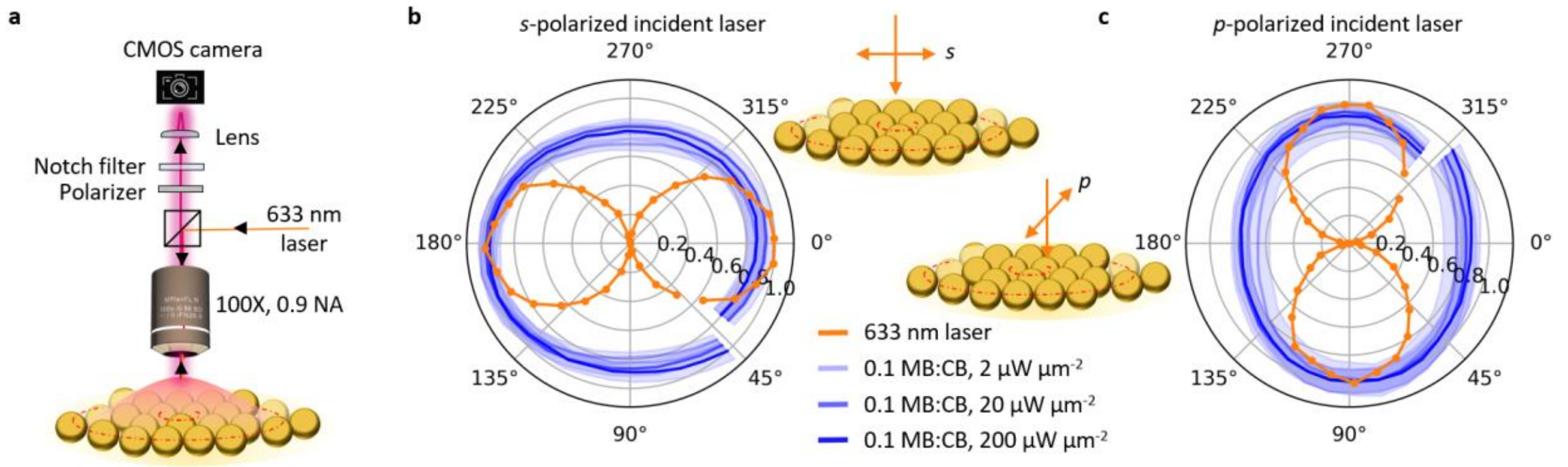

Supplementary Figure S35: **Emission polarization measurements**. **a**, Emission polarization setup: wire-grid polarizer measures emission polarization on CMOS camera. **b**,**c** Total normalized emission intensity *vs* polarizer angle for the pump laser control for (b) *s*-polarized and (c) *p*-polarized excitation. MB:CB=0.1 sample at three different pump intensities below/above synchronisation, over 10 locations (shaded area = standard deviation).

6. For the question on scalability, the authors comment: "This is the key nanotechnology advance: a soft matter, self-assembled platform showing synchronization of quantum emitters." Once again maybe I am not aware, what could one do with such short lived synchronization?

> The significance of our work lies not in the *duration* of the coherence, but in the fact that any collective phase order can be established at all within a "bad-cavity" system dominated by extreme losses. While traditional lasers and condensates require long-lived photons and narrow-linewidth emitters, our system demonstrates a qualitatively different route to optical phase order compatible with lossy, broadband, and nanoscale architectures. We reiterate that this platform offers several advantages for nanotechnology:

- **Robustness to Noise**: Operating in the bad-cavity limit suppresses "cavity pulling," making the synchronization robust against the environmental noise, temperature drifts, and structural disorder that typically degrade high-Q microcavities.
- **Sensing**: The $g^{(1)}$ maps and vortex statistics provide new observables sensitive to local dephasing and coupling, enabling "phase-based" detection of molecular changes.
- **Ultrafast Extraction**: Purcell-shortened lifetimes allow for photon extraction on timescales faster than slow environmental dephasing (e.g., spectral wandering), which is a key requirement for improving the indistinguishability of photons in ambient quantum emitters.
- **Scalable Architecture**: Because this synchronization emerges from self-assembled plasmonic networks at room temperature, it removes the need for microfabrication, epitaxy, or cryogenic cooling, enabling scalable integration into sensing chips and functional coatings.

7. I am glad the authors repeated the experiments with lower magnification objective (20X) and have shown the data in Fig. S39. However, I do not see the "halo" formation here. Am I missing something?

> Indeed underlying nonlinear growth of the emission area is consistent across magnifications. We do see a halo region (halo width now explicitly shown in SI Fig. S40, and below). We emphasise that there is nonlinear growth in the emission area, unlike that of the Gaussian laser spot which would precisely overlap when normalized, as is seen at low pump powers but not at high powers. We now explicitly describe in the main text that the emission 'halo' is still present with a less tightly focused pump.

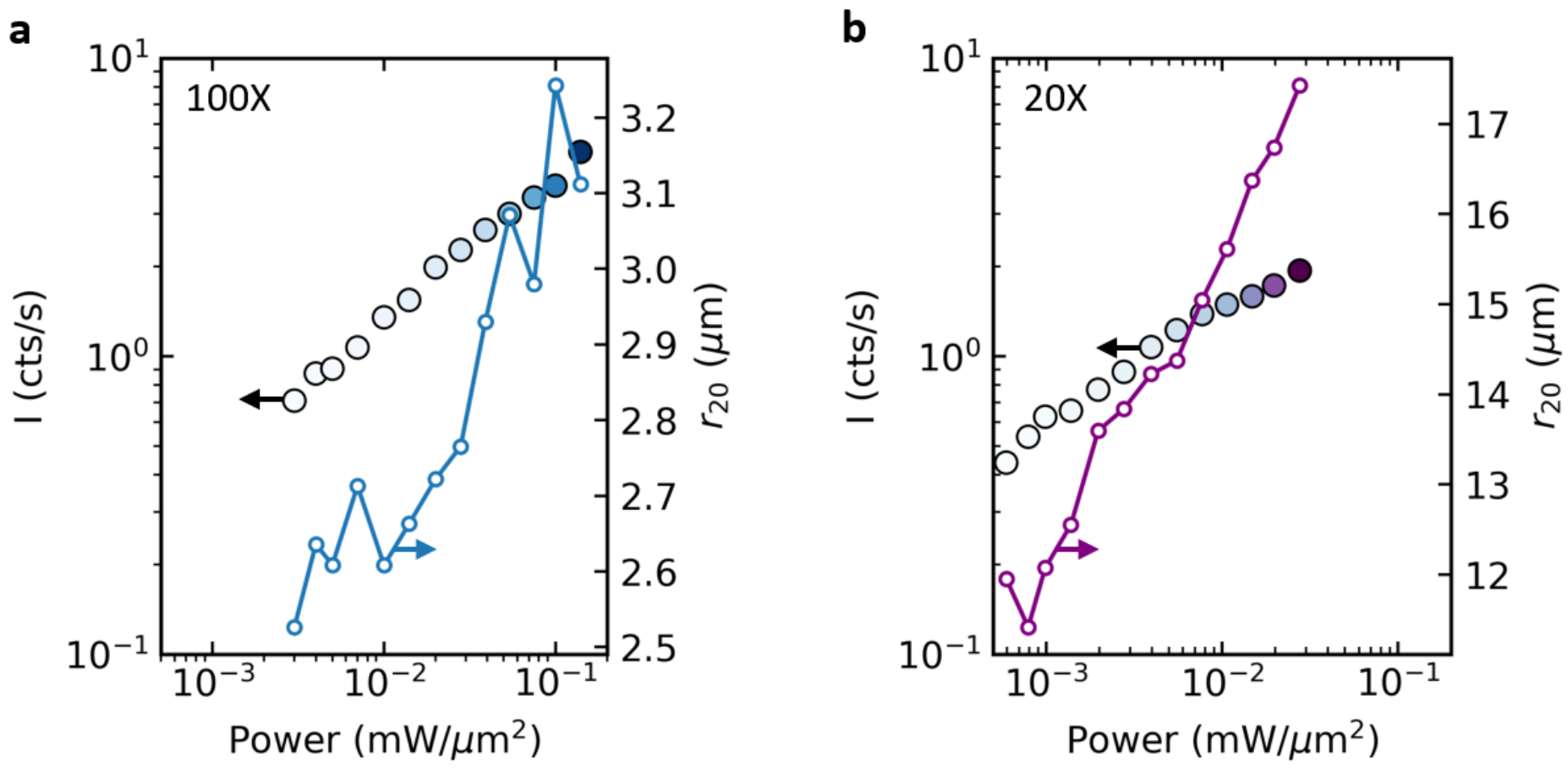

Supplementary Figure S41. Measurements comparing x20 and x100 objectives. **a,** Total emission intensity and spatial size (radial position where normalized intensity drops to 20%) *vs* pump power for MB:CB=0.1 with a 100X 0.9NA objective lens. **b,** Same as (a) with instead a 20X, 0.4NA objective lens. Colours corresponding to spatial profiles shown in SI Fig. 40.

8. The authors suggest two mechanisms for the collapse-revival feature of coherence. The second one sounds interesting - i.e. transient reconfiguration of spatial phase domains. However, what is puzzling is the periodicity of this collapse-revival phenomena (Fig. 2e). I would have imagined the latter effect to result in random locations for such collapse-revival to happen.

> As noted in the text our results are indeed intriguing, as the reviewer acknowledges, but details are not yet fully explained. We suggest that the periodic revival may be driven by spectral beating between distinct components of the emission (main electronic transition and resonance-Raman features), and/or transient reconfiguration (vortex formation) acting simultaneously to influence the amplitude and spatial phase structure of the revival. We also show the random locations for the collapse-revival clearly in additional SI Figs S32, S33, and S34 *vs* Fig. 5c. Not all decays show the same periodicity, the transient reconfiguration does not occur in all locations, and vortices do not nucleate in the same place each time. We reproduce this below in **Fig. R2**, showing the relevant panels and vortices.

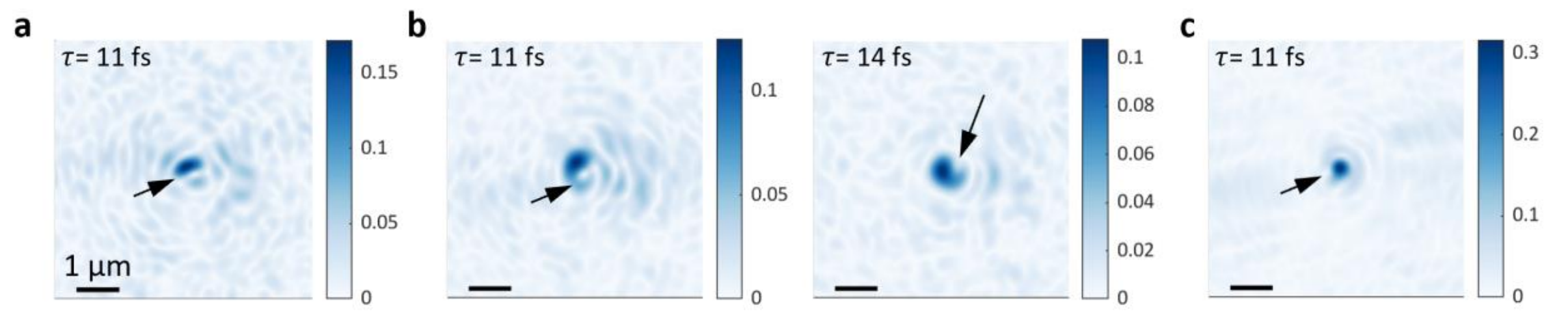

Fig. R2. Spatial maps of $g^{(1)}$ at different times, with arrows indicating the vortex location as a null in the field from a, SI Fig. S33. **b,** SI Fig. S34. **c,** SI Fig. S34. **d,** SI Fig. S35.

In summary, I am still not convinced that this work represents the kind of advancement that warrants publication in Nature Nanotechnology. The authors tend to use esoteric and complex theoretical ideas to interpret their experimental results. In the end what the authors seem to have is a fluctuation-dominated regime where the phase coherence is destroyed both spatially and temporarily in short time scales.

> While we agree that the language used in the first submitted version was perhaps too heavy with jargon, we substantially revised this after the first round of revisions, and further in the current

revisions, as suggested. Our work uses well-established tools in open quantum systems to describe the behaviour, and shows a nice match between theory and experiment. While fluctuations are present in any driven-dissipative system, our data reveals a clear **power-dependent evolution** to a synchronized state, at all sites.

We emphasize again that the clear novelty is we have a synchronization transition, which we clearly define by an increase in $g^{(1)}$ and halo size with pump power, *despite* the presence of disorder in the extreme bad-cavity deeply sub-wavelength plasmonic regime where long-lived coherence and linewidth narrowing is fundamentally impossible due to ultrafast radiative and non-radiative decay.

Evidence of Order: If the system were merely a fluctuation-dominated incoherent source, the spatial extent of the $g^{(1)}$ coherence would remain constant. Instead, we observe a nonlinear spatial expansion of the 'halo' of coherence as pump power increases, reflecting the active spread of phase correlations across the dipole population.

Collective Response: The observed spatial coherence arises specifically from dipoles synchronizing their phases through near-field plasmonic coupling, matching established state-of-the-art theoretical simulations. This represents a transition from independent emitter fluctuations to a collective, phase-ordered response that is the hallmark of synchronization, regardless of the dissipation rate.

Reviewer #3:

1. The rebuttal by Arul et al. is very extensive and answers many of the questions that were raised by the referees. I am still somewhat unsure about several of the claims, such as for example the interpretation of the vortices (referee #2 found this very interesting) in the g^1 map, which seems confusing (also for referee #2).

> We appreciate the reviewer's interest and support. We are happy to clarify what is understood about the vortices further.

The vortices we observe are spatio-temporal phase dislocations in the $g^{(1)}(R,-R,\tau)$ coherence map. The vortex is a phase dislocation between successive frames of the optical field 11 fs apart (in Fig. 5c). Their interpretation is rooted in the hydrodynamic description of quantum fluids [main text Ref. 13], where the velocity field of the synchronized state is defined by the gradient of its phase:

1. **Phase Winding**: The vortices represent points of zero visibility where the phase of the emission winds by 2π around a central defect. This is a topological signature that the system has established a degree of global phase order that is susceptible to topological excitations.
2. **Transient Reconfiguration**: The vortices represent the **transient reconfiguration** of spatial phase domains and not a loss of order. They typically nucleate near the first minimum of the temporal $g^{(1)}$ decay, marking the moment when different parts of the synchronized ensemble are adjusting their relative phases.
3. **Experimental Observation**: We have provided additional data (SI Section S7.1) showing that these vortices are persistent across different sample locations, though their exact nucleation time is not fully deterministic, a characteristic common to vortices in other polaritonic systems undergoing dynamical phase evolution.

By mapping these vortices, we gain a direct observable for how dipoles interact and dephase, offering a new diagnostic tool for monitoring the internal dynamics of coupled nano-systems. We now include a further discussion in the main text section "Vortices and non-equilibrium behaviour" clarifying this. We also note that the precise explanation for these vortices is not yet known, and we agree with the reviewer that this paper will "stimulate further experimental and theoretical research in this direction".

2. Also the spectrum that is now shown in the sup mat is more complicated than I can expect to follow from the theoretical model. It is stated by the authors that computing the different time correlators is beyond the current scope.

> The emission spectrum is indeed more complex than the simple two-level model used for the initial theory, however modelling spectra and two-time correlations require advances in theory. The cumulant expansion method used to solve the coupled system cannot compute spectra, and efforts are underway to develop this method further, but as yet this is a limitation at the frontier of solving open quantum systems with the cumulant expansion. Hence, we used conventional curve resolution to understand the experimental spectra in Fig.3d, S25.

3. Looking again at the experimental data and interpretation on the growth of the g^1(R=0), I do now indeed agree that it indicates the growth of spatial coherence. I do not think that the argument on the spatial averaging is entirely correct though. I think that it is rather the size of the interference fringes (on about the same scale) that limits g^1(R=0).

> The reviewer is happy with our robust identification of growth $g^{(1)}$, but suggests that the size of interference fringes may limit its magnitude. While we agree that fringe resolution is a factor in interferometer calibration, the measured growth of $g^{(1)}(R=0,\tau=0)$ with power remains a valid indicator of increasing phase order within the dipole population. The fringe spacing is still smaller than the spot size, hence its contrast is not limited by this.

4. I think that in the current state and with the extended discussions, the paper is now sufficiently sound for publication in Nature Nanotechnology since it can stimulate further experimental and theoretical research in this direction.

> We thank the reviewer for their support and suggestions which improved the clarity and impact of this work.
